# Supplementary material for: Inadequate Regulation of the Geological Aspects of Shale Exploitation in the UK
Source: Int J Environ Res Public Health. 2020 Sep 23;17(19):6946. doi: 10.3390/ijerph17196946 (PMC7579502; doi:10.3390/ijerph17196946)
Supplement: Supplementary file 1 [file ijerph-17-06946-s001.pdf]

Supplementary information for Inadequate regulation of the geological aspects of shale exploitation in the UK

David Smythe

Contents Page 1

Figure S1 2

Figure S2 3

Figure S3 4

Figure S4 5

1. Aminzadeh et al. 2013 extract 6
2. A note on licensing regimes outwith the UK 14
3. Smythe comments 2015 on letter from DECC to LCC 15
4. Case histories of relationship between BGS and government 20
5. Cuadrilla at Balcombe 21
6. Canonbie 23
7. Airth 2014 24
8. Micrites in the Kimmeridge Clay Formation 28
9. Europa at Leith Hill 30
10. Celtique Energie in the Weald 42
11. IGas at Springs Road, Misson 49
12. Angus Energy at Brockham 55
13. UKOG at Broadford Bridge 53
14. UKOG at Horse Hill 61
15. Lancashire 2009-2012 66
16. Correspondence between the EA and local resident 2014 68
17. Sherwood Sandstone aquifer below the western Fylde 71
18. Manchester Marls as a seal 75
19. Smythe objection 2017 to PNR variation of permit for microseismic monitoring 77
20. Smythe objection to approval of PNR-2 hydraulic fracture plan 83
21. Meeting Balcombe PC and Cuadrilla 2013 100
22. Minutes of SEPA Enzygo Greenpark Meeting 2011 103
23. Selley 2014 response to SDNPA letter of 24 June 2014 105
24. Smythe comments upon report to SDNPA by Prof. Selley 113
25. OGA response to Sutcliffe letter 7 July 2016 120
26. BGS comments 2013 to scoping opinion, Grange Rd 124
27. Charles Hendry (DECC) - Cuadrilla correspondence 2012 125
28. EA response to Estelle Dehon 22 July 2019 129
29. IGas letter 2017 to MPA Ellesmere Port-1 well 134

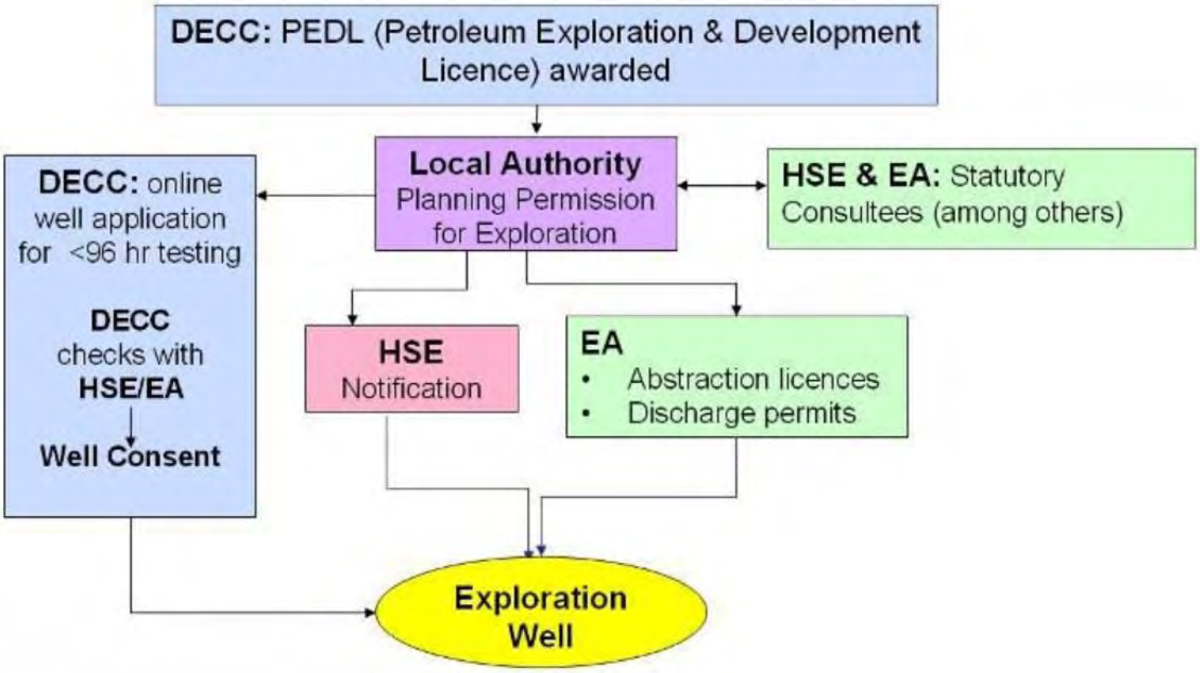

Figure S1. Who does what at the exploration stage. From:

Potts, C. 2013. Oil and gas development: planning perspective. South Downs National Park Authority presentation, 15 October 2013.

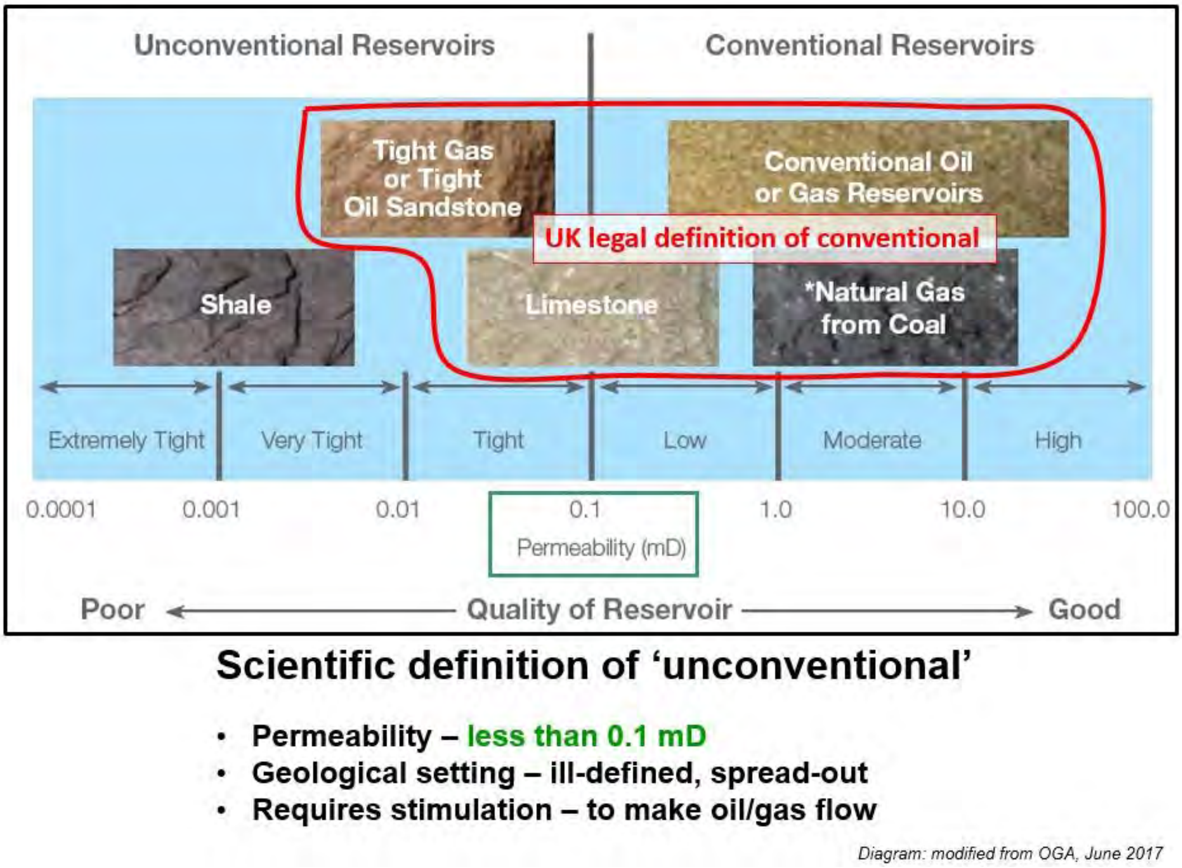

Figure S2. Scientific definition of an unconventional hydrocarbon resource as <0.1 millidarcies. The UK legal definition (red outline) of conventional resources encompasses rock type reservoirs falling into the unconventional category.

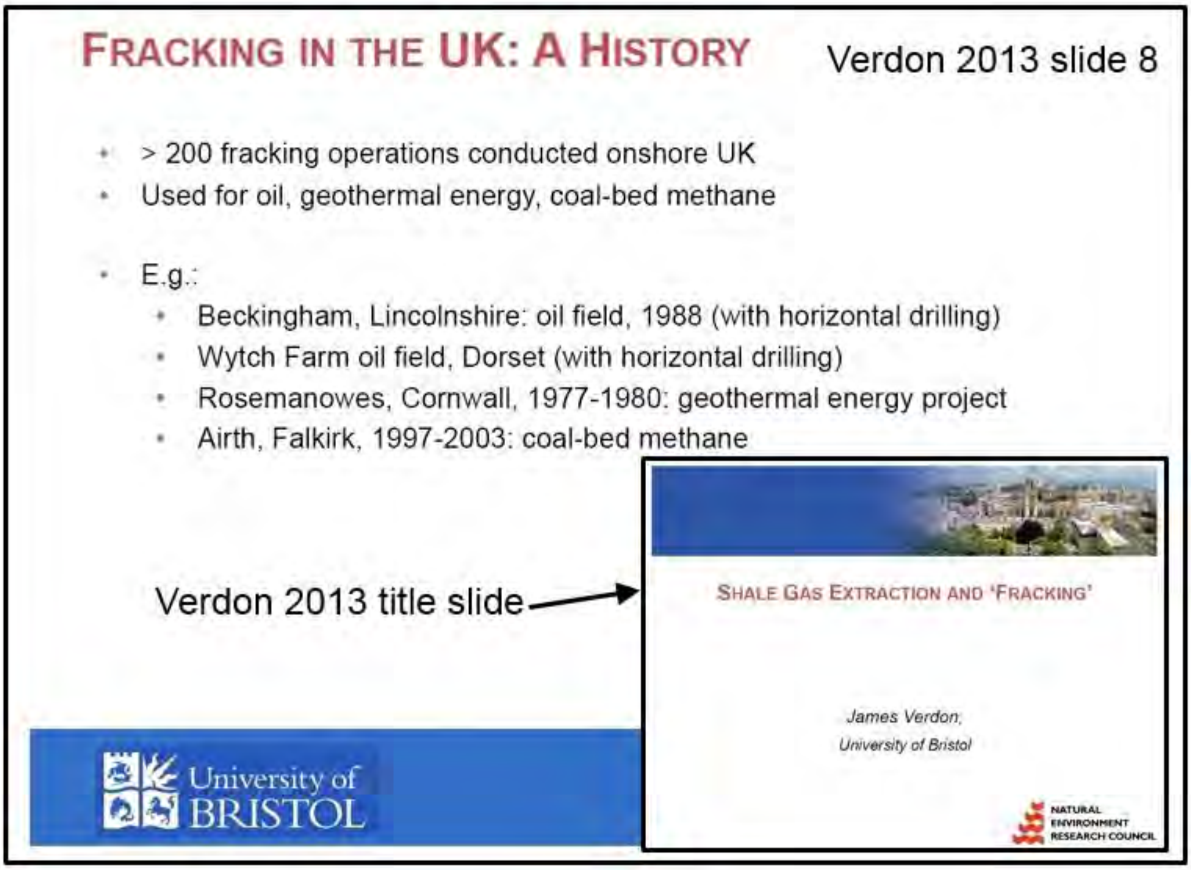

Figure S3. Slide from talk by Dr James Verdon at Glastonbury, 2013, wrongly illustrating Wytch Farm as an example of fracking with horizontal drilling. In fact the sandstone reservoir was drilled horizontally, but there has never been any fracking at Wytch Farm.

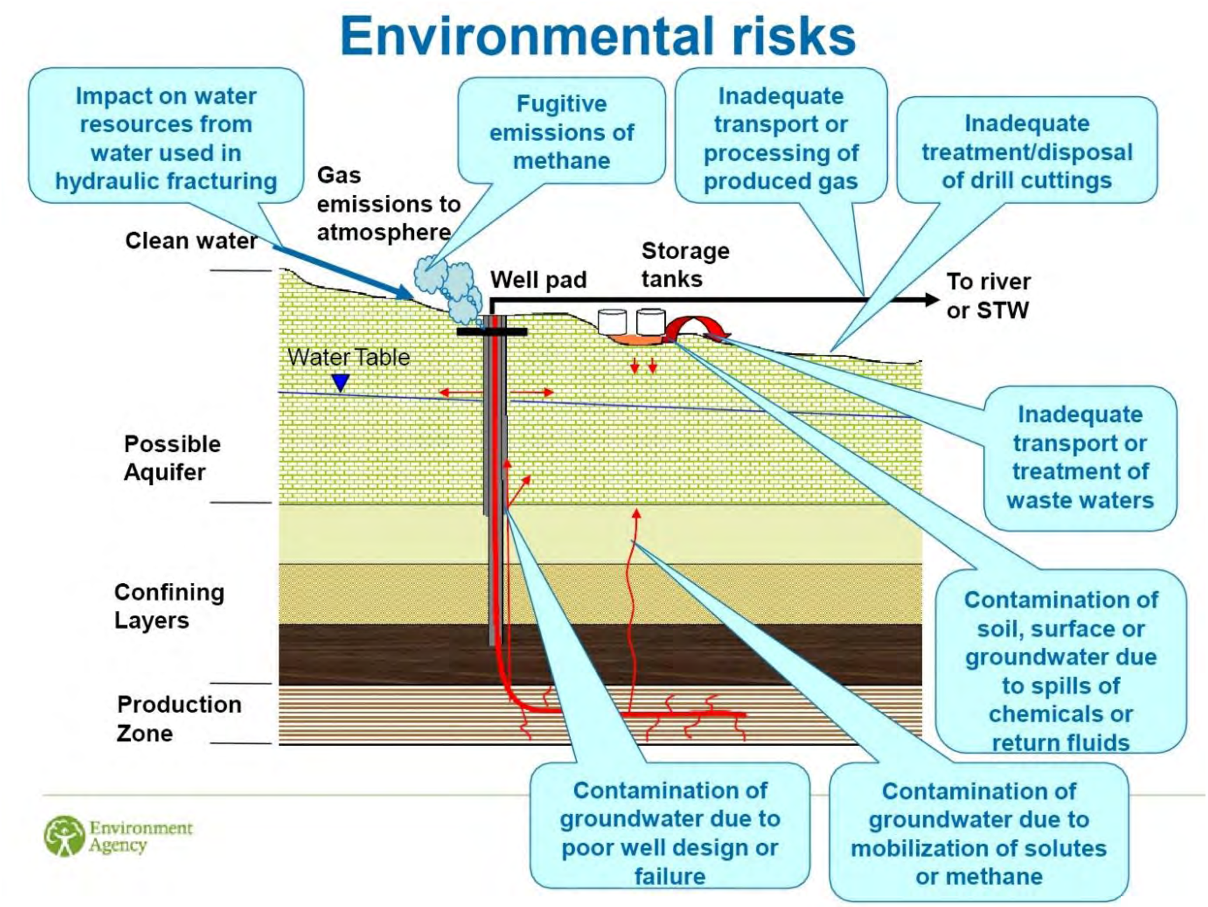

Figure S4. Diagram of environmental risks from fracking. From:

Davey, I. *Water environment risks from shale gas exploitation*. EA submission to RR2012, 23 March 2012.

Hydrocarbon seepage examples

Selected pages from:

Aminzadeh, F. *et al.* (eds.) 2013.
Hydrocarbon Seepage: From Source to Surface
Society of Exploration Geophysicists and American Association of Petroleum Geologists, 244 pp. ISBN 978-1-560-80-310-2.

Seven pages have been reproduced under the US doctrine on fair use (https://www.copyright.gov/fair-use/more-info.html).

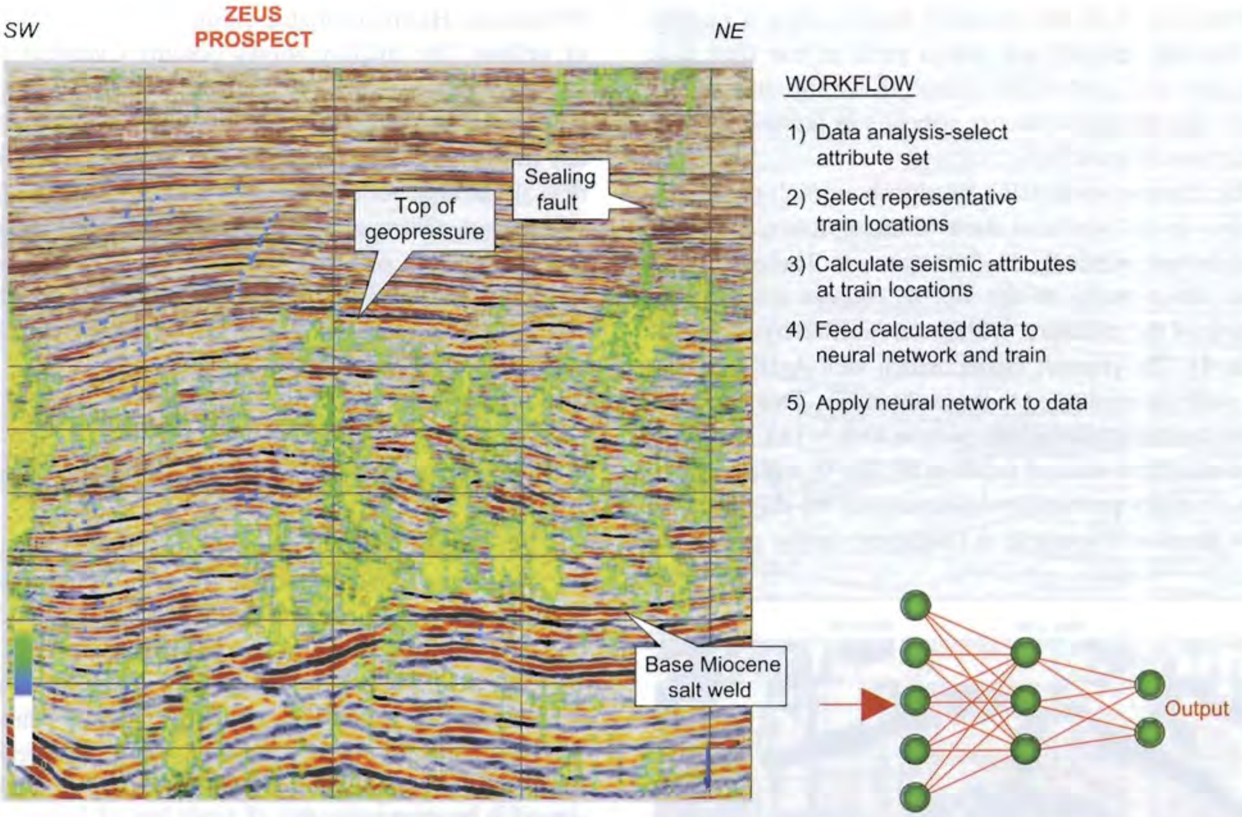

Figure 2. Gas-chimney processing workflow and results displayed on arbitrary line A–A′.

When the chimney processing results are overlain on an arbitrary southwest–northeast-trending line (Figure 2), we notice three things:

1) The chimneys originate at or below the salt weld; this corresponds to the Eocene and Cretaceous source-rock intervals.
2) The chimneys are often associated with shallow amplitude anomalies, which are usually pay.
3) The chimneys are often (but not always) associated with low-velocity zones.

To assess the quality of the chimney volume and to check for false positives and false negatives, we performed a quality-control and validation step. Initial validation was done by visual inspection. An in-depth validation was then done as part of the second phase of the project, including validation based on a set of criteria such as surface seepage, mud-logging shows, and association with direct hydrocarbon indicators. Mud-log and core data were available for well A-60 and were used during the validation step.

The next step was to interpret the chimney data. One way of doing this is to overlay the chimney time or depth slices (yellow to green) on fault time slices (gray scale). In this way, we can observe the major gas chimneys but also, more interestingly, distinguish leaking faults from those that may be sealing faults. When we overlay the chimney data on a time slice of the similarity data (Figure 3), we note that the chimneys have characteristic pockmark morphology. Major chimneys tend to occur at major fault intersections.

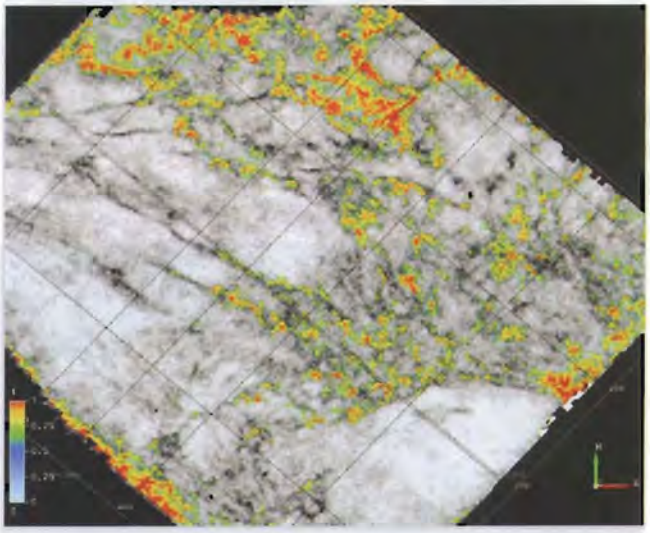

Figure 3. Chimney probability overlain on similarity attribute 2.628-s TWT time slice.

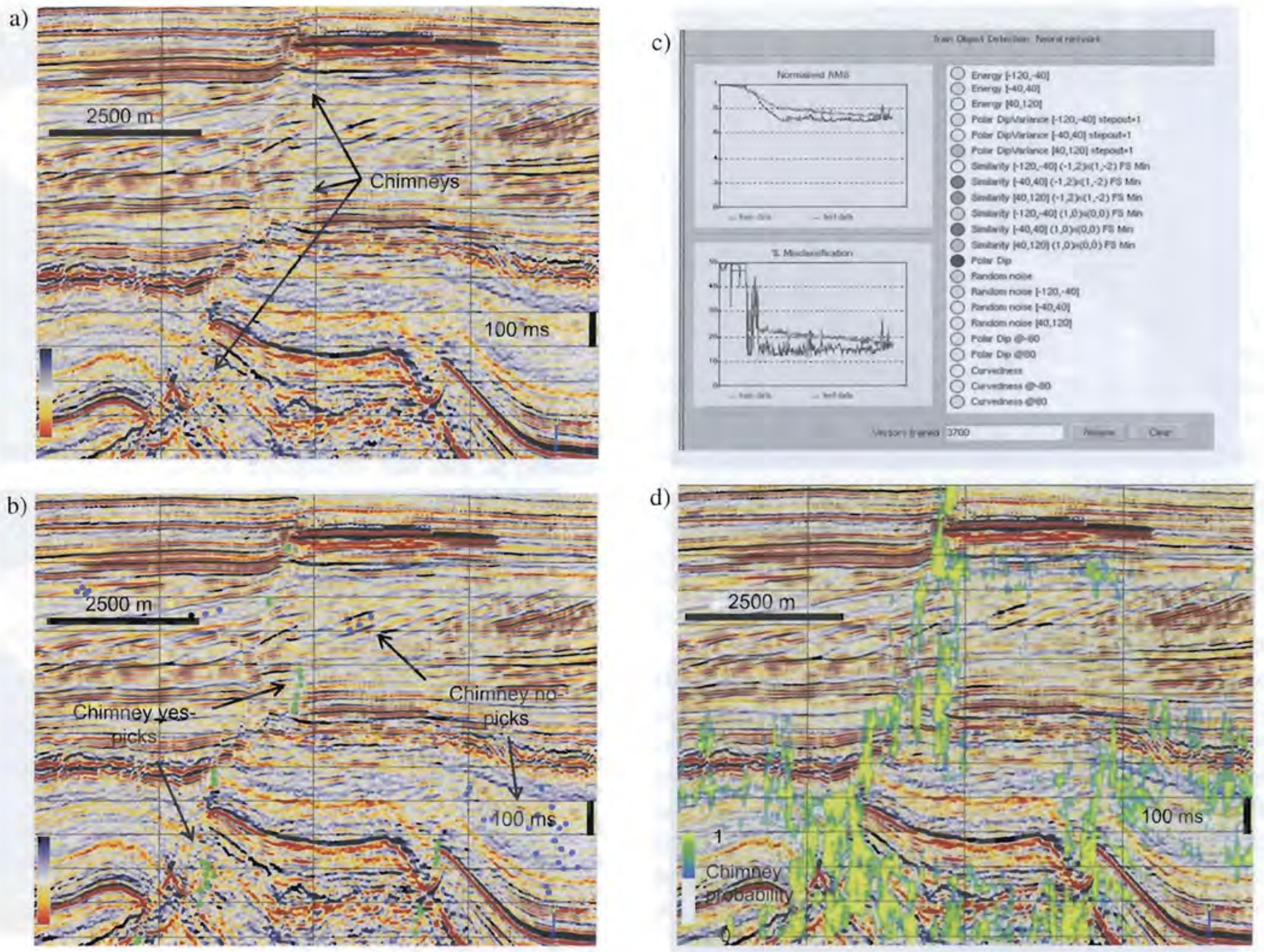

Figure 3. Chimney-detection methodology. (a) Chimneys are detected on key lines. (b) Examples of chimneys (green) and non-chimneys (blue) are picked on key lines. (c) A set of attributes is selected and calculated at picked sites. Graphs show training performance of MLP network and the percent error for the training and test sets. Input attributes with gray scale coding reveal the importance of each attribute (dark nodes are most important). (d) Results are displayed on key seismic lines. High-probability chimneys are yellow to green; low-probability chimneys are transparent.

The output of the neural network is a set of numbers between approximately zero and one, giving the probability of any point in the data volume to be or not be a chimney. The closer the value is to one, the more probable it is that the location belongs to the gas chimney. Results are evaluated interactively on key lines and slices to assess the performance of the neural network (Figure 3d), with possible iterations of the workflow updating the seismic attributes and example picks to increase the quality of the neural-network map. When the interpreter is satisfied with the performance of the neural network, the algorithm is applied to the entire volume and stored for more rapid visualization and analysis. These chimney-probability results can also be displayed on mapped horizons (Figure 4a), along mapped fault surfaces (Figure 4b), or on time or depth slices. In addition, the results can be displayed in 3D form (Figure 5a) or as geobodies (Figure 5b).

Validation of gas-chimney processing results

After a chimney-probability volume is generated, the results must be interpreted to validate them as true vertical hydrocarbon migration and to determine if they originate from a biogenic or thermogenic source. Vertically aligned chaotic data can occur for reasons other than gas chimneys. Certain geologic features and poor seismic imaging can also cause a low-amplitude chaotic seismic signature that looks like a gas chimney or gas cloud. It is

Visualizing gas-chimney-processing results

A major benefit of chimney processing is the potential to visualize the results in three dimensions. We can show the areal extent and origin of hydrocarbon charge into a prospect. We can also show the areal extent, vertical extent, and morphology of leakage from that reservoir.

Chimney-processing results can be visualized in a number of ways. One way is as *semitransparent overlays on seismic sections*. Chimney results are displayed on key dip or strike seismic lines (Figure 3). Seismic lines should be shown uninterpreted and overlain with chimney-probability results. This type of display is critical to show the seismic character of the detected chimney. Key seismic lines need to be oriented such that they transect the crest of the suspected trap as well as stay within the reservoir.

Another method is chimney results *displayed on horizon surfaces or time (or depth) slices* (Figure 4). Interpreted surfaces should be extensive enough to show any fault-related chimneys. Time or depth slices often show the chimneys more clearly than horizon slices because horizons are often mapped on strong, continuous seismic events that may obscure more subtle chimneys. Horizon surfaces and time slices show the pockmark morphology of the chimneys more clearly than a 3D display but do not show the origination of the chimneys or the vertical extent of the leakage.

A 3D *volume rendering*, shown in Figure 5a, is a third method. Generally, only the very high-probability results are displayed.

Finally, *extracted geobodies* (Figure 5b) can be classified into different groups, based on the objective of the interpretation. Thermogenic chimneys can be distinguished from biogenic chimneys; chimneys can be classified by their reliability. Reservoir geobodies can also be visualized with the chimneys with which they are in direct communication.

Applications in basin analysis and prospect risking

Detecting hydrocarbon migration related to faulting

Gas-chimney detection methods originally were used to highlight subtle vertical seepage pipes, mud volcanoes, and gas clouds. However, workers soon recognized that the methodology could be used to highlight more subtle hydrocarbon migration related to faulting and thus be used in assessing fault-seal risk (Heggland, 2002, 2005; Ligtenberg, 2003, 2005; Ligtenberg and Connolly, 2003).

The method consists of combining chimney- and fault-detection volumes to determine which faults, or portions of faults, have had associated active vertical hydrocarbon migration. Faults can be highlighted using several approaches. Single multitrace attributes such as coherency or similarity can be utilized. Also, faults can be enhanced using a neural-network supervised training approach, similar to chimney processing (Meldahl et al., 2001) or the neural-network approach, whereby examples of faults and nonfaults are picked on representative lines. Then a set of multitrace attributes is chosen which highlights the picked faults most clearly. These attributes are calculated at the picked locations, and the results are fed into a neural network. The resultant output algorithm is applied to the entire seismic volume to create a probability-of-fault volume.

The fault-attribute or neural-network volume can be displayed on seismic sections but is more useful when displayed on seismic time slices or key horizons (Ligtenberg, 2005). If the chimney-probability results are displayed over the fault-probability results, we can get an indication of which faults have been migration pathways for hydrocarbons and which faults have not (Figure 7). Figure 7a shows a time slice of the fault cube in grayscale with high-probability chimneys overlain (green to red = moderate to high). Large, circular chimneys can be recognized on these time slices (x). Chimney processing allows us to image more subtle hydrocarbon migration, which occurs at fault intersections. These intersections can occur at the junction of major faults (y) or at the intersection of a major fault with splinter faults (z). The splinter faults are inferred to be related to shear along that fault. Gartrell et al. (2004) suggest that these fault intersections may be one of the most important pathways for hydrocarbon migration in a basin. Their studies of the mechanical behavior of fault intersections using numerical modeling indicates that a dilation zone is formed at fault intersections where there are high concentrations of open faults and fractures. The shear strain at these intersections is very low, resulting in reduced fault gouge and higher fluid flux.

When the chimney data are displayed on the mapped fault surface (Figure 7b), we can observe which parts of the fault are migrating hydrocarbons. In this case, high-probability chimneys occur primarily at bends or kinks in the fault. Variations in plane orientation are very common in faults and can be defined as the roughness of the fault zone (van der Zee, 2002). When there is a strike-slip component to the movement of the fault, bends in the fault can create the dilation and/or the splinter faults that become preferential migration pathways for hydrocarbons. If these high-probability chimneys are observed in three dimensions

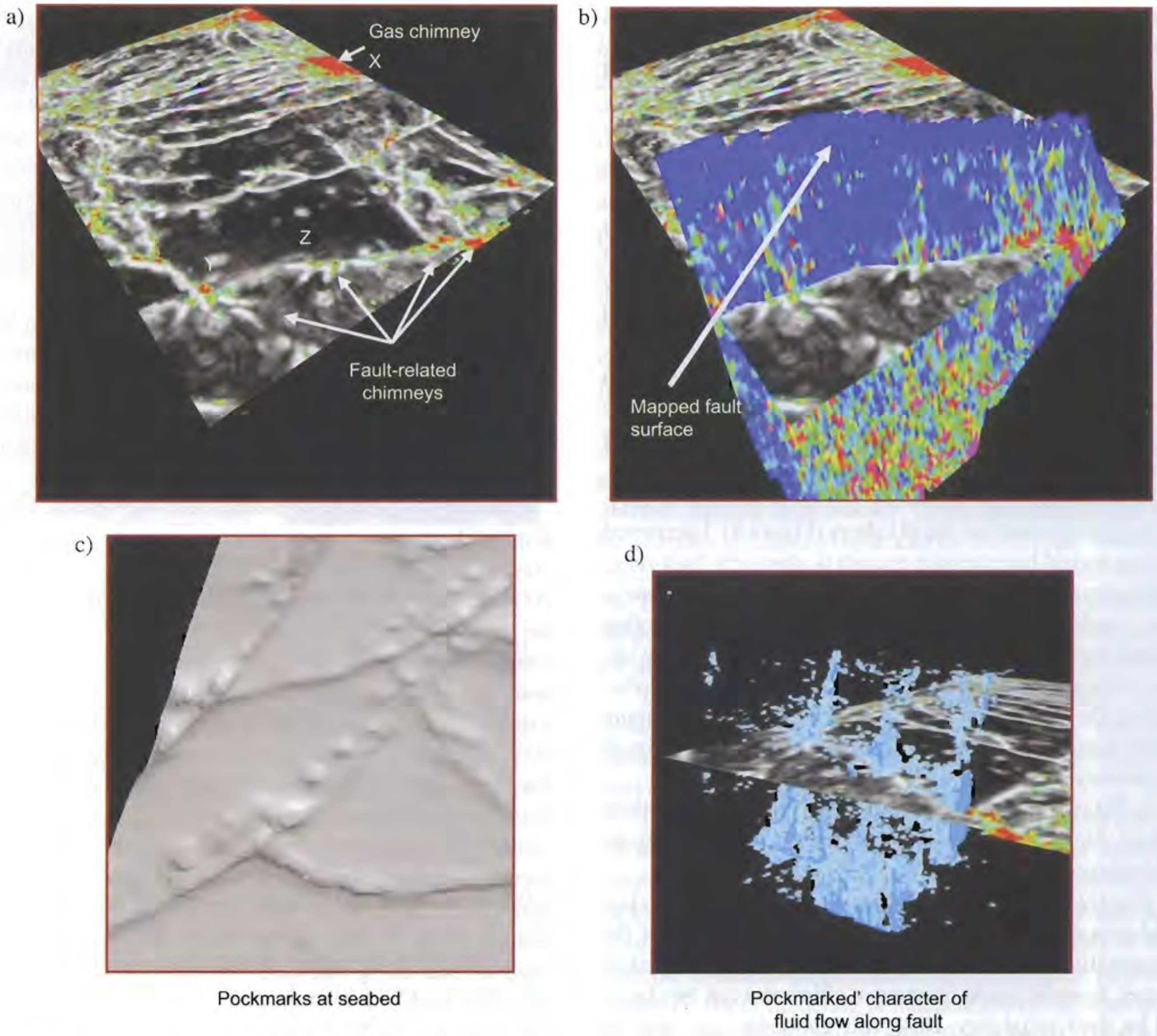

Figure 7. (a) Chimney-probability results overlain on fault attribute (grayscales). Chimneys occur frequently at major fault intersections and intersections with splinter faults. (b) Chimney-probability results (green to red = moderate to high probability) overlain on fault surface. Chimneys are observed at bends in fault. (c) Dip-azimuth horizon at surface shows pockmarks associated with hydrocarbon flow. Hydrocarbons are confirmed by piston coring. (d) A 3D visualization of chimneys, showing morphology of chimney. Green to red = moderate to high probability (adapted from Ligtenberg, 2005).

(Figure 7d), they appear as regularly spaced pockmarks. Ligtenberg (2005) relates these regularly spaced pockmarks to diapiric fluid flow, whereby minor weaknesses in the fault are concentrated and propagated in a vertical direction. Regularly spaced pockmarks are observed at the seafloor (Figure 7c). In addition, piston-core data confirm the presence of oil and gas in similar pockmarks in the Niger delta (Graue, 2000).

It is important to note that the detection of hydrocarbon migration pathways related to faulting does not tell us when that fault was leaking. The residual gas saturations and vertical fracturing related to the hydrocarbon migration leave an imprint on the seismic data. Thus, we can infer that the fault has leaked, but it may not be leaking today. Basin modeling can provide an idea of when hydrocarbons were actively generated in the basin. Timing can also be inferred from fault-stress analysis, an important tool to assess the failure potential of a fault (Zoback, 2007; Langhi et al., Chapter 2 in this volume). Thus, when there is evidence of fault leakage from the chimney analysis, it is critical to use fault-stress analysis to assess fault-seal risk.

Detecting hydrocarbon expulsion from source rocks

Modeling petroleum systems accurately in a basin requires a good understanding of the areal distribution of source rocks, the stratigraphic intervals in which source rocks occur, and their thermal maturity. Models depend on assumptions about source-rock presence based on regional outcrop information or limited well information because wells are rarely drilled in the hydrocarbon kitchen lows. Modeling must also be based on estimates of regional heat flow, which have a high degree of uncertainty.

In very-low-permeability source rocks, the pore pressures are quite high; thus, fracture generation is the primary mechanism of expulsion (Palciauskas and Domenico, 1980). These incipient fractures tend to propagate vertically due to buoyancy unless low-pressure, extensive permeable reservoir intervals are encountered. Hydrocarbon-saturated fracture zones show up in the seismic record as gas chimneys. This is especially true when the primary phase is gas or live oil, in the later stages of hydrocarbon expulsion.

From these theoretical considerations, we would expect to see gas chimneys above (and occasionally immediately below) thermally mature source rock. Critically, these chimneys should be absent over rich source rocks that are not thermally mature. Thus, if we can detect the stratigraphic interval from which the chimneys originate, that interval can be inferred to be a potential source rock. The lateral extent of the chimneys can then provide clues to the extent of the thermally mature kitchen or the lateral extent of organic-rich source rock (Ligtenberg and Thomsen, 2003).

An example from the Texas state waters of the GOM is shown in Figure 8. In this area, chimneys are clearly observed originating from the Eocene (Wilcox) interval, associated with a major décollement. The origin of these chimneys corresponds closely to the postulated gas-prone Eocene source-rock interval. There is no evidence for chimneys from the deeper Cretaceous oil-prone source-rock interval. Chimneys originating in the Eocene supports the observation, based on piston-core data, that the Texas shelf is dominated by a lower Tertiary gas-prone terrestrial hydrocarbon system (Hood et al., 2002).

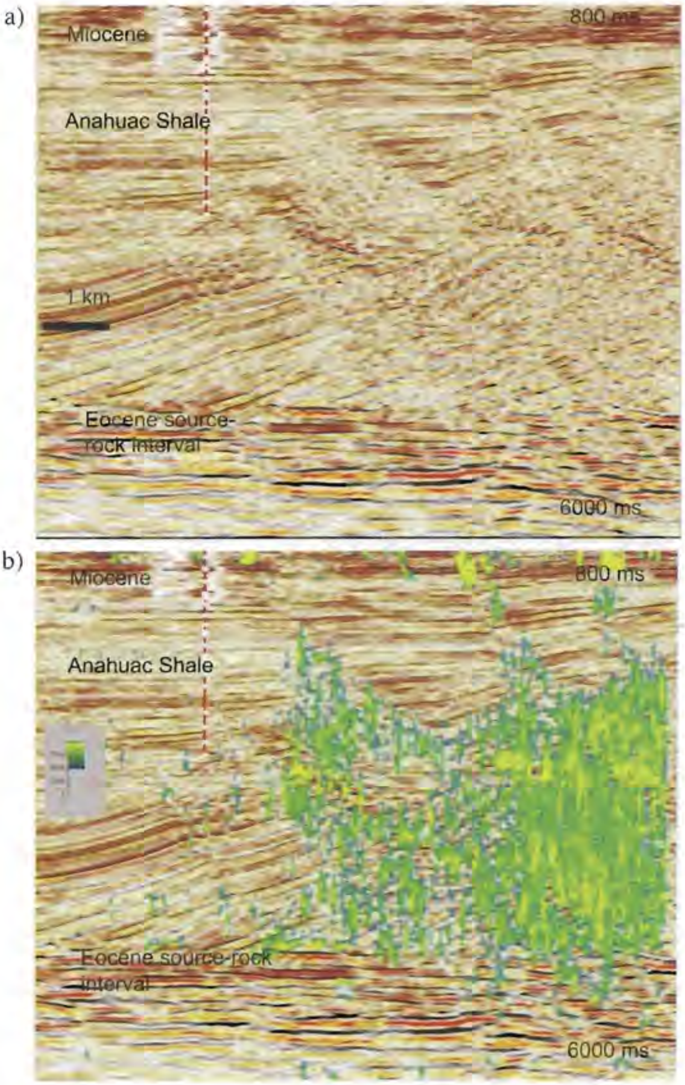

Figure 8. Seismic line in Texas state waters: (a) uninterpreted and (b) with chimney probability overlain. Chimneys originate from the gas-prone Eocene (approximate Wilcox) interval (seismic data owned or controlled by Seismic Data Exchange Ltd.).

Predicting vertical top-seal and vertical fault-seal risk

A major purpose of delineating gas chimneys is to predict vertical seal risk for prospects, prior to drilling. Heggland (2005) evaluates the character of gas chimneys above structural traps to predict seal integrity. His findings show that traps with chimneys on the flank of structures have effective seals. Traps with gas clouds over them generally have effective seals. However, traps with fault-related chimneys usually have breached seals. This model has been refined by Løseth et al. (2009); their new model distinguishes point-sourced chimneys (fault-related chimneys, blowout pipes, and mud volcanoes) from chimneys originating from a broad zone (gas cloud). These point-sourced chimneys can originate on the flank of a structure (providing a downdip limit to the accumulation), above a saddle in a structure (limiting the accumulation to that saddle), or at the crest of a structure. The latter case most likely indicates

a leaky trap. In all four cases (three point-sourced scenarios and one broad-zone scenario), the presence of chimneys in the overlying section can help determine the vertical-seal risk.

We have expanded this classification to include the character of gas chimneys above and below the oil or gas field or dry hole (Figures 9–11). The classification has also been expanded to include fault-related traps and top-seal traps, using nomenclature suggested by Cartwright et al. (2007). They divide fault bypass systems into two families, based on whether the faults define the trap (trap-defining fault) or whether they are embedded in the sealing sequence (supratrap faults). Supratrap faults are more often related to the hydrodynamic conditions within the reservoir and overlying sealing interval, whereas trap-defining faults are more related to regional tectonic stress conditions. Supratrap faults are often seen as polygonal faults with limited throw and areal extent for individual fault segments. These polygonal fault systems are generally good seals, based on the number of fields in the North Sea overlain by polygonal faulting (Cartwright et al., 2007). However, weakness in these polygonal fault systems can be exploited to provide vertical pathways for hydrocarbon migration (Berndt et al., 2003).

There is often some ambiguity in classifying a gas chimney because the features exist in a continuum and multiple mechanisms may act at the same time or sequentially. For example, it is often difficult to distinguish a mud volcano from a blowout pipe on the basis of seismic data alone. In the same way, a pipe may be initiated by fault movement. Additionally, there may be several pipes generally aligned along a fault zone. One way of addressing this issue is to limit the classification to point source (fault, blowout pipe, and mud volcano) or diffuse source (gas cloud) (Løseth et al., 2009). Additional case studies should determine if the distinctions between mud volcano, blowout pipe, and fault-related chimneys are statistically relevant in top-seal prediction.

Vertical fault-seal traps

Fault-related traps are divided into three classes (Figure 9), the first of which is a *fault-seal trap*. These structures show clear evidence of vertical charging from deep-seated faulting often related to basement structure, salt, or shale movement. The faults terminate at the reservoir interval or in the sealing interval above the reservoir. These traps are very low-risk, high-integrity traps (HITs) and often filled to spill. They are also a class 1 trap (Sales, 1997), gas prone in a multiphase system — very common in the GOM. Because the chimneys are located in the more poorly imaged section below the reservoirs, they are often unrecognized. The

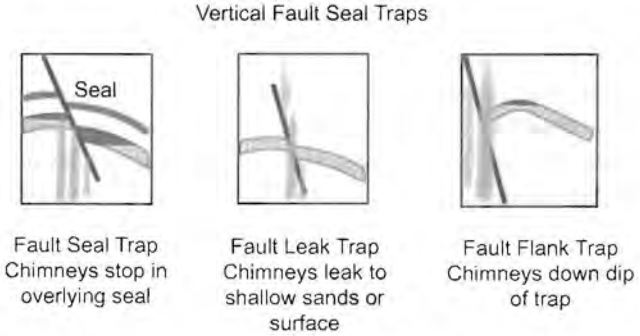

Figure 9. Classification of chimneys for predicting vertical fault seal, based on morphology of fault-related chimneys above the trap.

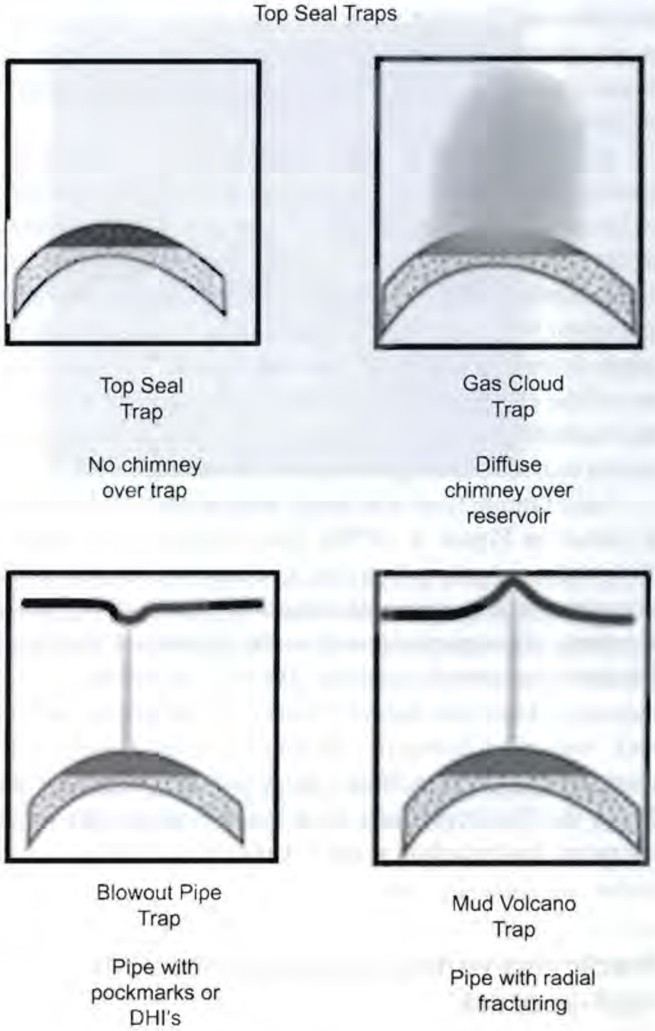

Figure 10. Classification of chimneys for predicting vertical top seal, based on morphology of nonfault-related chimneys above the trap.

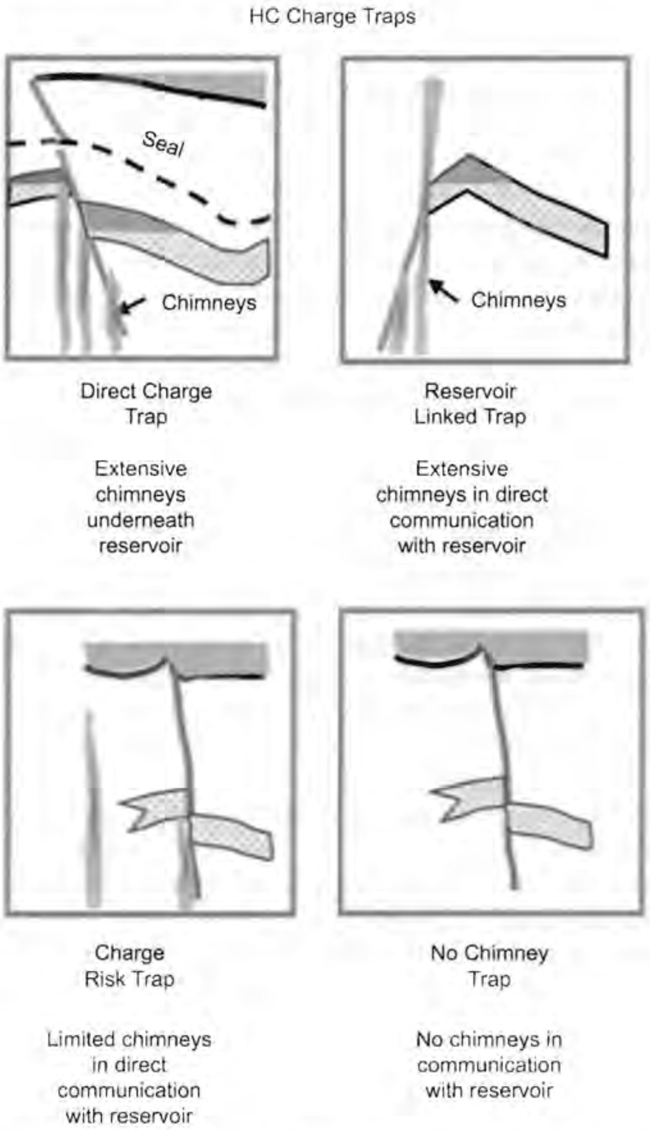

Figure 11. Classification of chimneys for predicting hydrocarbon charge, based on morphology of chimneys below the trap.

chimneys are often related to fracturing and provide a conduit for deep pressures to be transferred into the shallow section. Thus, the productive reservoirs generally occur near the top of abnormal pressure.

The second class is a *fault-leak trap*. These traps show fault-related leakage above the objective reservoir interval. These low-integrity traps (LITs) represent higher risk for vertical seal integrity. Fault-leak traps can be effective if the petroleum system is active today. Thus, as the fault leaks, it is continually being recharged.

The third class is *fault-flank traps*, with the fault involved in charging the trap but not in forming the actual trap. The fault in these traps is downdip of the crest of the structure; thus, the trap has a low risk of vertical fault or top-seal failure for the area of the structure above the fault intersection or the spill point. One possible risk with this type of trap is that the reservoir is of limited extent and is highly pressured. Thus, the reservoir would be bypassed in favor of shallower, low-pressure sands or expelled to the surface. These HITs are generally filled to spill or to their intersection with the charging fault. In multiphase systems, they tend to be gas prone because the oil phase will spill. This trap is equivalent to Sales' class 1 trap (Sales, 1997).

Fault traps can exist in a continuum between fault-leak traps and fault-seal traps. Faults can leak along a single point of weakness, often at an intersection of two faults, and resemble a blowout pipe. Studies have investigated the character of fault leakage over faulted traps in the Northwest Shelf of Australia (O'Brien and Woods, 1995; O'Brien et al., 1998; Cowley and O'Brien, 2000). Studies in the Bonaparte Basin (O'Brien et al., 1998) have determined that most oil fields are moderate-integrity traps (MITs) with fault leakage from 500 to 1500 m in length. LITs (breached traps) generally have faults with more than 1500 m of fault leakage. For fault-leak traps, the intensity of the leakage will also have an important control on trap integrity. Cowley and O'Brien (2000) offer a table that provides a likely interpretation of trap integrity based solely on HRDZ intensity (as measured by velocity pull-up) and areal extent.

Vertical top-seal traps

Top-seal traps may have faulting associated with the reservoir interval and may have some involvement in charging the trap. However, the faulting will not extend beyond the overlying sealing interval. These traps are divided into four classes, based on their morphology (Figure 10).

The first class of nonfaulted trap, a *top-seal trap*, shows no evidence of chimneys over the crest of the trap. These HITs have sufficient charge and are often filled to spill.

The second class of nonfaulted trap is a *gas-cloud trap*, with a broad, diffuse, low-amplitude zone above the trap. The mechanism of vertical migration is diffusion or fracturing below the resolution of the seismic data. Gas-cloud traps are generally MITs having hydrocarbon-column heights less than the extent of structural closure. These traps are often oil prone, leaking gas (and possibly oil) and trapping oil (Sales' class 3 trap). They can also be filled to spill, leaking gas and spilling oil (Sales' class 2 trap). We have observed gas clouds over gas-filled traps with hydrocarbon-column heights greater than 1000 m. In addition, gas clouds can occur over very low-gravity oil fields (Connolly et al., 2008). When occurring above an effective reservoir, gas-cloud traps are low-risk prospects. Gas clouds often occur in association with shale diapirism. Thus, in risking these prospects (especially with an absence of

A note on licensing regimes outwith the UK

Supplementary information for

Inadequate regulation of the geological aspects of shale exploitation in the UK

David Smythe

August 2020

It is not the intention of the main paper to discuss non-UK petroleum licensing regimes in general, nor even the offshore UK regime. However, the question of 'land banking' during or after the award of petroleum exploration licences in some regimes deserves discussion.

The offshore Australian sector, which is under the control of the Commonwealth, awarded licences (from 1992 onwards) by a work programme bidding process, but has since moved to a dual system of bidding for an exploration licence, either by a work programme or by a cash bid. Current Australian federal legislation states:

> "*There are 4 types of petroleum exploration permits:*
>
> *(a) a petroleum exploration permit granted on the basis of work program bidding (a **work-bid petroleum exploration permit**);*
>
> *(b) a petroleum exploration permit granted on the basis of cash bidding (a **cash-bid petroleum exploration permit**);*
>
> …" [https://www.legislation.gov.au/Details/C2020C00189]

On the question of 'land banking', Maloney (2005) states:

"The Petroleum (Submerged Lands) Amendment Act 1985 introduced a new form of tenement, the retention lease. ... In order to qualify for the grant of a retention lease, the holder of an exploration permit in which a discovery has been made must be able to satisfy the Joint Authority that the discovery is not now commercially viable, but that it is likely to become commercially viable within 15 years. ... 'Commercially viable' is not defined by the Act."

and

"An application for a retention lease must be accompanied by particulars of:
(1) the proposals of the applicant for work and expenditure in respect of the lease area during the initial term (being five years) of the lease;
(2) the commercial viability of the recovery of petroleum from the lease area; and
(3) the possible future commercial viability of the recovery of petroleum from the lease area."

Despite a recommendation to the Australian government (Hunter 2009) to consider a partial move towards discretionary awards, following Norway's example in frontier areas, the country has retained the bidding award system.

References

Hunter , T. 2009. *Review of the Australian upstream petroleum sector*. Submission to the Australian Productivity Commission. Bond University, Perth, Australia, 69 pp.

Maloney, D.A.W. 2005. Stranded Gas—Australia's Offshore Retention Leases. *Journal of Energy & Natural Resources Law* 22:3, 357-372. DOI: 10.1080/02646811.2004.11433375

Comments on letter from DECC to LCC dated 17 November 2014

Professor David Smythe
12 January 2015

Summary

- LCC asked DECC to comment on my two objections to Cuadrilla's drilling applications in Lancashire.
- DECC declined to comment on most of my critiques, saying it was the responsibility of the EA.
- The EA does not appear to have responded to my submissions, in which I point to the potential for contamination pathways to groundwater resources *via* natural geological faults and permeable geological layers.
- DECC asserts that the 3D seismic survey, which I contend is not up to the required standard, is "*adequate*", but has provided no evidence.
- A sample of the 3D survey published subsequently shows that both the quality of the data and Cuadrilla's interpretation of an important geological fault are questionable.
- The definition of what is meant by 'local' and 'regional' faults is left up to Cuadrilla.
- DECC asserts that it is safe to drill through so-called 'local' faults.
- There is no UK regulation or guidance on how or whether faults should be avoided, and by how much.

Introduction

Ms Clare Phillips of the Environment Directorate of Lancashire County Council (LCC) wrote to the Department of Energy and Climate Change (DECC) asking for comments on the objections to my submissions concerning Cuadrilla Bowland Limited's applications to drill at Preston New Road and Roseacre Wood. This is a comment on the response received from DECC, which is attached as Appendix 1.

Who is responsible?

The DECC letter restricts itself to comments on seismic hazard (the risk of triggering earthquakes). DECC absolves itself of responsibility for commenting on the wider question of whether drilling and/or fracking near to or through faults is an environmental risk. It states that this is the responsibility of the Environment Agency.

LCC asked DECC to comment on my two submissions. Did LCC likewise ask the EA to comment? I am unaware of any such request. All I have is an assurance from the EA that my comments, which were sent directly to the EA, would be taken into consideration.

Avoidance of faults

To return to the DECC letter; DECC agrees with me that all faults should be avoided, but, in DECC's view, only where fracking is to take place. DECC nowhere discusses how far away the nearest faults should be (the safety stand-off distance) from the fracking zone. I understand that there are as yet no UK regulations on this matter.

Cuadrilla's 3D survey in the Fylde

DECC considers that the 3D seismic survey is 'adequate' for the purpose of estimating seismic hazard. By this it means adequate to detect all faults which are "*likely to be*

significant". No criterion is supplied for this level of significance. DECC has based this view on a visit by an anonymous 'geoscientist' to the offices of Cuadrilla, where he or she was permitted to view the 3D data volume on a workstation. This, in my view, is a completely inadequate response to my question about the inadequacy of the 3D survey. The 3D data should be published, both in raw and interpreted versions, to enable truly independent scrutiny.

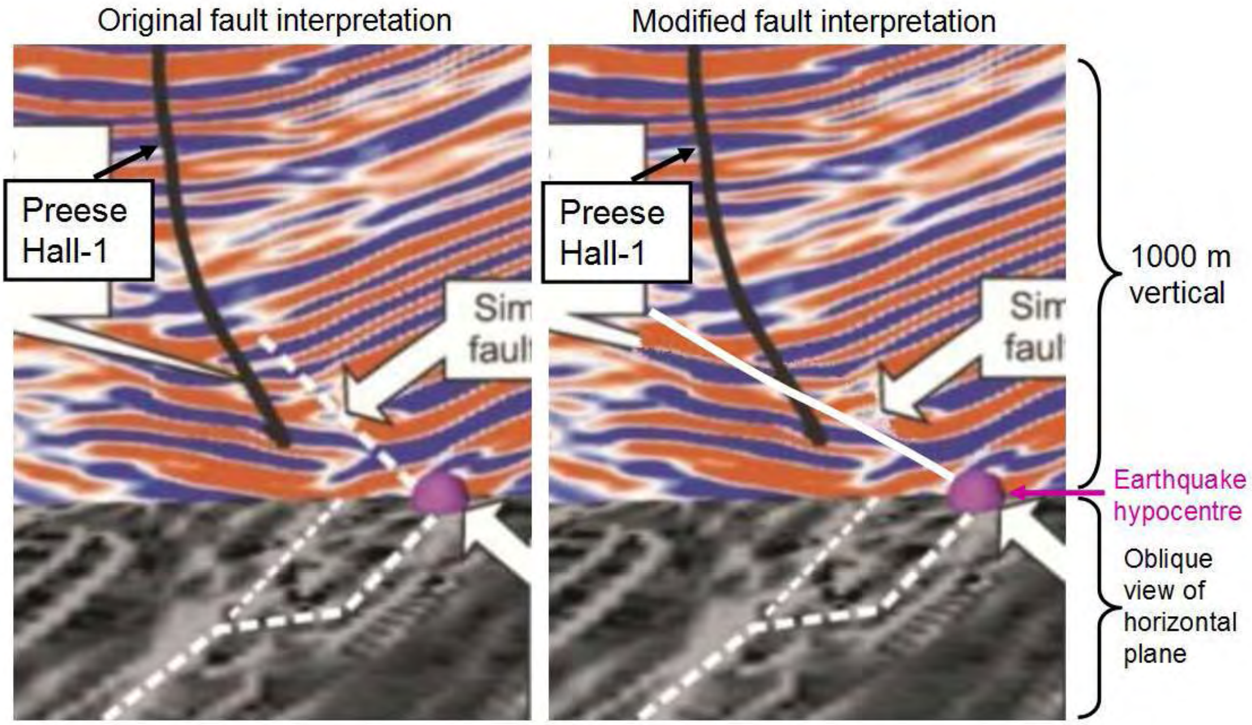

Figure 1. Comparison of fault interpretations on Cuadrilla 3D seismic survey.

Since DECC replied to LCC a small sample of the 3D dataset has been recently published in a scientific peer-reviewed paper co-authored by a Cuadrilla employee [1]. It demonstrates, in my opinion, that:

1. Cuadrilla's fault interpretation is open to question, and
2. The 3D dataset, even in the heart of the survey area, is of mediocre quality.

Figure 1 shows a detail of a diagram from the paper in question, on the left as published, and on the right as modified by me. The purpose of the comparison is to illustrate points 1 and 2 above. The detail is taken from an east-west vertical plane in the heart of the 3D survey around the Preese Hall-1 well. Faults are interpreted manually on such seismic data by recognising breaks in the continuity of the geological layers represented by the alternating sequences of red and blue. If the survey had been of a higher standard it should have been possible to see and map the faults directly as seismic reflector surfaces.

The 2 August 2011 earthquake has been located on a fault line, which is depicted by Cuadrilla as the dashed white line extending upwards and to the left from the purple ball representing the earthquake hypocentre (Fig. 1, left side). This event serves as a proxy for the main earthquake of 2 April 2011. All the shocks are believed to have occurred on the same fault. However, Cuadrilla's interpretation of the fault crosses the continuously layered seismic data some 200 m to the east of the projected location of the wellbore, which is depicted by the thick black line. The projection of the curved wellbore path is at most 50 m away from the vertical plane of the seismic section. On the right side of Figure 1 I have removed this interpretation and replaced it by a solid white line representing the fault on the vertical plane of the diagram. In my view this is a better interpretation because it follows

better the breaks in the seismic layering, as explained above. It also has the merit of passing obliquely *through* the well, just above the perforations of fracking stage no, 2, the operation which, it is agreed, triggered the earthquakes. In my interpretation the fault passes through the 50 m high zone where the well casing was deformed from a circular cross-section into an oval by the action of the fault slip caused by the main tremor. I shall be discussing the finer technical details of Cuadrilla's interpretation elsewhere, including a Comment on the original paper to be sent to the journal that published it.

In conclusion, regarding the 3D survey, it appears to be of generally mediocre quality, even in the centre where it would be expected to be at its best. We have only the word of someone asserting that it is adequate, particularly at the Roseacre Wood site, despite my pointing out that it cannot be of adequate quality there.

Definitions of faults

Cuadrilla has defined 'regional' faults, which it states it will avoid when fracking (but not when drilling) and 'local' faults, which it feels free to drill <u>and</u> frack without risk.

Who is making up the regulations here? Are we supposed to to accept that Cuadrilla will simply supply definitions which suit its purpose, i.e. to exploit the thick, faulted Bowland Shales? Its simplistic definitions are arbitrary, because faults do not fall into discrete categories such as 'local' or 'regional'. Not only do faults have a continuous spectrum of length, depth and displacement, but within sedimentary basins which have internal faulting (including the Bowland Basin) the faults even have a fractal distribution; that is, the pattern of the faulting (frequency of occurrence *vs*. size) looks self-similar at the small and the large scale. Therefore the division into two categories - local and regional - is arbitrary even if all the faults have been successfully and accurately identified.

The identification of faults depends on the methods used. Drilling, which might be thought intuitively to be the best method, can often fail to identify faults, as appears to be the case with the Preese Hall-1 fault depicted above. The orthodox 3D oil-industry seismic imaging method, if applied correctly and fully, can image faults having displacements of down to 5 m or so; this resolving power is about five to ten times smaller (better) than 2D seismic lines. The 3D method is also capable of directly imaging the fault surfaces, but this does not appear to be the case with Cuadrilla's 3D survey. As I have shown above, Cuadrilla's 3D survey, although a welcome improvement on the earlier data it relied upon, is not up to standard.

The safe distance for avoidance of faults - even assuming that they have been properly recognised and mapped in three dimenions in the subsurface - is not yet quantified in the UK by regulation nor by guidance.

Conclusion

DECC's letter does not adequately answer my objections. It remains to be seen whether the EA has responded in any substantive manner to my submissions.

Reference

[1] Clarke, H. et al. 2014. Felt seismicity associated with shale gas hydraulic fracturing: The first documented example in Europe. *Geophys. Res. Lett.* 10.1002/2014GL062047

Appendix 1

Received via email 17/11/2014

Department of Energy & Climate Change

Department of Energy & Climate Change
3 Whitehall Place,
London SW1A 2AW
T: +44 (0)300 068 6028

E: john.arnott@decc.gsi.gov.uk

www.gov.uk/decc

Ms Clare Phillips
Environment Directorate
Lancashire County Council
County Hall
Preston
PR1 0LD
(by e-mail)

Your ref: LCC/2014/0096/ASP/ASP
LCC/2014/0101/ASP/ASP

17 November 2014

Dear Clare,

Planning applications LCC/2014/0096 (Preston New Road) and LCC/2014/0101 (Roseacre Wood)

You invited comments on the objections submitted by Professor David Smythe to planning applications LCC/2014/0096 and LCC/2014/0101, and drew our attention in particular to his comments that regional faults are transmissive, and that Cuadrilla's definition of faults is defective.

The main focus of Professor Smythe's papers is on matters outside DECC's regulatory responsibilities, principally on the possibility of contamination of aquifers. We have no comments on those issues, which fall within the regulatory responsibilities of the Environment Agency. But some points in the papers do have some relevance to the regulation of seismic hazards, and we comment on those below.

First, it is said that all faults should be avoided, whatever their scale. So far as hydraulic fracturing is concerned, we would in general agree with this principle. However, from the viewpoint of seismic hazards, we do not think there is any need to be concerned about drilling through a fault, as opposed to hydraulically fracturing into or near a fault. Drilling, as such, is not in the experience of the oil industry an operation associated with seismic activity. We are not aware of any factor in the geology around the proposed drilling sites which should require avoidance of all faults, so far as the drilling phase of operations are concerned. (The paper cites a large German study in support of the proposition that all faults should be avoided. However, the relevant conclusions of the German study refer only to hydraulic fracturing and not to drilling operations.)

Second, it is said that the 3D seismic survey is inadequate in coverage, in particular because the proposed Roseacre drilling site is very near the edge of the survey area and the resolution of faults is consequently poor at that location. Since we consider that drilling through a fault does not entail any seismic hazard, the location of the

drilling site, or more precisely the trajectory of the initial vertical well, is not material to the adequacy of the 3D survey so far as seismic hazards is concerned. What matters is the resolution of faults available in the areas in which fracturing is proposed. A DECC geoscientist has reviewed Cuadrilla's 3D data on a workstation at their office, and we consider that the data quality is adequate in those areas to enable detection of all faults likely to be significant from the viewpoint of seismic hazard. We will scrutinise the Hydraulic Fracturing Plans and the plans for monitoring the growth of the fractures to ensure that the stimulated rock volume does not extend too close to any of the mapped faults.

Third, is said that faults should be assumed to be transmissive unless proved otherwise. This comment is not directly relevant to seismic hazards, but as noted above, the purpose of the HFPs and their scrutiny by DECC is to ensure that the full extent of the stimulated rock volume preserves a safe distance from any detectable fault. The fracturing fluids will therefore never enter the fault, and will not be transmitted along it.

Fourth, it is said that Cuadrilla's definition of faults is defective. However, the purpose of the definitions adopted is to distinguish between "local" faults, which Cuadrilla propose to drill through, and regional faults, which they do not intend to drill through. As noted earlier, we do not see drilling through faults as material to the assessment of seismic risk. As to the location and extent of fracturing operations, which are very material, Cuadrilla plans to avoid all detectable faults, which is the correct approach.

Finally, it is said that the current regulatory system is inadequate, in that no criteria have been specified in the "traffic light" system for shutting down operations, other than temporarily. We would not agree that this is a shortcoming. The association between hydraulic fracturing and seismic activity remains a relatively novel discovery and a developing area of knowledge. However, the data from the Preese Hall tremors indicate that careful monitoring of seismic activity in real time is likely to detect precursor events, providing scope to halt operations, reduce stresses, and avoid any more substantial tremor. That is the purpose of the traffic light system. But in the present state of knowledge, any predetermined protocol for action which should follow a red-light event would risk excessive precaution on the one hand, or avoidable disturbance to nearby residents on the other.

DECC's intention in any such instance is to explore the implications of the occurrence of the red-light event promptly but thoroughly, with a view to deciding whether operations can be resumed without undue risk of disturbance to local residents; and if so, what operations are acceptable and whether any further precautions are appropriate. We think this strikes an appropriate balance in present circumstances between precaution and protection. And we have no doubt that our powers are sufficient to curtail operations in any such case should it prove necessary.

We would be happy to address any further questions you may have on these matters.

Yours sincerely,

John Arnott
Oil and Gas Licensing Policy, Energy Development Unit

Case histories of relationship between BGS and government

Following the Rothschild report of 1971 on the management and funding of civil science, the BGS became a 'contractor' of commissioned research, with government departments like Energy as the 'customer'. See, for example, Parker (2016) for a history[1].

My own experience of pressure from management to alter or distort science to suit the 'customer's' perceived needs dates from 1984, when I was a Principal Scientific Officer at the BGS, and regarded as an expert on the crustal structure of the north-east Atlantic region. This subject was politically controversial for a period because of conflicting claims by four sovereign states over the Rockall region. I held a particular set of views - let us call it case A - and did not subscribe to an alternative viewpoint, case B. I was not permitted to publish my views, because my views were not considered 'helpful' to the UK claim on Rockall, either at Department of Energy or at the Foreign and Commonwealth Office (or both). I was then formally requested in writing (a very unusual step) to prepare a report advocating case B. I supplied the required report for management, in order to avoid being disciplined; from memory, my report comprised one page of text and one map. I do not know from which government department the request originated, but in my view this was a disgraceful attempt to manipulate the science for political ends. The episode was also counterproductive, because a more nuanced reading of my views (case A) would have shown that they were indeed 'helpful' to the UK territorial claim. But the chief geologist at the Department of Energy was incapable of apreciating such a nuance.

More recently, the behaviour of the BGS around geological nuclear waste disposal, another controversial topic, reveals hidden government influence. In March 2006 the BGS published a joint note with Nirex (at the time the government agency charged with disposal) stating that more than 30% of the UK landmass was potentially suitable for radwaste disposal. But the map was never published, despite requests under FOI. The crucial question was whether West Cumbria was included in the 30% deemed to be potentially suitable.

In May 2012 the radwaste team leader of the BGS, Dr Richard Shaw, claimed on BBC Radio Cumbria that West Cumbria had the "*potential to offer a good* [radwaste] *site*". This assertion flew in the face of all the scientific evidence, including a public inquiry, that the geology of West Cumbria is unsuitable. Dr Shaw was advocating a particular solution, without presenting any evidence, but no doubt supported by his superiors.

A closed meeting of the Geological Society of London and West Cumbria MRWS (Managing Radioactive Waste Safely) was held in June 2012. Two officers of the BGS attended this meeting in an official capacity (one of them being Dr Shaw), but were listed only as 'Fellows' (members) of the Geological Society, and not as BGS geologists. This deception had the support of the BGS management. In my view this covert attempt by the BGS to influence the discussion around nuclear waste disposal was misleading.

[1] Parker, M. 2016. The Rothschild report (1971) and the purpose of government-funded R&D—a personal account. ***Palgrave Communications*** **volume** 2, Article number: 16053

Cuadrilla at Balcombe in the Weald

In its planning application of January 2010 for Balcombe (called by Cuadrilla the Lower Stumble site) Cuadrilla describes fracking[1]:

There may be a need to stimulate a stage which flows gas/oil but at a low rate to ascertain if the gas was being held back by poor porosity or permeability or lack of natural fracture or a combination of all three. Stimulation is carried out by pumping water under pressure into the natural fractures in the shale formations to open them up to allow the gas to flow more freely. ln some cases silicone sand is then pumped in to hold open the fractures once the water is removed.

The application[2] was ambiguous about whether such fracking would be carried out on the vertical well or on an optional horizontal well, stating:

The target formation for the Lower Stumble well is the Middle to Upper Jurassic including the Corallian Sandstone, Kimmeridge and Portland Sandstone. The Cuadrilla exploration plan is to drill vertically through the Middle to Upper Jurassic sequence, recover core from some of the intervals and drill to a total depth of 4700 feet (below surface) in the Great Oolite. If the result of the core analysis and geological investigations appear promising we may choose to drill a horizontal well section to further test the presence of hydrocarbons.

The fault previously postulated[3] at the heel of Balcombe-2z, before the well data were released, to account for the repetition of the top of the I-micrite does not exist. The released well data show that there is no step or sidetrack in the wellbore. The repetition occurs just below the casing change from 7 inch to 4.5 inch. The 7 inch casing was set at 2694 feet, then the cement plug was drilled through and drilling continued with a 6 inch bit. The apparent repetition of the sequence, as recorded by cuttings, is probably due to the lag time of the cuttings reaching the surface (perhaps 1-1.5 hours) not having been properly accounted for.

I have compared the Balcombe-1 released well log, as a proxy for Balcombe-2, with other well logs in the basin, as reproduced by the BGS (Andrews, 2013). Balcombe-1 is only 10 m east of Balcombe-2. The aim is to see whether the Paddockhurst Park Fault can be recognised cutting the Kimmeridge Clay in the gamma ray and sonic logs of Balcombe-1. The BGS study does not include the two wells about 7 km north of Balcombe, Worth-1 and Turners Hill-1. It does include Bolney-1, km SSW of Balcombe; the Bolney-1 and Balcombe-1 logs are very closely correlatable in the clay from the top of the I-micrite to about 80 m higher up, but then become poor. The Bolney-1 section is likely to be cut by faults intersecting the uppermost Kimmeridge Clay below the Portland and Purbeck Beds.

The log correlation between Southwater-1 (SW in main paper, Fig. 3c) and Balcombe-1 is remarkable (Figure S5). Southwater-1 lies 14.7 km WSW of Balcombe, approximately along strike of the basin axis and sub-parallel to the regional E-W faulting. Figure S5 shows the pairs of logs, gamma ray and sonic, on the left and right, respectively, of the lithological log for Balcombe-1. The Southwater logs are in red, and Balcombe logs in black. The log scales and ranges are 0-150 API units for the gamma ray and 140-40 µs/ft for the sonic.

[1] Cuadrilla Resources Limited, 2010. Lower Stumble Hydrocarbon Exploration Site Planning Application [January 2010] Appendix C.

[2] Cuadrilla Resources Limited, 2010. Lower Stumble Hydrocarbon Exploration Site Planning Application [January 2010] Supporting statement to the application.

[3] Smythe, D. K. 2016, section 4.1.2 and figure 8b.

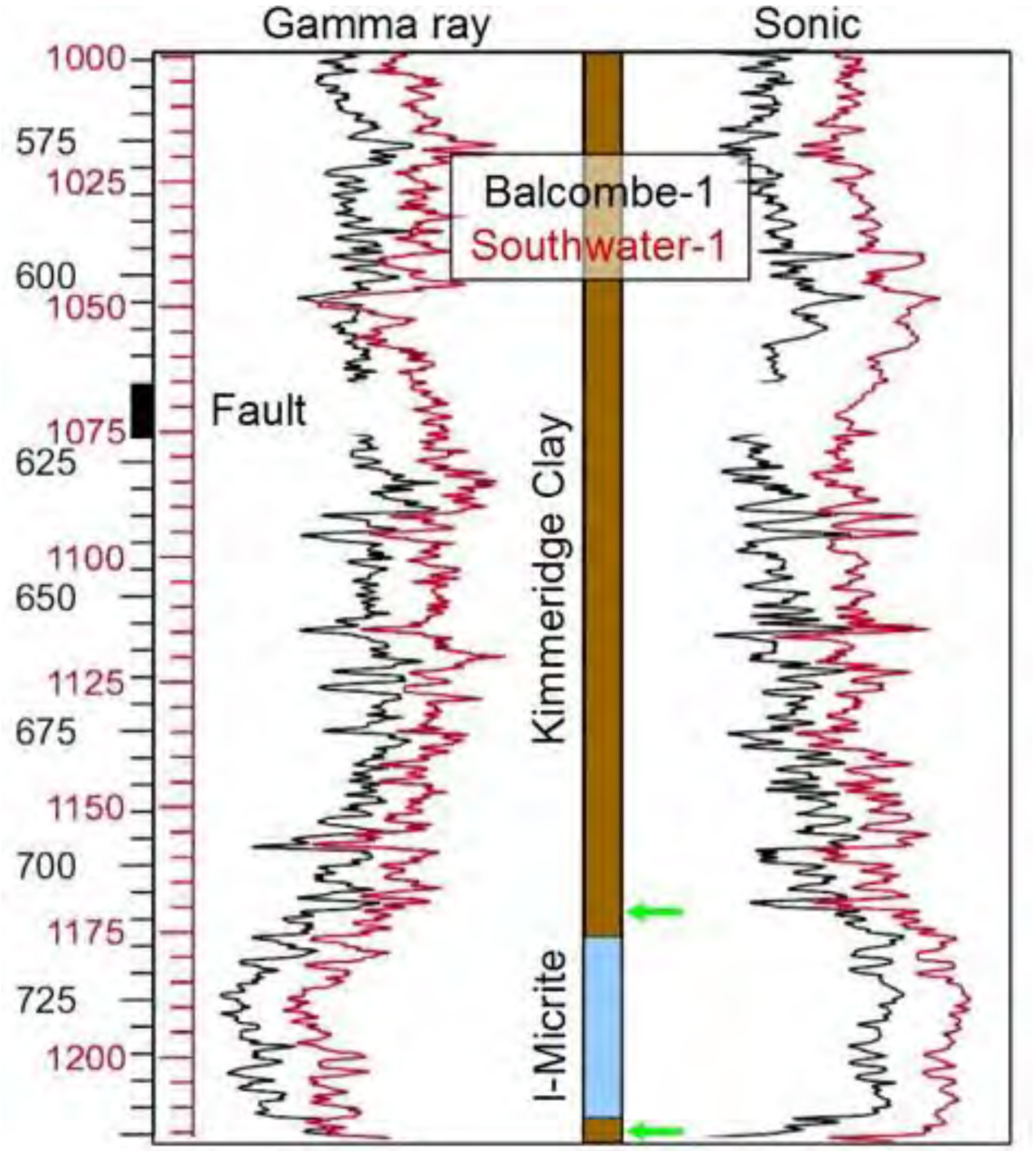

Figure S5.

A near-match, down to the decimetre scale, has been obtained by (a) scaling the Southwater logs to 93% of the Balcombe scale, and (b) inserting a 10 m gap in the Balcombe logs at 620 m driller's depth. This gap, demonstrating missing Balcombe section relative to Southwater, is strong evidence that the normal Paddockhurst Park Fault cuts the vertical well at about 620 m driller's depth (615 m below ground level). The fault geometry corresponds closely to my prediction[4] before Balcombe-2 was drilled in summer 2013, but has a smaller throw than predicted. Given that the 10 m throw is somewhat larger than the estimated 6-9 m throw at the surface, it also implies that the fault penetrates at least as deep again – to 1200 m depth – before possibly dying out downwards. Cuadrilla has not to date acknowledged the existence of this fault.

[4] www.davidsmythe.org/fracking/cuadrilla%20sussex%20critique%20V2.0.pdf

Canonbie

Extract from the Scottish government's expert scientific panel report[1] on unconventional oil and gas:

3.15. Greenpark/Dart Energy, Canonbie PEDL 159 – Historical analysis

- The coalfield within the PEDL 159 area was known to have high gas contents from ten boreholes drilled between 1955 and 1983 by the National Coal Board (later British Coal).
- Records for these boreholes are held by BGS;
- A large subsurface area, between the Scottish and Cumbrian outcrop areas of the coalfield has never been mined;
- Greenpark-Marathon conducted exploration at the site, drilling 8 wells before April 2012. 18 additional borehole sites are already permitted;
- Two of the former Greenpark licences issued by SEPA allowed for "the injection of fracking fluids into groundwater" in coal seams at Mouldyhills and Broadmeadows, using laterals drilled into 5 individual seams. The plan was to use nitrogen foam frack (70% by volume nitrogen gas) rather than water, at an operating pressure of 17 MPa at depths between 560 and 1020 metres below ground, with microseismic monitoring to check no fracture propagation reached overlying aquifers. These fracking operations were never implemented;
- Dart Energy Ltd acquired the licences in 2012 and are proposing development without fracking, along the same lines as their Airth operations.

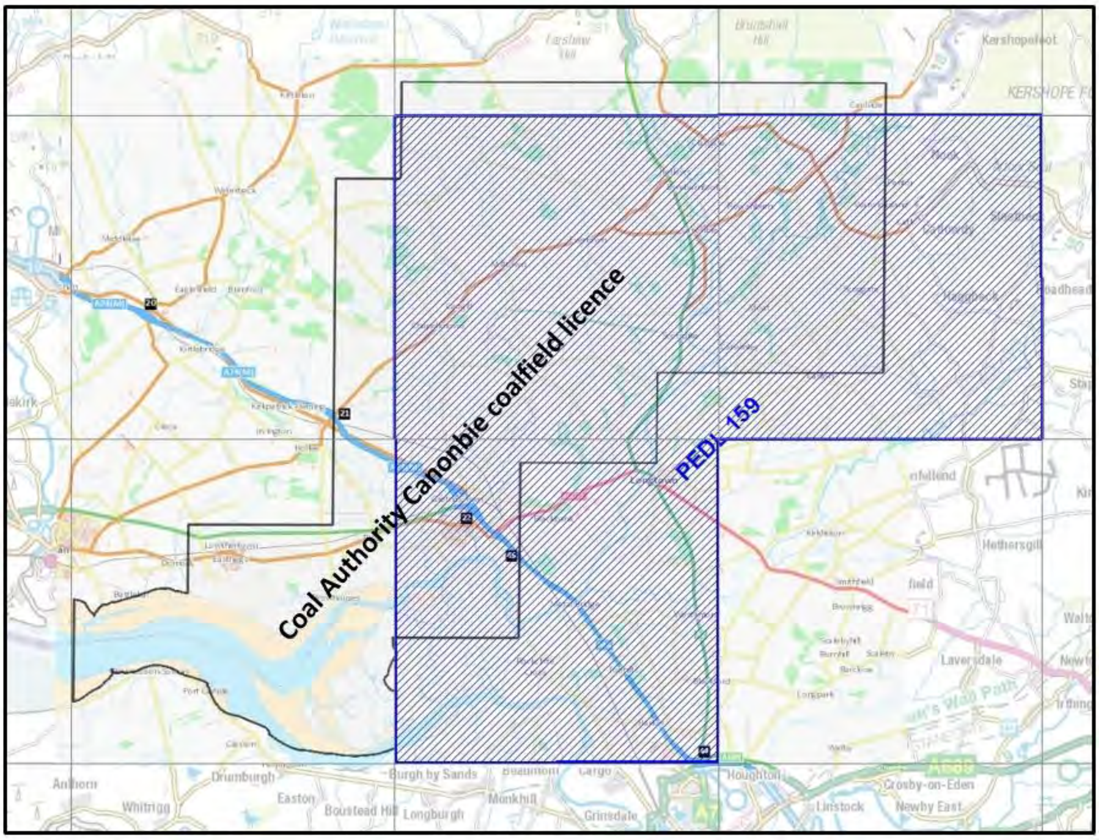

Figure S6. Overlap of the Coal Authority licence with the contemporaneous PEDL 159 CBM licence at Canonbie.

[1] The Scottish Government 2014. Independent expert scientific panel – report on unconventional oil and gas. https://www.gov.scot/binaries/content/documents/govscot/publications/advice-and-guidance/2014/07/expert-scientific-panel-report-unconventional-oil-gas/documents/00456579-pdf/00456579-pdf/govscot%3Adocument/00456579.pdf

Airth 2014

The following comprises edited extracts from the enquiry precognition and rebuttal by Professor Smythe, to give an example of the misleading geological interpretation and/or geological errors by Dart. Figure numbers have been renumbered herein.

[Extract from the precognition]

Dart has a licence from DECC to explore within the area of PEDL133. I shall only be concerned with the sub-area within this licence block (hereinafter referred to as the proposed development area, or PDA), where Dart proposes to drill a number of wells. Underground connecting tunnels or bores will be constructed, and lateral, or horizontal wells will be bored out from the vertical wells. All these operations will take place at a depth of around 800 - 900 m below mean sea level. According to Dart, no stimulation, such as hydraulic fracturation ('fracking') or acid treatment, is necessary. Because no fracking will be used, there will be no problem of earthquake triggering, according to Dart.

The original planning submission and later documents singularly fail to disclose adequately the current state of drilling, background geological knowledge to date, and details of drilling methodology to be employed. I would expect to find (*inter alia*) within the submissions:

- Composite well logs for every existing well,
- A brief history of why each existing well was drilled,
- Fence diagrams linking the wells,
- Seismic data used (if any), both raw and interpreted,
- Contour maps of the PDA showing structure of certain key horizons,
- Details as to how the horizontal wells will be constructed.

For example, in the original application the logs for 8 shallow water boreholes are reproduced, but the only log supplied for a deep borehole is Airth-6. There are stock extracts from industry manuals and brochures for the drilling rigs, generators, and so on, but what is missing is an account of how the horizontal wells will be drilled so as to confine the borehole path within each coal seam. Such information is crucial for making a safety case within an environmental statement, particularly as such technology is new to the UK.

Dart presented a set of maps showing old coal-mine workings over the entire area of PEDL133, and two geological cross-sections, dated May 2013, in its original G20 submission to the councils. The location of the two cross-sections is shown in Figure 1. The east-west cross section is 4 km north of Letham Moss (which can be considered as the centroid of the proposed operations) and 1500 m north of the most northerly lateral. It nearly intersects the Inch of Ferryton-1 oil well. The north-south cross-section runs some 200 m west of Airth-4, Airth-11 and one of the proposed vertical wells, but only intersects four linear features (lateral wells or underground tunnels) of the proposed development.

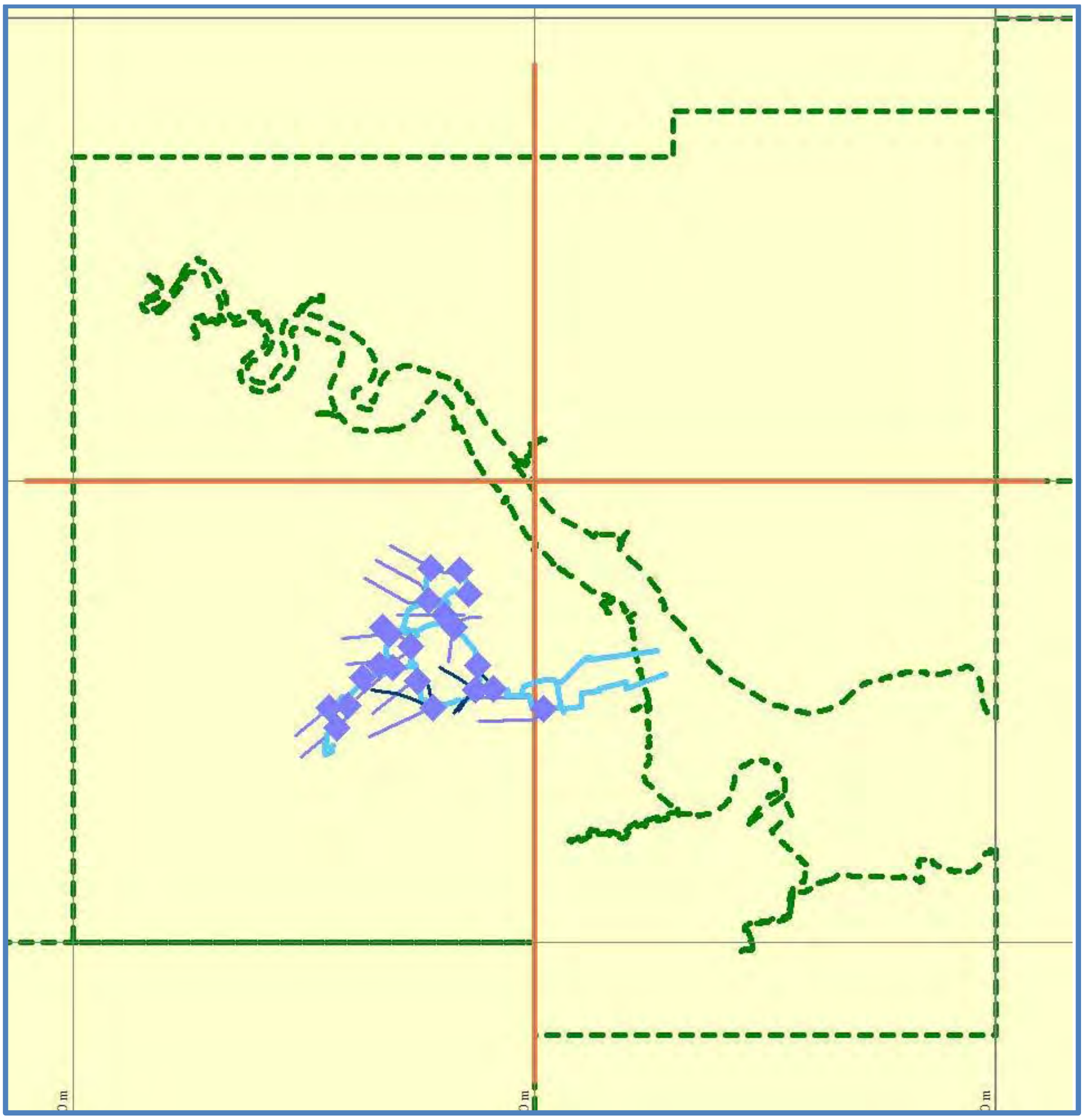

Fig. 1. PEDL133 (green dashed area) and location of two geological cross-sections shown in orange; AA' (E-W) and BB' (N-S). Blue shapes show the PDA.

The two cross-sections supplied by Dart are of very limited use, due to their inappropriate location relative to the PDA.

The throws (vertical displacements) of some faults are indicated on the old edition of the Airdrie solid geology sheet, measured in fathoms. I have multiplied the figures by two to get an approximate throw in metres. These figures presumably refer to the maximum throw in the central portion of the fault, and if the fault extends horizontally for several kilometres, the throw figure can be presumed to be valid vertically down to at least a kilometre, i.e. at least as deep as the target zone.

Dart's section BB' is misleading, as it excludes several of these normal faults, trending east-west, within the PDA (Fig. 2). Two of these have throws of 34 m and 50 m, respectively, at the location of the cross-section. The latter fault lies at the southern margin of the PDA at the section location, and runs westwards right through the PDA. Another E-W fault about 1 km south of the PDA has a throw of 40 m.

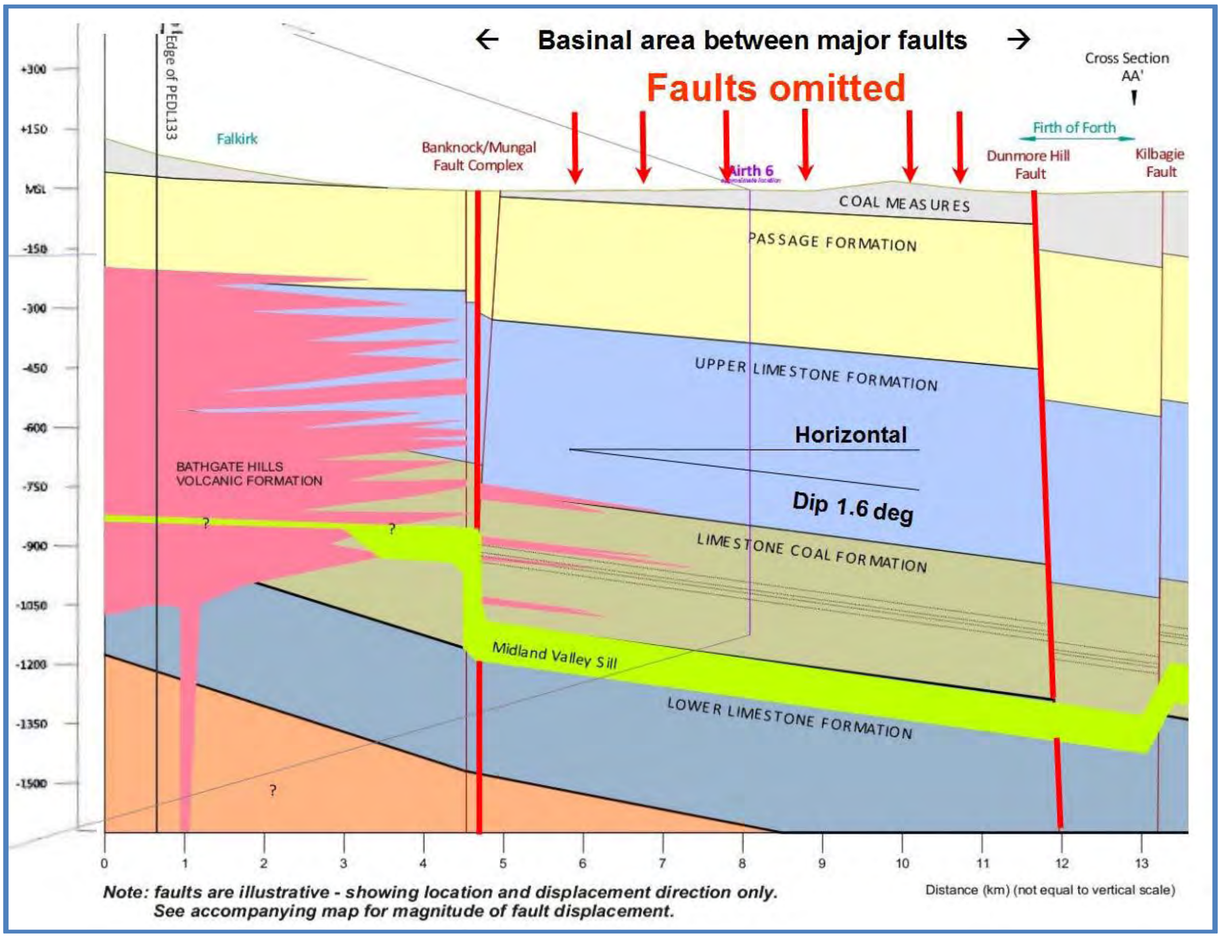

Fig. 2. Southern half of cross-section BB', as revised by Dart, October 2013, south on left. The locations of six faults (vertical red arrows) have been superimposed from the BGS maps. These should have been shown on the cross-section.

I have schematically re-interpreted the central part of Dart's cross-section BB' to show what the geology should look like. This is shown in Figure 3. The base of the Coal Measures is shown by the black lines between the various faults; this replaces the over-simplified constant-slope line depicted by Dart (the upper blue dashed line in Figure 3). The same principle will apply to all the geological layers below, including down to the target coals at 900 m or so below sea level, but I have not attempted to show all these.

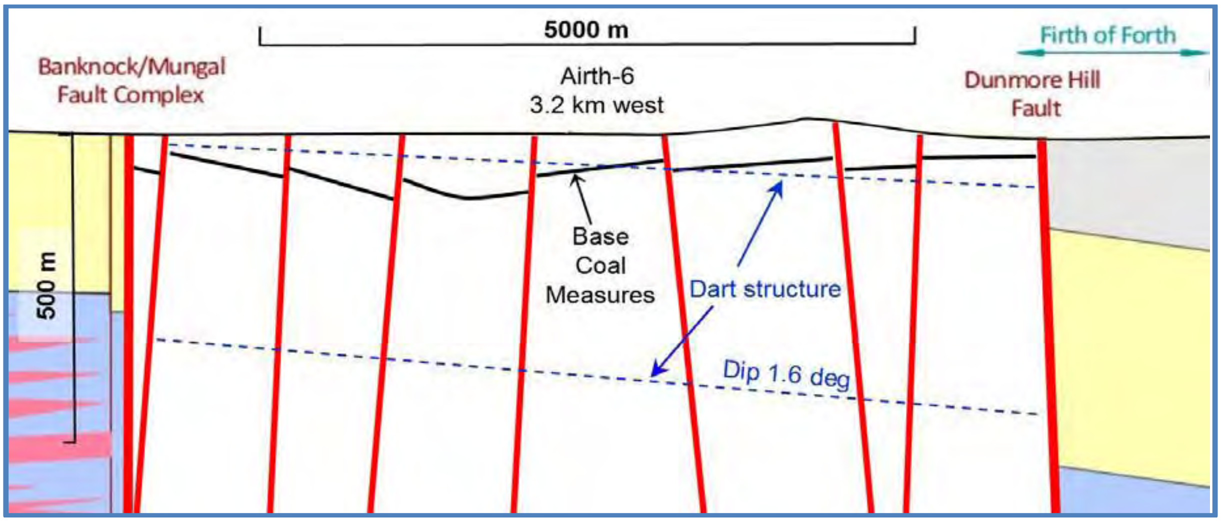

Fig. 3. Schematic re-interpretation of the central part of Dart cross-section BB'. The oversimplified Dart structure of a constant northward dip if about 1.6° has been replaced by a more accurate depiction of the structure of the base of the Coal Measures.

In addition, Dart has failed to depict the large quartz-dolerite dyke running E-W through the area about 2 km south of the PDA. This should also have been shown in its section BB', where it runs up and along a fault. It is coeval with the Midland Valley Sill, which is shown in the cross-section in light green. It is not apparent why Dart chose to omit this major feature from the cross-section.

[Extract from the rebuttal, responding to precognition by Dart geologist Mr David Goold]

Mr Goold also takes me to task for criticising both the location and the accuracy of the two cross-sections supplied by Dart. It concerns, in particular, the Dart N-S cross-section (Fig. 2). He makes no attempt to respond to the inaccuracy of the cross-section , but merely asserts that its location is "fit for purpose". As I pointed out in my precognition, Dart acknowledged the errors in the cross-section, but, instead of correcting them, Dart simply removed the vertical scale and added a disclaimer. Mr Goold defends this irresponsible approach in his precognition. Far from being 'fit for purpose', I contend that to present the only N-S cross-section through the PDA made available by Dart in a form in which important faults are knowingly omitted is misleading.

Mr Goold argues that my view of faults persisting to depth is over-simplified, citing two reasons:

- Rapid lateral and vertical variation in fault throw due to depositional conditions (i.e. growth faults),
- Fault geometry, where two separate faults at the surface meet at depth in a fork-like structure.

Neither of these two complicating factors in fault geometry is an excuse for omitting faults, as Dart has done. I concur in principle with the first reason cited above, because the BGS memoir to accompany Falkirk sheet 31E says so.

The second reason cited above by Mr Goold has been fully accounted for by me where appropriate, as seen by my seismic interpretations. Turning back to my provisional re-interpretation of Dart's N-S cross-section (Figure 3), it can be seen that the three missing faults to the south of Airth-6, which I have re-inserted, all have *southerly* downthrows. The most northerly of these three faults, just left of the 'Base Coal Measures' label is the Carbrook - Kinnaird House Fault discussed above. Due to their southerly downthrow it is unlikely, according to the BGS view, that these were growth faults. The first fault to the north (just to the right of the label) is the Letham Fault. This fault, and the next one to the north, may well show evidence of growth faulting.

None of the above affects in any way my conclusion that Dart's N-S cross-section is misleading, and that my Figure 3 is a much more realistic interpretation of both the folding and faulting.

Therefore Mr Goold's assertion that:

"Professor Smythe's assertion that the throw on faults indicated on Geological Survey maps is valid to depths of at least 1km is a gross oversimplification which does not take into account fault geometry and sedimentology."

is invalid, not only qualitatively as I have explained above, but also quantitatively.

In conclusion, I have not misinterpreted the faulting as asserted by Mr Goold, due to the *"paucity of data"* that I have collected. On the contrary, I have more than enough data to be able to analyse Dart's work. I stand by my view that Dart has a limited understanding of the geological structure of the PDA; in particular, there is insufficient understanding for Dart to be permitted at present to proceed with the development.

Micrites in the Kimmeridge Clay Formation

Conoco drilled Balcombe-1 in 1986. Part of the log for the Jurassic is shown in Figure 1.

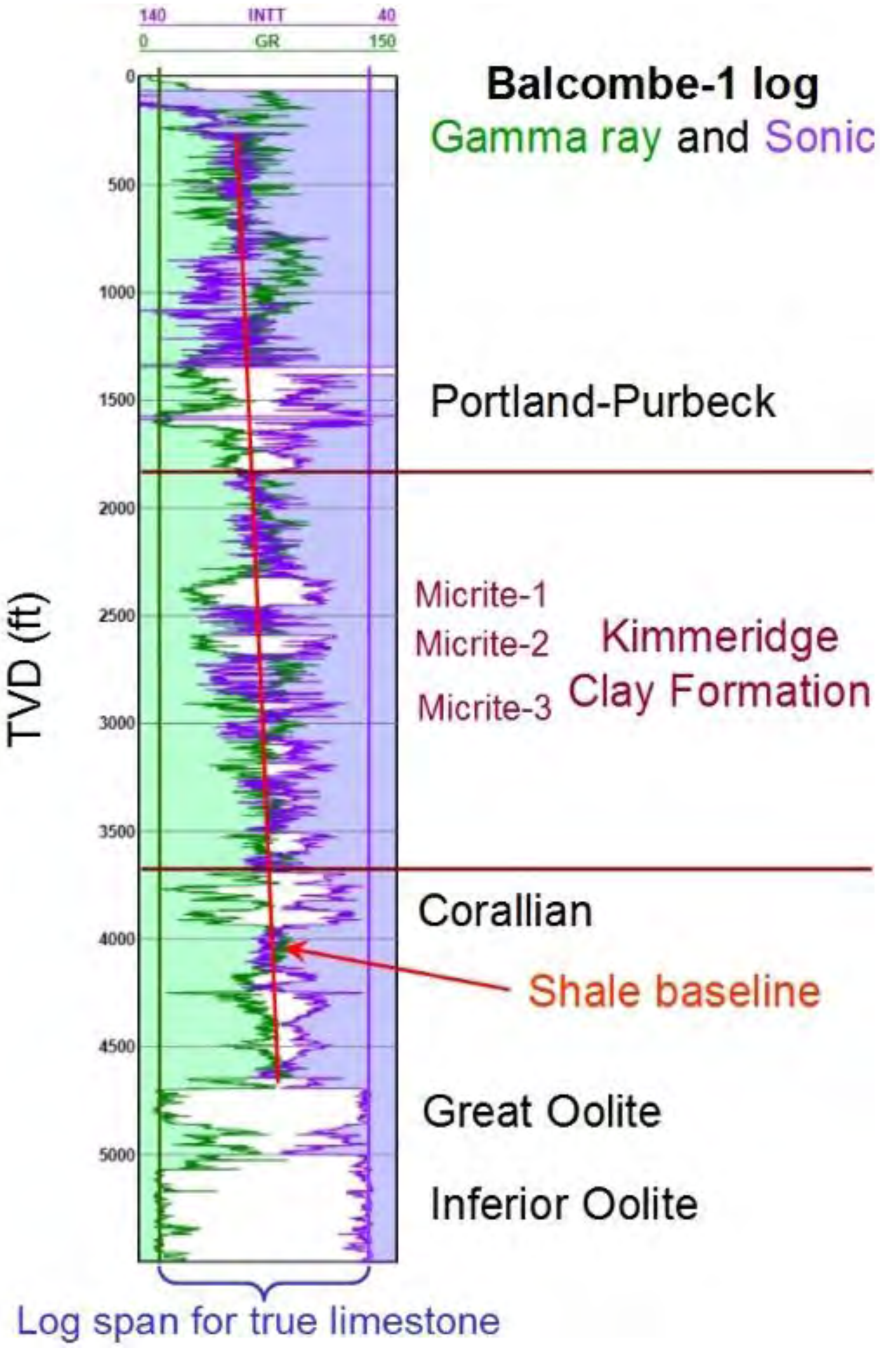

Figure 1.

The two log attributes are gamma ray (green) and sonic (purple). I have arranged the scales so that the logs coincide for shale, along the shale baseline marked in red. A shift leftwards of the green curve to low gamma ray readings, coinciding with high sonic velocity (shift of the blue curve to the right) opens up the gaps shown in white, aligned symmetrically on either side of the shale baseline. The gap for a true, pure limestone is illustrated by the Great and Inferior Oolites. I have put in two parallel vertical lines to highlight the span, or divergence, of the two logs when 100% limestone is present, such as is the case with the Oolites. The two main Kimmeridgian micrites, 1 and 2, recognised by Conoco at Balcombe-1, are clearly far from being pure limestone (calcium carbonate). These correspond to the BGS 'I' and 'J' micrites, respectively. They are calcareous mudstones or 'dirty' (impure) limestones, known informally

in the hydrocarbon industry as micrites. Conoco did not recognise any deeper micrites, noting merely the presence of occasional groups of argillaceous limestone beds a few feet thick at most, below the second micrite. The BGS recognises a third micrite (‘K’, labelled Micrite-3 in the log above) of about 50 ft (15 m) in thickness.

[extract from a consultation submission, 2019[1]]

I have carried out my own correlation of the Kimmeridge Clay Formation from the exposures in cliffs in Dorset into the Weald. This exercise had already been carried out two decades ago as part of an academic study, but was somewhat flawed in that the fence diagram (a well-to-well log correlation with no seismic control) proceeded northwards over the Purbeck-Wight Fault zone. The stratigraphy there was explained by a severe thinning of the formations over a supposed palaeo-high in Dorset. My revised interpretation comes to the same conclusions as this earlier study about the detailed link from Dorset to the Weald, but is more robust in that my correlation ties each well to the next *via* seismic data. Furthermore, my fence diagram starts from the Dorset coast, heading eastwards across Bournemouth Bay and the Isle of Wight, and only finally heading north-eastwards into the Weald using mainland wells east of the Isle of Wight. This avoids the Purbeck-Isle of Wight Fault Zone.

This study confirms that the exposures of the lower main micrite (KL2 in UKOG parlance) as seen at Horse Hill are the very same rock as seen in the cliffs (Fig. 2). The cliffs show calcarous mudstones, as photographed and labelled by Dr Ian West of Southampton University. The thin white shelves are sandstones, not limestones. Therefore by no stretch of the imagination can this 30 m thick cliff section be termed a 'limestone' as UKOG (and other Weald licensees) call it.

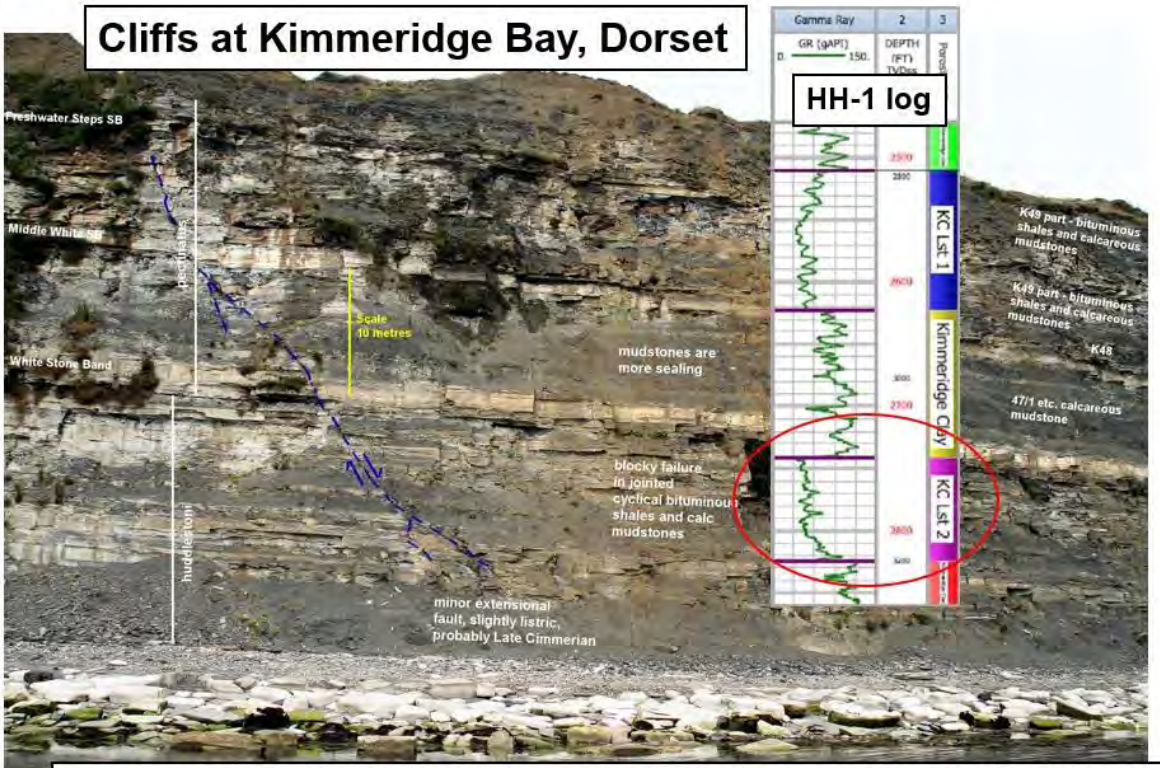

Fig. 4.3

Exposure of lower micrite, 30 m thick, corresponding to UKOG Kimmeridge Limestone 2 in Horse Hill-1 log (circled, scale in feet). In detail, the exposure comprises calcareous mudstones with thin sandstone bands. My log correlation from the borehole at Southard Quarry, just inland from this picture, along 2D and 3D seismic via the Isle of Wight to the Weald confirms that the micrites at depth in the Weald are essentially the same as shown here. Photo © Ian West 2011.

Figure 2.

[1] Smythe, D. K. 2019. *Geological objections to Surrey County Council planning application no. 2018/0152 by UK Oil & Gas for hydrocarbon developments at Horse Hill*. 21 June 2019, 19 pp.

Europa at Leith Hill

Below, timeline from 'The battle for Leith Hill':
https://www.keithtaylormep.org.uk/publication/battle-leith-hill-how-community-came-together-protect-beautiful-surrey-hills-oil-and

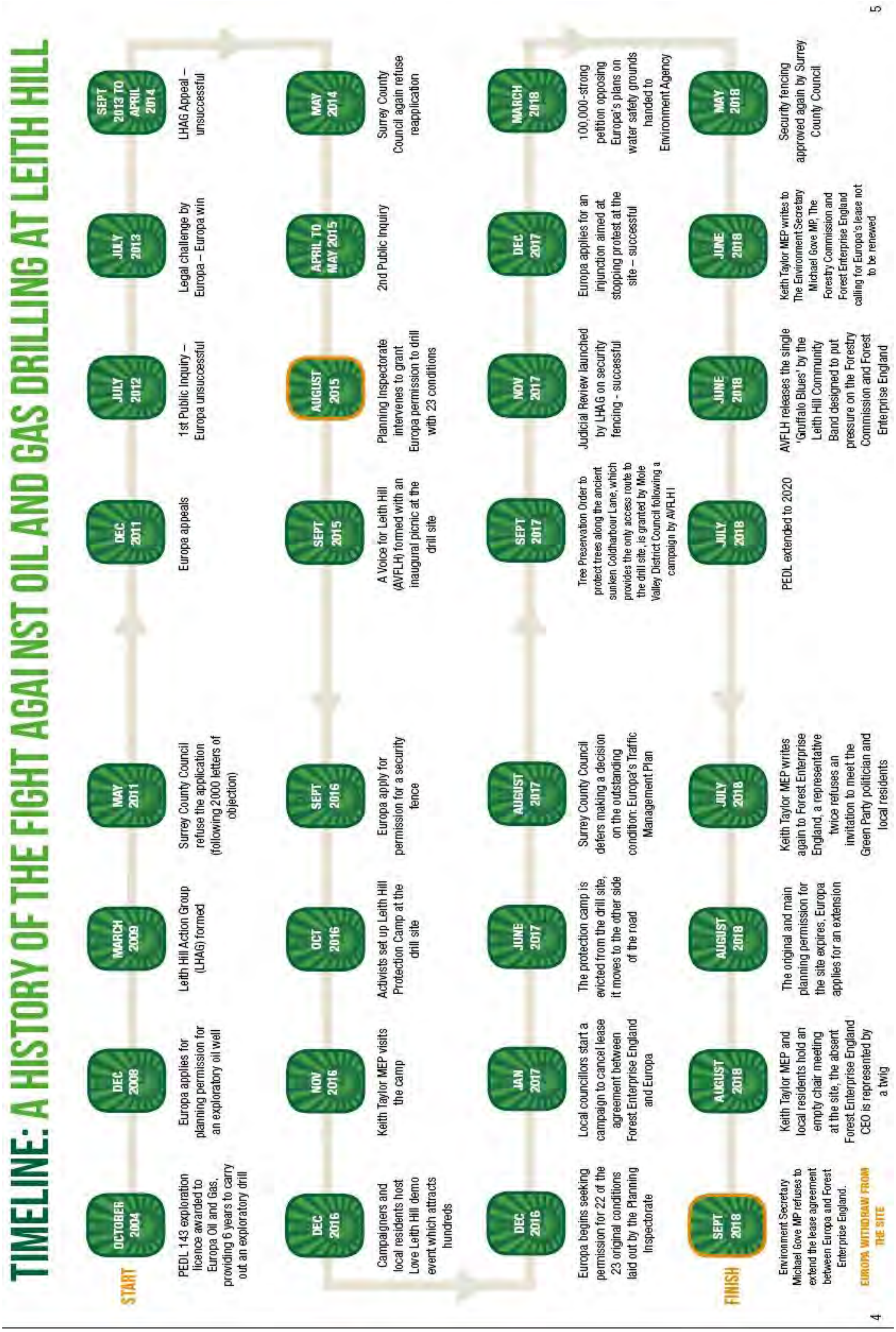

Extracts from consultation submission

[Original figure numbering has been retained]

Faulting

Faulting in the neighbourhood of the wellsite

Figure 4.1 shows the seismic database around the target zone of the Applicant's proposed well. It also shows the Applicant's version of the district faulting (cf. Figure 2.4 above) in more detail.

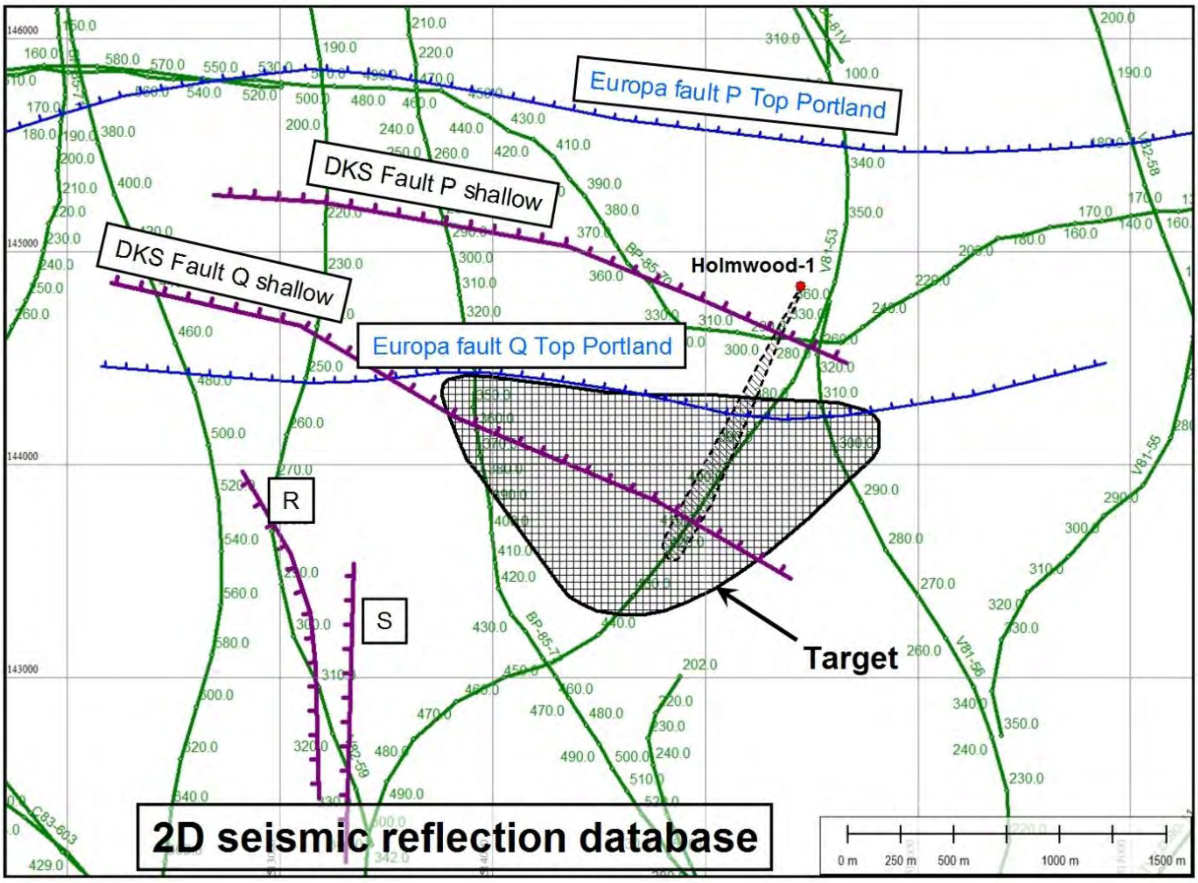

Figure 4.1. Seismic data (green lines) around the Applicant's target area and wellsite (red dot). The wellbore trajectory is shown by the dashed-line hatched area extending SSW from the wellsite. The Applicant's interpretation of faults at Top Portland level is shown by blue lines; my version of faults (at a shallower depth) is shown by purple lines.

My version of the two main faults P and Q (see Figure 2.5 above) is shown in Figure 4.1 by purple lines. My fault P at shallow depth probably corresponds to Europa's fault P at Top Portland level; it is mapped further south than the latter because of the northerly dip of the fault plane. In contrast, my version of fault Q runs at an angle of about 30° to the east-west trend of Europa's fault Q. Recall also that BP's version of the faults at Q (Figure 2.1) trend towards the ENE, i.e. different again.

The problem with correlating the faults from one seismic line to another is basically that we do not have enough data. The E-W spacing the the seismic lines running N-S is between 1 and 2 km, which is insufficient for identifying structures accurately at the sub-one-kilometre scale. In addition, there is only one seismic line (BP-85-70) running E-W, and even that line takes a very sinuous path.

Faulting along the well trajectory

Figure 4.2 shows seismic line V81-53, on which the wellbore trajectory design is based, in a horizontally compressed form, and with various faults identified by the termination and/or offsets of seismic reflectors. The green line is the topography, converted to a pseudo-reflection time.

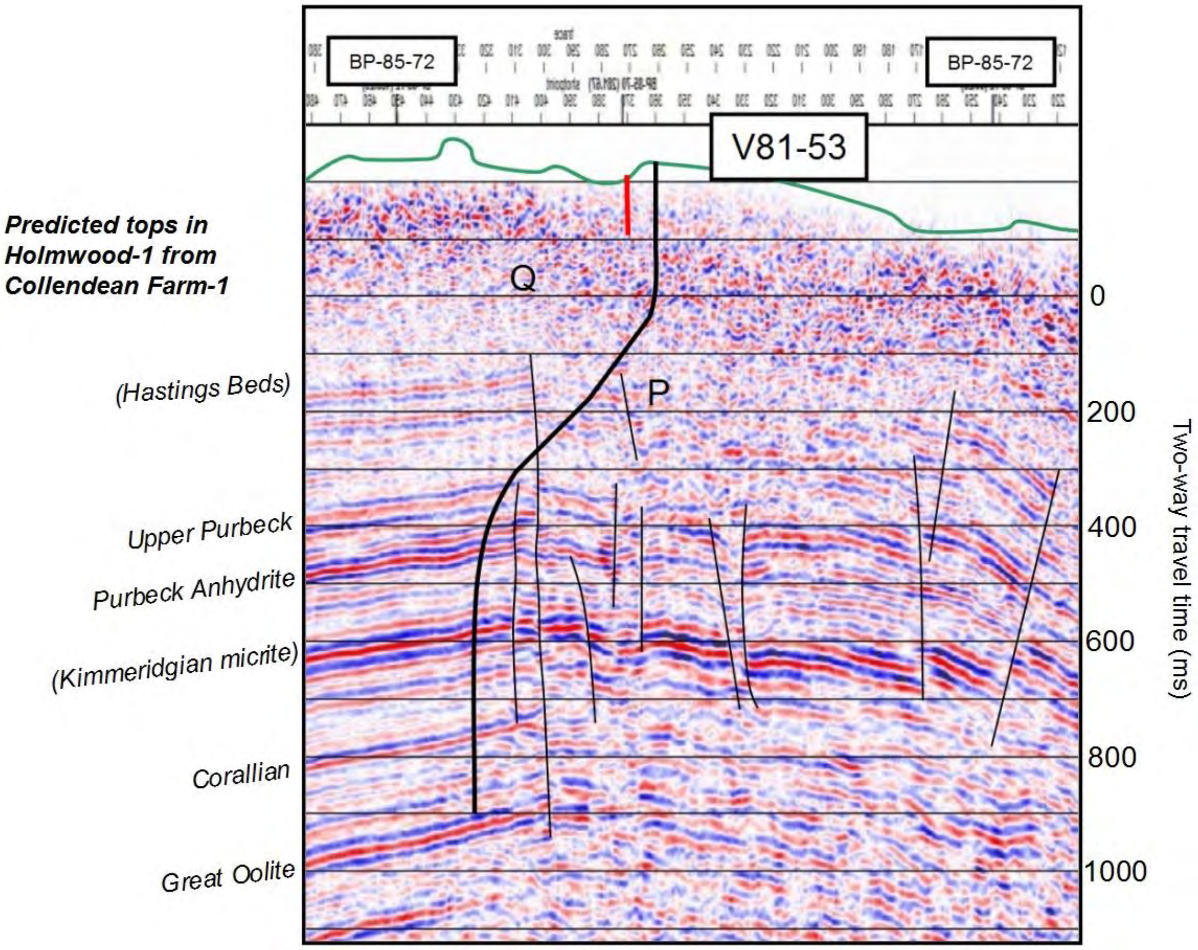

Figure 4.2. 'Squash-plot' of V81-53 along the wellbore track (heavy black line). Faults are indicated by thin black lines. The horizons at the south side (left hand side) are tied in from Collendean Farm-1. Not that fault Q extends upwards to 100 ms, and could be imaged even shallower, but for the poor quality of the shallow seismic data.

The wellbore trajectory is shown by the Z-shaped path; the exact shape of the bend is approximate, because I do not have access to an accurate time-depth conversion; however, the initial and final points are accurate. The vertical red line at the top just south of the wellbore is a BGS-mapped surface fault, which appears in the 1933 published geology map, but is omitted from the 1:10K digital database.

The principal point of note is that fault Q clearly cuts the wellbore, and displaces the Hastings Beds. Tha fault trace can be identified in the upward direction to about 100 ms TWT. Above that depth it is not necessarily absent; it is just not imaged (if it is present) on the shallowest portion of the seismic data.

The Applicant's version of the geology along the well trajectory

Figure 4.3 shows the Applicant's new version of its well trajectory and revised horizons superimposed upon the geology as interpreted three years earlier.

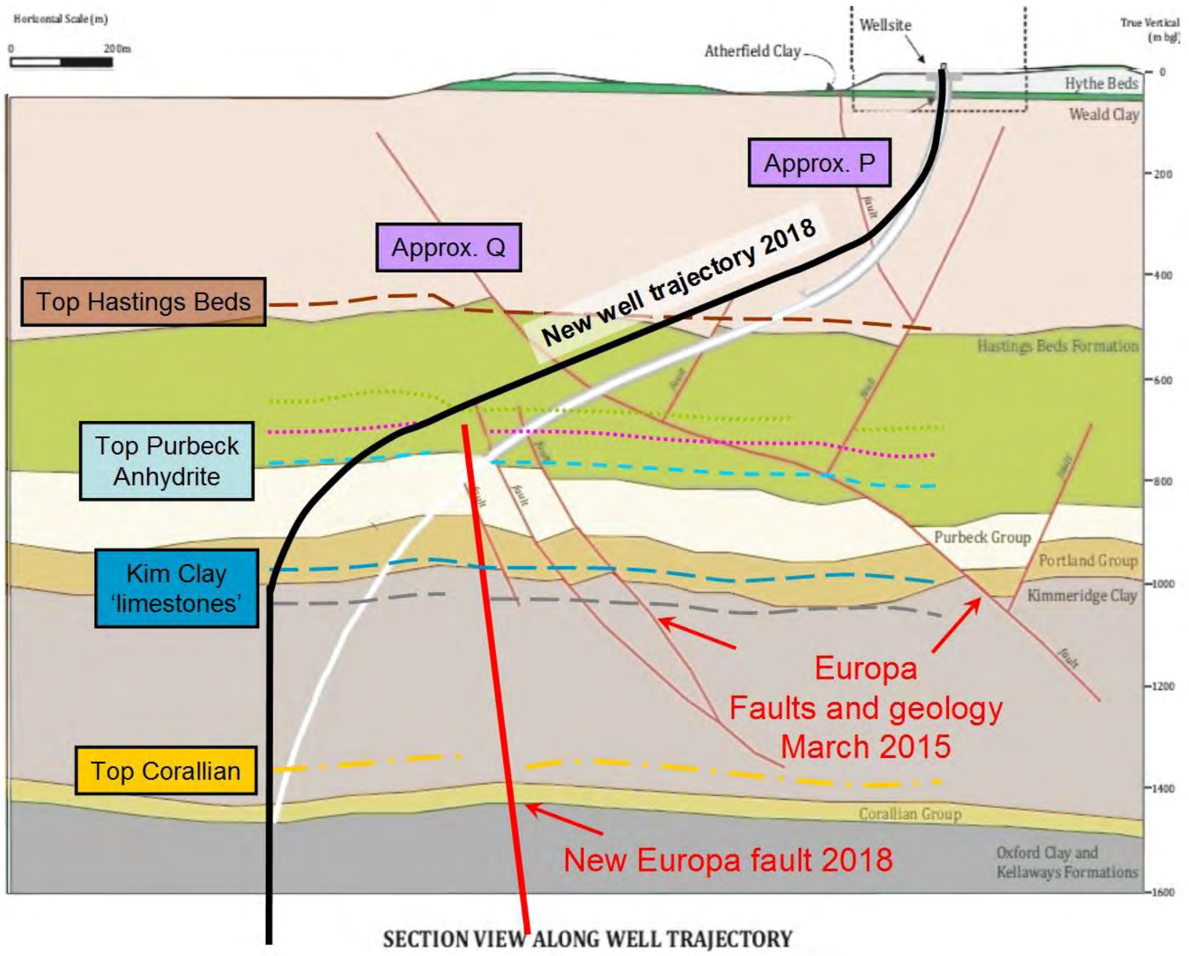

Figure 4.3. Revised well trajectory (black line) and revised horizon tops (coloured dashed lines, labelled), superimposed upon the Applicant's previous version (2015) of the geology. The approximate positions at shallow depth of faults P and Q from Figure 4.2 are marked by labels in purple boxes.

The new position of a fault (bold red line in Figure 4.3) has necessitated the upward shift of the deviated portion of the wellbore from that shown in white. In addition the Applicant has designed the wellbore to intersect the Top Purbeck Anhydrite just south of the new fault.

Firstly, it appears that almost all of the faults from 2015 (thin red lines in Figure 4.3) have now been discarded, as shown by the fact that the revised tops now run across the cross-section with no offsets. This suggests that the Applicant's interpretation of the geology was unsound in 2015, and there is no reason to suggest that it is any more sound now.

Secondly, the Applicant's new fault corresponds to my location for fault Q, as can be seen by comparison of Figures 4.2 and 4.3. However, in my interpretation it continues upwards to cut the Top Hastings Beds (Figure 4.3) where the Applicant indicates merely a small monoclinal feature in that horizon at around 420 m bgl. So this fault cuts through the geology at a crucial location in the cross-section, some 50 m north of the 'design point' of the wellbore, and where the inclination of the wellbore is running at what the Applicant concedes to be at the limit of its technical capacity. The limits of the new wellbore design have been pointed out independently in the hydrogeological review by EGG (2018).

Hydrogeology

Hythe Formation

The Hythe Formation is a Principal Aquifer within the Lower Greensand Group. It crops out at the wellsite. It is unconfined, and underlain by the Atherfield Clay Formation.

Envireau Water (2015) prepared a hydrogeological risk assessment for the Applicant in March 2015. It stated:

> *"Several springs are indicated on the OS map to be present in the valleys to the east and west of the wellsite. Whilst there are no mapped springs in close proximity to the wellsite, it is reasonable to assume that a spring line may be present along the intersection between the permeable sandstone bedrock (Hythe Beds) and the underlying mudstone (Atherfield Clay Formation). Springs may be present in closer proximity to the site than indicated by the OSmap. The significance of springs is described in more detail in Section 5.1."* [section 3.2]
>
> ...
>
> *"The regional groundwater flow direction is expected to be northwards and locally, flow direction is expected to be variable on account of topography and surface water features. Groundwater flow directions in the Hythe Formation in the vicinity of site are likely to be westwards towards Pipp Brook.*
>
> *As described in Section 3.2, the Ordnance Survey map indicates that several springs are present in the valleys to the east and west up to 500m from the site. The springs are most likely issuing at the intersection between the Hythe Formation and the underlying Atherfield Clay Formation. Whilst there are no mapped springs in close proximity to the wellsite, it is reasonable to assume that a spring line may be present along this intersection and springs may be present in closer proximity to the site in addition to those indicated on the Ordnance Survey map.*
>
> *The springs provide baseflow to Pipp Brook, which has eroded the Hythe Formation and exposed the Atherfield Clay Formation at surface. The Hythe Formation at the site is therefore effectively disconnected from the Hythe Formation northwest of Pipp Brook. It is however hydraulically possible that some of the groundwater issuing from springs and flowing into Pipp Brook could infiltrate into the Hythe Formation northwest of Pipp Brook,where it is targeted for public water supply downstream of the wellsite.*
>
> *The absence of a direct groundwater pathway between the downstream public water supply and the wellsite is consistent with the view of Peter Brett Associates; as outlined in Sections 3.2 and 3.5 of their letter to Surrey County Council in January 2015 [Ref. 3]."* [section 5.1]

The assessment goes on to describe a conceptual hydrogeological model, supported by a map and cross-section (fig. 4a) which I reproduce for reference in Figure 6.1.

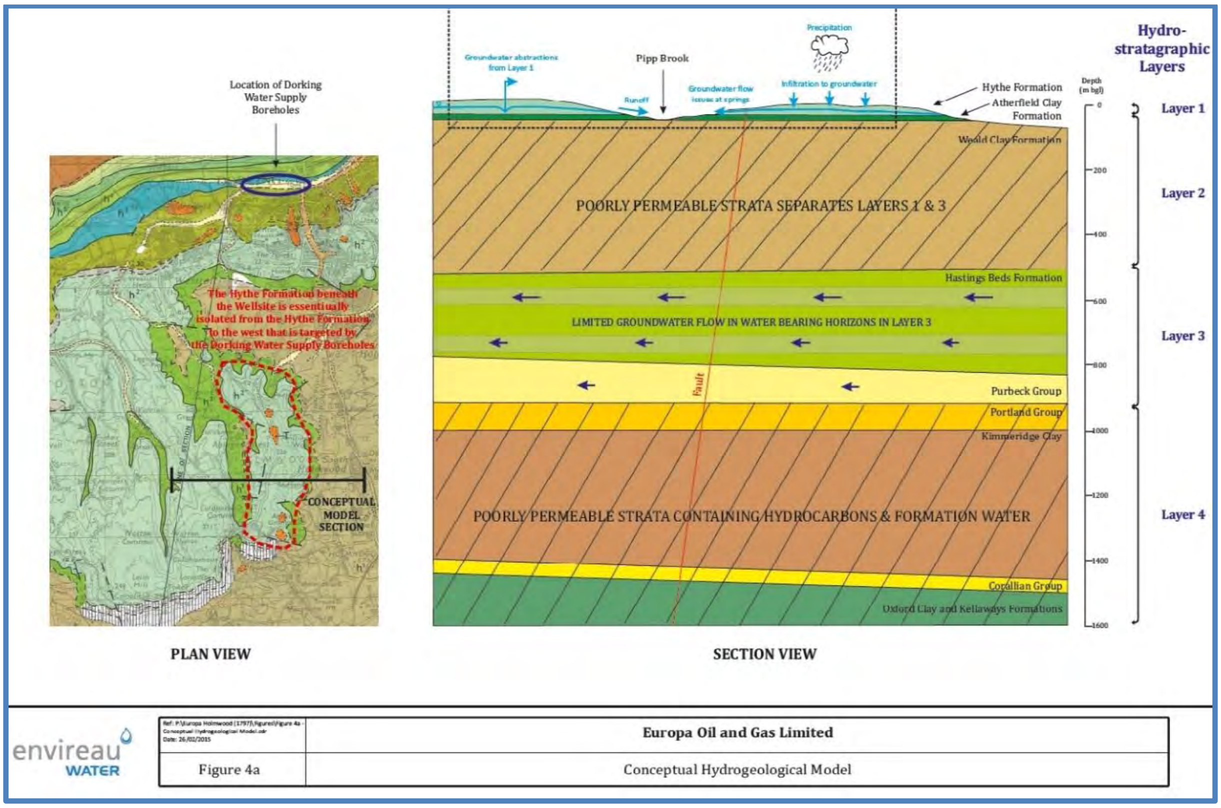

Figure 6.1. Envireau map and E-W cross-section figure 4a.The location of the Dorking public supply boreholes is shown in the elliptical area in the geology map on the left. The geological cross-section on the right is located by the horizontal bar in the map (contains British Geological Survey materials © NERC 2018).

There are several serious errors with this model. Firstly, it relies on an out-of-date version of the solid geology, the Reigate sheet no. 286, for which the geological field mapping was carried out 90 years or more ago. As a result, the E-W cross-section shown in Envireau's figure 4a is inaccurate. In addition, the claim that there is no groundwater pathway between the well site and the public water supply is incorrect.

I have mapped the base of the Hythe Formation, using the modern BGS 10K digital database together with the best available DEM. The contoured result is shown in Figure 6.2a. The control points for the contours are the elevations along the outcrop of the base of the formation, together with the constraint that to the west of Pipp Brook the contours must be below ground level. I have taken account ot the correct sense of throw of the four faults shown in the digital database.

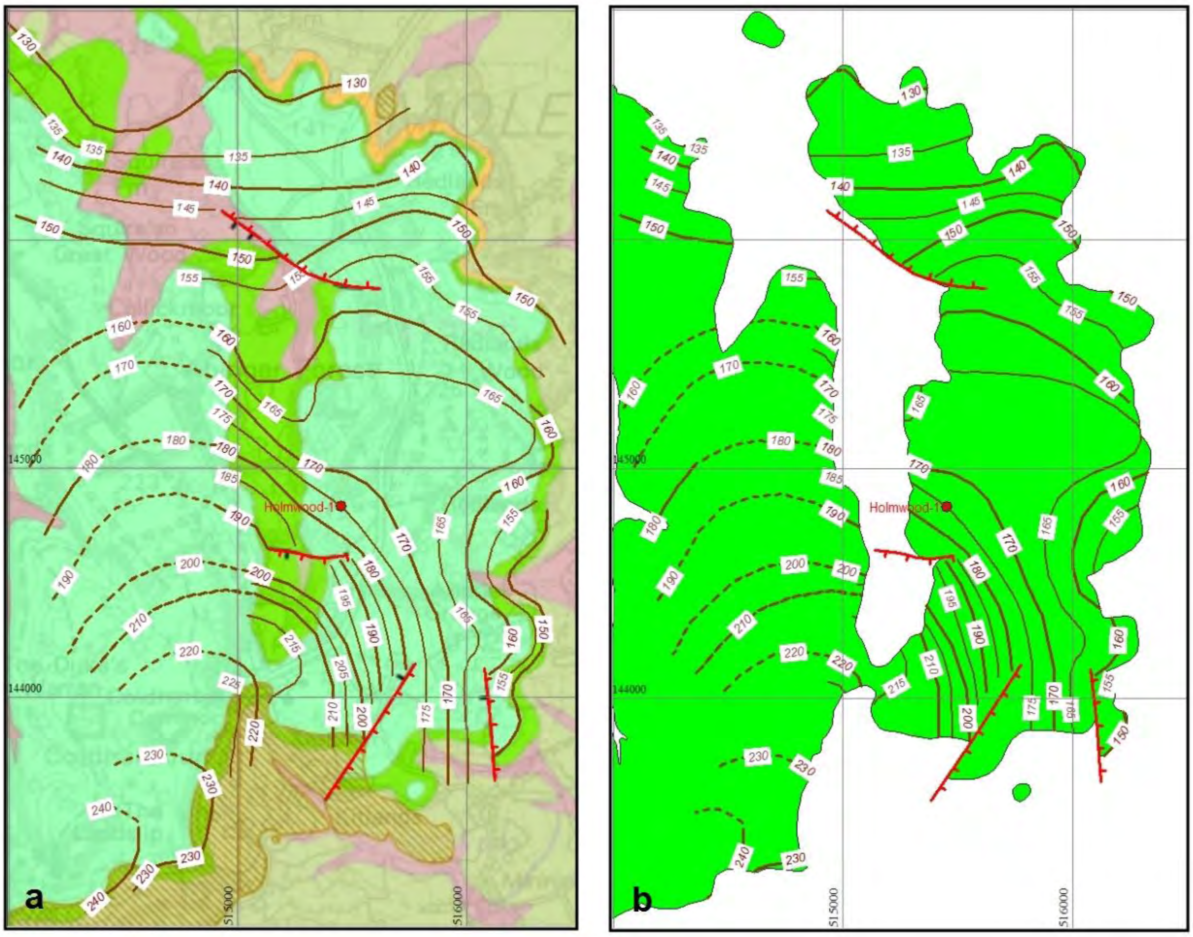

Figure 6.2. a. Contour map of the Base Hythe Formation, labelled in meters above sea level, on the BGS roaming solid and superficial geology image. The Hythe Formation is shown in bright green, the underlying Atherfield Clay in darker green.

b. Contour map of 6.2.a cropped to the Hythe Formation outcrop (contains British Geological Survey materials © NERC 2018).

It must be noted that the linear features, including faults, in the BGS digital database do not necessarily show the correct sense of throw. The BGS roaming images constructed from this database have about 50% of the faults showing a throw in the wrong sense. I communicated this problem to Professor John Ludden, Executive Director of the BGS, a couple of years ago. The faults need to be individually examined in context in order for the correct throw sense to be marked.

Three of the four faults in Figure 6.2a have the wrong sense of throw, as can be discerned from the black tick mark visible from the underlying roam image. Only the E-W fault just south of the wellsite is correct. The contours run into the air across the Pipp Brook valley; this is done for continuity; the resulting map with the above-ground contours cropped to the Hythe outcrop is shown in Figure 6.2b.

It is evident from the contours that the E-W cross-section by Envireau is seriously defective. Furthermore, the statement by Envireau that there may be unmapped springs along the western edge of the Hythe outcrop is incorrect, since the consistent easterly to north-easterly dip of the Base Hythe horizon explains why there are no springs just west of the drillsite. This is shown in Figure 6.3, where all the springs, wells and issues taken from the OS 10K map have been marked.

There is one issue in the Pipp Brook valley at Crockers Farm some 650 m SSW of the wellsite, which evidently originates at the NE-dipping base of the Hythe Formation at around 220 m elevation about 200 m to the SW. The only other issue in the Pipp Brook valley is

north of the wellsite at Collickmoor Farm, and probably originates at the base of the formation some 125 m to the west.

Therefore there are no springs or issues which could be said to originate at the west side of the Hythe Formation outcrop encompassing the wellsite. In contrast there are about ten issues along the eastern flank of the Hythe outcrop to the east of the wellsite, and a further eight along the northern flank, adjacent to where the dip is northerly. In conclusion, the potential problem of contaminated run-off is not to the west, into Pipp Brook, as presumed by the Applicant, but to the east into the Mole catchment.

An accurate geological cross-section along the E-W line of Envireau (see Fig. 6.1) is shown in Figure 6.4, at a vertical exaggeration of x5 (lower section) and compared with the Envireau version compressed horizontally to about the same scale (upper section). The main error in the Envireau cross-section lies in portraying the Atherfield Clay as flat-lying in E-W profile.

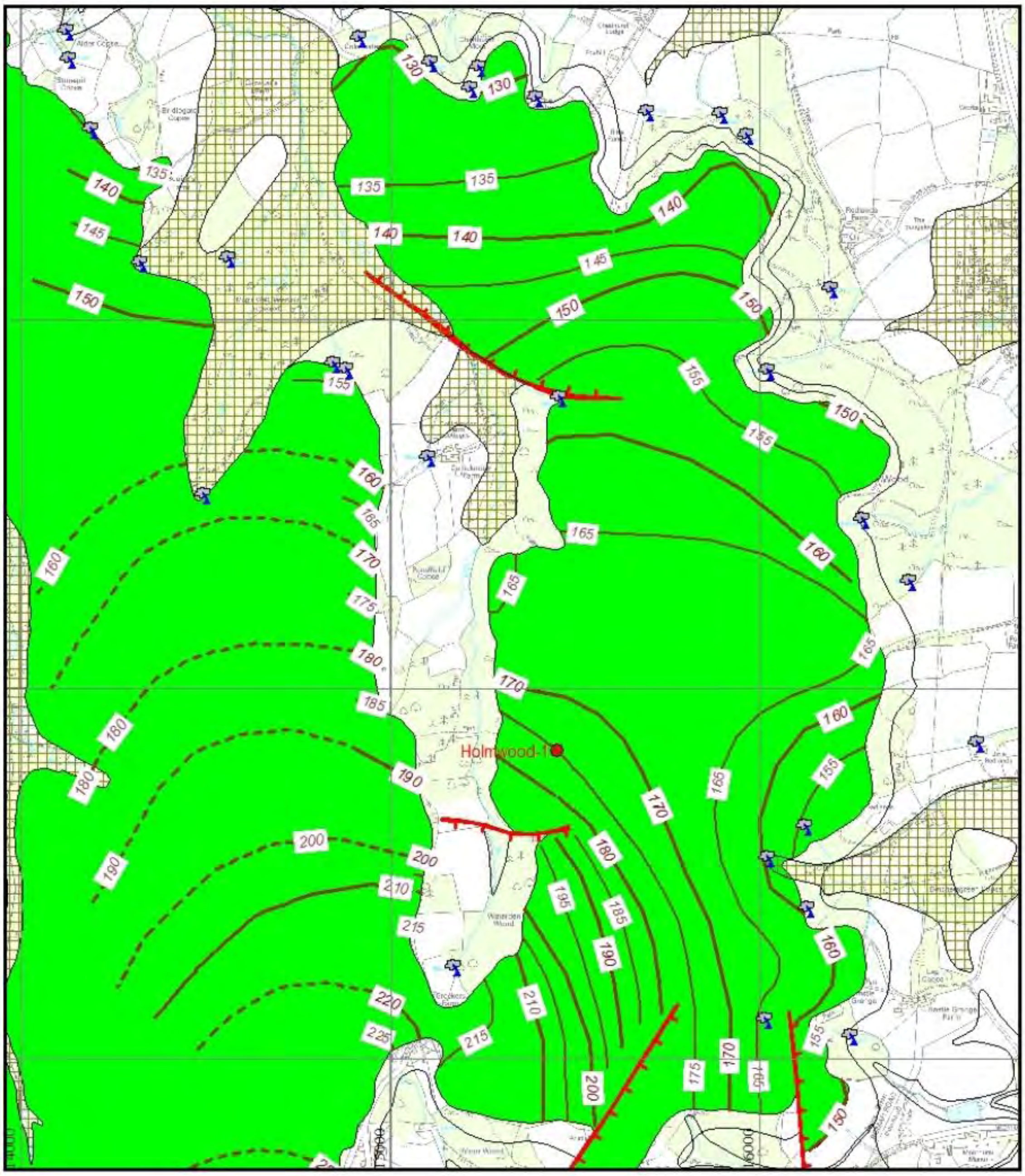

Figure 6.3. Springs and issues (spouting water symbol) around the Hythe Formation outcrop (green). Faults are shown in red; the Applicant's wellsite is shown by the red dot. Superficial deposits are shown by cross-hatching (contains British Geological Survey materials © NERC 2018).

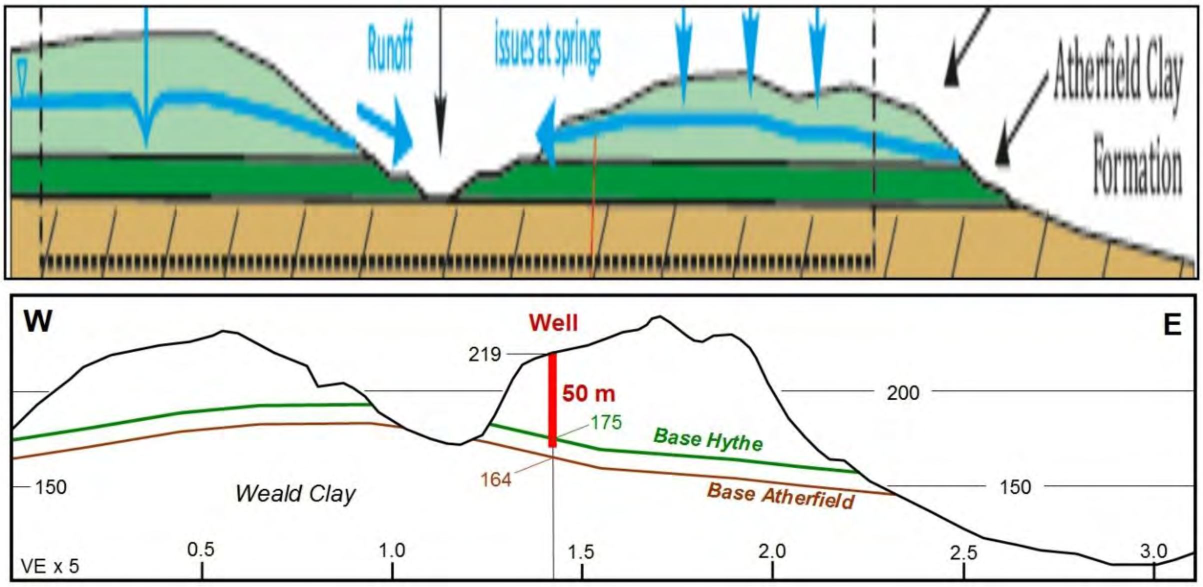

Figure 6.4. Revised and corrected E-W cross-section along the line shown in the map of Figure 6.1 (lower) compared with the Applicant's version (upper) scaled to approximately the same dimensions.

Hydraulic continuity to the public supply wells

The Applicant asserts that the Hythe Formation outcrop at the wellsite is hydraulically isolated from connection to the public supply boreholes at Dorking. This is incorrect. Figure 6.5 shows a combined solid and superficial geology map, in which only the permeable solid formations have been coloured, and superimposed on those are the superficial Head and Alluvium deposits indicates by cross-hatching. There is a continuous permeable pathway from the well, northwards and with a downdip component, to the water supply boreholes indicated by the mauve triangles at the top of the map. One such path is illustrated in cross-section along the blue line A-G, of which the part A-F is shown in Figure 6.6.

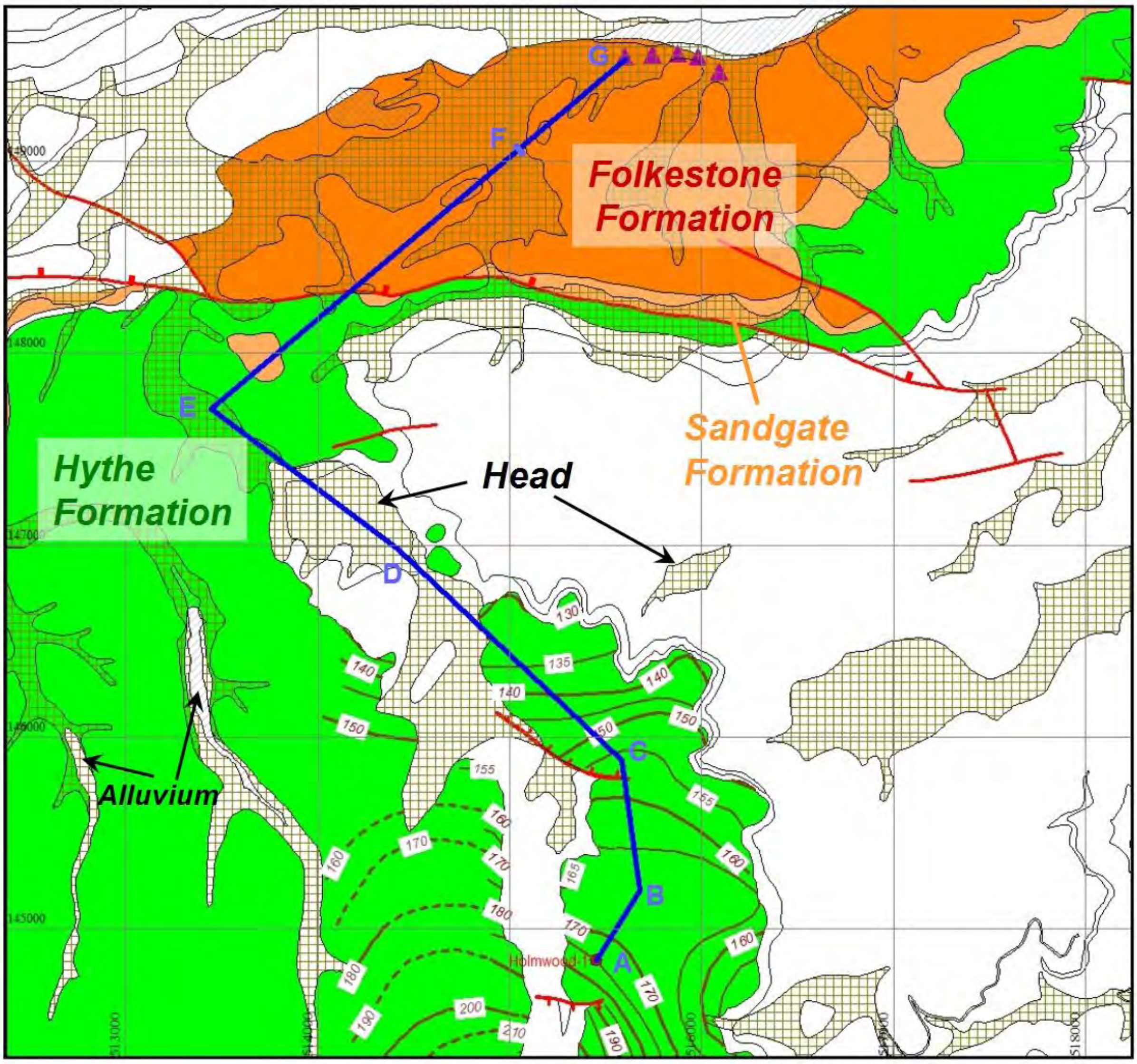

Figure 6.5. Permeable solid geology formations coloured: Hythe Formation - green; Sandgate Formation - orange; Folkestone Formation - orange-red. Other impermeable formations are left uncoloured. Cross-hatched areas over the solid geology comprise permeable Head and Alluvium. The blue path A-G from the Applicant's wellsite at A to the most westerly public water supply well at G is shown in Figure 6.6. Digital data from the BGS (contains British Geological Survey materials © NERC 2018).

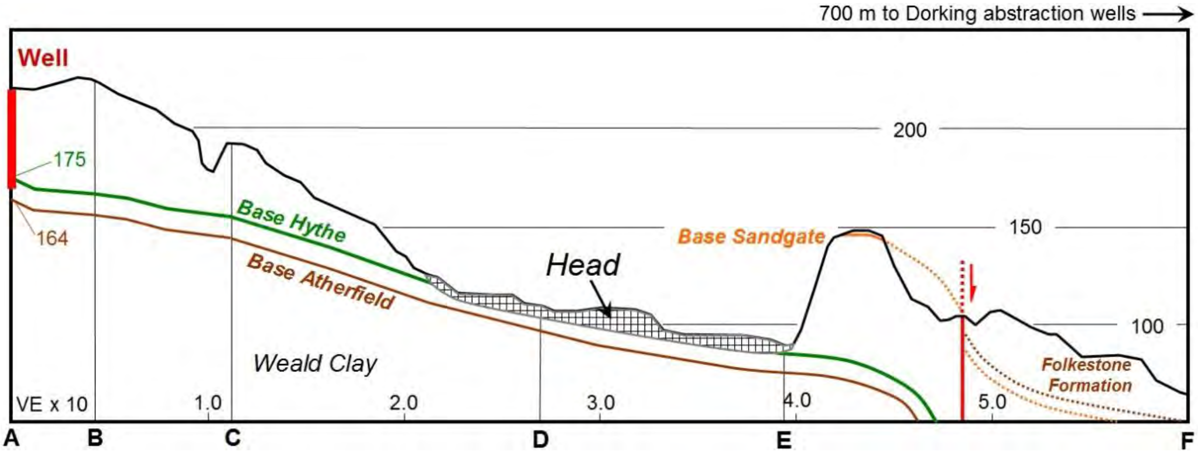

Figure 6.6. Shallow geological profile along section A-F shown in Figure 6.5. The proposed well is at A. Vertical exaggeration x10.

Figure 6.6 shows the connection northwards from the Hythe Formation into the Head, for some 2 km, and then back into the Hythe. The profile is constructed only from points A to F, but the profile continuation to point G, some 700 m further to the NE, stays within the permeable Folkestone Formation at outcrop. Note the presence of the major fault running for some 6 km in an east-west direction through Dorking. This fault was not recognised on the old BGS published 1:50,000 sheet. It cuts a northerly-verging monocline with a downthrow to the north, and in the area of interest it appears to have a vertical component of displacement of at least 20 m, as estimated from nearby outcrops of the Sandgate Formation along either side of the fault trace.

Therefore the claim by the Applicant that the Hythe Formation around the wellsite is hydraulically isolated is wrong.

Protection of the Hythe Formation at the wellsite

The Base Hythe Formation is at 175 m (Figure 6.3). The error in this figure is probably no more than ±1 m or so. The ground surface at the wellsite is at 219 m, therefore the base of the proposed 50 m of 20 inch conductor casing will be at 169 m above datum. The Hythe Formation is supposed to be protected by a 20 inch conductor casing to a depth of 50 m TVD from ground level (Europa 2018, table 5.1). However, the accurate shallow geological cross-sections of Figures 6.4 and 6.6 show that this is insuffcently long (the red line in the figures). The bottom of the conductor at 169 m ASL terminates within the Atherfield Clay Formation, and does not penetrate through to the top of the Weald Clay at 164 m ASL. Does the Atherfield Clay Formation act as a robust aquitard?

Several of the mapped springs shown in Figure 6.3 around the eastern edge of the Hythe Formation outcrop appear to originate, not at the base of the Hythe, but at the base of the Atherfield Clay Formation. This formation is depicted in Figure 6.3 by the uncoloured narrow outcrop around the edge of the Hythe outcrop. That some of the spring locations are at the base of the Atherfield, and not at the base of the Hythe, has been independently pointed out in the submission by EGG Consulting Limited (EGG 2018, p. 22).

The BGS memoir for the Reigate sheet 286 (Dines and Edmunds 1933) describes the Atherfield Clay Formation thus:

"The beds are of marine origin, and consist of red-brown, blue or yellow clays, sometimes mottled and often sandy or silty. A sandy basement bed is known as the Perna-Bed, in which nodules of fossiliferous ironstone are frequently found, particularly near the base. Godwin-Austen noted that the Atherfied Clay of Surrey contains "subordinate nodular concretions in the lower part of the bedding of great size and thickness, and cemented into an exceedingly hard rock by calcareous matter." "

The modern BGS lexicon describes it as:

"Generally massive yellowish brown to pale grey sandy mudstone throughout most of its outcrop, with an impersistent phosphatic pebble bed with vertebrate bones, gritty sandstone or very shelly sandy mudstone with glauconite, at the base."

So the formation is not simply a pure clay as implied by its name. Therefore the Atherfield Clay Formation may not be the impermeable layer assumed by the Applicant.

In conclusion, the termination of the 20 inch conductor casing within the Atherfield Clay Formation at 169 m above datum provides inadequate protection of the Hythe Formation.

Conclusions

The Applicant claims that its permit is for a purely conventional exploration drilling programme, when in fact its new proposals include unconventional testing by matrix acidisation. The Kimmeridge Clay Formation, with its tight thin semi-limestone 'micrite' bands, is an unconventional target.

Whether in pursuit of conventional or unconventional targets, the Applicant should be required to acquire additional 2D seismic data, or preferably 3D seismic, and interpret them before pursuing its objectives at the Holmwood-1 site. The application for a permit has the following serious weaknesses and problems which need to be addressed:

- Use of out-of-date geological mapping information.
- Problems of shallow faulting from old and new BGS information not considered or reconciled.
- Poor understanding of shallow geological structure of the Hythe Formation principal aquifer below the wellsite, leading to misleading conclusions on groundwater flow directions.
- Proven hydraulic continuity *via* Lower Greensand formations and unconsolidated deposits from the wellsite to public supply wells at Dorking.
- Shallow geological structure includes poorly-understood faulting, with a thrust fault near the wellsite.
- Conductor casing too short and does not penetrate into the Weald Clay.
- Hastings Beds cut by a fault in vicinity of the wellbore.
- Insufficient seismic reflection information properly to define the faulting and target structures.
- Lack of evidence presented to justify geological structures.
- Lack of justification for seismic ties to existing wells.
- No evidence presented for time to depth conversion of the seismic data.
- Two promised seismic reflection lines never obtained.
- Equidimensional and complex faulted nature of the target structures necessitates a 3D seismic survey for accurate characterisation.
- Redesigned wellbore at the very limit of technical capacity, with no leeway for manoeuvre.
- Likelihood of cement bond failure along wellbore at shallow angle.
- Unconventional (tight, low permeability) target micrites added to the work programme at a late stage despite claim that prospects are conventional.
- Confusion between acid wash to clear borehole and stimulation of unconventional formations to enhance flow.

The information supplied by the Applicant is incomplete and misleading. The problems summarised above lead to the inescapable conclusion that the Applicant has a poor understanding of the geology, and of the technical problems that it is likely to encounter in drilling. In turn, its understanding of the hydrogeology is seriously defective. In consequence there is a serious risk that the drinking water aquifers in the district may be contaminated by the Applicant's proposed activities, both in the short term and in the long term.

In conclusion:

The Environment Agency should refuse the environmental permit.

Celtique Energie at Fernhurst and Wisborough Green in the Weald

Critique of presentation by Professor Peter Styles to LDNPA

This is an abbreviated critique of the presentation that Professor Styles made as a presentation to the South Downs National Park Authority on 15 October 2013. I have no access to what he said, only a pdf copy of his slides. Based on what can be inferred from the slides, there appear to be a number of errors and omissions:

- He appears to argue that one cannot distinguish between conventional and unconventional exploration (slides 9-10). This is incorrect.
- He implies that fracking is not new (slides 14-15) – this is untrue. Fracking of the high-volume slickwater type in horizontally deviated wells has only been around for less than 15 years. Previous types of fracking (which are generally safe) consume only modest quantities of water by comparison with what the public is concerned about.
- His slide no. 18 illustrating by a cross-section of a fracking well omits any depiction of faults. The geology is grossly oversimplified.
- Micro-seismic mapping of the progress of fracking is not the whole picture (slide 20). There is evidence of frack fluid progressing up a fault to a new level; this progress is silent, i.e. unaccompanied by the tell-tale signs of microseismic tremors, because a pre-existing fracture has been used.
- He uncritically quotes a paper by Professor Richard Davies of Durham University (slides 21-23) on the empirical limit of how far fracks can progress upwards, which in turn refers uncritically to a questionable earlier study by Halliburton.
- Styles suggests that 'good quality cementing' will protect wells from leaks (slides 23-25). This ignores the fact that all wells will degrade and leak in the long term. His attempt to use the Roman Pantheon as an example of the supposed longevity of concrete is inappropriate.
- He suggests (I presume) that the Wytch Farm oilfield development in Dorset and below Bournemouth Bay is environmentally safe. I agree with this, but there is no valid comparison with the kind of fracking proposed in the UK shale basins.The 'extended reach' wells drilled out eastwards under the bay are through (or targeting on) the Sherwood Sandstone, the oil reservoir, and were never fracked (Hogg *et al.* 1999).
- He points out that the magnitude 2.3 Blackpool earthquake of 2011 is exceptionally large (slide 41), by comparing it with the thousands of far smaller earthquakes generated by fracking in the Barnett Shale of the USA. No explanation is offered (in the slides) as to why this Blackpool earthquake is so exceptional.
- He quotes the main conclusions of the Royal Society report of 2012 (slides 50-51), but this report failed to discuss the differing geological regime in the UK compared with the US.
- Slide 42 states *"Characterisation of any possible active faults in the region using all available geological and geophysical data (BC always has 3-D seismic)"*. This statement, citing a report of which he was a co-author (Green *et al*. 2012) is incomplete, as it should have included all faults, not just 'possible active faults'.

In summary, I find Style's views on the risks of fracking to be complacent and incomplete. The precautionary principle suggests that any region or rock volume cut by faults, whether active or inactive, should not be fracked.

IGas at Springs Road, Misson
[Edited extracts from my objection[1] to drilling at Springs Road]

Geology of the Gainsborough Trough

The Gainsborough Trough is a basin hidden below the younger rocks that are seen ('crop out') at the surface. Figure M1 shows the Misson Springs proposed wells in the context of the solid geology, that is, the solid rocks with the superficial sediments removed. There is a very gentle dip of around 2° to the east as shown by the black arrow. A cross-section accompanying the British Geological Survey (BGS) 1:250,000 regional scale Humber map happens to run through the proposed site. Part of this is reproduced in Figure M2. These two figures – map and section – show that the older Coal Measures (Carboniferous age) crop out in the west, but are successively buried by younger rocks as one proceeds eastwards.

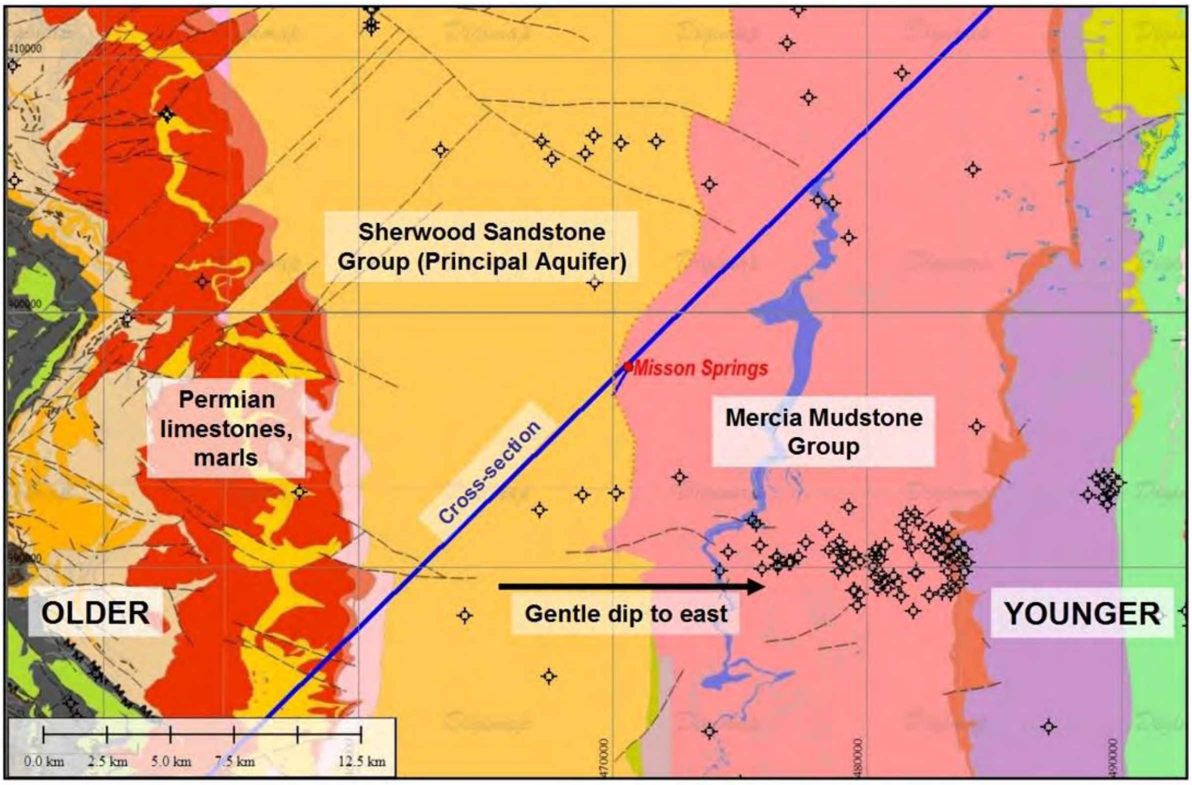

Figure M1. Regional solid geology around the Misson Springs site (red dot). The location of the BGS regional cross-section (reproduced in Figure M2) is shown by the blue line. Hydrocarbon wells are shown by the circle/cross symbol. The geology goes from old to young in an eastward direction.

The Sherwood Sandstone Group (SSG) is a Principal Aquifer. Figure M2 shows that the Applicant proposes to drill through a few metres of Mercia Mudstone Group (MMG), then through the SSG and older rocks. The target rock is the Bowland Shale beneath, at 2000-2500 m.

The Gainsborough Trough, containing the Bowland Shale (Bowland-Hodder Unit), underlies the Millstone Grit. The BGS regional structural map reproduced in Figure M3 (Andrews 2013) shows that the Trough is aligned NW-SE, hidden below the younger east-dipping sediments shown in Figure M1. The word 'trough' is usually used when a narrow basin is faulted on one or both sides. Red shading is shallow depth, yellow through to blue indicates progressively deeper depths, and faults cutting the base of the basins are shown as red lines. It can be seen that at this regional scale, covering a large part of northern England (Figure M3a), the

[1] Smythe, D.K. 2015. Planning application no. ES/3379 by Island Gas Limited to drill at Springs Road, Misson, Nottinghamshire: Objection on grounds of geology and hydrogeology. 15 December 2015, v1.1, 58 pp. (pdf)

Gainsborough Trough is less faulted than the Bowland Basin of West Lancashire, and is not as deep as the Cheshire Basin. The Applicant's area of search, PEDLs 139 and 140, is shown outlined in red with black hatching in Figure M3b. The proposed site is shown by the red dot.

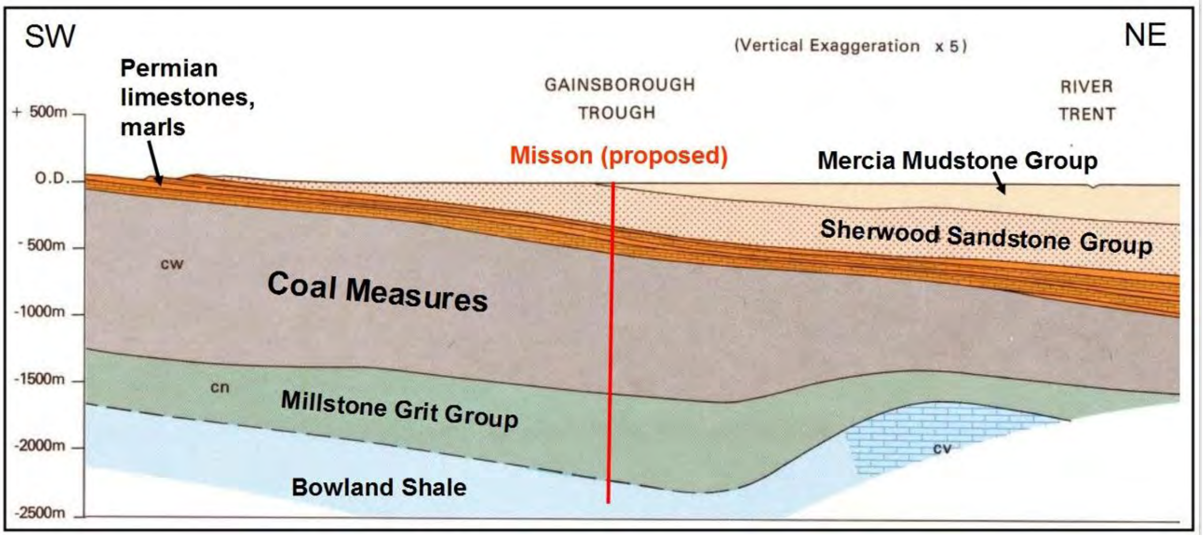

Figure M2. Part of the BGS regional cross-section from the 1: 250,000 Humber map, which passes through the Misson site (red line). The extract, which is about 40 km long, runs from Worksop in the SW to Scunthorpe in the NE.

The north-eastern half of cross-section D of Figure M3a is shown in Figure M4, where the target Bowland-Hodder shales are shown in dark green. Note that they are 2-3 km thick.

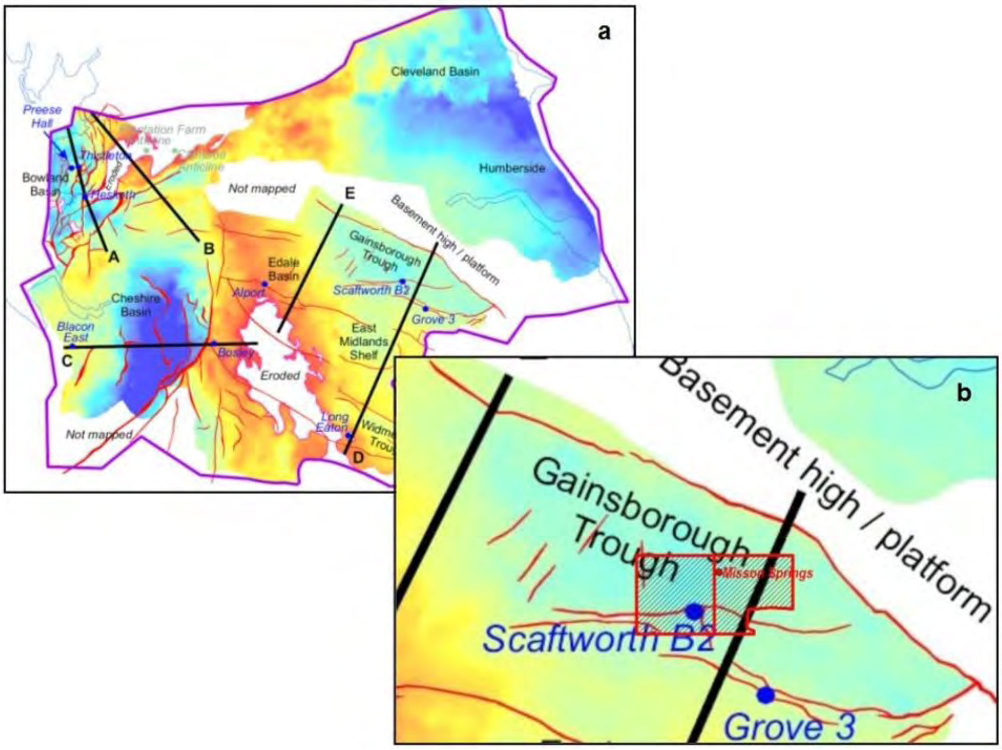

Figure M3. (a) Colour map of depth to the base of the Bowland Shale in the north of England[2]. Dark blue is deepest, red is shallowest.
(b) Detail of the Gainsborough Trough showing the Misson Springs well (red dot) and the two contiguous PEDLs. Part of cross-section D is shown in Figure M4.

[2] Andrews, I. J. 2013. The Carboniferous Bowland Shale gas study: geology and resource estimation, British Geological Survey for Department of Energy and Climate Change, London, UK https://www.gov.uk/government/publications/bowland-shale-gas-study

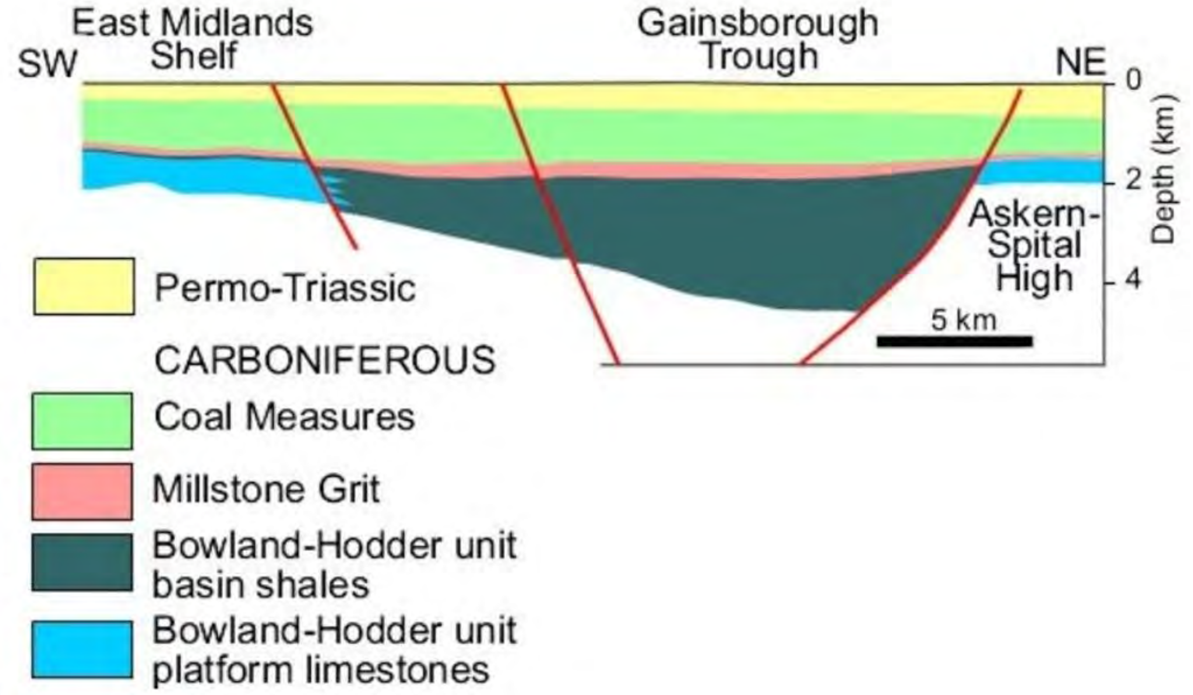

Figure M4. NE end of BGS cross-section D through the Gainsborough Trough[2].

Inaccurate and insufficient supporting data

Introduction

The current application comprises the first exploratory step both in the East Midlands and in the Gainsborough Trough in which high-volume fracking of horizontal will be eventually be employed, if a later appraisal application is approved. As such, it is incumbent on the applicant to supply full justification for the current stage of the work, and why the whole appraisal is not included in the current application. This has not been done.

Existing well and borehole data

IGas Appendix F Groundwater, section 4.6, of the application discusses the bedrock geology, with reference to borehole SK79NW/30. This is the Rocket Site borehole shown in Figure M5, situated 540 m SW of the vertical wells and above the proposed horizontal well (the latter is shown in blue). But this borehole is not shown on the relevant Application Drawing no. 22, which forms the backdrop to Figure M5. Only five boreholes are marked on this drawing, shown by the black dots labelled with the well name in upper case.

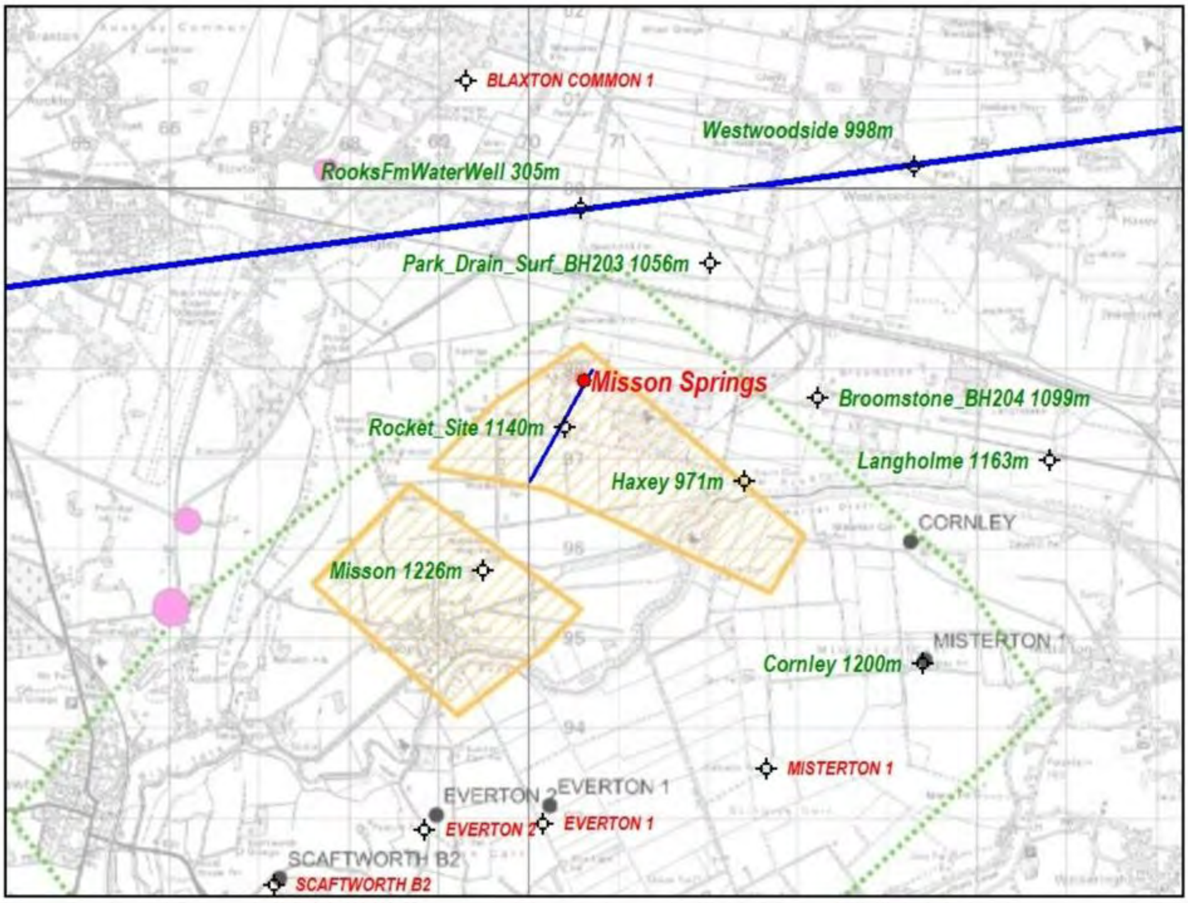

Figure M5. Reproduction of part of the Applicant's figure 22 showing the 3D seismic surface coverage area (green dotted line) and the detailed search areas (yellow hatching). Existing hydrocarbon wells as positioned by the Applicant are shown as black dots; the correct positions are shown by the circle/cross symbol and red upper case label. Deep water and coal boreholes are shown with their name and depth in green. The location of the BGS cross-section from Sheet 88 Doncaster, 1: 50,000 map is shown by the blue line.

The most distant borehole depicted by the Applicant is Scaftworth-2, which lies 6.6 km SW of the Misson site. But there are a further 8 boreholes, excluding Rocket Site, which should also have been taken into consideration, because they are nearer than Scaftworth-2. All these relevant boreholes are shown in Figure M5 and tabulated with correct grid coordinates in Table 1. Every one of the five boreholes depicted by the Applicant is mispositioned. The positioning error varies from 80 m in the case of Scaftworth-2 up to 2130 m for Misterton-1. Such gross errors are inexcusable and misleading.

The Applicant concedes that Sherwood Sandstone Group is at solid outcrop below superficial deposits at the Rocket Site borehole, but qualifies this admission: *"... it is possible that part of the recorded 'Drift' (superficial deposits) represents weathered Mercia Mudstone*." (Appendix F section 4.6). Evidence for the solid rock outcrop around the site is discussed below. The nearest that the Applicant comes to portraying a geological cross-section through the proposed wells comprises two cartoons, reproduced herein as Figure M6, purporting to show the strata to be encountered. The errors in thicknesses depicted in these cartoons are discussed below.

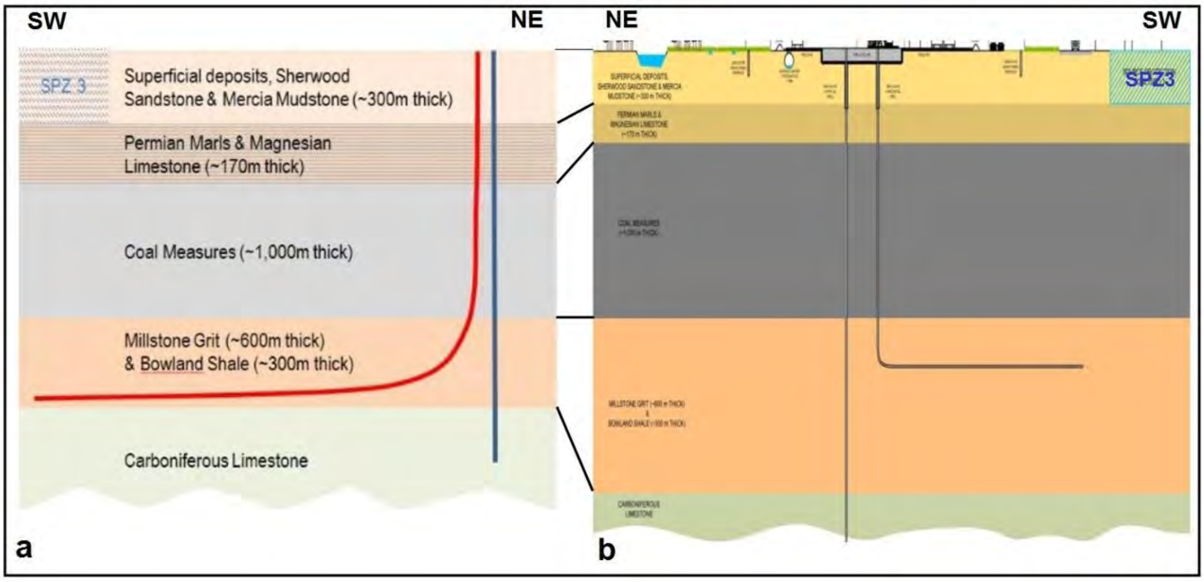

Figure M6. Two cartoons from the Applicant's drawings purporting to show the geology to be encountered. The right-hand picture has been mirrored for easier comparison of the two images. The two images have then been scaled so that they match horizontally at the surface and at Top Millstone Grit. There is no horizontal scale. SPZ3 is Source Protection Zone 3, misleadingly marked at the corner of each diagram, far from the wells.

Table 1. Relevant boreholes and wells omitted from or mispositioned by the Applicant.

Easting	Northing	Borehole	Depth (m)	Comments
473238	397691	Broomstone BH204	1099	Missing from Igas map
474401	394740	Cornley	1200	Mispositioned 1350 m to N
472419	396772	Haxey	971	Missing from Igas map
475819	397004	Langholme	1163	Missing from Igas map
469510	395770	Misson	1226	Missing from Igas map
472037	399183	Park Drain BH203	1056	Missing from Igas map
470410	397369	Rocket Site	1140	Missing from Igas map
470600	399800	Rooks Farm Water Well	305	Missing from Igas map
474302	400269	Westwoodside	998	Missing from Igas map
		**Oil or CBM well**		
469320	401215	Blaxton Common-1	957	Missing from Igas map
470175	392960	Everton-1	2079	Mispositioned 200 m to NE
468850	392885	Everton-2	229	Mispositioned 210 m to NE
472662	393576	Misterton-1	560	Mispositioned 2130 m to NE
467179	392283	Scaftworth-B2	2326	Mispositioned 80 m to NE

Two areas of search were identified by the Applicant (Vol. 3 Environmental Statement, section 5.6). These are shown in cross-hatched yellow in Figure M5. The search criteria are stated to be as follows:

"*These were defined as the best areas of search for exploration wells from a reservoir and structure point of view having had regard to factors including geological structure and the thickness and depth of the target strata as identified by the 3D seismic survey*."

This sentence is essentially meaningless without the provision of extra information. The Applicant should have supplied the information required to justify the statement. Figure M5 shows that neither the Misson nor the Haxey well were used by the Applicant, even though they both lie within or at the edge of these two selected areas. In addition, the Rocket Site borehole does not have the necessary electric logs for tieing in well depths to the 3D seismic. Note again from Figure M5 the gross mispositioning by the Applicant of the Misterton-1 and Cornley wells. Therefore the well-to-seismic ties essential for the Applicant's geological interpretation could be grossly in error, and will therefore nullify any (unpublished)

conclusions that the Applicant may have come to regarding geological structure and reservoir properties. This in turn means that the site selection process is demonstrably inaccurate.

Area of search

The Applicant has not demonstrated that the application site presents the best option in comparison with alternative sites within the area of search; that is, the contiguous Petroleum Exploration and Development Licence (PEDL) areas PEDL 139 and PEDL 140. Figure M3b demonstrates that the top of the Bowland Shale is to be found at a similar depth throughout the search area. The BGS report on the Bowland Shale[2] also shows that the shale is at its thickest north of the E-W fault shown in red in Figure M3b running through the Scaftworth-B2 well. So if maximum thickness of shale is a search criterion (but which is not actually stated by the Applicant) this still leaves about 75% of the area of search available.

Such failure contravenes the County Council's Minerals Local Plan (preferred approach), policy MP12: Hydrocarbon Minerals, which states, under the heading Exploration:

"*2. Where proposals lie within an environmentally sensitive area, evidence must be provided to demonstrate that exploration could not be achieved in a more acceptable location and that within the area of search the proposed location would have least impact*."

Given that the site lies just 125 m from the Misson Carr Nature Reserve, an SSSI, it is incumbent on the Applicant to show why, within the total PEDL area available of 182 km2, no better site could have been selected.

The choice of the two areas of search within the overall PEDL search area, that is, the two yellow hatched polygons in Drawing 22 (reproduced with overlays in Figure M5), is not justified by the imprecise definition under the Applicant's heading Areas of Search:

"*the best areas of search for exploration wells from a reservoir and structure point of view having had regard to factors including geological structure and the thickness and depth of the target strata as identified by the 3D seismic survey*." (Vol. 3 Environmental Statement, para.5.6.1).

We need further information. In addition, there is another unjustified assertion under the heading Site Selection:

"*The significance of the areas of search in the context of site selection is that drilling from locations outside the boundaries would not achieve the objectives of the exploration programme*." (Vol. 3 Environmental Statement, para.5.7.1)

Conclusions on targets and area of search

Given the fact that the primary target, the Bowland Shale, can be accessed essentially anywhere within the PEDL region, totalling 182 km^2 in area, we need much more detail as to what exploration criteria resulted in a narrowing down of the search to just 8 km^2 – some 4% of the PEDL licence area available.

It would also be surprising that the area of search for the secondary, conventional target, sandstones within the Millstone Grit, could be narrowed down to one or both of the tiny areas selected; but if that were indeed the case the presumed conventional target, such as a four-way closure, must be so small as not to be worth the effort.

The inevitable suspicion therefore arises that the two search areas were selected on non-geological criteria, in which case the Applicant is misleading the Council.

Fault interpretation in the vicinity of the site

Evidence from the boreholes near the proposed development

The feather-edge of the Mercia Mudstone Group (MMG) in the vicinity of the proposed development, as marked on the BGS Doncaster 1:50,000 map, mismatches the evidence from boreholes which were drilled after the map was published in 1969. But the Applicant persists in using the out-of-date information in this regard. Figure M11a shows the feather-edge of the MMG from the 1969 map (yellow dotted line) in relation to the site and surrounding boreholes. Solid rock encountered in the boreholes is indicated by the old names; Keuper Marl (KM) is now the MMG, and Bunter (Sandstone) is the Sherwood Sandstone Group (SSG). But the feather-edge is discrepant at two localities, circled in red in Figure M11a, where SSG was drilled at solid rock outcrop, and not MMG, as would be expected from the mapped boundary.

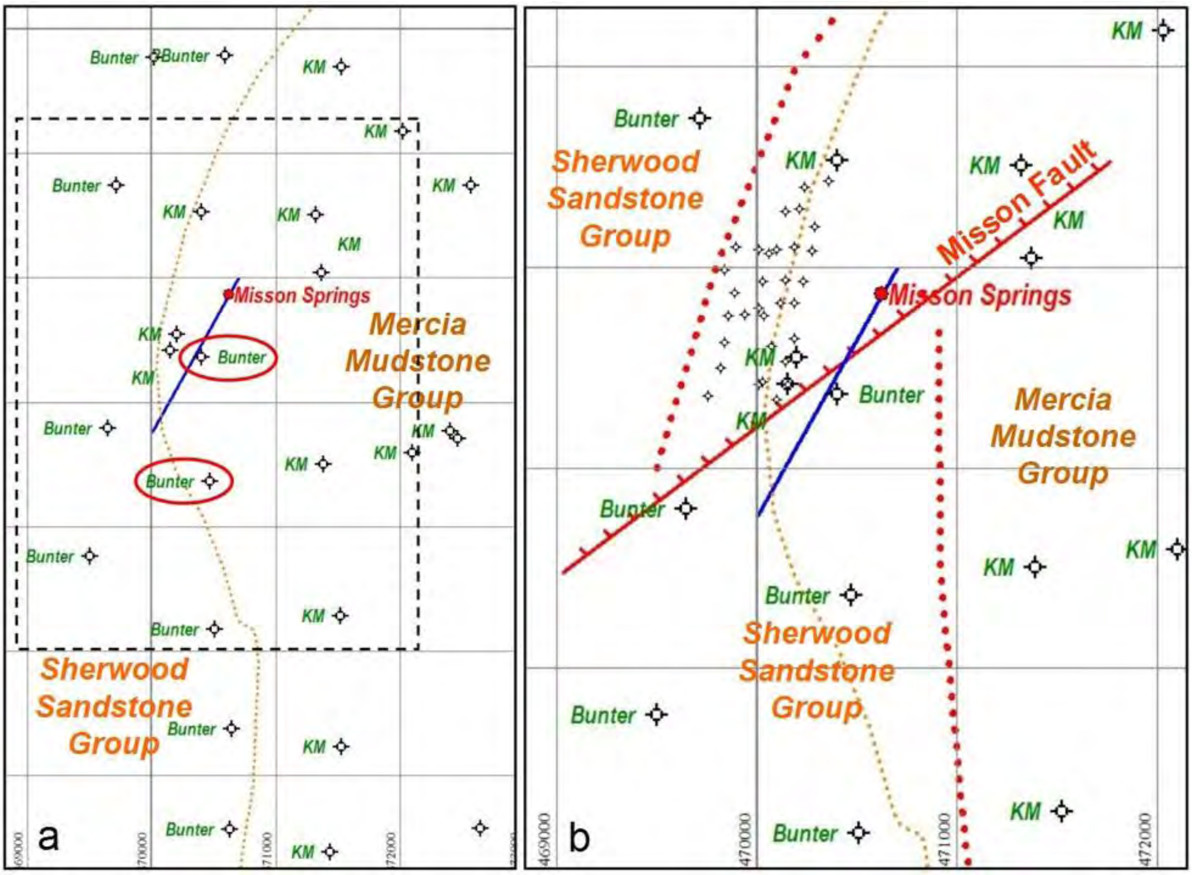

Figure M11. (a) Shallow boreholes proving Bunter (= Sherwood Sandstone Group) or Keuper Marl (KM = Mercia Mudstone Group) at subcrop below drift (unconsolidated material). The feather-edge of the MMG (the western limit where it wedges out), taken from the BGS Doncaster geology map, is shown by the yellow dotted line. But this boundary mismatches the two boreholes outlined by the red ellipses.
(b) Detail of the site locality (the black dashed-line rectangle in Figure M11a). The revised location of the MMG boundary is shown by the two red dotted lines. These honour the solid rock data as shown by the boreholes. Given the absence of folding, the only feasible way to explain the offset of the boundary of about 1800 m is by faulting. I have labelled this the Misson Fault, with a normal downthrow to the north.
Ordnance Survey gridlines are shown at a 1 km interval.

A more detailed area around the site is shown in Figure M11b, which covers the area shown in Figure M11a outlined by the dashed black rectangle. Thirty-one Misson Springs shallow boreholes are shown in this detailed map by small well symbols; all these boreholes indicate that bedrock was either not reached, or that it may have been weathered MMG. So the feather-edge of the MMG has to be revised to honour the data, and this is shown by the heavy red dotted lines in Figure M11b.The only feasible way to account for the offset is by postulating a fault, which I call the Misson Fault, trending NE-SW through the proposed development. It is

constrained to lie north of the Rocket Site borehole, which lies on the trajectory of the proposed horizontal well. Geometrically, the data could be also fitted by a sinistral wrench fault, but since this style of faulting does not occur in this region, such an alternative explanation is unlikely.

The fault may be curved, but interpreting it as rectilinear fits better the fault style in the area. The trend also matches that of the coal seam faults seen in the Rossington colliery data. The vertical displacement on the Misson Fault in the near-surface is of the order of 30 m; this is calculated as the lateral offset of the feather-edge (1800m) multiplied by tan(1º).

The existence of the Misson Fault is supported by the several references to springs in the locality. It is true, as the Applicant observes, that springs would be expected at the feather-edge of the MMG – but if so, as seems reasonable, why only here and not elsewhere along the boundary? In fact the springs are present here because of the coincidence of the edge of the sealing overlying MMG with the fault cutting across the boundary; the fault is even more transmissive than the unfaulted aquifer, the SSG - hence the springs.

Groundwater resources

Provision of inaccurate and misleading geology by the Applicant

The Applicant has provided a groundwater map (Appendix F figure F1) which shows the groundwater contours in the SSG. The map shows the drawdown of contours around abstraction boreholes. The map and its accompanying inset cross-section (reproduced in Figure M6a) of the path to be followed by the horizontal well are highly misleading, in that they seek to distance the Principal Aquifer, the Sherwood Sandstone Group, from the drill bore:
"As shown on Figure F1, the sub-surface works pass beneath the Source Protection Zone 3 but at considerable depth below the base of the Sherwood Sandstone Aquifer. Due to the significant thickness of strata between the sub-surface works and the aquifer there is considered to be no potential for the sub-surface works to impact on water quality in the aquifer."
The figures quoted by the Applicant in both cartoon cross-sections (Figure M6) contain gross errors. Firstly, we compare the cartoons with an annotated version of the BGS cross-section traversing the Doncaster 1:50,000 geological map. This cross-section passes the proposed development 1.8 km to the north. The annotated cross-section is shown in Figure M12. It has been vertically exaggerated by a further five times, on top of the original's x2 exaggeration, so it is now x10 vertically exaggerated. The hydrogeological implications of this cross-section are discussed below.

Secondly, I have redrawn the Applicant's cartoons (Figure M6) as a properly scaled cross-section along the line of the proposed horizontal borehole. This is shown in Figure M13. The Rocket Site borehole is projected onto the section from 55 m to the SE. The position of the Misson fault is uncertain by about ±150 m either way along the section from its marked place, but it has to lie to the NE of the Rocket Site borehole. I have put an arbitrary slight hade (inclination from the vertical) on the downthrown side, and retained a constant estimated 30 m throw for the whole depth. The actual dip and the throw may be somewhat different.

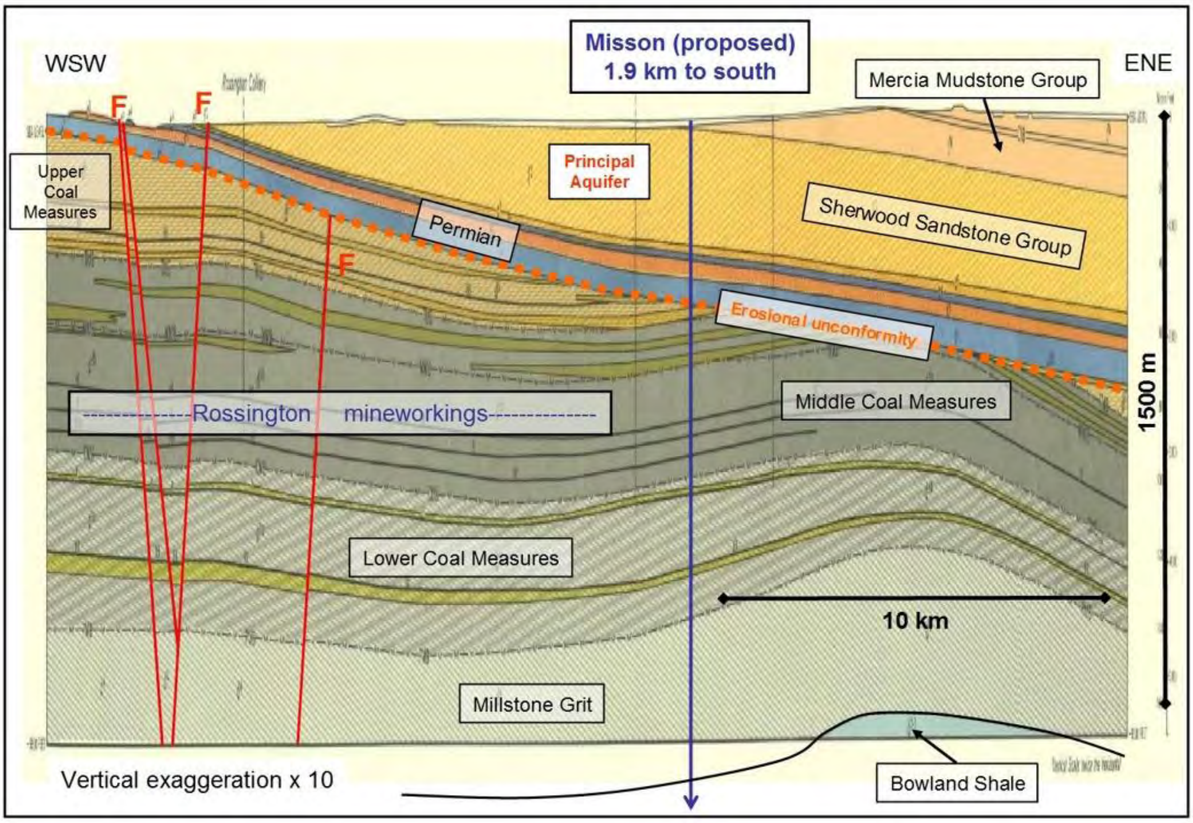

Figure M12. Cross-section from the BGS Doncaster 1:50,000 solid geology map, horizontally compressed by x5; the total horizontal compression is x10. The proposed Misson site is 1.9 km to the south of this cross-section; I have located it at the feather-edge of the MMG. The Rossington mineworkings have been extended about 8 km further east than the extent when the map was made, and more normal faults than shown here have since been found in the Coal Measures.

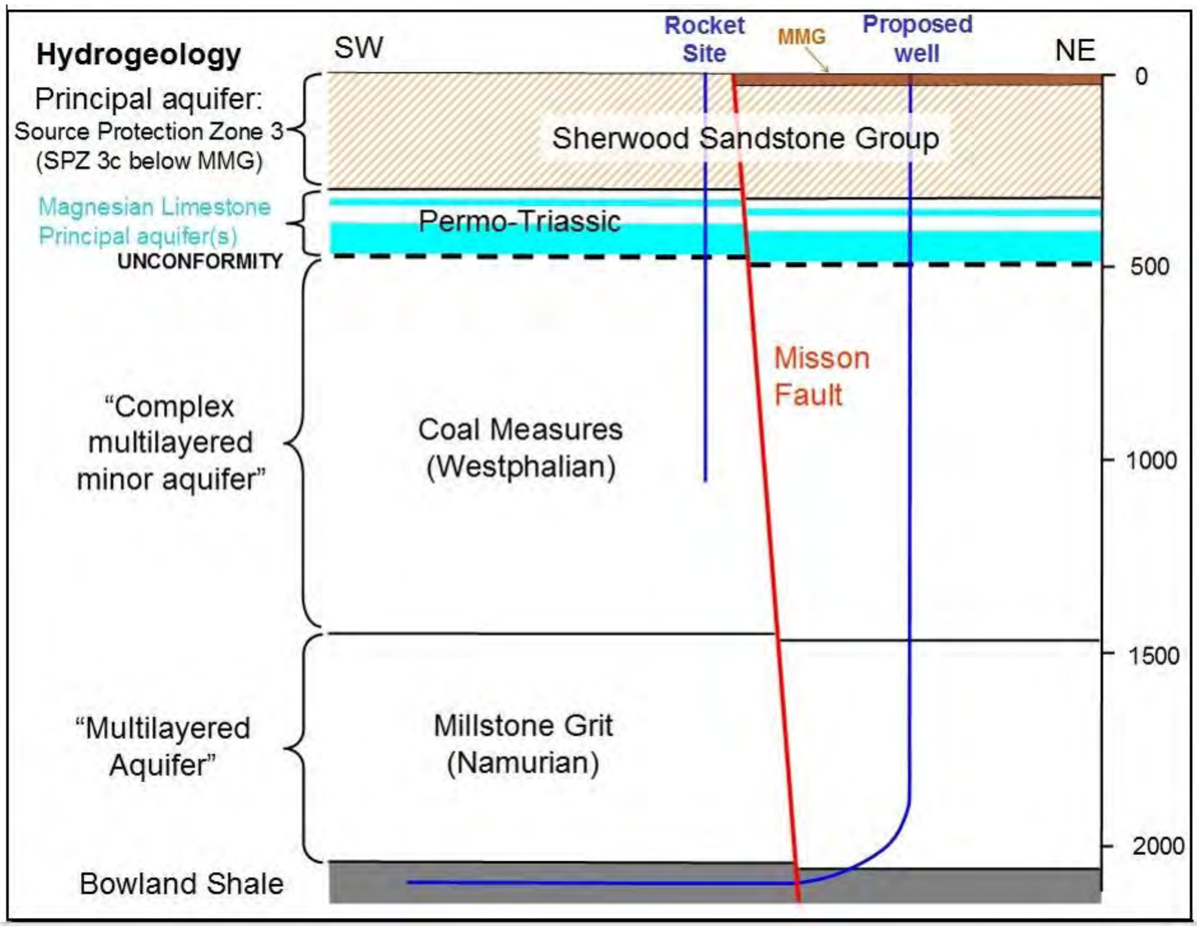

Figure M13. Properly scaled cross-section along the line of the proposed horizontal borehole, for comparison with the Applicant's cartoons (Figure 6). No vertical exaggeration. The Rocket Site borehole is projected onto the section from 55 m to the SE. The position of the Misson fault is uncertain by about ±150 m either way along the section from its marked place, but it has to lie to the NE of the Rocket Site borehole. There is an arbitrary slight hade (angle from the vertical) on the downthrown side, and I have retained a constant estimated 30 m throw for the whole depth. Summary hydrogeological information is shown on the left; the quoted classifications are from BGS authors.

Formation tops from Everton-1, some 5 km along strike to the south, and Scaftworth-B2, 6.6 km to the SW, are used to estimate the thicknesses of the Lower Coal Measures and the Millstone Grit. According to the BGS (Andrews 2013) the Bowland-Hodder Unit reaches more than 2 km in thickness (Fig. M4), of which only 234 m was penetrated at Scaftworth-B2. The Applicant's cartoons (Figure M6) lump together the Millstone Grit and the Bowland Shale into one geological unit, the thickness of the Bowland Shale being stated as about 300 m. Even if this last figure is supposed to represent net shale in the upper Bowland Unit only, the actual figure for this is 474 m at Scaftworth-B2 (Andrews 2013). Such errors are unacceptable.

Hydrogeology at the site

The hydrogeological summaries on the left-hand side of Figure M13 are taken from the BGS[3]:

"*The Millstone Grit constitutes a multilayered aquifer in which the thick, massive grit and sandstone horizons effectively act as separate aquifers with the intervening mudstones and shales acting as aquicludes or aquitards*."

[3] Jones, H. K. et al. 2000. The physical properties of minor aquifers in England and Wales. *British Geological Survey Technical Report*, WD/00/4. 234pp. Environment Agency R&D Publication 68.

“*The Coal Measures Group form* [sic] *a complex multilayered minor aquifer. Argillaceous strata predominate, acting as aquitards or aquicludes, isolating the occasional thicker sandstone horizons which effectively act as separate aquifers*

The 174 m thick Permo-Triassic sequence below the SSG comprises two Magnesian Limestone aquifers with a combined thickness of 93 m. They are separated by marls which act as aquitards. Allen et al. 1997 state[4]:

“*Further south [from the Durham area] it is divided into two: the Upper and Lower Magnesian limestones, again separated by marls and siltstones. The limestones comprise compact fractured dolomite, which is brecciated and cavernous in some areas ... There is some hydraulic continuity between the [upper and lower] units and they are generally treated as one aquifer. ... however the permeability of the whole aquifer is extremely variable as a result of the fracturing*.”

The Magnesian Limestone is classified by the EA as a Principal Aquifer[5]. As seen in Figure M13, these two aquifers lie between 338 m and 475 m depth, and will therefore contain fresh water. Since these aquifers (or effectively one aquifer, following the BGS view stated above) lie shallower than 500 m below the development site, they (or it) have to be considered as potentially at risk as well as the SSG aquifer.

The Sherwood Sandstone Group is a Principal Aquifer. It is at outcrop above about half the length of the proposed horizontal well, and covered by about 30 m of Mercia Mudstone Group to the NE of the Misson Fault.

Extent of the Sherwood Sandstone Group aquifer

The EA defines groundwater Source Protection Zones (SPZs) to show the “risk of contamination from any activities that might cause pollution in the area”. The aim is to protect “*groundwater sources such as wells, boreholes and springs used for public drinking water supply*.” (from the EA website[6]).

The proposed development lies within the total catchment, SPZ 3, defined as:
“*the area around a source within which all groundwater recharge is presumed to be discharged at the source. In confined aquifers, the source catchment may be displaced some distance from the source. For heavily exploited aquifers, the final Source Catchment Protection Zone can be defined as the whole aquifer recharge area where the ratio of groundwater abstraction to aquifer recharge (average recharge multiplied by outcrop area) is >0.75. There is still the need to define individual source protection areas to assist operators in catchment management*”.

There is a separate category, SPZ 3c, for “subsurface activity only”. (EA website[6]). But the Applicant's map (Appendix F figure F1) unaccountably omits this latter category, despite the fact that what it is seeking planning approval for undoubtedly constitutes 'subsurface activity'.

Let us be quite clear about the extent of the Sherwood Sandstone Group aquifer. It is an unconfined aquifer where it is at the surface, and confined where it lies below younger rocks. The BGS hydrogeological map of the northern East Midlands (Institute of Geological Sciences 1981) shows contours on the base of the SSG, which lies at almost precisely 300 m below MSL at the proposed development. The 500 m contour lies some 10 km to the east. This depth is

[4] Allen, D.J. *et al.* 1997. *The physical properties of major aquifers in England and Wales.* BGS technical report WD/97/34.

[5] For example the BGS web page: http://www.bgs.ac.uk/research/groundwater/shaleGas/aquifersAndShales/maps/aquifers/home.html

[6] http://apps.environment-agency.gov.uk/wiyby/37833.aspx

generally taken to be the approximate limit of potable water within the aquifer, since below that depth the water will be saline. But the top of the SSG aquifer occurs at 500 m depth an additional 10-15 km further east of the site, so technically the extent of fresh water within the aquifer extends that distance eastwards, i.e. 20-25 km to the east. It is therefore highly misleading of the Applicant to mark the limit of water to be protected as west of the site.

The BGS hydrogeological map also shows the zero height contour (relative to MSL) of the potentiometric surface of the aquifer lying some 350 m east of the development. This is the 'water table' (as of March 1978).

Allen et al. (1997) state[4], concerning the SSG aquifer:

"*In Nottinghamshire the Sherwood Sandstone Group is an important source of water for Nottingham and Mansfield. ... The aquifer is also usable to the east beneath the Mercia Mudstone outcrop to a greater degree than in the north*."

Therefore there is no question that the SSG aquifer extends under the entire development area, encompassing all the quadilateral area shown in the Applicant's site location plan where 'subsurface development' may take place. Indeed, the aquifer underlies all the PEDL licence area shown in Figure M3b.

The Applicant's statement that the "*Site is ... 300 m to the east of a Groundwater Source Protection Zone (SPZ) 3*" (Vol. 3 Environmental Statement para. 3.5.8) is wrong and misleading. The publicly available geology shows that the site lies partly in SPZ3 and partly in SPZ3c.

Angus Energy at Brockham in the Weald

A report[1] prepared by the planning development department of Surrey County Council stated:

"*Brockham Oilfield, Felton's Farm, Brockham – In September 2016, Angus Energy sought CPA agreement for them to undertake maintenance using a 15m work-over rig on one of the three wells located within the site compound. They were subsequently advised that the proposed work would be covered under existing maintenance agreements, but that the drilling of any new wells, including sidetracks, plus the testing or production from wells not already authorised, would not be permitted.*

Having been advised by Angus Energy that the presence of hydrocarbons within the X4 well construed it as being 'live' and led to them working overnight, officers relayed this information to the Health & Safety Executive, and on the basis of good practise it was considered reasonable to allow the night working for a period of one week to provide a safe environment for those working on site whilst the work-over was completed.

It subsequently transpired that a sidetrack had in fact been drilled. Angus Energy later claimed they already had permission for this. Officers did not believe that to be correct and sought Counsel's opinion, which once received, reinforced officers views. However, the operator also obtained Counsel view, which apparently upheld their belief that the drilling of the sidetrack was authorised. We have encouraged the submission of a retrospective planning application to regularise the unauthorised drilling of the sidetrack as well as the required testing of oil from the sidetrack, which Angus Energy's planning consultant has agreed would be done.

This unauthorised development has highlighted discrepancies between the legislators of the oil and gas industry, as permits for the drilling of a new sidetrack were issued by both the Environment Agency and the Oil & Gas Authority, and their legislative requirements do not require planning permission to be in place before they are issued. Whilst perfectly understandable in terms of legislation, it makes it somewhat confusing and at times misleading for both those involved and those monitoring such development."

The following timeline of the unauthorised drilling is based on that prepared by Brockham Oil Watch (BOW)[2], with inline links to BOW documentation.

2014 Angus Energy was advised by a specialist planning consultant that any works at Brockham would need a fresh planning permission (*Private Eye* No. 1466, 23 March – 5 April 2018). **(link to transcript)**

8 August 2016 Letter from Angus dated 8 August 2016 indicating the intention to drill a "side track" under the provision of existing planning consent MO08/0894. (**link**)

14 September 2016 SCC says that Angus Energy does not have planning permission to undertake drilling operations at Brockham under any of the existing permissions and that should it "*wish to carry out a 'side track' a fresh new planning application seeking planning permission will be required.*" (**link**)

28 September & 24 November 2016 Meetings between SCC and Angus. On 28 September Angus say it wishes to do a workover programme, and on 24 November that it wants "*the workover rig to be placed on Brockham-1 to mirror that borehole to the same depth and*

[1] Surrey County Council 2018. Enforcement and Monitoring Update Report. Item 8 to Planning & Regulatory Committee from Planning Development Control Team Manager, 21 March 2018.

[2] https://brockhamoilwatch.org/timeline-drilling-of-brx4z/

log the data." There is no mention of drilling in records from either of the meetings. (link **here** and **here**)

2, 9 & 9 December 2016 Email from from Mark G. Oldridge on behalf of Angus Energy to SCC describing the work to be done as "*abandon several open hole sidetracks*" and "*to mirror the existing well to gather geological information that was not acquired when originally drilled. No formation deeper than the original wellbore is to be evaluated.*" .Emails on 6 and 9 December clarified that works would be undertaken at Brockham Number 2 well. (**link**)

12 December 2016 SCC confirmed that Angus Energy could carry out maintenance activities on Brockham No.2 (as set out by Angus in email on 2 December) under an existing planning permission (later clarified to residents as MO 06/1294), and that if Angus wished to drill any new boreholes or sidetracks, then fresh planning permission would be required. (**link**)

13 December 2016 Jonathan Tidswell, CEO of Angus Energy, explained in an email to SCC that Angus Energy plans to "*re-enter the original Brockham well that BP drilled in 1987 down to the great Oolite formation (...) to geologically assess these formations before running liner and cementing in place.*" *[note: the original wellbore that went down to the Oolite formation is Brockham-1]* (**link**)

16 January 2016 SCC visit to the well site. Workover rig was installed. A relevant note from the CPA to a local resident said: "*When the three County Planning Authority (CPA) Officers visited the site on Monday 16th January 2017, there was nothing out of the ordinary at the surface that indicated a new sidetrack well was about to be drilled. The equipment in place on site was to be expected as part of a work-over, and the drilling took place from an existing well head.*" (**link to site visit notes**)

17 January 2016 Email from Jonathan Tidswell (CEO of Angus Energy) that the day before at 16:00 they opened the well and "*found the well to be live and decided to run in hole a kill/cementing string straight away and displace the well to heavy fluid.*" They anticipated to have completed this process within seven days. This issue was passed onto the HSE, which allowed work to continue on safety grounds. (**link to J Tidswell email and to correspondence between SCC and HSE**)

18 January 2017 Angus reported in an RNS announcement that the well intervention on Brockham-1 was underway and that re-entry was expected to be completed within the week. (**link**)

26 January 2017 Angus reported in an RNS announcement that "*further to the 18 January 2017 announcement, work to complete, log, case and cement the well at the Brockham oilfield has now been successfully completed*" and that the well would, upon OGA confirmation, be renamed BR-X4Z. (**link**) But OGA later confirmed in response to an FOI request (reference FOI-2017-0019) that it did not have any documents relating to the renaming of Brockham-X1 well to BR-X4Z. (**link**)

26 January 2017 During a joint site visit from the EA, HSE and SCC, Angus confirmed that it had drilled a sidetrack from BRX4. (**link**)

15 February 2017 Meeting between Angus Energy and SCC including legal representatives on both sides to discuss the unauthorized drilling and well numbering confusion. SCC asked Angus to regularise the situation and said that Angus must address retrospective planning application for the sidetrack. (**link**)

8 March 2017 Meeting between Angus Energy and SCC including legal representatives on both sides following Angus announcing the drilling results via RNS on 3 March 2017 and the story breaking in the news. SCC says that there was no planning permission to produce from the unauthorised sidetrack either. (**link**)

3 April 2017 Letter from Angus Energy's legal representative setting out their position and explanation as to why sidetrack BRX4Z was considered to be authorised. (**link**)

5 April 2017 Letter from SCC to Angus again restating SCC's position that the sidetrack and production from it was unauthorised, and that the Planning Authority is also seeking Counsel's advice. **(link)**

20 April 2017 Site visit report highlighted unauthorised drilling and the need for retrospective planning in "Actions and information for the operator." **(link)**

28 April 2017 Angus's legal representative restated the operator's position and threatened legal action. The letter demanded "*that the County Council will not issue any further public statements or other documents which wrongly assert that the current Brockham operation is unlawful, failing which Angus Energy will take* ***whatever legal steps are necessary in order to protect its position***" **(link)**

28 April 2017 Letter from SCC's Principal Lawyer that SCC did not accept Angus's position and that the County Council is seeking advice from Queen's Counsel. **(link)**

21 July 2018 An email from SCC to Angus Energy restated the Planning Authority's position in response to Angus's 'Investor call' on 12 July 2017, where it was stated that production from both of the production wells at Brockham would commence in the summer of 2017. **(link)**

19 October 2017 Letter from SCC to Angus confirmed the planning authority's position following the receipt of Counsel's advice with respect to drilling of BRX4Z and production from it. The letter also highlights that BRX4 (the mother well to sidetrack BRX4Z) "*is now in breach of planning control with an outstanding requirement to close the well and restore the land*". **(link)**

20 December 2017 Angus submitted retrospective planning application for BRX4Z. The initial application was deemed invalid and needed to be amended before it was published online on **6 March 2018**. It was for a retrospective permission for drilling BRX4Z and retention of the parent borehole, BRX4, for which consent had expired in 2008, as well as for appraisal of BRX4Z (changed from **'production evaluation'** in the initial screening opinion request related to this application). (links **here**, **here** and **here**).

7 March 2018 In its **annual report**, Angus restated a claim that it had made to investors on numerous occasions, i.e. that it would achieve "*the first* ***commercial production*** *from the Kimmeridge layers at Brockham (Brockham-X4Z) in 2018.*"

Angus also maintained that sidetrack BRX4Z was fully authorised, having obtained its own Councel opinion: "*The Queen's Counsel has confirmed her considered view that well BR-X4 (the donor well of the X4Z sidetrack, also known as well no. 3) has planning permission until 2036. Similarly, the QC confirms that* ***the sidetrack to Well BR-X4, drilled in January 2017, is authorised by the 2006 planning permission***". This was also stated on its website. **(link to annual report).**

8 August 2018 After delaying deliberation on this application three times (apparently due to insufficient information provided by the operator), the SCC's Planning and Regulatory Committee granted permission, without imposing any meaningful conditions in response to the main objections about lack of up-to-date environmental permit, ongoing investigation into seismic activity, and operator competence (**SCC Ref 2017-0215_planning_Decision Notice)**

Link to Applications on SCC Planning Register:

The retention of the BRX4 well, the regularisation of the BRX4Z sidetrack, and the appraisal of BRX4Z using production plant and equipment within the existing site, for a temporary period of three years (part retrospective).

- Details of a Scheme of work in respect of a workover rig pursuant to Condition 8 of planning permission ref: MO/2018/0444 dated 15 August 2018.
- Details of linear rod pump pursuant to Condition 7 of planning permission ref: MO/2018/0444 dated 15 August 2018

UKOG at Broadford Bridge

Planning permission from West Sussex County Council (WSCC)

UKOG has consistently claimed that it had all necessary permissions to drill Broadford Bridge-1:

"*namely from the Oil & Gas Authority (OGA), the Environment Agency (EA), the Health & Safety Executive (HSE) and West Sussex County Council (WSCC). Despite false and defamatory claims from the pressure group called Keep Billingshurst Frack Free (KBFF), all permissions are in place. For reasons of transparency, the documents demonstrating consents are available on our website (www.ukogplc.com).*"[1]

But in its Broadford Bridge web page[2] the links to the necessary permissions exclude the planning consent from WSCC, the MPA. This omission appears to be because the permit was one inherited from Celtique Energie, which obtained the permit[3] on 11 February 2013. The permit states explicitly that the well is for:

"*the exploration, testing and evaluation of hydrocarbons in the willow prospect*"

The approved operations programme states that:

"*The development hereby approved shall be carried out in accordance with the particulars of the development contained in the application and plans and attached to planning application WSCC/052/12/12 (Environmental Statement dated July 2012, drawing numbers 3261/BB/02 Revision A, 3261/BB/03, 3261/BB/07 Revision B, 3261/BB/08 Revision B, 3261/BB/09 Revision A, 3261/BB/10 Revision A, 3261/BB/ll Revision A, 3261/BB/12 Revision A, 3261/BB/13, 3261/BB/15 Revision A, 3261/BB/17, 3261/BB/18 and the Grassland Management Plan (date stamped 15 Jan 2013). except as modified by condition hereafter.*

Reason : To ensure the development is carried out as proposed"

The Environmental Statement submitted by Celtique in July 2012 staes explicitly that the development was for the "exploration, testing and evaluation of hydrocarbons in the Willow Prospect". Further,:

"To enable the exploration of the Willow Prospect, the Applicant has identified a "bottom hole target" (i.e. the calculated depth to which the borehole will drill that will hopefully lead into the "target" oil or gas reservoir). The target is located approximately 800m to the north of the Application Site and the Applicant proposes to use deviated drilling from the Application Site to the bottom hole target."

A structure contour map of the Willow prospect was provided[4]. This prospect was a sandstone fault-bounded structure of Triassic age (Sherwood Sandstone Formation). There is no mention in the Celtique environmental statement of the Kimmeridge Clay Formation (KCF). There are no documents on the WSCC planning website to indicate any subsequent modification to the approved 'Willow' target.

Therefore in drilling at a much shallower angle to test the KCF UKOG breached the MPA permit. No sanctions have subsequently been applied.

[1] Letter from Steven Sanderson to Nick Herbert, 28 May 2017

[2] https://www.ukogplc.com/page.php?pID=128

[3] https://westsussex.planning-register.co.uk/Planning/Display/WSCC/052/12/WC

[4] https://westsussex.planning-register.co.uk/Document/Download?module=PLA&recordNumber=1383&planId=14393&imageId=70&isPlan=False&fileName=ES%20Figures%20-%20Volume%202%20-%20Chapter%205%20-%20Need%20and%20Alternative%20Sites.pdf

Micrites misleadingly labelled as limestones

[extract from consultation submission[5]]

UKOG's production plans are targeting two so-called 'limestones' within the Kimmeridge Clay Formation, labelled KL #3 and KL #4, respectively. These layers are actually micrites, or calcareous mudstones. They are very far from being true limestones. Each of these is 30 m thick. Some of the wells appear be planned to traverse some of these micrites at very oblique angles. The only means of tracking the lithology that the bit is drilling through is by upward- and downward-pointing gamma ray sensors installed just behind the drill bit. These sensors are supposed to detect whether the bit is within a calcareous layer (low gamma readings) or within shale (high readings). The gradual approach of the bit to the roof or to the floor of a micrite layer being drilled horizontally is detected by a rise in gamma level. But if the bit passes through a small undetected fault and then proceeds through shale, there is no way of detecting whether the bit should be directed upwards or downwards to regain the micrite. The target layer is lost. The 30 m thickness of the two main micrites is at the limit of the vertical resolution of the 2D seismic method, even if there were seismic control at the location of the bit. Thus a fault with a 30 m (or greater) throw could displace the micrite completely away from the track of the horizontally-progressing drill bit.

There are no magic markers in the subsurface which tell the driller 'This is KL no. 3', for example. There are just several thin layers of shale which have a slightly greater proportion of calcium carbonate than normal shale. Therefore the risk of undertaking the proposed drilling is that the target layers may be missed, may disappear abruptly, and/or the wrong layer may be drilled. In consequence, the geology may then be re-interpreted wrongly, as I have demonstrated to be the case with UKOG's drilling fiasco at Broadford Bridge. In brief, UKOG firstly breached the conditions laid down in the permit issued by West Sussex County Council, The operator drilled, not to the explicitly defined permitted Triassic conventional target nearly vertically below the well pad, but at a shallow angle and at a very different azimuth to target the Kimmeridge micrites (Figure 4.1).

It ran into borehole washout problems by foolishly trying to traverse the fault zone where the Purbeck Limestones are cut by the fault. It then drilled a sidetrack well BB-1z. My reconstruction of the geology in the two parallel wells (Fig. 4.2) accurately fixes the tops and bottoms the the Kimmeridge Clay Formation. I have interpolated the expected depths of the four micritic layers by scaling the nearby Wineham-1 well log to fit, as a proxy. My explanation for the UKOG discovery of a supposedly new fifth and uppermost micrite is that the sidetrack well drilled micrite KL4 twice, once on either side of the fault. I have marked the fault as vertical, but applying a dip on it to the north or to the south makes no difference to the explanation.

In conclusion, the exploration of Broadford Bridge by UKOG was:

- Unpermitted,
- A technical fiasco, and
- Led to claims of a supposed new micrite layer.

Lastly, it should be noted that the BGS, in its Weald study of 65 wells, only recognises a maximum of three Kimmeridgian micrites in the Weald Basin; in UKOG terminology these are numbers 3 and 4, with a locally recognised number 2. The micrites depicted in the application documents by UKOG labelled 0, 1, and 5 have never been documented by any non-UKOG earth scientists. UKOG's understanding of the geology falls woefully short of the professional standards of the hydrocarbon industry.

[5] Smythe, D. K. 2019. *Geological objections to Surrey County Council planning application no. 2018/0152 by UK Oil & Gas for hydrocarbon developments at Horse Hill*. 21 June 2019, 19 pp.

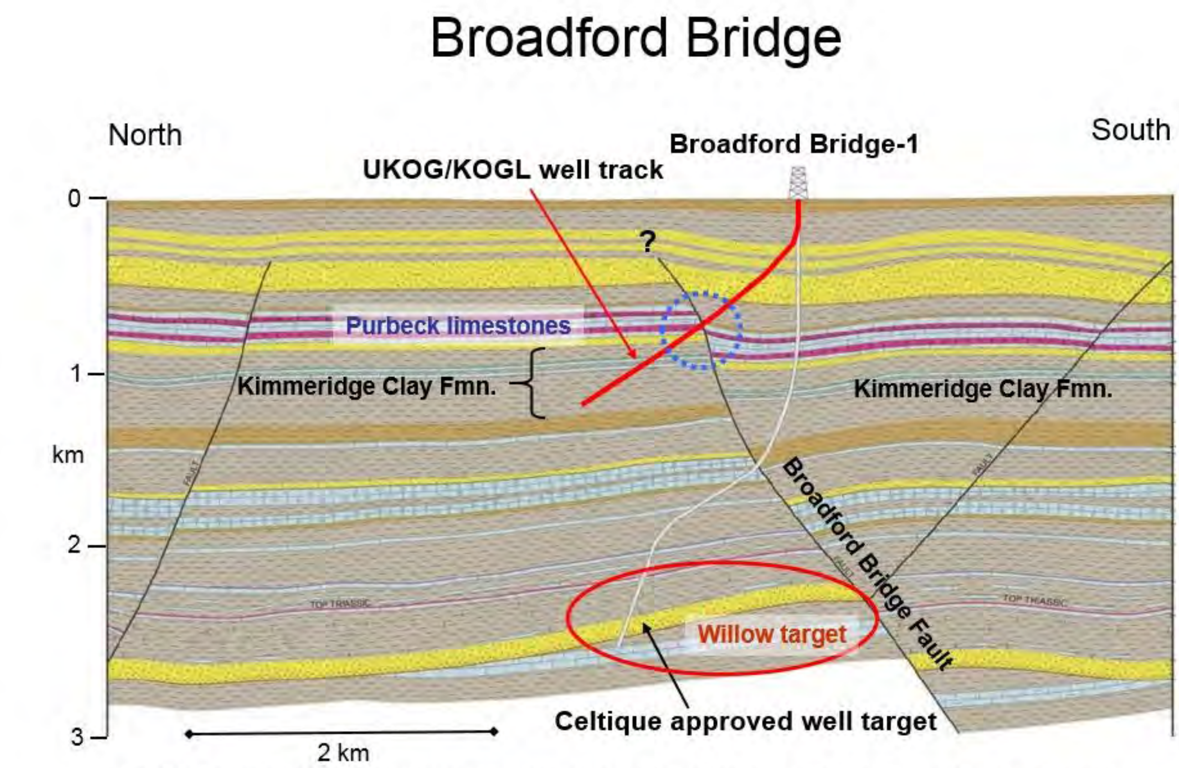

Fig. 4.1 UKOG subsidiary KOGL illegally flouted the permit conditions, drilling a very steeply inclined well at a different azimuth to test the Kimmeridge Clay Formation and not the permitted 'Willow' conventional target. In doing so it encountered borehole washout problems because they were crossing the Purbeck Limestones at a fault zone. KOGL then had to sidetrack. The next diagram (Fig. 4.2) shows a reconstruction of the main well and the sidetrack.

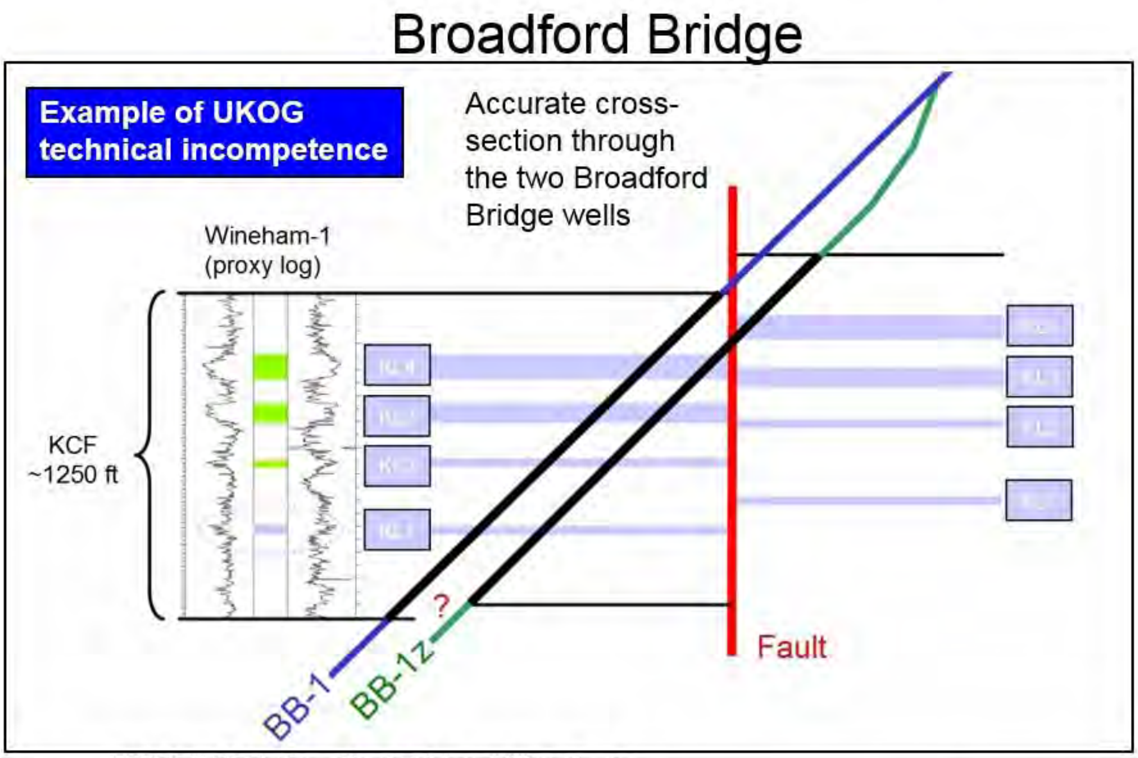

UKOG / KOGL Broadford Bridge drilling fiasco

Fig. 4.2 I have interpolated the tops for the micrites by using nearby Wineham-1 as a proxy, scaled to the Kimmeridge Clay Formation thickness here. The light green micrites are as recognised by the BGS. KL1 is dubious; My explanation of the fifth 'limestone' seen by KOGL is that KL4 has been penetrated twice by going through a fault, so that UKOG's KL5 = KL4.

UKOG at Horse Hill

[extract from consultation submission[1]]

Executive summary

The 1980s 2D seismic database used by UKOG was adequate for mapping the simple hydrocarbon prospects of that era, which were then tested by vertical exploration wells, but is inadequate for drilling slant or horizontal wells away from the seismic control. No new seismic data have been obtained, either 2D or 3D. Problems arising from this inadequacy are illustrated by the history of UKOG drilling at Broadford Bridge.

The UKOG interpretation of the Horse Hill geology has several internal contradictions, geologically unlikely geometry, and/or mapping errors:

- The location of the Collendean Farm-1 (CF-1) well used by UKOG is 150 m NW of its true position.
- It is placed on the wrong (northerly) side of a large fault, the Collendean Farm Fault.
- UKOG's attempt to explain the resulting mismatch of well tops (geological layers) appeals to a highly unlikely shallow velocity anomaly.
- The UKOG interpretation of a single fault with a sharp bend and changing sense of throw is geometrically unlikely.
- Misleading diagrams have been employed to try to show that the deviated Horse Hill-1 (HH-1) well has not been drilled in the vicinity of faults.
- In contrast, I have re-interpreted the geology to resolve these fundamental problems:
- There are two separate *en echelon* faults in the vicinity of HH-1 and CF-1, the Collendean Farm and the Horse Hill Faults.
- An accurate and simple well tie from CF-1 to HH-1 can be made without errors or recourse to velocity anomalies.
- HH-1 has been drilled into the HH Fault damage zone, and possibly intersects the fault itself. This accounts for the temporary high, but rapidly declining, Kimmeridge oil flow.

It is not clear whether UKOG has revised its geology in the light of my previous published critiques (a non-technical blog and a technical analysis). Some of the planning application diagrams imply two faults, with CF-1 now on the correct (upthrown, southerly) side of the Collendean Farm Fault, but other submission documents stick to the erroneous interpretation. UKOG's own submissions are inconsistent on this matter.

The 3D cartoon perspective views of actual and proposed drilling submitted by UKOG are inadequate for a proper scrutiny of the planning application. The application should be supported by internally consistent structure contour maps of the several horizons which it is proposed to develop, together with illustrative properly-scaled cross-sections. No such maps and cross-sections have been submitted.

Several of the proposed wells, to be deviated due south, stray outside the defined development deviation area.

The supposed target 'limestones' are in fact micrites, which are calcareous mudstone layers. UKOG purports to recognise six such layers, but the BGS recognises only three micrites in the Weald Basin. One of the additional layers is an existing layer repeated by faulting, but mis-diagnosed by UKOG as a new layer. UKOG proposes only an acid wash (5% acetic acid) for the target micrites, but 15% hydrochloric acid is stored on site. It is not clear how UKOG will be able to stimulate flow within the Kimmeridge target shales and micrites without matrix acidisation or fracking. Its appeal to 'natural fracturing' of the micrites as a flow mechanism is based upon the results from drilling HH-1 into a fault zone, and therefore cannot be extrapolated more widely.

[1] Smythe, D. K. 2019. *Geological objections to Surrey County Council planning application no. 2018/0152 by UK Oil & Gas for hydrocarbon developments at Horse Hill*. 21 June 2019, 19 pp.

UKOG misleadingly cites historical earthquakes from outside the Weald Basin in support of its view that the Newdigate swarm of 2018-19 is not unusual. My analysis shows that earthquakes above local magnitude 2.0, had they occurred within the last few hundred years, would have been documented, but records of these do not exist; therefore the recent swarm is highly unusual. UKOG also supplies diagrams which misleadingly seek to minimise any link from faulting and well operations at Horse Hill to the Newdigate swarm.

The applicant should be required to undertake a 3D seismic survey of the area of interest and submit these data, properly interpreted, before permission is granted for such a development.

Because of the severe internal errors and inconsistencies and unacceptably poor standard of submission of the geology and proposed development drilling, documented herein, the current application should be refused.

UKOG inconsistencies in fault intepretation

[extract from section 3]

Detail in UKOG's proposals is woefully inadequate. Consider firstly an example 'well plat' from Pennsylvania (Figure 3.5).

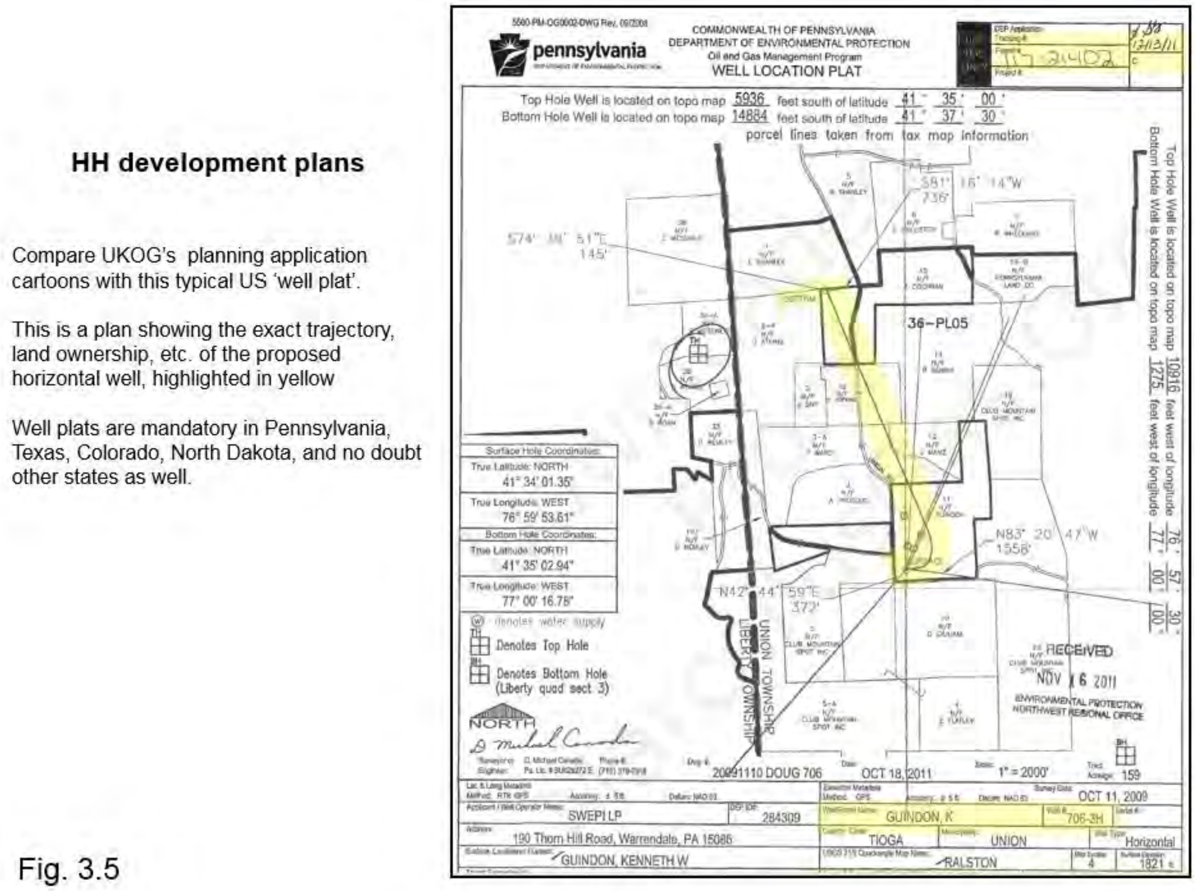

Fig. 3.5

This is a detailed surveyor's plan for just one well, highlighted in yellow, and is a requirement before a drilling permit is issued. It shows the precision required before permission to drill is granted. Furthermore, this is in an area where the geology (the Marcellus Shale) is particularly simple. In contrast, Weald geology is heavily faulted and folded. Similar requirements for mapping prior to drilling exist in the other US states where unconventional drilling is taking place.

Instead of submitting the necessary detailed structural geology maps, UKOG sees fit to supply merely a couple of cartoon perspective views of the geology and the proposed development drilling. Figure 3.6 shows the first of these.

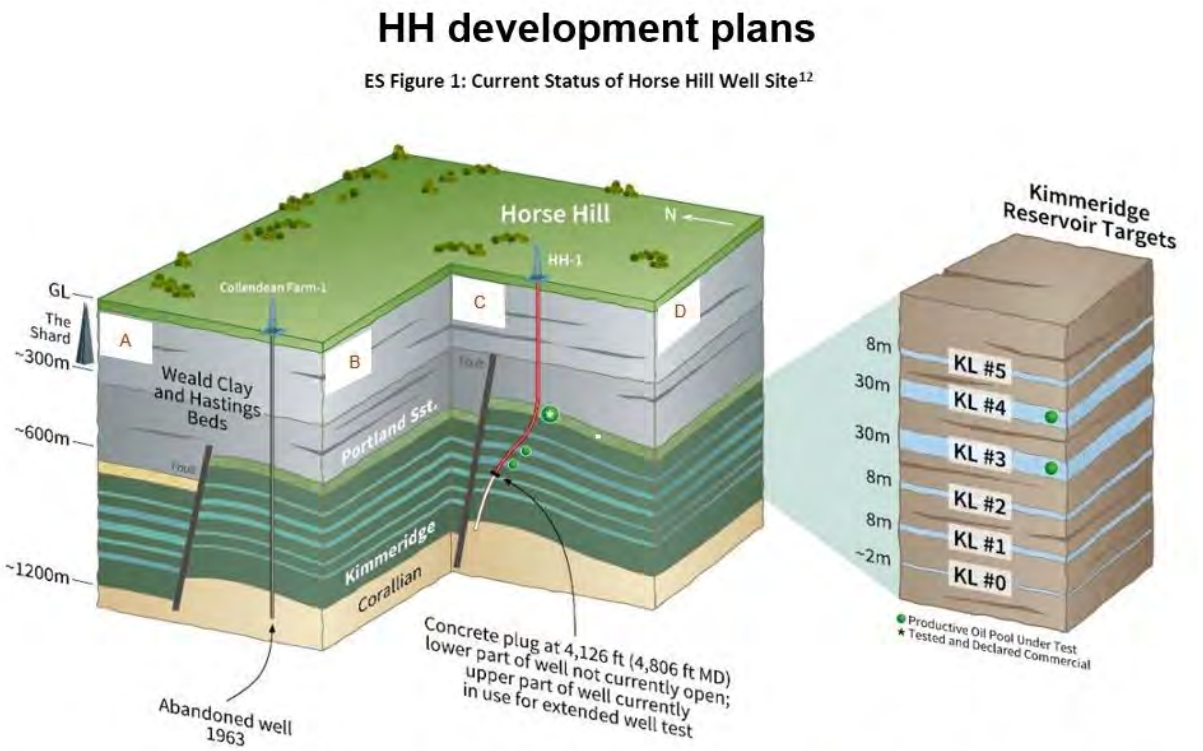

This cartoon implies two separate faults, because if there were only one fault it should intersect the south-facing vertical section B.

Fig. 3.6

It purports to be a perspective or isometric view of the geology, with the two existing wells shown in the N-S vertical faces here labelled A and C respectively. East-west face B shows no fault. Referring to Figure 3.7, this absence implies that there are two separate faults, conforming to my Collendean Farm and Horse Hill Faults, respectively. If the UKOG interpretation of the Top Portland Sandstone shown in Figure 3.3 is correct, there should be the single 'Horse Hill Fault' cutting face B.

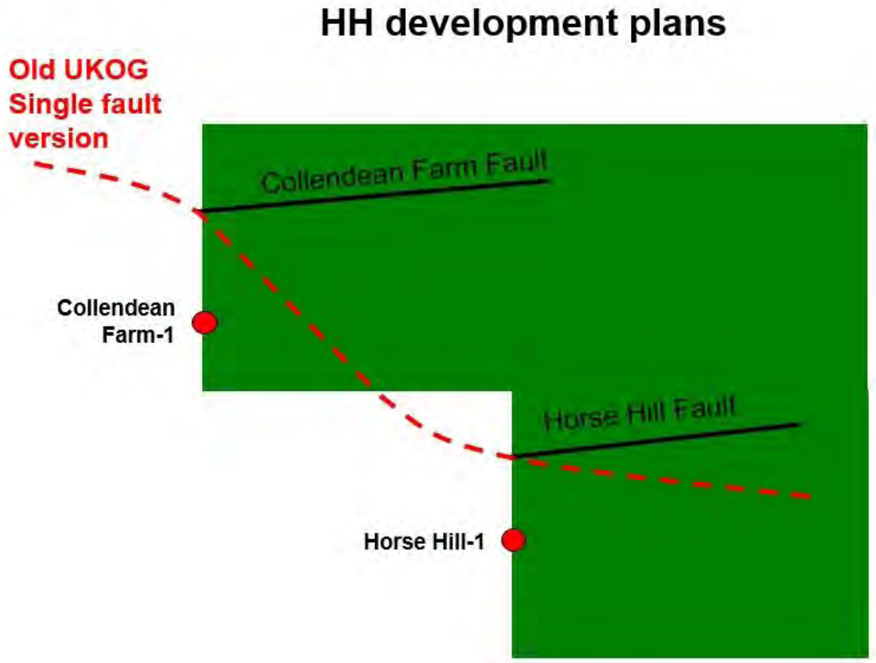

Plan view of Figure 3.6, demonstrating that the previous UKOG single fault version is no longer current. Note also that CF-1 is now on the south side of the Collendean Farm Fault.

Fig. 3.7

Note that both the faults in the cartoon of Figure 3.6 displace the Top Portland down to the north. So UKOG's maps and cartoons are internally inconsistent.

The location of CF-1 in relation to the major nearby fault is also internally inconsistent. In Figure 3.6 the well lies to the *south* of the major fault, but in the even more recent structural map of the deeper Great Oolite horizon (Fig. 2.1) the well is positioned by UKOG to the *north* of the major fault. If the correct location for CF-1 is taken into account, the vertical well intersects the fault at Top Great Oolite level.

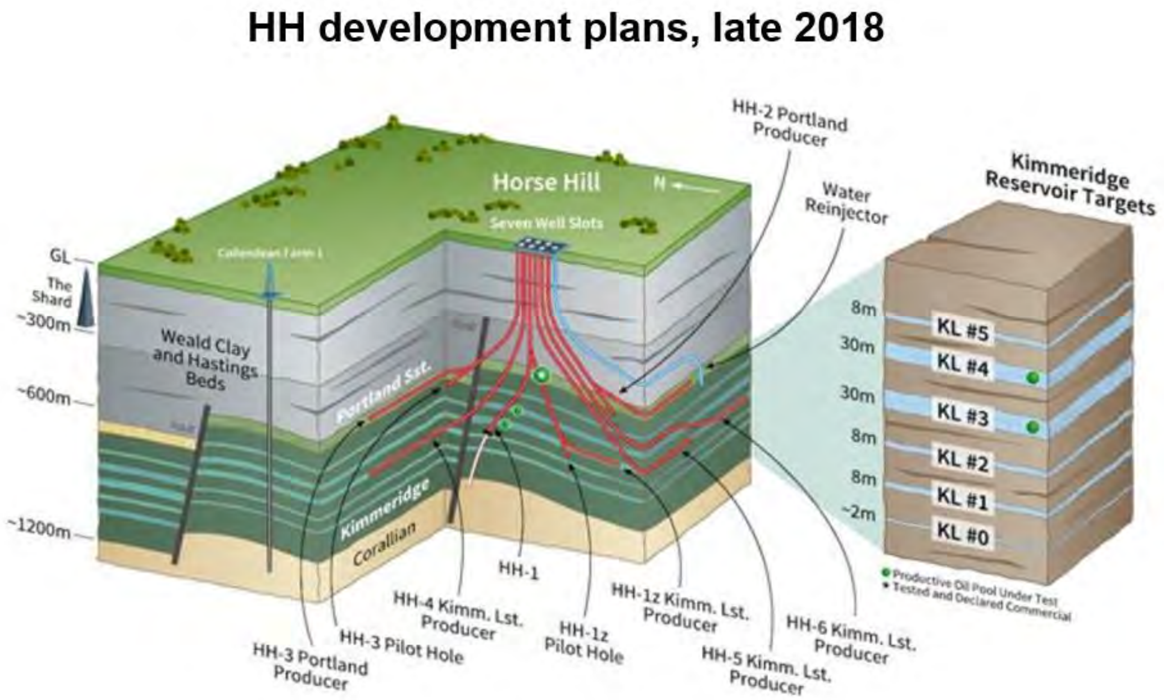

ES Figure 5: Proposed Development at Horse Hill Well Site[12]

This cartoon grossly is insufficient in detail for a serious production planning application. But the required detail has not been submitted.

Fig. 3.8

The second cartoon submitted by UKOG shows the proposed development (Figure 3.8). Note that the vertical faces of the 3D perspective view are aligned E-W or N-S. So the wells depicted on these faces deviate in the same two directions. But the more easterly of the two subsurface deviation sectors specified by UKOG (Figure 3.9) does not allow for wells from the wellpad to deviate initially southward, viz. HH-1z, HH-1z "producer" (which should be labelled more correctly as HH-1y), HH-5 , HH-6, HH-2, and the "Water re-injector" well.

Comments on development plans

- Plans submitted are mere sketches.
- Wells have inadequate seismic (structural) control.
- Geology assumed flat, no faults.
- Wells deviating south go outside specified deviation sector.
- 3D seismic volume needed before drilling permitted.
- Geology needs to be re-interpreted from scratch.
- Learn from BB-1 fiasco.
- UKOG now claims neither fracking nor acidisation needed.

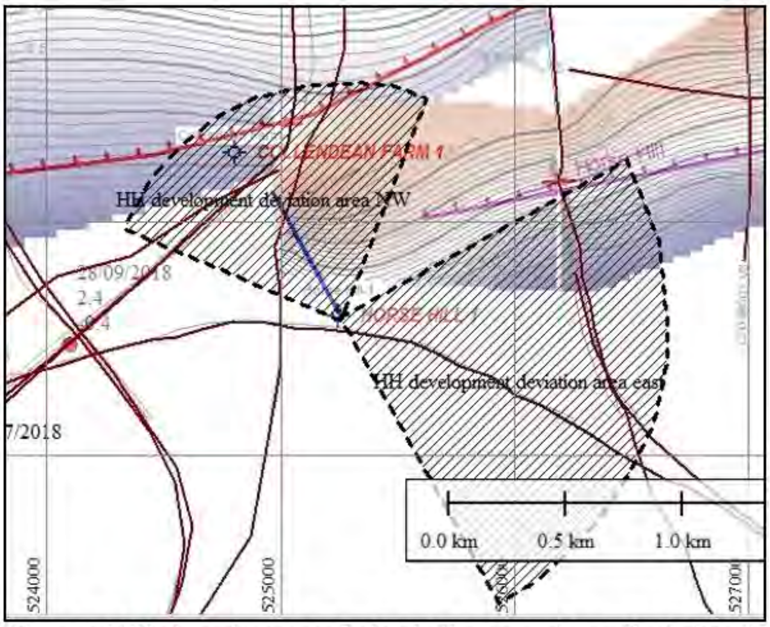

Horse Hill development deviation sectors (hatched).
2D seismic lines – brown.
HH-1 – blue line.
Collendean Farm Fault at c. Top Portland – red toothed line.
Horse Hill Fault at c. Top Portland – mauve toothed line.
Contours on fault surfaces blue (shallow) to red (deep).

Fig. 3.9

The geology in the two cartoons is depicted as essentially flat, disturbed only by the one (or is it two?) faults at depth. Such an assumption is simplistic, and too optimistic for 'blind' drilling. The lack of adequate seismic data, especially in the easterly deviation sector, implies that these wells will be drilled up to several hundred metres from any seismic control.

Lancashire 2009-2012

An application to drill at Hale Hall near Kirkham was prepared by Cuadrilla in late 2009, but the site was never drilled. The application[1] states:

"*The Bowland Shale gas reservoirs in these wells are described as "unconventional' reservoirs. In such reservoirs the source, reservoir and seal rocks are all in the same formation and methane gas in widely distributed as adsorbed and free gas throughout the formation in relatively low concentrations. Subsurface stimulation is needed to help flow the gas to surface.*"

Preese Hall-1 was drilled in 2010. The exploration and earthquake investigations were undertaken in the following order:

- Reprocessing of 2D seismic reflection lines (1980-83 vintage).
- Geological interpretation.
- Drilling of the vertical appraisal well Preese Hall-1 (2010-2011).
- Fracking stages 1 and 2 carried out (28-31 Mar 2011).
- First events triggered (30 Mar 2011 – 5 Apr 2011), incl. $M_L = 2.3$ (1 Apr 2011).
- Wellbore deformation identified (4 Apr 2011).
- Installation of two local seismometers from Keele University (7 Apr 2011).
- Fracking stage 3 carried out (8 Apr 2011).
- Installation of two extra seismometers (11 Apr 2011).
- Removal of two Keele seismometers (20 and 28 Apr 2011).
- Fracking stages 4 and 5 carried out (26-28 May 2011); more events induced, incl. $M_L = 2.3$ (27 May 2011).
- Well operations suspended; de Pater and Baisch report commissioned (June 2011).
- De Pater and Baisch report (2011) completed (2 November 2011).
- Acquisition of the 3D seismic reflection survey (March – June 2012).
- 3D seismic data processing (July – October 2012).
- Geological re-interpretation.
- Abandonment of site (December 2013).

Grange Hill-1/1z was drilled in 2011. According to a later planning application[2]:

"*Drilling activity commenced on 15th January 2011 and was completed by the 13th May 2011. The drilling operations overran slightly due to the hard formations encountered. It is noted that no complaints were received during the drilling period or as a result of the mobilisation/ demobilisation of the drilling rig.*

Well testing operations at Grange Hill were not carried out due a moratorium on fracturing imposed by the Department of the Environment and Climate Change (DECC). Operations ceased at Grange Hill in May 2011 and the site has been retained, with a suspended well, awaiting further announcements from DECC."

[1] Cuadrilla Resources Limited 2009. Planning application for Hale Hall Exploration site. Section 2. Supporting statement, November 2009. Lancs County Council no. 5/09/0813.

[2] Cuadrilla Bowland Ltd 2014. Grange Hill. Retention of Site Compound, Pressure and Seismic Monitoring, Plug & Abandon Existing Well and Site Restoration.Planning Statement 22 May 2014.

This statement appears to contradict the record in the composite well log, which agrees with the start date of 15 January, but states that drilling was only completed on 19 July 2011. There then followed three days of logging.

DECC stated, in a letter[3] to Trina Froud:

"*Grange Hill 1 was drilled to a total measured depth of 6478 ft (6400 feet true vertical depth) and after cementing the open hole back to the 7" casing the operator re-entered the well to drill through the cement at the bottom of the borehole and had a mechanical sidetrack when the bit sidetracked out of the old hole at a measured depth of 5800ft (5570 feet true vertical depth). Grange Hill 1Z was drilled from this point to a total depth of 10775 ft measured depth (10709 feet true vertical depth).*"

An email from Cuadrilla to the HSE dated 21 March 2011 described the problem:

"The original plan was to run 13-3/8" surface casing, 9-5/8" intermediate casing, and 5-1/2" production casing. But we have been experiencing serious hote problems that have already caused us to be struck and loose [sic] part of our bottomhole assembly, which took 17 days to fish. After retrieving the fish we conditioned the hole and started to drill again, but are experiencing the same problem of having large rounded pieces of shale (some up to 3" diameter, and 1 inch thick), that fall in and stick our drill string. So we have to change our program to prevent getting stuck again.

Our plan is to run 7 inch casing from about 6000 ft. back to surface, and cement it up inside the 9-5/8 casing. We think the hole problems are coming from washouts up in the Kinderscout 5hale at around 4200 to 4800 ft (note: we ran a calliper log previously over that section when we first started having problems). We will then drill ahead to TD (est. 9500 ft.) using a 6.25 inch bit, and will finish off the hole with a 4-1/2" liner."

The Old Bankfield no. 1 borehole was drilled in 1947, 1060 m NW of Grange Hill-1/-1z. It encountered the top SSG at 62 m below sea level (see Figure 6). Therefore very shallow or non-existent MMG could have been predicted at Grange Hill. The EA made no comment about the risk to the SSG primary aquifer of having such shallow predicted cover.

[3] DECC letter, 7 March 2016.

Correspondence between the EA and a local resident, 3 October 2014

From: ***@environment-agency.gov.uk [text in black]
Sent: Friday, 3 October 2014 08:32
To: 'm*********@*******' [text in red]

[NB Mike Hill was writing to the EA in a personal capacity as a local resident of Lytham St Annes. The name of his correspondent, the EA manager for Lancashire, has been redacted]

Thanks Mike

I think I may have confused you here Mike. The Environment Agency have not required groundwater permits for the drilling of the PH1.

I did not say anything about groundwater permits ***. I think you have got yourself a bit mixed up here.

The seismic activity caused a deformation of the borehole close to the base of the well around 8500ft below the ground and the independent report that Cuadrilla commissioned for the Department of Energy and Climate Change (DEC) 'Geomechanical study of Bowland Shale Seismicity' dated November 2011.

***: Mark [Mark Miller, of Cuadrilla] commissioned this and asked me where I thought it should be done, How could he ensure it was independent and would I review it!

The location, at greater depth than the higher perforated and hydraulically fractured zones and degree of the deformation makes it clear that the well bore integrity was not compromised.

Here I have to stop you. I am afraid you are not correct. On what basis do you make this statement ***? Do you know about calipers? Have you seen one run? Do you know about casings? I know that you do not *** so please do not comment on these things in that manner because you are giving the (wrong) impression that you know all about it and well integrity could not have been compromised. This is simply not true. Just because the ovalisation occurred in the production zone does not (I am afraid) mean that the intermediate and surface areas are in the clear. I informed the DECC of this fact. A USIT was required but never ran. Styles admitted this too based on engineering advice. A CBL of the 9 5/8 was also needed at the time of construction but the HSE rejected the idea in forceful language to me (only to ask Cuadrilla for it three years later when it was discovered the well had an integrity problem (Please see correspondence to HSE from HILL on this matter which I have taken up with them and asked for an explanation of their actions – as they simply beggar belief). The degree of deformation was severe over a considerable interval so odd you would think this points to being a positive. It doesn't. *** as I stated above Mark gave me that report to review before it was issued. Have you read it even now? The fact that you are in theory the person responsible at the EA when Cuadrilla frack again is giving me great cause for concern.

The Environment Agency reviewed the impact of the seismic activity on the well at Preese Hall and did not consider that there was a heightened risk of pollution to groundwater. We are satisfied that the assessment carried out to determine whether the activity required a permit to protect groundwater under the Environmental Permitting (England and Wales) Regulations 2010 remains valid. The environment Agency assessment concluded that there was no requirement to permit the hydraulic fracturing operations requirements of the Groundwater Directive 2006/118. *Provided that the well is closed and decommissioned in*

line with the requirements of the Health and Safety Executive there is no need to review our risk assessment in relation to groundwater bearing strata in the vicinity.

Ok this line above in italics is of concern. It is your responsibility to monitor that well for 30 years not the HSE and not Cuadrilla. You! You cannot assume that the well has been abandoned in line with requirements of the HSE. You have to go out there and monitor it! Lancs CC have told me they are already "assuming" you are doing your job effectively and on that basis have awarded planning permission for extension to this very well. PH-1 and its abandonment. Now you are saying you are assuming that Cuadrilla will abandon the well in line with HSE requirements. So we have LCC assuming on you and you assuming on the private operator and nobody actually checking !!! Have you any idea how ludicrous this sounds and indeed is? Can you see how as a professional I may now be getting a little concerned ***? Just a little ! These wells have a rather unfortunate habit of leaking (I know but also now DU have published a study on it). They leak. Nothing to do with the tremors *** so please don't get confused here. It is all to do with time and deterioration of the casing and cement. They leak Radon, Benzene, methane etc. They need monitoring. They need monitoring for decades. The EA are saying they are "assuming" all is fine. They are saying they will not monitor for even one day! You are entirely abdicating your responsibility. You are leaving us very exposed. You are assuming others are doing their jobs just as others are assuming you are doing yours! But it is not true and so hence, neither is doing anything at all. It's a farce. I pointed this farce out to the Gov 3 years ago. Nothing has changed! If anything it is now worse. This is not a good situation we find ourselves in here ***. The EA (you) need to start being a bit more open and frank with your abilities and knowledge of oil and gas and just what you can achieve. Lancs CC need to know what these limits are. Maybe they might not have been "satisfied" then that the EA were effective and maybe they might have just rejected Cuadrilla's planning application. This "satisfied" test I had to point out to them because it appears they were not advised correctly and were not aware of the change to planning guidance issued by the Gov back in March 2014. I have asked LCC to re-consider their decisions based on my information.

As we've set out we are aware of gas being found and pressure monitored in the B annulus of the well. HSE notified us and our Groundwater Team carried out a joint inspection and a meeting on site took place on the 30th April 2014 with the Health and Safety Executive well inspectors to review the plans the company has in place for the well abandonment. There is no escape of gas to strata surrounding the well bore (I am glad to hear it – how do you know this? – please don't say Cuadrilla told you) and the gas is isolated within the annulus by the well head structure. (What exactly is this ***? Can you explain to me what exactly do you mean by "head structure"? BOP? Christmas Tree? There is no current environmental risk posed by this issue and we are happy that the changes HSE are requiring to the companies well abandonment plan will mean no on-going or residual environmental impact post abandonment. (Just the minor detail of a 2 mile borehole that has and most likely will suffer further integrity failure and unmonitored will lead to an escape gasses that pose a significant threat to the life of people /environment living in the vicinity of the well. The HSE are requiring monitoring to be undertaken to ensure the procedure has resolved the gas issue and we're liaison with them directly about this.

Hope this helps

No it doesn't – it falls way short. I am confused as to why you put this at the bottom of your mails *** when in fact you have just demonstrated you are at best "confused" and at worst "job protecting" but in either case you are not, the EA is not, in my humble opinion, doing its job properly and is leaving the people of the Fylde open to some severe health implications. At the same time you are reassuring Lancs CC that all is fine. I am getting a little annoyed now that the EA are not doing their job. The time for messing around is over. If you do not

know this industry (and you don't) then admit it. Tell Tony Grayling [of the EA] you are not in a position to regulate this industry. You admit yourself you are not going to inspect it!! The very detail that I gave evidence to Simon Toole (DECC) on 3 years ago and he admitted it was a serious "problem". It is still not "fixed" 3 years later!!! This mess cannot go on. I cannot allow unregulated fracking on the Fylde. I cannot.

Regards,

Mike

***

*** ***

Environment Manager, Lancashire

Sherwood Sandstone aquifer below the western Fylde – fresh or saline?

[extracts from blogs published in 2017 by Professor Smythe]

The Kirkham borehole

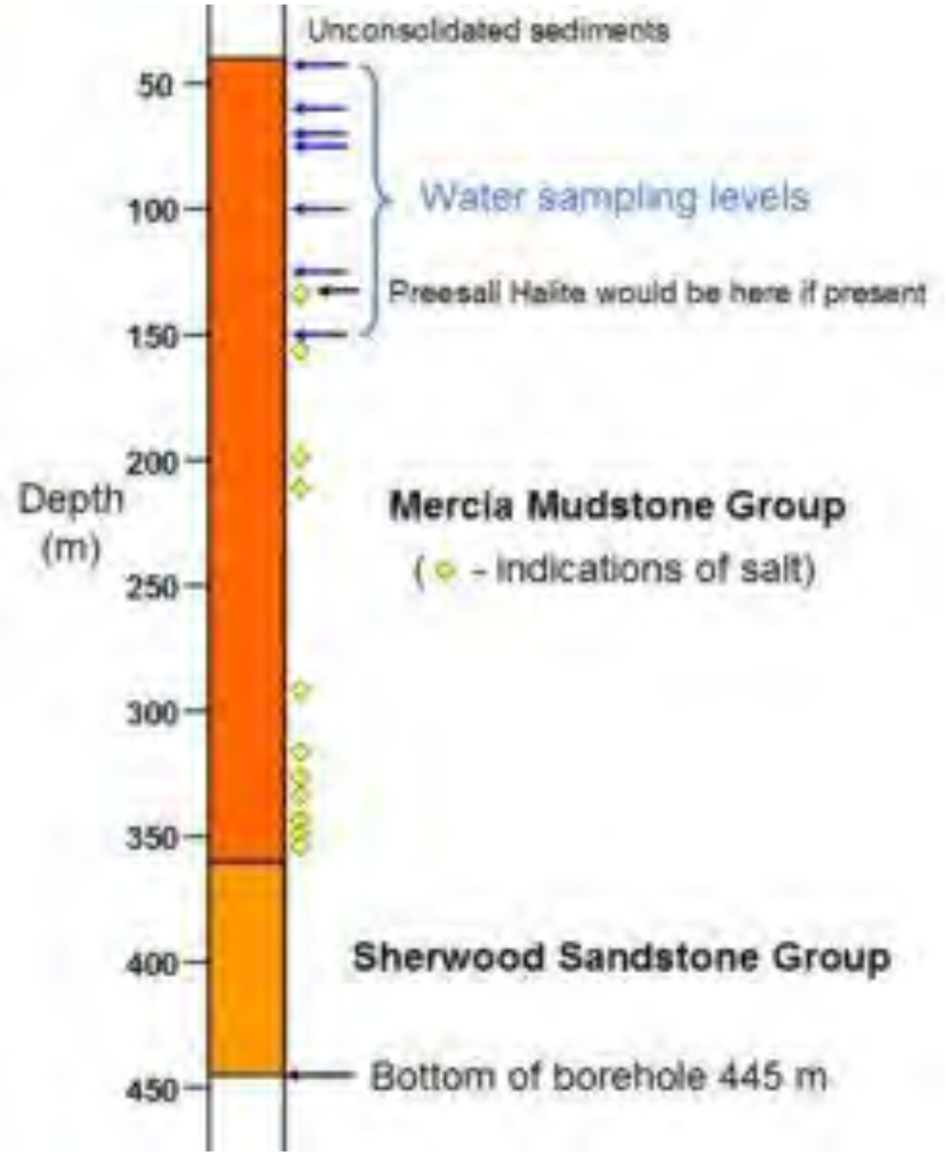

Fig. 1. Simplified geological log of the Kirkham borehole, western Fylde. Groundwater sampling levels measured by the EA are shown by blue arrows. Evidence of halite is shown by yellow diamonds. If the Preesall Halite, found further NW, were present, it would be at the level shown by the black arrow.

This borehole was drilled in 1970 as a test for underground gas storage, but has since then been used as an observation borehole. It penetrated the Sherwood at 366 m and went on down to 445 m.

The only evidence that the EA seems to consider, in dismissing the groundwater potential of the entire western Fylde, is the hypersalinity of the water samples observed in this borehole. I pointed out that this evidence is invalid because two of the three hypersaline samples were taken from levels within the Mercia Mudstones, where the observed hypersalinity, some two

or three times more salty than seawater, can be explained by perched (hydrogeologically isolated) relict halites known to exist within the Mercia Mudstones. …The groundwater within the Sherwood was never sampled.

Rowe's Model Dairies, Inskip

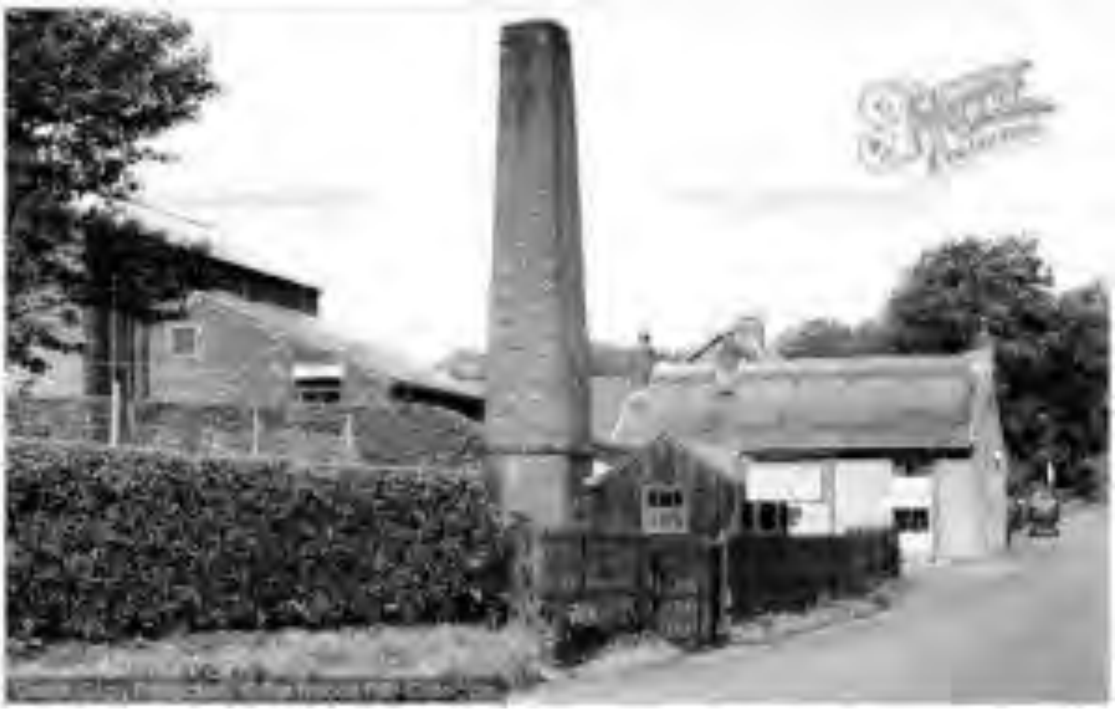

Fig. 2. Rowes Model Dairies, Inskip, c. 1950, where a 161 m deep borehole penetrated the Sherwood Sandstone aquifer at 124 m. Photo credit: Francis Frith Collection.

This borehole was drilled in 1940-41. It lies 6 km NNE of the Kirkham borehole, and, like it, is just west of the Woodsfold Fault. It is 2.7 km NE of Cuadrilla's Roseacre Wood site. It supplied a dairy and cheese factory untill the mid 1960s. Would Professor Younger have us believe that saline water was used here?

Phoenix Mill, Wesham

This borehole was drilled to a depth of 445 m in the nineteenth century. It lies 1.7 km west of the Kirkham borehole and 4.2 km east of Cuadrilla's Preston New Road site. No geological details are available, but this depth appears to be through the Mercia Mudstone Group and well into the Sherwood.

The water would have been required chiefly for the compound steam engine to run the weaving looms. The water would have re-used in a closed cycle, with a reservoir on-site. The existence of the reservoir has been confirmed from a 1911 map. Mr John Phillp of the Northern Mill Engine Society says that such an engine would require about 40,000 litres per day, but that most of this would be recycled. However, it is unlikely that a source of salty water would have been countenanced, because it would lead to precipitation and corrosion in the boiler, with resulting inefficiency.

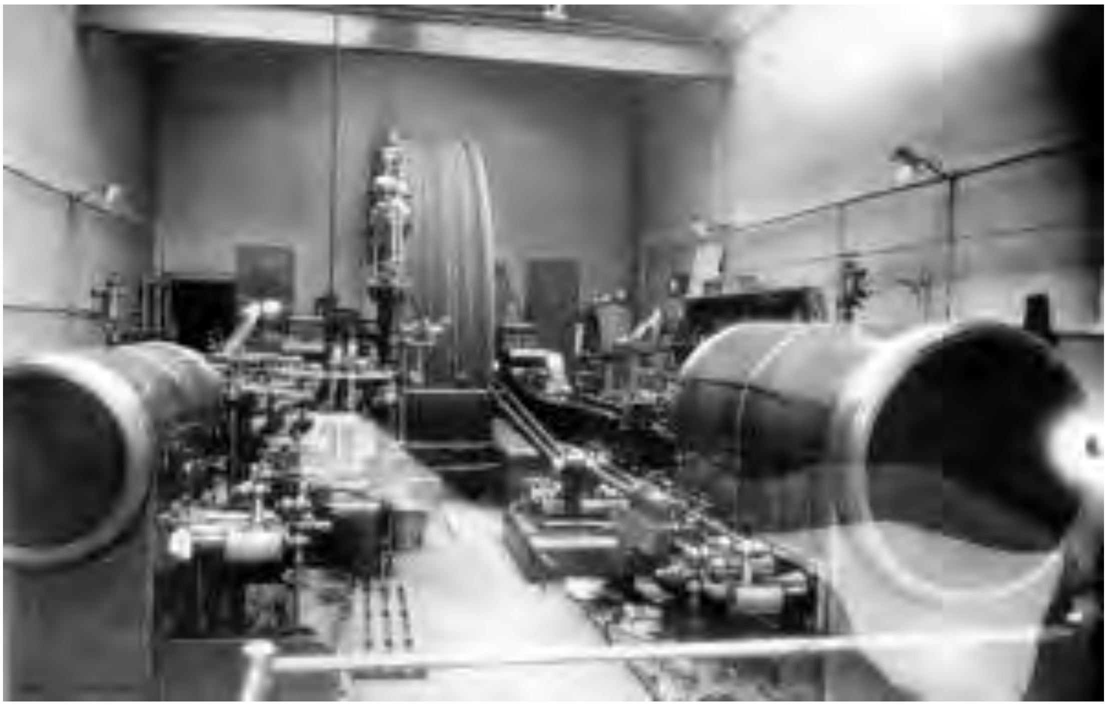

Fig. 3. Compound steam engine of the type used at Phoenix Mill in the nineteenth century. Consumption of water, which would necessarily have been fresh, was about 40,000 l per day, of which most could be recycled.

Fylde discussion

The boreholes described above are part of a group which I examined, comprising some 39 relevant wells west of the Woodsfold Fault, of depth greater than 30 m, which are available on the BGS borehole mapper website. About five of these borehole records are confidential, and/or there is no information. In addition, I studied the water composition records of 56 boreholes, which I obtained from the EA.

I think it is reasonable to conclude that it is unlikely that hypersaline or even saline (undrinkable) water was used either for the cheesemaking at the dairy or for powering the looms at the mill.

The EA believes, based on no solid evidence, that the flow across the Woodsfold Fault will be low. Next, it assumes that there will be little or no vertical flow – but this assumption ignores the presence of faults cutting the Mercia Mudstones. These could be transmissive pathways, particularly when one considers the stress regime in the uppermost 300 m below ground level.

The EA cannot find a discharge for the flow, if present, but this again ignores the presence of faults. Lastly, since the Woodsfold Fault is *defined* as a no-flow boundary, the lack of westward flow in the model cannot be used as an argument to prove that there is no westward flow.

Since I published the paper in January 2016, and taken account of the ensuing commentaries on it, I have done a little more research on the possible flow pattern of the groundwater under the western Fylde. I have obtained the 2D seismic data for the area shown in Figure 4, and mapped a newly-recognised fault, which I call the Wakepark Fault, running (coincidentally) from the Cuadrilla wells Preese Hall-1 to Anna's Road-1. In fact the fault has been previously recognised at depth, both independently by an oil company in 1994 and more recently by the

BGS, but has not previously traced up through the Mercia Mudstones to the surface. It does not appear on the current BGS paper or digital geology maps.

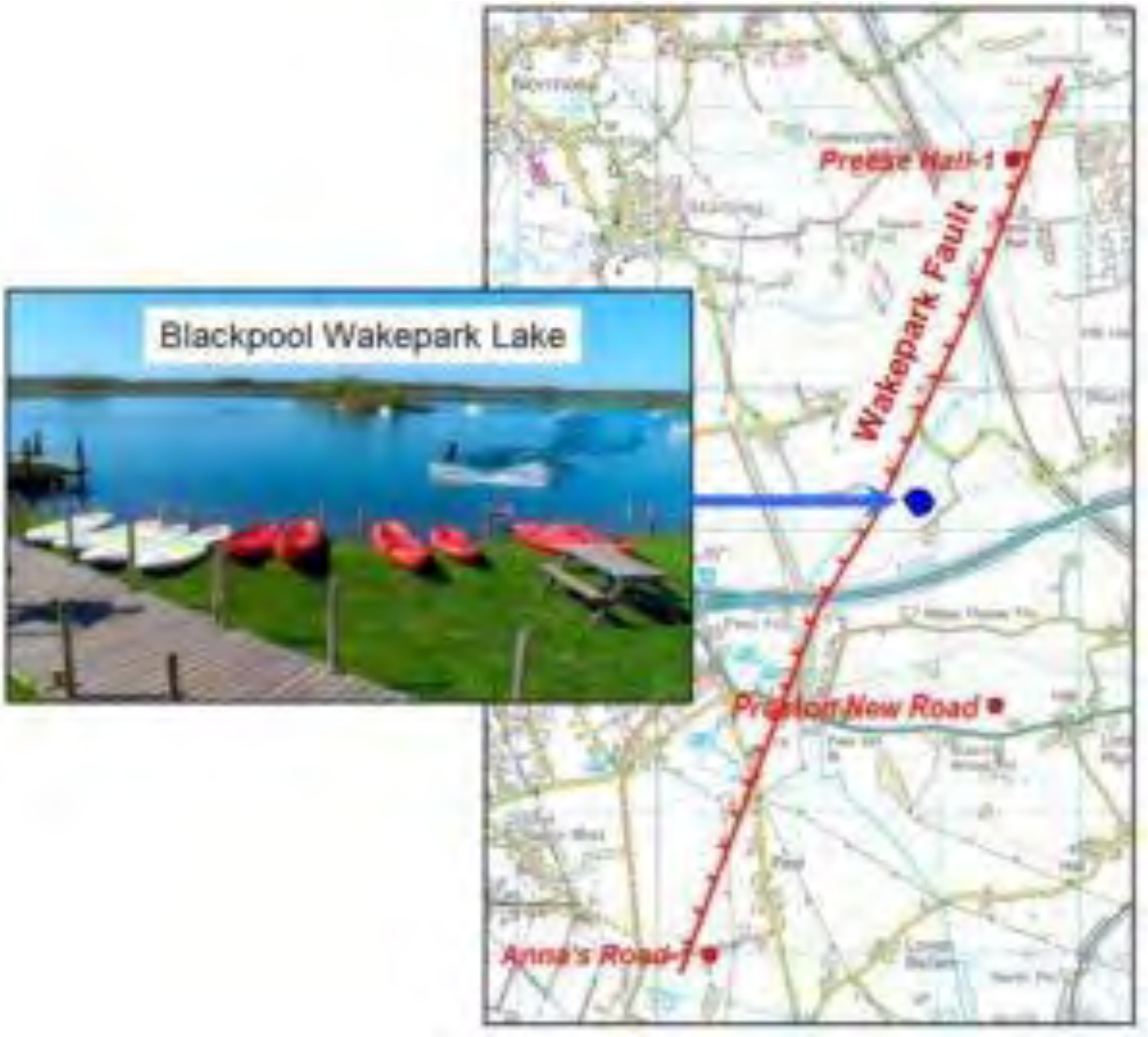

Figure 4. Blackpool Wakepark recreational lake is fed by four pure freshwater springs. These could originate in the postglacial sandy deposits in the top 30 m of the subsurface (unlikely) or else could be fed by flow up the Wakepark Fault (red toothed line). Use of copyright OS digital data acknowledged. Photo credit: Ream Hills Holiday Park.

The Blackpool Wakepark Lake is an artificial lake created from boggy ground (Figure 4), and replenished by four springs. Now, it is possible that the springs supplying this lake are recharged from the unconsolidated sandy post-glacial deposits in the top 30 m of the subsurface, but the volume of flow from such a source would seem to be far too small. The more likely alternative, in my view, is that they are fed by upflow along the Wakepark Fault from the Sherwood. This newly identified discharge is the missing link in the flow cycle, originating as recharge in the Bowland Fells, that the EA failed to find.

A spokesperson at the Ream Hills Holiday Park told me that the lake water is tested annually, and is of such good quality that it could be bottled and sold as spring water. So this is not a resource that should be lightly written off, as the EA and Professor Younger conclude.

Conclusions

My concern that the EA has written off a past and future potential groundwater resource in the Fylde is justified. Before any unconventional exploitation begins it would be prudent for the EA and/or the BGS to sample the water at SSG levels.

Manchester Marls as a seal

A crucial facet of Cuadrilla's claim that no fracking-related contamination will travel upwards at either of the two sites is that the Permian Manchester Marls underlying the SSG will provide the required seal. This layer is shown by the uppermost half of the pink layer in Figure 7, the lower half being the Collyhurst Sandstone. But this layer is only 270 m thick in the area of the proposed Roseacre-1 well[1]. Cuadrilla stated:

"The Manchester Marl locally forms a seal to underlying hydrocarbon bearing geological units. The Collyhurst Sandstone is the gas reservoir at Elswick gas field in central Fylde, where it immediately underlies the Manchester Marl."

This statement is misleading because it implies that the sandstone is a conventional gas reservoir requiring a top-seal. But the Collyhurst Sandstone is a low permeability, low porosity sandstone. It was described by the developer which took it over from the BGC-BP consortium, Eukan Energy Limited (1994), as "*a low grade reservoir rock. the porosity and permeability readings being very low.*" The Sherritt relinquishment report of 2009 shows a photo of Collyhurst Sandstone core from the Elswick-1 borehole, where it is described as:

"Very-fine-grained well-rounded quartz arenite with quartz and calcite cement. Porosity = 2 – 12 %, Permiability [sic] *= 0.01 – 8 mD (DTI well file)."*

The sandstone required fracking to produce the gas (although it should be noted that conventional fracking of this vertical conventional well has little in common with high-volume horizontal fracking of shale). Therefore its modest success as a gas producer owes little or nothing to the overlying Manchester Marls being a seal.

The Collyhurst Sandstone varies considerably in thickness, from over 500 m in the Elswick-1 well to zero in the Thistleton-1 borehole. At the application site it is 270 m thick.

Cuadrilla uses the BGS lexicon (British Geological Survey 2014) to describe the Manchester Marls, as:

"a red marl (calcareous mudstone and siltstone) with thin beds of fossiliferous marine limestone and dolomite; locally green; sandy in places especially in top part; local breccias and pebbly beds".

The thickening of the Manchester Marls in the Garstang sheet 67 area across faults is attributed to syndepositional movement on the faults (Aitkenhead *et al.* 1992). Such thickness changes are seen in the neighbourhood of the proposed Roseacre Wood well.

The Manchester Marls have a similar lithology (and a similar desert salt-flats or sabkha origin) to the Mercia Mudstone Group. Therefore their mechanical and hydrogeological characteristics will also be similar, and it is unlikely that they will provide an adequate seal to prevent upward fluid migration. In addition, the layer is cut by numerous faults, many presumed to be syndepositional in nature. Such faults zones will comprise breccias and crush rock, and so are unlikely to be good fluid seals; on the contrary, they will be transmissive.

The Health and Safety Executive report (Watson *et al.* 2008) investigating releases from potential UK underground gas storage facilities stated, regarding the Elswick gas field:

[1] Cuadrilla Elswick Ltd 2014. Temporary Shale Gas Exploration Roseacre Wood, Lancashire Environmental Statement, volume 1, June 2014

"The Collyhurst Sandstone forms the reservoir formation for the currently operating Elswick gas field. Porosity averages 5.6% and the permeability of the formation is <1mD. Evans (2007) does not give the initial pressure in the gas reservoir. The hydrocarbon trap appears to be a graben [sic]. *Some of the faults that cut the crest of the graben* [sic] *extend to the surface but are expected to be sealing over at least part of their length*."

Here we have further confirmation of the hydrogeological properties of the Collyhurst Sandstone (incidentally, the authors have confused *horst* with *graben*). But note that the faults are expected to be sealing only "*over at least part of their length*".

In conclusion, the Collyhurst Sandstone and overlying Manchester Marls are not a reliable seal for upwardly escaping fluids.

Application by Cuadrilla Bowland Limited to vary an environmental permit at Preston New Road, Lancashire: Objection to modification of proposals for microseismic monitoring

David K. Smythe
Emeritus Professor of Geophysics, University of Glasgow
La Fontenille, 1, rue du Couchant, 11120 Ventenac en Minervois, France

www.davidsmythe.org

3 August 2017

Summary

The developer proposes to scrap the previously permitted buried near-surface microseismic monitoring array, replacing it by a downhole array. No justification has been provided for this alteration. Overall, the new array would be less able to detect and accurately locate events than the originally approved array. **The requested variation should therefore be rejected.**

In the absence of previously supplied information concerning the buried near-surface array, the EA should now take the opportunity to impose two new conditions on its deployment: (1) that the eighty stations should comprise three-component seismometers, and (2) that the monitoring period be extended for at least 24 hours after the end of the last frack stage in each well. These conditions should also apply to the ten-station surface event threshold detection array.

Previously approved seismic monitoring

The original seismic monitoring plans were described in the Planning Statement of May 2014. The monitoring is to be two-fold (Section 3):

1. A ten-station surface monitoring array, and
2. An eighty-station array of buried near-surface seismometers.

The surface array is intended to monitor the fracking in real time. The seismometers are each buried in shallow pits up to 0.8 m deep. The seismometers of the buried near-surface array are each placed at 100 m depth in individual specially-drilled holes.

The type of seismometer is nowhere specified. It has to be assumed that they are all broadband vertical-component. There is an overlap in the purpose of the two arrays, since both are for monitoring the fracking. The difference is in the way that the data are handled.

The 10-station array is designed to alert the drilling (fracking) operator in real time to the occurrence of a seismic event which might trigger the so-called Traffic Light System (TLS). The monitoring at the exploration site is carried out by a seismologist who will inform the driller if a significant TLS event occurs. Appropriate action regarding the fracking can then be taken.

The Planning Statement says:

> "*The TLS system will be operated* ***during hydraulic fracturing operations*** *and will require personnel to visit each site to change the batteries used to power the seismometer, data recording and communications equipment that feed data back to operators at the well pad in real time.*" [my emphasis].

This statement is unclear as to whether the TLS system will continue to operate after the cessation of a frack stage, when the well is 'shut in'. The history of earthquake triggering at Preese Hall-1 shows that the largest event occurred 10 hours after shut-in at the end of frack stage 2.

It is therefore imperative that the TLS array must be operated 24 hours per day throughout the whole period of stage-by-stage fracking, and should cease no less than 24 hours after the end of the last frack stage at any one well. If not, significant seismic events may be missed.

In addition, the ten-station TLS array must comprise 3-component seismometers, not simple vertical-component seismometers, to enable more accurate location of the events.

In contrast, the data from the buried near-surface 80-station array are intended "*to measure the extent and rate of fracture propagation within the shale rock. … These stations will provide data on the direction and extent of the small fractures that are opened up in the underground shale.*" In practice these stations will also provide a more accurate determination of any triggered earthquakes than the surface array of ten stations. However, this cannot occur in real time, because "*During hydraulic fracturing operations, data will have to be downloaded from the buried array points after each time a well undergoes hydraulic fracturing. This will be done remotely via the mobile phone network*" (Planning Statement, p. 21). In other words, the data are stored locally for subsequent downloading and processing in batches.

Variation requested

The summary statement on variation of the existing permit states (section 3.1 Seismic Monitoring):

> "*The variation is to maintain the same quality of seismic monitoring information being generated but use a different approach to capture the information. The waste management plan includes details of downhole seismic equipment in an offset well rather than the previously proposed buried micro seismic array.*
> *The purpose of the seismic monitoring is to monitoring the extent and orientation of fractures to ensure groundwater is protected and fractures remain within the permitted boundary.*"

Some greater detail is provided in version 8 of the Waste Management Plan (June 2017, pp 14-16), which states:

> "*Monitoring of fracture growth will be captured by the temporary installation and operation of downhole micro seismic geophones (sondes). The approach of drilling multiple wells before hydraulic fracturing subsequently means that an offset well, i.e. the well next to or in close proximity (on the same pad) to the well which is about to be*

hydraulically fractured, can be utilised to locate micro seismic geophones for the monitoring of fracture growth. Micro seismic geophones are lowered into the offset well at depth within the target formation before hydraulic fracturing occurs (see Figure 3). Data is [sic] *then acquired and transmitted through wireline and collated at surface. The data will be processed to show event location, orientation and extent of induced fracture growth within the target formation.*

Future wells (3 & 4) which are to be hydraulically fractured can utilise an offset well for monitoring of their fracture growth using the same technique. This can be achieved in wells which have already been hydraulically fractured by plugging the well (bridge plug) and segregating the hydraulically fractured area from the micro seismic geophones which are lowered into the heel of the well. Check shots will be performed to calibrate the micro seismic geophones. The estimated accuracy is 20m of x, y and z [.]

Potential propped fracture growth is detailed further in the ES Induced Seismicity Chapter."

It is proposed that the surface 10-station TLS monitoring array remains as previously approved. But the developer now proposes that the 80-station buried near-surface array be scrapped and replaced by downhole monitoring using an array placed in an adjacent (offset) well. This requires that at least one additional well be drilled before the first well is fracked – a significant departure from the original timeline of sequential drill-then-frack on a well-by-well basis.

According to the figure 3 referred to above, the downhole array appears to comprise 12 sensors at an approximate 100 ft interval, positioned with the lowermost sensor at the curve (the heel) where the hole turns from vertical to horizontal. It is asserted that the "*variation is to maintain the same quality of seismic monitoring information".* This would only be true of microseismic events in the near field of the array, that is, within a few hundred metres or so; for events situated further away than that the buried array will generally provide more accurately located data.

Comparisons of near-surface and downhole arrays

Eisner *et al.* (2010) compared a large surface array of single-component receivers with a downhole array. They performed model studies and also presented a case history. Their model surface array comprised 121 seismometers on an 11 x 11 grid with a 600 m spacing. Thus the overall aperture is 6 km. The modelled event is at 3 km.

Their modelling results show that, overall, the surface array event positioning is more robust than the downhole results, because it is less sensitive to the velocity structure. Furthermore a velocity model can be derived from the surface data by calibration check shots. The surface array data could be improved if three-component seismometers were used, to enable independent identification of P- and S-waves.

The case study showed that locations derived from surface monitoring have less scatter in both vertical and horizontal directions.

A comparison of monitoring arrays was carried out by the hydrocarbon industry service company Schlumberger (Peyret *et al.* 2012). In their terminology the near-surface buried array is called a shallow hole grid. They note that a surface array, even if comprising hundreds of seismometers, is always more susceptible to environmental noise than the

shallow hole grid. They recommend a minimum of 25 holes (in a 5 x 5 array) for the shallow hole grid to avoid spatial aliasing. Their conclusions read, in part:

> "*microseismic events caused by hydraulic fractures can be reliably detected and located with both surface and Shallow Hole Grids. … When a monitoring well is close to the stimulated well, the downhole data set is richer. … The inclusion of 3C sensors in the design is crucial to detect the S signal which, among other things, reduces event depth uncertainty. The 2D geometry of a … Shallow Hole Grid allows more information to be extracted from the individual event signals and lends itself to reservoir-wide interpretation.*"

Maxwell (2014) has published a book about microseismic monitoring specifically for shale fracking. Regarding the importance of full (continuous) monitoring, he writes:

> "*Traditionally, hydraulic fracture monitoring is a short-term monitoring activity that starts and stops with the treatment injection period. However, continuous monitoring including baseline and post-injection monitoring is becoming increasing common, particularly for concerns about induced seismicity. Post-injection monitoring also can provide information about long-term performance of the stimulated fracture network*."

In a section entitled *Choosing surface or downhole monitoring*, Maxwell does not favour one method over the other, stating merely that the project engineering objectives should guide the choice. He does, however, suggest that in new (shale) formations, where microseismic source strength is unknown, a conservative approach would be to start with a downhole array as close as possible to the target.

Swiech and Wandycz (2015) compared surface and downhole monitoring by means of synthetic models, and found that their downhole array provided better hypocentral locations. But their downhole array comprised 21 three-component seismometers distributed over 400 m vertically, whereas their surface array comprised 21 seismometers in a double concentric circular pattern with an outer diameter of about 300 m. This array diameter is unrealistically small for locating accurately the model event at 2830 m depth. The array aperture is only about one-tenth of the depth to the target. Therefore their conclusions can be discounted.

Discussion

No justification has been provided by the developer for the requested variation, and, as with the original application, insufficient detail has been provided. There have been no technical advances since 2014 which might justify the requested permit variation from surface to downhole monitoring.

The main limitation of a surface array is the lower signal to noise ratio caused by the greater distance from the surface to the source, and the greater ambient (environmental) noise. But the latter problem has been solved by the plan to place the seismometers at the bottom of 100 m deep holes. Furthermore, this is also well below the unconsolidated ('drift') layer which is typically up to 30 m or so thick in the Fylde, and which degrades the signal coming up from depth. The buried array provides better accuracy in the horizontal (x and y) directions, while less accurate in vertical depth (z direction) estimation. Overall, the use of a downhole array in several wells will yield mutually inconsistent datasets, and the better accuracy in depth (z direction) estimation with a downhole array is also outweighed

by the poorer x, y direction source location.

The 2014 Environmental Statement concluded:

> "*The interpretation of the 3D geophysical (seismic) survey, in the context of defining strata boundaries, has been made on the basis of a correlation between the results of the vertical seismic profile (VSP) and downhole geophysics. This data has come from other nearby wells including, Preese Hall, Grange Hill, Elswick and Thistleton. The interpretation of ground conditions is therefore based on geological information that does not specifically cover the Preston New Road site, however, interpretation of strata boundaries has been found to be consistent throughout all wells and it is unlikely that the ground conditions will vary to an extent that will affect the results of the assessment, particularly as information is available from the nearby Preese Hall well.*"

Thus the project area cannot be described as being in virgin territory, since there exists already a 3D seismic survey for which a velocity-depth model has been accurately calibrated by the several pre-existing wells in the locality. Therefore Maxwell's suggestion, that in previously unknown geology a downhole monitoring array is preferable initially to a surface array, does not apply here.

The intrinsic extra robustness of data obtained from the buried near-surface array can be improved further by the use of calibration shots, as noted above. This has already been promised, as a by-product of the perforation process prior to fracking, in the 2014 Environmental Statement:

> "*Explosive charges have the benefit of providing calibration data for the buried array, and as such, may be used on initial perforations*." (p. 47)

The buried near-surface array data should thus be regarded as the definitive dataset for locating microseismic events, including any possibly triggered earthquakes in the near or far field which should have been detected in real time by the surface array. The latter array, with only ten stations, will provide relatively poorer event location. It is crucial that accurate event location be provided if events on pre-existing faults are triggered, to avoid any controversy similar to that surrounding the location of the 2011 events at Preese Hall-1.

Recommendations

The buried near-surface array has been well-designed, with a large aperture, a sufficient number of stations, and with the outstanding benefit of being placed in 100 m deep holes.

The proposed change from use of the buried near-surface array to a downhole array is unjustified on technical grounds. It is a technical degradation and should not be permitted. The EA should take this opportunity to place further conditions on the deployment of the surface array, to fill the vacuum caused by the developer's failure to specify sufficient detail in its original plans, as follows:

1. The 80 seismometers should be three-component, not single-component.
2. The duration of the recording should begin at least 24 hours before the start of any fracking and should run continuously for 24 hours after the end of the last frack stage.

Condition 1 does not impose an additional significant burden on the developer, since the marginal cost of installing a three-component seismometer in place of a single-component seismometer is negligible, compared to the cost of drilling the 100 m hole and installing the data storage and power facilities. The tripling of data volume entailed by this condition is similarly a trivial extra burden. The benefits of being able to process P- and S-waves separately if three-component data are thereby acquired may subsequently be seen to be invaluable, for example if the velocity model needs to be modified to account for anisotropy. Event positioning will thereby be far more accurate than using a 12-sensor downhole array.

Condition 2 is required in view of the Preese Hall-1 experience, as well as in view of the recommendation by Maxwell (2014). The collection of baseline data before the start of fracking has already been promised (2014 Environmental Statement, Table 4.1). In the case of the TLS ten-station array the advance timescale for baseline data collection has been promised as "*at least four weeks before hydraulic fracturing commences*" (2014 Environmental Statement, p. 29).

References

Eisner, L., Hulsey, B.J, Duncan, P., Jurick, D., Werner, H. and Keller, W. 2010. Comparison of surface and borehole locations of induced seismicity. *Geophys. Prospecting*, doi: 10.1111/j.1365-2478.2010.00867.x

Maxwell, S. 2014. *Microseismic Imaging of Hydraulic Fracturing: Improved Engineering of Unconventional Shale Reservoirs.* Distinguished Instructor Series, No. 17, Society of Exploration Geophysicists, Tulsa, Oklahoma, 214 pp.

Peyret, O., Drew, J., Mack, M., Brook, K., Cipolla, C., and Maxwell, S.C. 2012. Subsurface to surface microseismic monitoring for hydraulic fracturing. *Annual Technical Conference and Exhibition, SPE 159670*.

Swiech, E. and Wandycz, P. 2015. Differences between downhole and surface micro-seismic monitoring. *Mining Sci.* 22, 65-73.

Objection to approval of Cuadrilla hydraulic fracture plan for PNR-2

David Smythe

12 November 2018

Summary

Cuadrilla Bowland Limited (hereinafter the Operator) still does not understand the geology at its wellsites, even after nine years of active exploration in the Fylde. Its geological interpretation of the pre-Permian at Preston New Road (PNR) has changed significantly between early 2015 and now. Some of the interpretations do not make structural sense, so are untenable even on their own internal evidence. A major error in the prior interpretations was discovered by the absence of the prognosed 300 m of Millstone Grit at Preston New Road-1, spudded on 16 September 2017 and completed on 11 January 2018. My access to the 3D Bowland-12 seismic survey, finally obtained on 6 November 2018, confirms that Cuadrilla's current interpretations are untenable. Major faults cutting the entire section, including the post-Hercynian Permian and Triassic sediments, have been omitted from Cuadrilla's example cross-sections. These were interpreted by myself based on the 2D seismic dataset, and have been confirmed by the 3D survey. I have resolved the paradox of the absent Millstone Grit, by interpreting major faults within the Carboniferous as reverse faults with a strong strike-slip component. This style of faulting is consistent with the earthquake fault plane solutions.

Cuadrilla should be required to re-interpret properly its 3D dataset in the light of PNR-1, before the HFP for PNR-2 is authorised by the OGA and the EA.

Cuadrilla's changing geological interpretations

It is normal for interpretations to be modified when new data, such as the results of drilling a well, become available. The biggest change in interpretation usually arrives with a 3D seismic survey replacing a network of 2D surveys. However, Cuadrilla has long had available the results of Preese Hall-1, drilled in 2011, and the Bowland-12 3D seismic survey shot in 2012. The only significant addition has been the drilling of PNR-1, completed in January 2018.

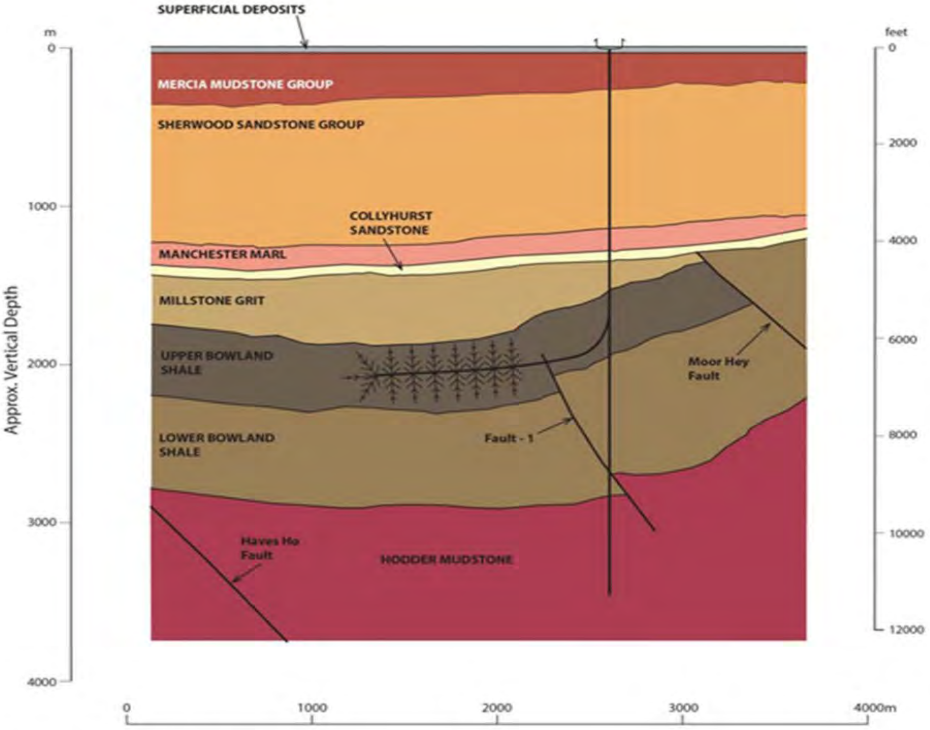

Figure 3.2: Geological strata.

Fig. 1. E-W geological interpretation through PNR (2015).

Figure 1 comes from the Environmental Statement, Vol. 1 (2015). It is an E-W section through the PNR site, as are all the succeeding cross-sections. This interpretation is dubious in its own right. No seismic reflection data were used to support it. I wrote in my Objection document to Lancashire County Council in 2015, questioning the geological soundness of the diagram:

> *" The 'local' fault, labelled Fault-1, through which it is proposed to drill the vertical pilot hole has a throw of 100 m down to the west at Base Upper Bowland level, increasing to about 150 m at Base Lower Bowland Shale. It has the geometry of a reverse fault, but it may have been a vertical normal fault at the time of deposition of the Bowland Shales, and only later tilted down towards the west. It is mapped by the Applicant as if it were a growth fault active during the deposition of the Upper Bowland Shale. But this is suspicious for two reasons:*
>
> *What other evidence is there of tectonic activity during this time? What is the flexure above the prolongation of the fault at the Top Upper Bowland shale horizon?*
>
> *The Applicant's interpretation is questionable, because it may be an attempt to minimise the possibility that faults such as this one post-date the Carboniferous, i.e. they are of Hercynian origin or re-activation, like most of the faults in this area, and therefore are likely to penetrate higher up than the Bowland Shale. It is more plausible that the fault cuts up through the Millstone Grit to the base of the Collyhurst Sandstone, just as does the Moor Hay fault lying 1 km to the east. In other words the little monoclinal flexure above the fault should be remapped as a fault offset. This interpretation is more likely in my view (unless evidence can be adduced that the Upper Bowland was a time of growth faulting) because it is geometrically implausible that a fault with a throw of 100 m can die out upwards within another 200 m, as Cuadrilla has interpreted.".*

In November 2017 the identical interpretation was used for the draft HFP for PNR-1z (Figure 2).

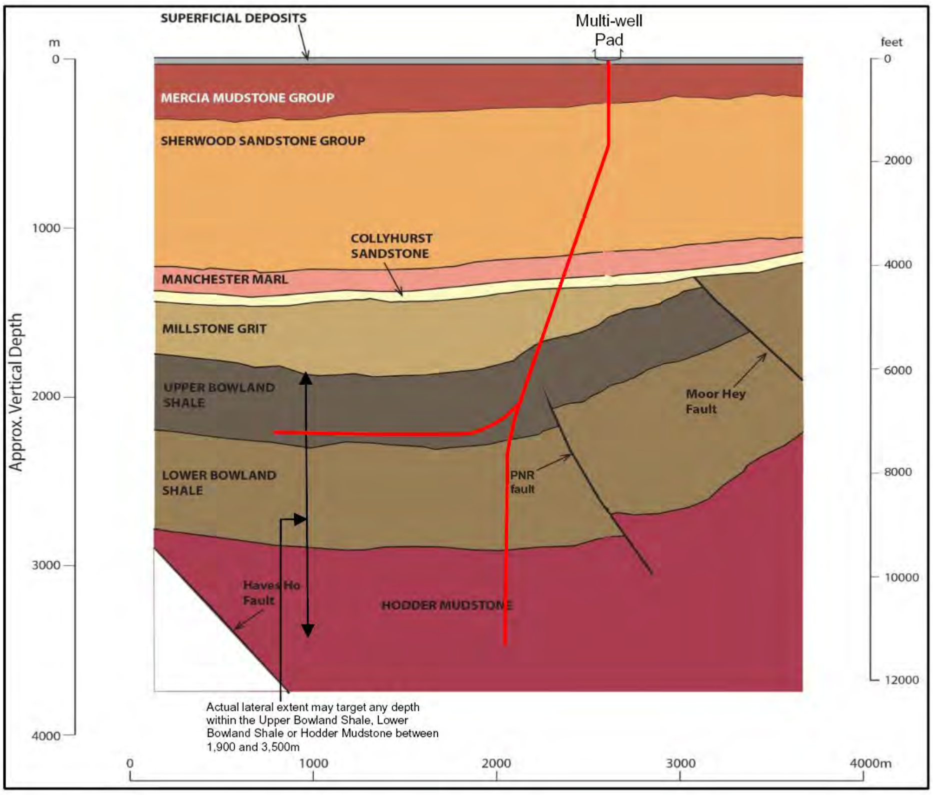

Fig. 2. HFP cross-section (November 2017) showing revised positioning of PNR-1 (vertical) and PNR-1z (horizontal).

The wellpath has been shifted to avoid Fault-1, now relabelled as the PNR Fault. The likelihood that this fault penetrates higher up, as I suggested in 2015, has been ignored. The horizontal to be fracked lies at about 2300 m depth, near the base of the Upper Bowland Shale.

In June 2018 the diagram was altered to take account of the fact that no Millstone Grit was encountered in drilling PNR-1 (Fig. 3). The post-Hercynian geology remains identical. The locations of the Moor Hey Fault and the Haves Ho Fault are unchanged, as is the base of the Lower Bowland Shale.

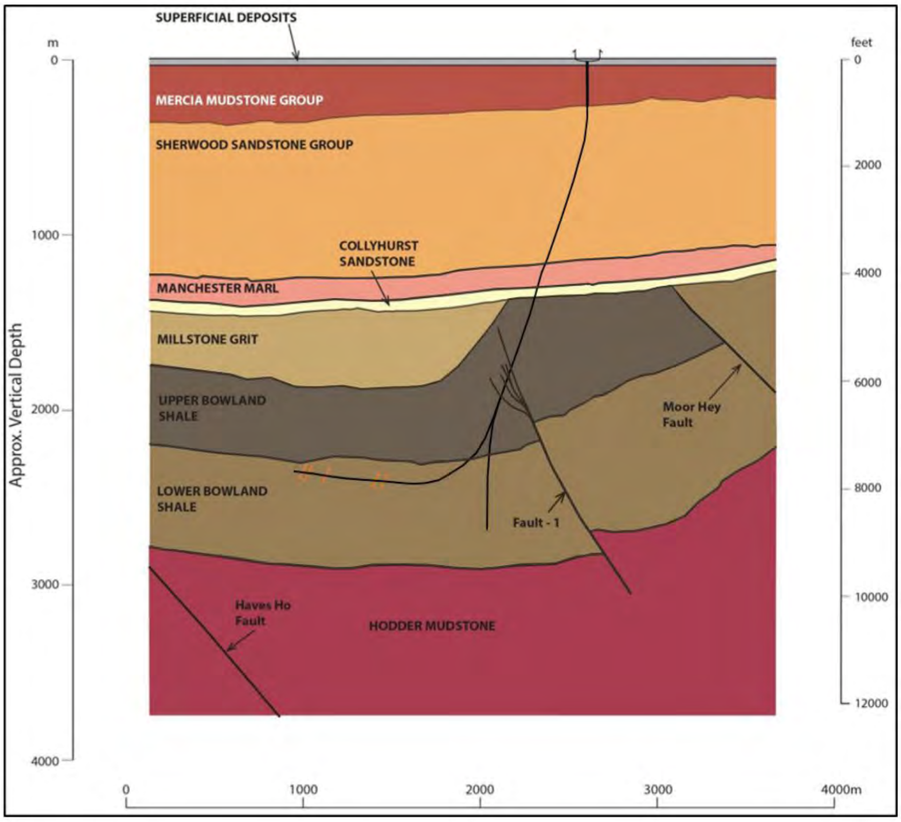

Fig. 3. June 2018 interpretation (HFP for PNR-1z).

The PNR Fault (now re-named back to Fault-1 again) is shown cutting the Upper Bowland Shale, together with a number of splays. Presumably there was some evidence for faulting encountered in the drilling. Note that a reverse fault like this would result in repetition of log data and of lithologies. The base of the Millstone Grit has been folded upwards to avoid having the layer intersecting the wellbore. This is intrinsically unsound, since it leaves the Upper Bowland abruptly thickening to the east. It might have been more logical to have extended Fault-1 up to the sub-Permian unconformity (Base Collyhurst Sandstone), while re-drafting the base of the Millstone Grit to avoid intersecting the well.

The latest version of the geology is in the current version of the HFP awaiting final approval (Fig. 4). This time the thickness increase of the Upper Bowland has been 'solved' by making its base turn upwards to a dip of about 60°, parallel to the top of the layer west of the wellbores. The base of the Lower Bowland has been similarly upturned in the vicinity of the Moor Hey Fault to an unlikely dip of about 80°. The post-Carboniferous succession remains unchanged from 2015.

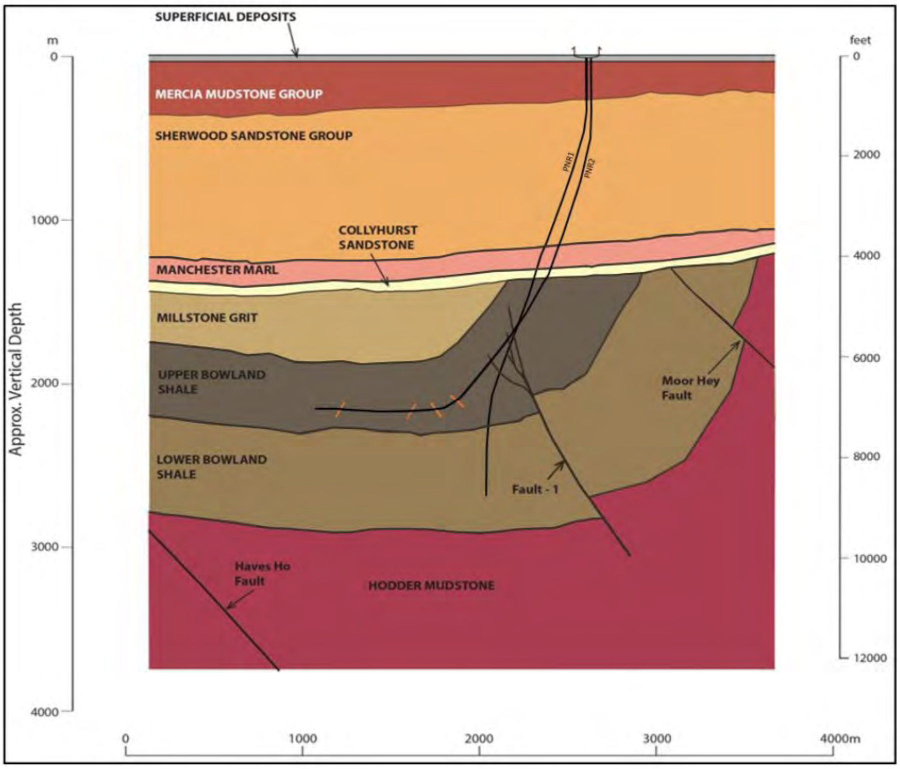

Fig. 4. Current version of the HFP for PNR-2 (November 2018).

Evidence from the 3D seismic survey

The three versions of HFP geological interpretations (Figures 2-4 above) have been accompanied by E-W vertical seismic displays in depth, not two-way time. These are shown in Figures 5-7 below, respectively.

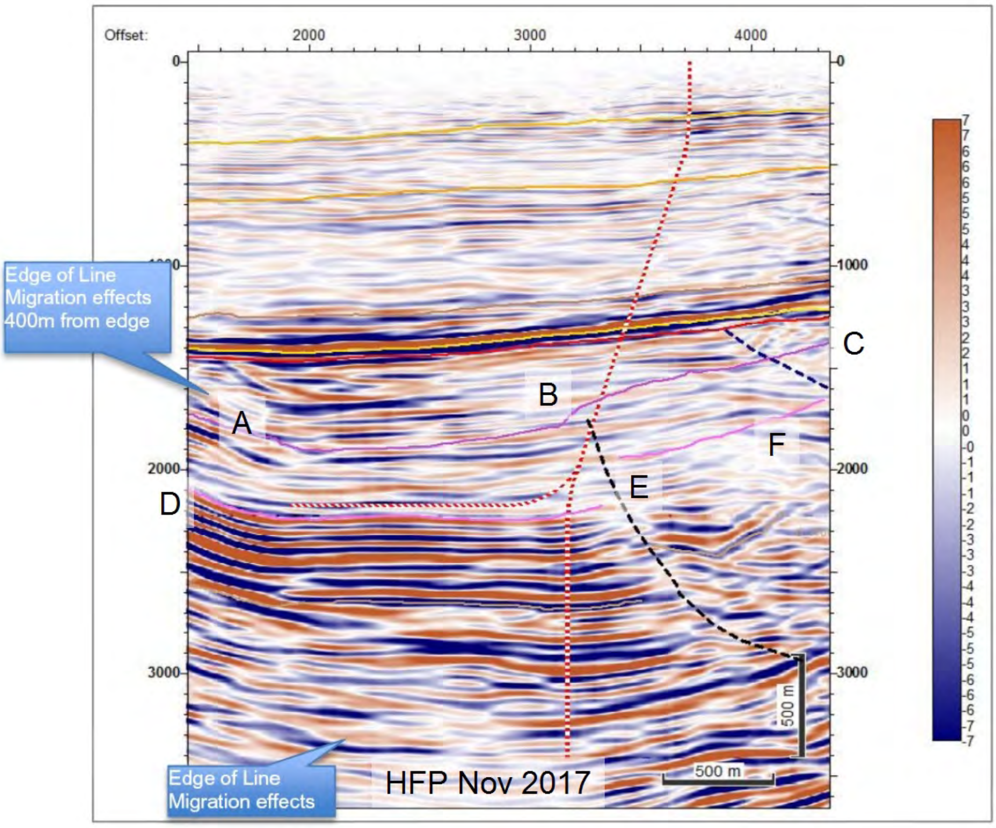

Fig. 5. Vertical seismic display (November 2017).

The seismic display covers the same E-W extent as the geological interpretation which it accompanies (Fig. 2). The diagram correctly points to the migration artefacts (blue labels) coming in from the western edge of the survey. There are three yellow markers visible in the upper part of the section indicating (presumably) Top Sherwood Sandstone, Top St Bees Sandstone and the main Top Carboniferous unconformity. Less visible are two other brown markers. All these horizons have the appearance of having been picked automatically, by an algorithm that searches through the data volume from a seed point. This method is suitable as a first pass interpretation tool in simple layering. There are minor errors which should have been manually corrected, but presumably the Operator is not interested in the accuracy of mapping that does not bear directly on the target rocks.

I have added labels A-F to the diagram. At A, the Base Millstone Grit is shown bending upwards, following the possible migration artefacts. This may or may not be correct, but it is inconsistent with the geological diagram (Fig. 2). At B the horizon takes a dog-leg (monocline) to avoid the tip of Fault-1 (aka the PNR Fault); however, the tip of the fault is now shown cutting the planned wellbore - again, inconsistent with Figure 2. At C the Base Millstone Grit is shown cutting through the Moor Hey Fault at significantly greater depth than shown in Figure 2. The lack of offset across the fault implies that the Operator must regard the fault is having a purely strike-slip component. At E the PNR Fault offsets the Top Lower Bowland by about 200 m. The track of the fault through reflector offsets looks reasonable. But at F the Top Lower Bowland cuts across reflectors; this is unreasonable, and gives the impression of careless or hurried mapping.

Figure 6 accompanies the June 2018 version of the HFP for PNR-1z. The E-W extent of the image has been truncated relative to the geological section (Fig. 3), so that the only point of reference is the wellhead.

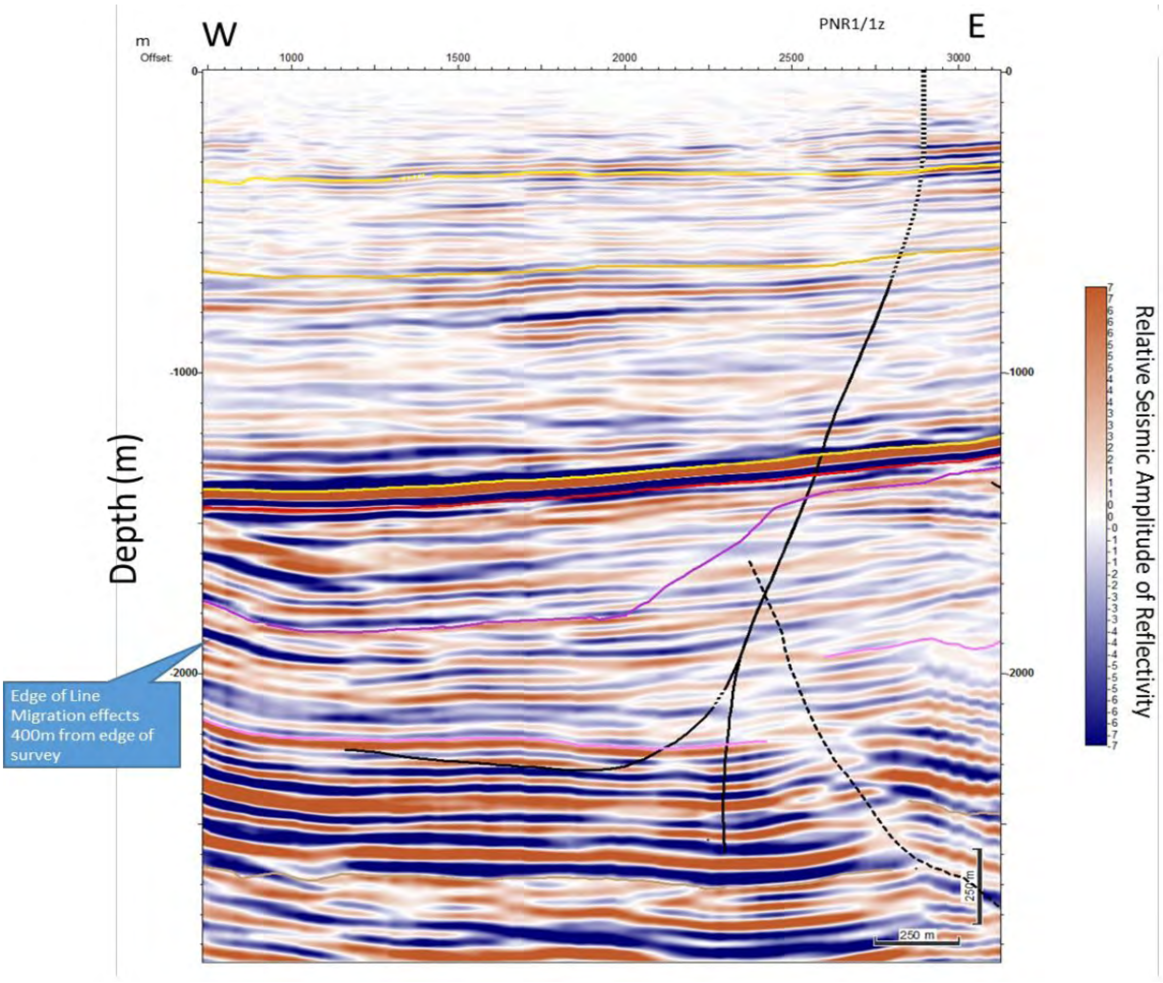

Figure 6. Seismic display for PNR-1z HFP (June 2018).

Here we see the Base Millstone Grit thinning in a wedge just west of the wellbore, and cutting across reflectors at and angle of about 40°. It then continues east, implying that there is about 50 m of Millstone Grit at the well. Again, this is careless and inconsistent mapping. Below it, Fault-1 is mapped as intersecting the wellbore, cutting across continuous reflectors at 1600-1800 m depth.

Lastly, Figure 7 shows the vertical seismic display to accompany the current geological cross-section shown in Figure 4 above.

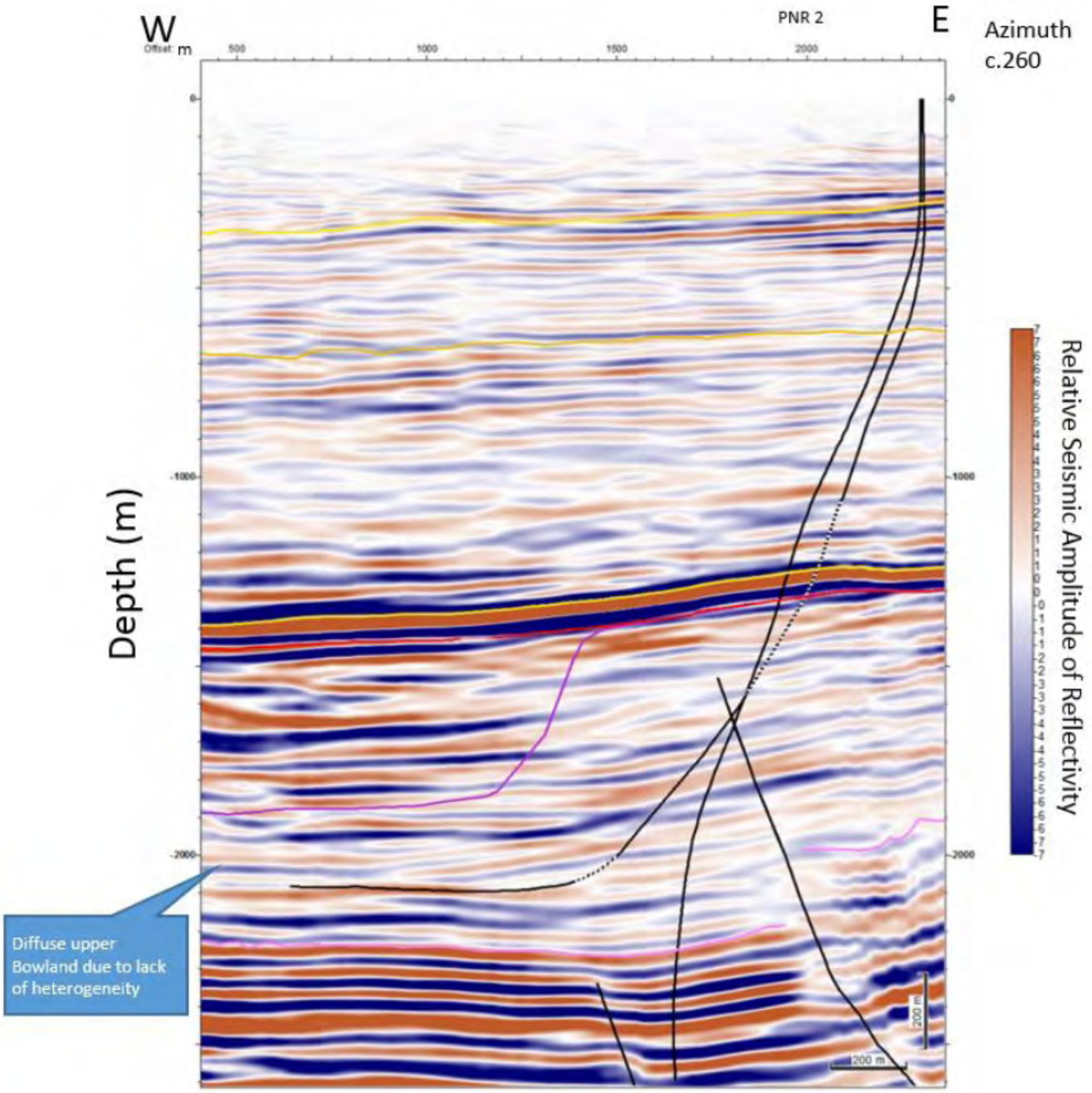

Figure 7. Seismic display for PNR-2 HFP (November 2018).

Once again there is no map on which to locate the display, which is now stated to be at an azimuth of about 260° (presumably parallel to the horizontal section of PNR-2). The display is even more truncated than earlier versions. The Base Millstone Grit is now shown wedging out abruptly over about 250 m, cutting across strong reflectors. There is now no thickness of Millstone Grit shown at the wellbores. Fault-1 similarly cuts unrealistically through strong reflectors around its intersection with the wellbores.

Preliminary evidence from the 3D seismic survey

The available version of the 3D dataset is the original migration (UKOGL suffix _OM). No other documentation has been supplied, such as the acquisition and processing reports. The Inline and Xline numbering have been taken from the trace headers; their names seem possibly swapped over, because I understand that in the acquisition the shot lines were N-S, and these are conventionally called the Inlines. Herein, for the avoidance of doubt, the Inlines are oriented E-W. Also, for the avoidance of doubt, the results discussed below from my independent mapping are based on just a few hours' work, and may well be subject to revision.

Some of the Operator's seismic displays appear to have been based on a revised pre-stack migration, with vertical scale in depth. It is unclear whether the processing involved pre-stack time migration with subsequent depth conversion, or pre-stack depth migration. In either case, the additional processing available to the operator will make no significant difference to the discussion herein.

Wakepark Fault

Using the 2D seismic database only, in 2017 I mapped a new fault at the west of the 3D survey area which I named the Wakepark Fault (see Appendix A). It had been independently mapped at Top Permian level by British Gas and by Eukan Energy, former licensees, in 1991 and 1994. I remapped this fault and showed that it extends up to near-outcrop (Figure 8). It is a normal fault, extending in a NNE-SSW azimuth, with its outcrop approximately from the vicinity of from Anna's Road-1 to near Preese Hall-1, passing by the artificial Wakepark water sports lake some 1500 m NW of the PNR pad.

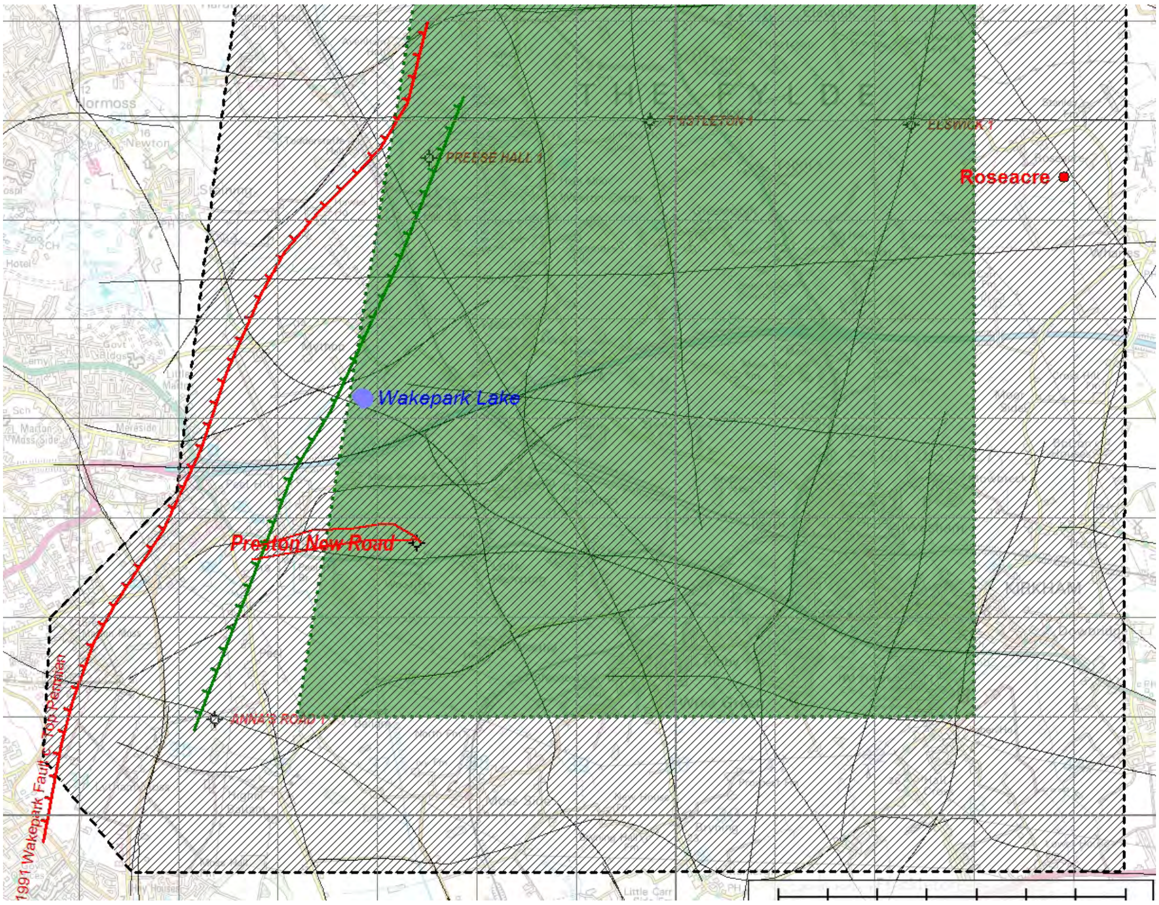

Figure 8. Wakepark Fault at outcrop (green) and at Top Permian level (red), based on 2D seismic data (thin black lines). The surface extent of the 3D survey is shown by the hatched area, within which the green polygon indicates the full-fold coverage.

The outcrop lies over the toes of PNR-1z and PNR-2, and at the depth of these two wells it is over 1 km to the west. I have confirmed the existence and accuracy of this mapping now that the 3D dataset is available. The 2D-mapped fault lies within one or two 3D bins (25-50 m) of its more accurate placement using the 3D data. However, it can be seen from Figure 8 that the fault lies mainly in the outer fringe of the 3D volume, where fold diminishes progressively from 100% to zero. Thus a combination of the 2D data and the 3D volume is needed to map the fault.

The importance of this fault is that it might be a feeder for the four freshwater springs that replenish the Wakepark lake. If so, then the source of the water might be fresh water in the upper Sherwood Sandstone at 300 m depth. This is discussed further below.

There is no mention or depiction of the Wakepark Fault in any of the Operator's documentation.

Absence of Millstone Grit

I have concentrated on the western portion of the 3D data volume, to determine why the Millstone Grit is present at Preese Hall-1 but proved to be absent at PNR-1. I illustrate my findings with a timeslice at 996 ms, Xline 2144 and Inline 5285. The timeslice is shown in Figure 9.

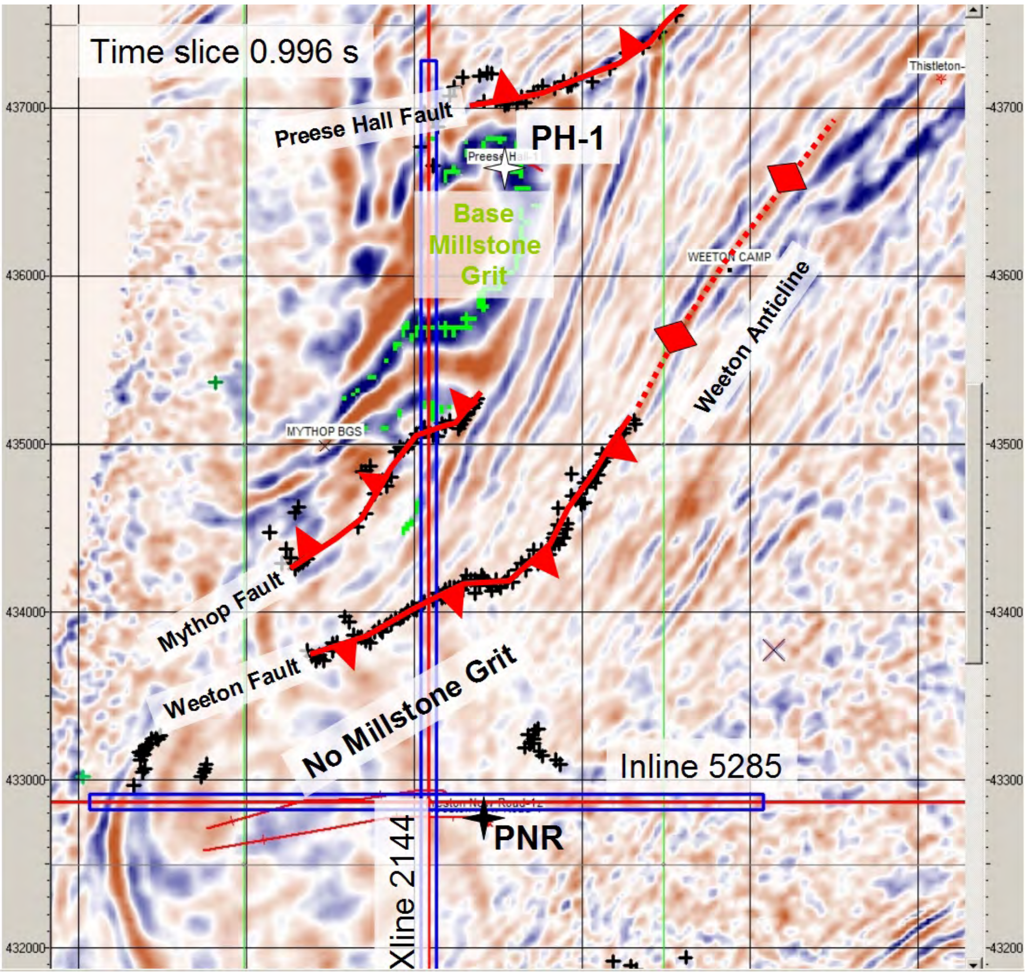

Figure 9. Timeslice at 996 ms showing structure at near base Millstone Grit. Green picks of the Base Millstone Grit are shown on the dark blue (positive amplitude) seismic data.

My main finding is that there is a complex steep reverse fault below the pre-Permian unconformity, called the Weeton Fault. It bounds a block to the NW where the Millstone Grit is present, as proved at PH-1, from the area to the south including PNR where the Millstone Grit is absent. The Weeton Fault changes character running north-eastwards, becoming a sharply pointed (chevron) anticline

with a fault bifircating the axis. This style of faulting is consistent with the earthquake fault plane solutions.

Figure 10 shows a preliminary contour map of the Base Millstone Grit, in units of TWT.

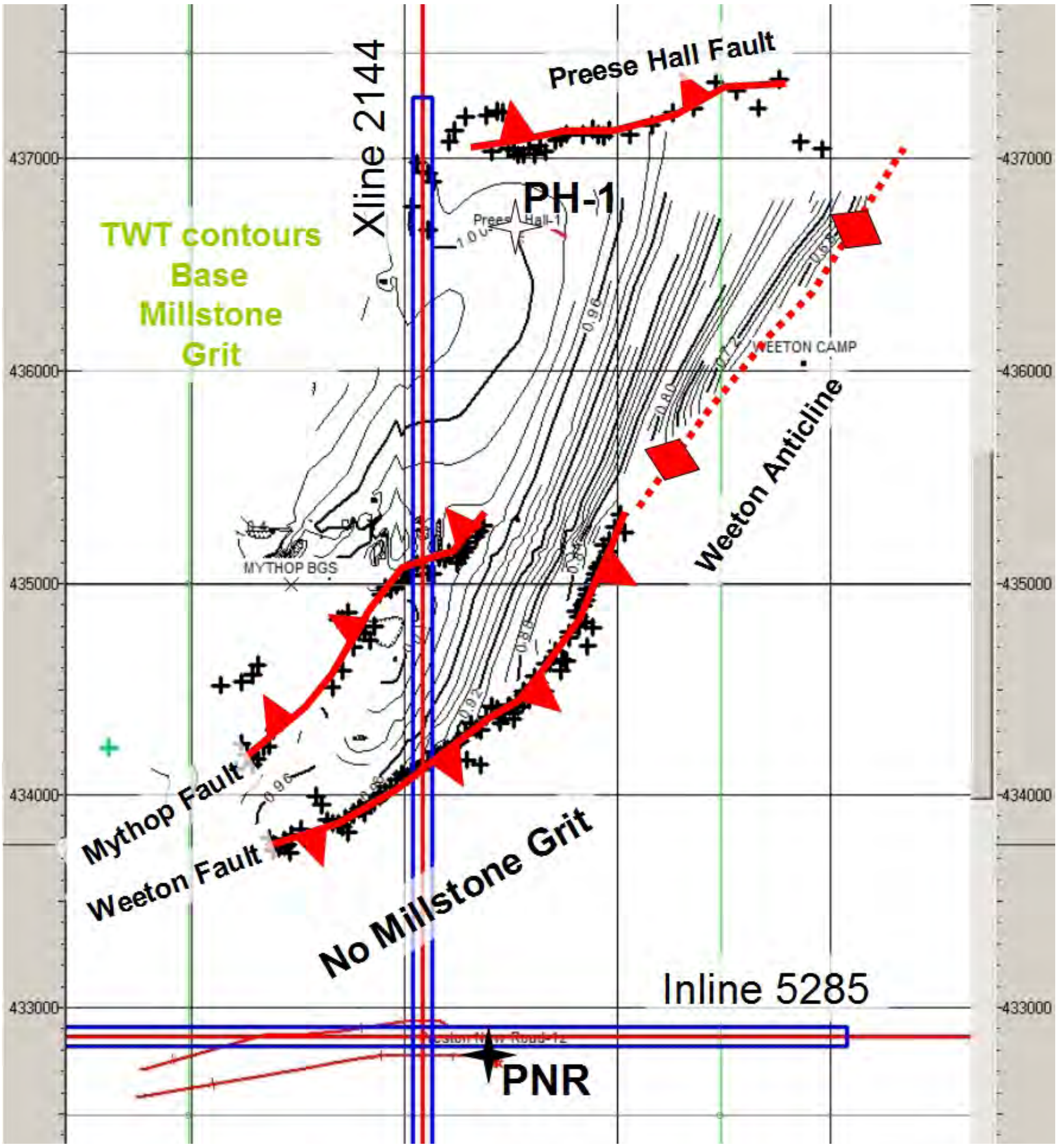

Figure 10. Contour map of Base Millstone Grit.

The Preese Hall Fault is a south-verging high-angle reverse fault with a classic horse structure. However it is not shown on sample line Xline 2144 (Figure 11). Xline 2144 runs from 500 m west of PH-1 south through the heels of the PNR wells. The Weeton and Mythop Faults, both reverse faults, have opposite senses of vergence, but the former is the principal fault. It may be argued that the Base Millstone Grit (light green pick) must continue on the south side of the Weeton Fault, because of the small vertical offset of the Lower Bowland Shale beneath; but this is not the case. Once it is recognised that these high-angle reverse faults probably have a considerable component of strike-slip displacement, the lack of significant vertical offset is understood, and explains the thickening of the Upper Bowland Shale to the south. The mismatch of dips in the Upper Bowland Shale across the Weeton Fault - dipping NW on the NW side, and flat-lying to gently south-dipping on the south - is also explained by invoking lateral slip. Two-dimensional sections cross the fault zones do not balance; this fact requires that lateral displacement be invoked.

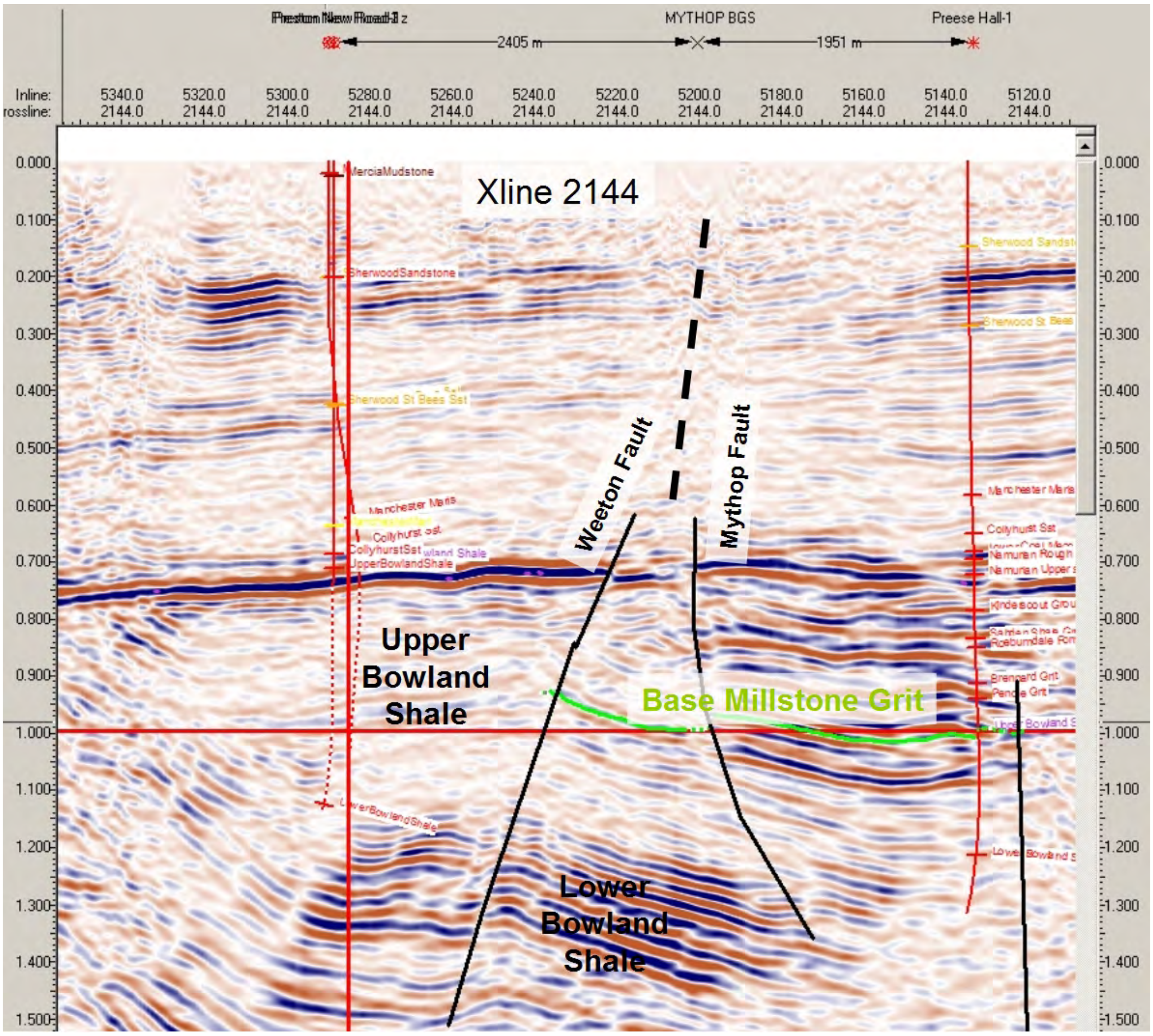

Figure 11. Xline 2144, illustrating the reverse faults 1-2 km north of the heel localities of PNR-1z and PNR-2.

The Weeton Fault and the Mythop Fault both displace the sub-Permian unconformity, the Collyhurst Sandstone and the Manchester Marls in a vertical component of displacement (in addition to presumed but non-measurable strike-slip displacement). This shows that the Manchester Marls cannot act as the supposed cap rock to prevent possible upward migration of fluids from the frack zone situated some 1200 m to the south. Indeed, further west, the horizontal distance from the Weeton Fault to the horizontal section of PNR-2 is about 1 km.

Another significant feature of the two reverse faults is that they converge upwards into a fuzzy seismic reflection zone within the Sherwood Sandstone Group. This is shown by the heavy dashed black line in Figure 11. There is a vertical offset of the Top Sherwood Sandstone of around 50 ms. This shows that the major strike-slip fault zone extends up into the Mercia Mudstone, practically up to outcrop.

None of this information appears to be known to the Operator, unless the Operator knows it but is withholding it from disclosure.

Figure 12 shows the E-W vertical display of Inline 5285, running along the horizontal legs of PNR-1z and PNR-2. The former is shown by the solid red line, the latter by the dashed red line above. The well tops confirm the absence of Millstone Grit, with Upper Bowland Shale directly underlying the pre-Permian unconformity. The reverse fault shown by the solid black line corresponds to the Operator's Fault-1 (aka the PNR Fault). To the east, where the strong lower Bowland Shale reflectors die out rather abruptly, there is a zone of very poor reflection quality. This zone is also apparent in the SE corner of the timeslice shown in Figure 9 above.

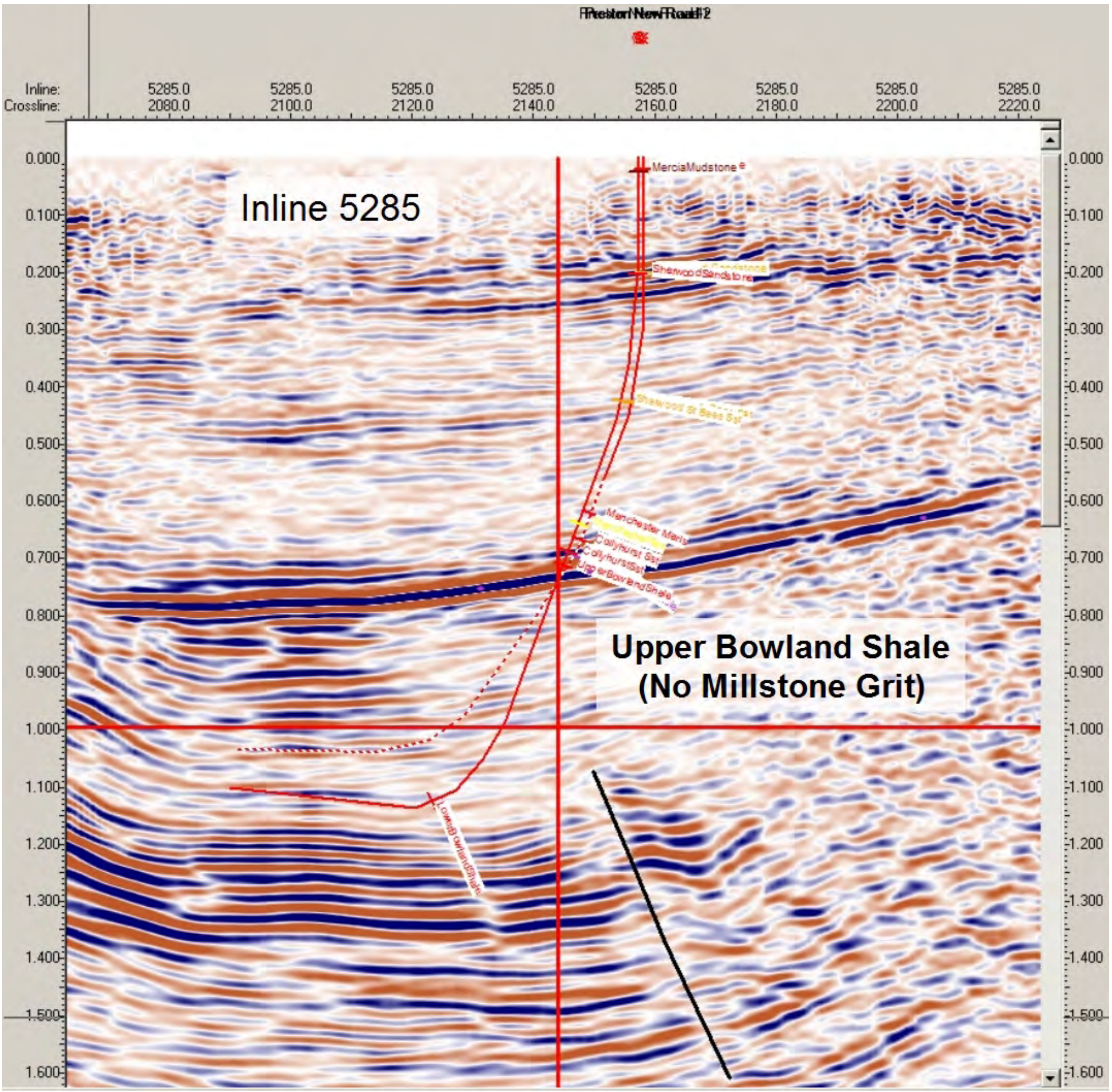

Figure 12. Inline 5285 running along the PNR wells.

I believe that this poor-seismic quality zone is not due to acquisition problems, as is claimed, but is due to very complex low-angle thrust faulting below the Moor Hey reverse fault (shown in Figures 1-4 by the Operator; not marked in Figure 12). This will require meticulous study to unravel.

Discussion

It is apparent that the Operator still misunderstands the rather complex geology of the western Fylde. The prognosed presence of Millstone Grit at PNR is perhaps understandable, but was based on the false assumption that the many mappable faults in the licence area have only vertical displacement, whether in the normal or the reverse sense. The Operator's geologists seem to have underestimated the importance of strike-slip faulting. In fact the 3D survey provides many instances of classic strike-slip geology.

The omission of the Wakepark Fault from the Operator's submissions and publications is a severe error. The breach of the supposed Manchester Marls seal by faulting appears to have been withheld from scrutiny by the regulators - it is difficult to accept that the Operator is unaware that some faults cut the Permian.

The margin of error between prognosed geological layer depths and subsequent results from drilling is typically a few metres up or down. The absence of an entire formation some 300 m thick constitutes a major error in interpretation, which cannot simply be patched up by minor changes.

The many errors, together with the minimal cosmetic changes to the geology made by the Operator late in the day, imply that the Operator is seeking to do the absolute minimum of work in order to satisfy the regulators. This is unacceptable.

Approval of the HFP by the EA

The current version of the HFP for PNR-2 has serious and fundamental shortcomings. It would be unacceptable for the regulators to approve it. Nevertheless, the EA appears to ready to issue its permit, based on the incomplete, erroneous, and at times mendacious data supplied by the Operator in its HFP. A recent leaflet issued by the EA (6 November 2018) states, under the heading 'What is the risk to groundwater or drinking water?':

> "*The EA has fully assessed the risk of operations at Preston New Road before deciding to issue a permit. There is no plausible risk to people's drinking water from the hydraulic fracturing operation. The layer of rock that is being fractured is more than 2 kilometres beneath the surface, and several layers of impermeable rock lie between it and the shallow water table.*
>
> *We have assessed that there is no groundwater in the shale and we do not expect fractures to enter the layer of Millstone Grit above. If fractures were to go beyond the underground permitted boundary (set at the upper boundary of the shale rock) the impact is likely to be materially insignificant. The environmental permit does not allow for any chemicals that are hazardous to groundwater to be used in hydraulic fracturing fluid.*"

Clearly the mention of "*Millstone Grit above*" is untenable, because there is no Millstone Grit either at the well or above the fracking zone.

A recent FOI request to the EA (CL101888HR) states:

> *"Question: Given that they had misinterpreted the section of the vertical well before drilling, what level of confidence do you have in their assertion that 'the seismic shows' that the MG is actually present above the lateral well ?*
>
> *Response: The seismic cross section data at the Preston New Road site originally showed the millstone grit at the vertical part of the well. However, as you say, this was not identified in*

the borehole itself, and the Environment Agency requested a modification to the Hydraulic Fracture Plan (HFP) to take account of this new information. The operator still believes this formation to be present, from the interpretation elsewhere. As a result they have retained the original interpreted level of the top bowland shale where it is above the lateral to be hydraulically fractured. This interpretation has been the subject of discussion between the Oil and Gas Authority (OGA), ourselves, and the British Geological Survey.

We have accepted the interpretation in the HFP on the basis that the alternative is that the top bowland shale horizon corresponds to a higher reflection event, and as this horizon defines the upper permit boundary, the current interpretation of a lower horizon is a safe one."

So the EA accepts the scanty diagrams and explanation given in the current HFP, based on vague assurances ("*the operator still believes ...*") that it has also discussed the matter with other agencies (OGA and BGS). This complacent attitude is inconsistent with sound regulation.

The same FOI request yields the following exchange:

[Query 4] "Have you helped with all these interpretations or have Cuadrilla taken extra advice maybe ? What confidence do you have in their understanding of the seismic survey ?

Response: We request enough information about the 3D seismic results to understand the quality, reliability and fault resolution capability of the cross-sections extracted, and challenge these aspects if appropriate. At this site, the final data quality was not the best, due to site factors. We do not assist in the original interpretation or re-interpret the operator's data volume, but in this case the 3D dataset has been reviewed by the OGA. Please note that a complete reinterpretation of such a 3D seismic data volume could potentially take weeks, and may involve an element of re-processing."

The response above shows that the EA seems to be more concerned about saving the Operator some time and money, than about actual regulation. The Operator has had some nine months since the results of PNR-1 came in, and therefore should have had ample time to fully re-interpret the 3D survey. Instead, the Operator seems to have relied on superficial tinkering with a couple of horizons in the vicinity of the PNR wells. As shown above, these re-interpretations are contradicted by the seismic data.

Lastly, the EA has responded to the FOI query about the multiple changes to the geology around the Moor Hey Fault (see Figures 2-4 above):

"Question: Given that Well 2 does not go anywhere near the Moor Heys fault, how /why have Cuadrilla changed the interpretation?

Response: As the area around the Moor Hey fault is not relevant to the hydraulic fracturing of PNR1z and PNR2 laterals, we have not challenged the interpretation of this area as part of the HFP assessment."

This demonstrates again that the EA is taking a very narrow view of its regulatory remit. It is unacceptable that major changes in the geology within one or two kilometres of the well are deemed to be "*not relevant*".

The EA has written off all the groundwater in the Sherwood Sandstone Group below the Fylde west of the Woodsfold Fault as being saline, static, and therefore not of concern should it be contaminated by drilling and fracking activities. I published a detailed discussion of the groundwater in the western Fylde, in the context of a critique of the late Professor Paul Younger's expertise in hydrogeology. I attach extracts from this article as Appendix A below.

The principal points I made were:

- The EA is wrong to assume that the Woodsfold Fault is a non-transmissive boundary. It cites the modelling studies of 1997 and 2009, in which the fault was *defined* as a boundary, *a priori*. Therefore it cannot then take the modelling results as proof that the fault is a barrier to flow.

- The Kirkham borehole, which encountered hypersaline water, penetrated salt horizons within the Mercia Mudstone Group. Use of this anomalous result cannot then be applied to the whole of the western Fylde. The same applies to the recent deep borehole drilled by the BGS near Roseacre Wood.

- The EA is unaware of the implications of two old boreholes which penetrated to the Sherwood Sandstone, and which presumably drew fresh water.

- The EA has never sampled, nor considered the implications of, the water in the Wakepark lake.

- The Wakepark Fault, if acting as a conduit for upward flow from the confined Sherwood Sandstone aquifer, completes discharge end of the cycle of recharge-discharge, which was missing from the EA's investigations and used as an argument for no flow.

Conclusions

The concerns that I have raised above need to be addressed as a matter of urgency by the OGA and the EA.

There exist pathways from the neighbourhood of the frack zone upwards to the Millstone Grit and Sherwood Sandstone Group, and even *via* faults through the Mercia Mudstone Group to the surface. It is the primary responsibility of the EA to see that these secondary and primary aquifers are protected. The EA cannot and should not rely on the incomplete and sub-standard information supplied by the Operator.

The Operator should be required to undertake a wholesale remapping and submit a revised HFP before approval is granted to proceed with fracking of PNR-2. I would expect the new plan to include many more samples of interpreted seismic data. It must also encompass a wider area than just the narrow zone above the horizontal wells. Structure contour maps of various horizons need to be included, including the Permian and Triassic horizons.

Appendix A
Extracts from Frackland blog:
'The expertise of Professor Paul Younger – Part 3. Hydrogeology of fracking (Part B)'
published by David Smythe on 3 February 2017

Introduction

... The scientific issue here is whether or not the Sherwood Sandstone Group aquifer below the salt-bearing Mercia Mudstone Group in the Fylde west of the Woodsfold Fault (where Cuadrilla's drilling and fracking activities are focussed) is saline or not.

Citation of previous research

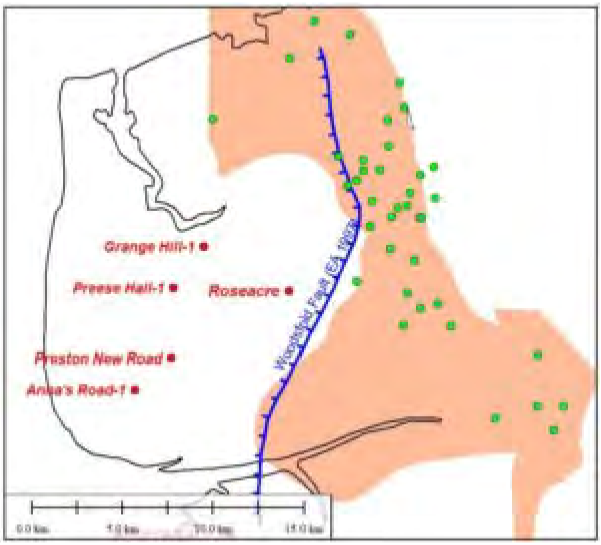

... Fig. 1. Hydrogeology of the Fylde. The tan colour is the major Sherwood Sandstone aquifer at the surface. All the available hydrogeology studies concern the aquifer east of the major Woodsfold Fault (blue toothed line), where the water boreholes that were studied are situated (green dots). None of these studies have any bearing on the hydrogeology of the western Fylde, where Cuadrilla has drilled and proposes to drill (red dots).

But all nine of the citations that Professor Younger accuses me of neglecting are either irrelevant and/or outdated. None of the cited work has any bearing on the hydrogeology of the western Fylde, west of the Woodsfold Fault - the area of interest where Cuadrilla wishes to frack (Fig. 1). They all concern the major aquifer at outcrop (i.e. at the surface), some 10-20 km to the east.

... I have disputed the EA's claim that groundwater here is saline, and that it is therefore is not of concern if it were to be contaminated by fracking. ...

Western Fylde borehole evidence

Kirkham borehole (type E)

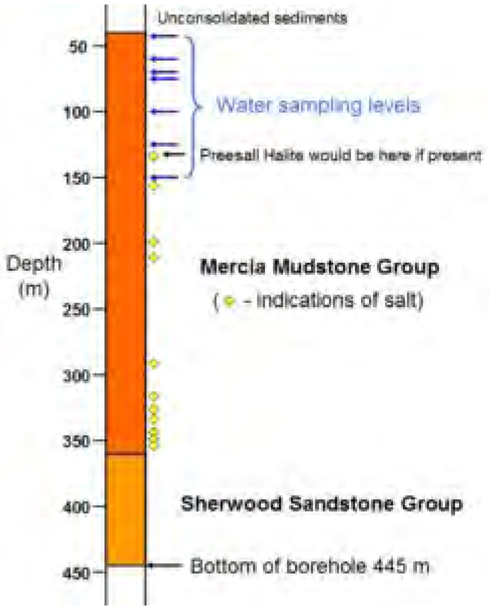

Fig. 6. Simplified geological log of the Kirkham borehole, western Fylde. Groundwater sampling levels measured by the EA are shown by blue arrows. Evidence of halite is shown by yellow diamonds. If the Preesall Halite, found further NW, were present, it would be at the level shown by the black arrow.

This borehole was drilled in 1970 as a test for underground gas storage, but has since then been used as an observation borehole. It penetrated the Sherwood at 366 m and went on down to 445 m.

The only evidence that the EA seems to consider, in dismissing the groundwater potential of the entire western Fylde, is the hypersalinity of the water samples observed in this borehole. I pointed out that this evidence

is invalid because two of the three hypersaline samples were taken from levels within the Mercia Mudstones, where the observed hypersalinity, some two or three times more salty than seawater, can be explained by perched (hydrogeologically isolated) relict halites known to exist within the Mercia Mudstones.

Rowe's Model Dairies, Inskip (type E)

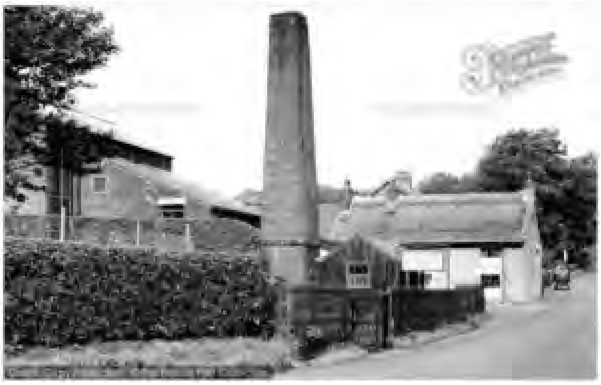

Fig. 7. Rowes Model Dairies, Inskip, c. 1950, where a 161 m deep borehole penetrated the Sherwood Sandstone aquifer at 124 m. Photo credit: Francis Frith Collection.

This borehole was drilled in 1940-41. It lies 6 km NNE of the Kirkham borehole, and, like it, is just west of the Woodsfold Fault. It is 2.7 km NE of Cuadrilla's Roseacre Wood site. It supplied a dairy and cheese factory untill the mid 1960s. Would Professor Younger have us believe that saline water was used here?

Phoenix Mill, Wesham (type E)

This borehole was drilled to a depth of 445 m in the nineteenth century. It lies 1.7 km west of the Kirkham borehole and 4.2 km east of Cuadrilla's Preston New Road site. No geological details are available, but this depth appears to be through the Mercia Mudstone Group and well into the Sherwood.

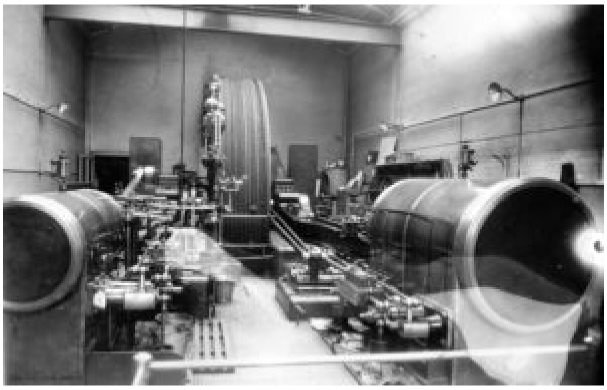

Fig. 8. Compound steam engine of the type used at Phoenix Mill in the nineteenth century. Consumption of water, which would necessarily have been fresh, was about 40,000 l per day, of which most could be recycled.

The water would have been required chiefly for the compound steam engine to run the weaving looms. The water would have re-used in a closed cycle, with a reservoir on-site. The existence of the reservoir has been confirmed from a 1911 map. Mr John Phillp of the Northern Mill Engine Society says that such an engine would require about 40,000 litres per day, but that most of this would be recycled. However, it is unlikely that a source of salty water would have been countenanced, because it would lead to precipitation and corrosion in the boiler, with resulting inefficiency.

Fylde discussion

The boreholes described above are part of a group which I examined, comprising some 39 relevant wells west of the Woodsfold Fault, of depth greater than 30 m, which are available on the BGS borehole mapper website. About five of these borehole records are confidential, and/or there is no information. In addition, I studied the water composition records of 56 boreholes, which I obtained from the EA.

I think it is reasonable to conclude that it is unlikely that hypersaline or even saline (undrinkable) water was used either for the cheesemaking at the dairy or for powering the looms at the mill.

The EA believes, based on no solid evidence, that the flow across the Woodsfold Fault will be low. Next, it assumes that there will be little or no vertical flow – but this assumption ignores the presence of faults cutting the Mercia Mudstones. These could be transmissive pathways, particularly when one considers the stress regime in the uppermost 300 m below ground level.

The EA cannot find a discharge for the flow, if present, but this again ignores the presence of faults. Lastly, since the Woodsfold Fault is *defined* as a no-flow boundary, the lack of westward flow in the model cannot be used as an argument to prove that there is no westward flow.

Since I published the paper in January 2016, and taken account the ensuing commentaries on it, I have done a little more research on the possible flow pattern of the groundwater under the western Fylde. I have obtained the 2D seismic data for the area shown in Figure 7, and mapped a newly-recognised fault, which I call the Wakepark Fault, running (coincidentally) from the Cuadrilla wells Preese Hall-1 to Anna's Road-1. In fact the fault has been previously recognised at depth, both independently by an oil company in 1994 and more recently by the BGS, but has not previously traced up through the Mercia Mudstones to the surface. It does not appear on the current BGS paper or digital geology maps.

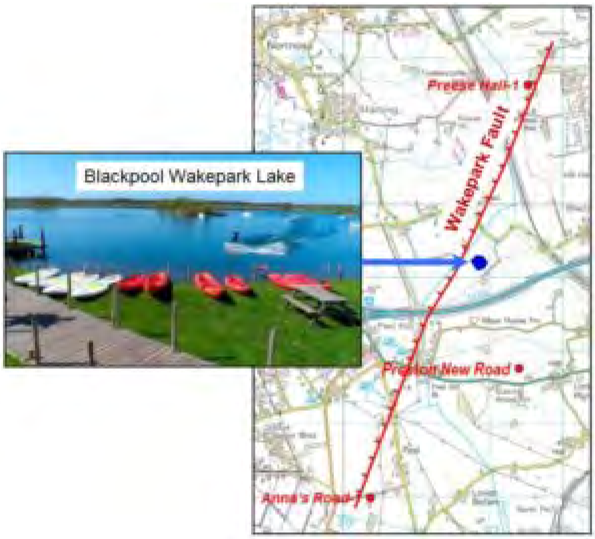

Figure 9. Blackpool Wakepark recreational lake is fed by four pure freshwater springs. These could originate in the postglacial sandy deposits in the top 30 m of the subsurface (unlikely) or else could be fed by flow up the Wakepark Fault (red toothed line). Use of copyright OS digital data acknowledged. Photo credit: Ream Hills Holiday Park.

The Blackpool Wakepark Lake is an artificial lake created from boggy ground (Figure 9), and replenished by four springs. Now, it is possible that the springs supplying this lake are recharged from the unconsolidated sandy post-glacial deposits in the top 30 m of the subsurface, but the volume of flow from such a source would seem to be far too small. The more likely alternative, in my view, is that they are fed by upflow along the Wakepark Fault from the Sherwood. This newly identified discharge is the missing link in the flow cycle, originating as recharge in the Bowland Fells, that the EA failed to find.

A spokesperson at the Ream Hills Holiday Park told me that the lake water is tested annually, and is of such good quality that it could be bottled and sold as spring water. So this is not a resource that should be lightly written off, as the EA and Professor Younger conclude.

Conclusions

My concern that the EA has written off a past and future potential groundwater resource in the Fylde is justified. Before any unconventional exploitation begins it would be prudent for the EA and/or the BGS to sample the water at SSG levels.

...

MEETING BETWEEN BALCOMBE PARISH COUNCIL

& CUADRILLA RESOURCES LTD

at MSDC Offices on Friday 3 May at 3.30 pm

PRESENT: From Balcombe Parish Council – Alison Stevenson (Chairman), Rodney Saunders (Vice Chairman), Robin Williamson (Responsible Financial Officer) and Rosemary Robertson (Parish Clerk)

From Cuadrilla Resources Ltd – Francis Egan (CEO) and Matt Lambert (Government & Public Affairs Director)

Balcombe Parish Council (BPC) opened the meeting by confirming that all information provided by Cuadrilla Resources Ltd (Cuadrilla) would enter the public domain.

Cuadrilla stated that they had requested the meeting in order to inform BPC of their plans for the site at Lower Stumble. Following the grant of an Exploration Licence, in 2010 they had received Planning Permission for an exploratory well on the site, and this expires in September 2013.

Commencing in mid June they intend to drill, **but not to hydraulically fracture (Frack)**, an exploratory well on the site. The well will be drilled to a vertical depth of about 3,000 ft, and thereafter horizontally (at a depth of about 2,500 ft) in a direction between west and northwest (under land owned by Balcombe Estate) for a distance of about 2,000 ft. They will then extract samples of the rock. They will "stimulate" the reservoir rock using low pressure hydrochloric acid in a concentration between 7.5 and 15% that they say is classified as non-hazardous. They will be looking for oil within the layer of micrite, and assessing the potential flow rate to enable them to decide on a subsequent course of action.

BPC asked for, and was given, an unequivocal assurance that current plans do not involve any Fracking on the site. Cuadrilla advised that the necessary applications had been submitted to the Environment Agency and the Health & Safety Executive, and that on the day of the meeting an application for drilling consent was being submitted to the Department of Energy & Climate Change (DECC). These applications do not include a proposal to Frack the well.

BPC asked how long it would take to decide a course of action once the data from the exploratory well had been obtained. Cuadrilla said that there would be a quick decision and that there would only be three options:-

1. If there was negligible oil flow the well would be capped with cement and abandoned.
2. If there was sufficient natural flow an assessment would be made of how much oil might be extracted without Fracking and if so whether and where further exploration wells might be required to assess what quantity of oil might be recovered from the rock.
3. If there was insufficient natural flow consideration would be given to whether or not the reservoir rock could be safely fracked and if so the necessary environmental impact assessment and planning permissions might be sought to Frack and flow test the well (this would be a separate and future operation if approvals were sought and granted).

Options 2 and 3 would require a complete new application process, including new Planning Permission and a full Environmental Impact Assessment for option 3, and would take a substantial amount of time.

Cuadrilla informed BPC that they plan to monitor water quality by drilling a very small bore about 200 ft deep into the aquifer from which they will obtain water samples before and during the main activity. Specific approval is not required for this exercise which is expected to be done during May using a very small rig. Water samples will be analysed by an independent company, Ground-Gas Solutions and Cuadrilla will make the data on water quality public.

BPC pointed out that the stream that ran alongside the site should also be monitored, because Southern Water had used it to “over pump” water into Ardingly Reservoir, although the aquifer did not, as far as BPC are aware, feed the Reservoir. BPC requested that the monitoring should include this stream, both above and below the site.

BPC raised the concerns of some villagers over air pollution. Cuadrilla said that they would look into the possibility of air quality monitoring.

Diesel fuel, drilling “mud” and other fluids stored on site would be held within secondary containment in the bunded area.

Delivery and erection of the rig will take one week, drilling is expected to take 40 days (working 24 hours per day, 7 days per week) following which dismantling and removal of the rig will take a further week. The rig to be employed is smaller that that used on their Lancashire site, and the sound caused by the drilling process should not exceed 45 decibels. Lighting from the site would be visible from some houses but the positioning and direction of lights would be designed to minimise this.

Discussion then moved to traffic implications for the proposed work. For the delivery and removal of the rig, each taking one week, West Sussex County Council has given permission for the London Road through the village to be used and Cuadrilla have given assurances that the lorries would not travel past the School during drop-off or pick-up times. There would be approximately 2 vehicles per hour between 9.30 am – 2.30 pm and between 4.30pm – 6.00 pm. BPC pointed out that after school clubs meant a lot of activity at the School until at least 4.15 pm and Cuadrilla undertook to respect this. There would be around 60 vehicles (HGV) during each of the weeks for delivery and removal of the rig but only 2 per day while drilling was being carried out. Security on site is being provided by G4S.

When asked about the likelihood of gas being found on the site Cuadrilla stated that there could be some gas but this was unlikely. They are expecting to find oil. Any gas would probably be flared during exploration.

BPC criticised Cuadrilla for using a Market Research Company that had conducted a survey in the village and had been disingenuous with parishioners about who had commissioned them and the purpose of the survey. Cuadrilla said if this happened this was against the instructions given to surveyors and gave assurances that they would not undertake another survey in the village without notifying the BPC.

Cuadrilla was asked if they had approached Balcombe Estate about an extension to their lease of the site; apparently discussions have taken place.

BPC asked if its Fracking Report had been an accurate representation of issues associated with the site and was told that it was a comprehensive and informed document.

BPC reminded Cuadrilla that, following the poll of residents, it is committed to opposing Fracking. BPC was not sure what the residents' views on exploration without Fracking would be.

Cuadrilla is due to meet with Head of Planning at WSCC. They had also spoken to Francis Maude in his capacity as the local MP as a courtesy.

Cuadrilla went on to give information about other locations in which they are operating. They currently have a number of sites in Lancashire and sites in Holland and Poland, but nowhere else in the UK. BPC expressed the wish that Balcombe should not be "industrialised," it is a beautiful village surrounded by one of the largest shooting estates in the South. Cuadrilla insisted that industrialising the area forms no part of their plans. Nevertheless they are a commercial enterprise and this exploration could be beneficial to the UK. Cuadrilla explained that full exploitation of a gas or oil field may not be possible due to conditions or constraints above ground.

Cuadrilla stated that they wanted to open a dialogue with Balcombe residents. They were planning to write to every house-hold and to organise a Drop-In session at which residents could put questions on matters that concern them. Cuadrilla wanted an opportunity to tackle some of the scare stories that had been promulgated as a result of "shoddy practice" in the USA. They emphasised that they wanted to co-operate with local elected officials, but understood that BPC is committed to supporting the majority view of residents. They would consider organising pre-booked visits to the site during operations subject to health and safety constraints.

BPC welcomed Cuadrilla's proposal to letter drop and to hold a Drop-In meeting in the village and would encourage villagers to attend. Thursday 23 May, 4.00 pm – 8.30 pm, was pencilled in as a possible date for such an event.

The meeting closed with Cuadrilla undertaking to keep BPC informed of any future plans. They said that they are open to questions at any time.

Minutes of Meeting

Date: 3 August 2011 / 11.00am
Venue: Stirling
Site: Broadmeadows
Persons Present:
SEPA

Enzygo

Greenpark Energy

Agenda distributed

Introductions

Purpose of meeting to explore why SEPA considered boreholes to be non-compliant with GBR3 and to examine ways of progressing current application.

explained that SEPA had previously stated, during the Mouldyhills application, that one of the principal concerns was the borehole construction – this had been accepted by all parties during those discussions.

explained that non-compliance due to:

- Inadequate construction of existing boreholes (lack of cement behind casing)
- Possibility of waters of different chemical composition cross contaminating

raised uncertainties regarding the SEPA interpretation of GBR3. Interpretation not supported by wording of regulations. Legal Opinion supports view.

disagreed with interpretation and maintained SEPA position similarly supported by legal interpretation. Group discussed at length the option of exchanging legal interpretations.

General acceptance that preoccupation with legal interpretations would be non-productive and that way forward would be to find a mechanism for providing SEPA with level of confidence to accept borehole construction methodology and thereafter favourably determine current discharge application.

offered re-assurance to SEPA that new boreholes proposed at Mouldyhills West and all other future sites will be cemented from TD to surface.

Generally accepted that as GBR3 no longer driving mechanism, area of concern should be protection of Permian aquifer from saline intrusion from underlying Middle Coal Measures. supported by suggested an HRA which would identify source/pathway/receptor for contamination. This would thereafter inform as to level and nature of remedial works required. This accepted by all.

clarified that SEPA's issues are twofold. HRA's target objectives would be to address existing risks AS WELL AS consider future risks as part of discharge licence activities.

Issue of monitoring wells as part of the above raised by . Level of monitoring would be determined based on the outcomes of the HRA. Any monitoring would need to be appropriate to the sites circumstances (location, nearby sensitive receptors, etc), and would be dependent on the potential risks. Remedial works would involve injection of cement into annulus behind steel casing. indicated that base of Permian strata at ~210m based on information supplied within the submitted application. HRA would inform depth to which cement should be injected. Agreed that if not possible to inject cement then this would be indicative of an existing sub-strata/casing seal and that this would be acceptable as a means of demonstrating lack of pathway for contaminants. agreed that a draft Licence could be issued with development conditional upon the submission and acceptance of a satisfactory HRA and remediation works.

Next steps agreed:

HRA methodology/decision tree to be submitted to by for approval prior to any work being commenced.
On agreement of the above, HRA top be carried out by and remediation works proposed for approval by SEPA
to issue Licence with appropriate conditions precedent. Progress on HRA and draft licence to run in parallel.

requested copies of interpretative reports for Greenpark wells at Broadmeadows.

requested that those Broadmeadows boreholes not used for development be plugged and abandoned thereby addressing any shortcomings in engineering/construction. agreed.

advised SEPA preference would be for P&A to take place prior to fraccing operations of remaining wells.

raised issue of installation of monitoring boreholes at all future sites. Much discussion re this point. Agreed that monitoring boreholes would be installed where circumstances dictated that groundwater monitoring necessary to safeguard a sensitive receptor, eg River Esk or abstraction borehole.

asked if any other issues to be addressed. confirmed that there were none.

Professor R C Selley DSc, DIC, FGS
Consulting Geologist
P O Box 425
Dorking RH5 4WA
UNITED KINGDOM

THE GEOLOGICAL SOCIETY
CGeol
CHARTERED GEOLOGIST
Fellow No. 8962

Phone: +44 (0)1737 841585 Mob.: +44 (0)7714490725 Email: r.selley@btinternet.com

3 July 2014

REPORT ON PLANNING APPLICATION
Reference SDNP/13/05896/CM)

I have been asked in a letter to me from Mrs L Harding dated 26th June of the South Downs National Park Authority to comment on the application by Celtique Energy Weald Ltd. to drill a petroleum exploration well at Nine Acre Copse, Fernhurst.

I have been specifically asked to address the issues below. My comments are in italic font coloured blue.

Subject: Provision of petroleum geology advice to the South Downs National Park Authority

In December 2013, the South Downs National Park Authority (the Authority) received a planning application (reference SDNP/13/05896/CM) for the development of a temporary exploratory oil and gas well 'Fernhurst-1' at Nine Acre Copse, Fernhurst. The applicant (Celtique Energie Weald Ltd) has also specified that this is not a 'fracking' application. I have carried out a full ES Review, however the Authority would welcome further technical advice with regards to the geology of the Weald Basin and how it relates to this planning application.

<u>Work brief:</u>

We would like you to carry out a critical review of the geological information submitted by the applicant. This consists of:

1. The original planning application submission documentation (environmental statement included); and
2. The further information received in May 2014.

A key difference between the two, is that the further information submission amends the application by removing the horizontal drilling element, the reason for this is not clear.

I have asked Celtique's CEO, Mr Geoff Davies, for further clarification on this amendment. He replied that such queries should come direct from the SDNPA. Celtique's website states that they are not pursuing with the application for a horizontal well to save time and money. It is possible that the issue of trespass by underground drilling may have been a further factor in reaching this decision. Until this matter is resolved it might cause further delay in the application being processed and a decision reached. Recent pronouncements by Michael Fallon, the Minister for Energy & Climate Change, suggest that the issue of trespass may be resolved speedily to allow horizontal drilling below a depth of 300m to not require the permission of the landowner.

As part of the critical review, we would like to understand:

a) The consistency, accuracy and level of completeness of the geological information submitted. Is the information submitted with the application consistent with other sources of geological data/research for the Weald Basin?

The geological information of the application is consistent, accurate and sufficiently complete. In particular it is consistent with the definitive published report on the Weald by:

Andrews IJ 2014. The Jurassic Shales of the Weald Basin: geology and oil & gas resource estimates. British Geological a Survey for the Department of Energy & Climate Change. London 79pp.

Swan, G & Munns J. 2003. The Hydrocarbon Prospectivity of Britain's Onshore Basins. Department of Trade & industry. London. 81pp.

The Fernhurst well must regarded as high risk. The reservoir characteristics of the Kimmeridge limestones are unknown. Furthermore the BGS study suggests that only a small thickness of the Kimmeridge Clay source rock may have been heated sufficiently to generate oil.

b) To understand the target formations within PEDL 231 and how they compare to those outside the PEDL boundary, but within the Weald Basin. Can the same geology be found outside PEDL 231?

*The general geology of the Weald is very well known after nearly 200 years of research. The surface geology has been mapped both by the British Geological Survey, by academic and amateur geologists **(Figure 1.).***

*The subsurface geology of the Weald is less well known, obviously, though many wells have been drilled across the basin since the Netherfield wells in 1875. The Weald is a basin of sedimentary rocks (limestones, sandstones & mud rocks) deposited layer on layer over many millions of years. Surface mapping and subsurface boreholes show that the main rock units are laterally continuous across the Weald **(Figure 2)** though there are some minor changes in rock type across the basin.*

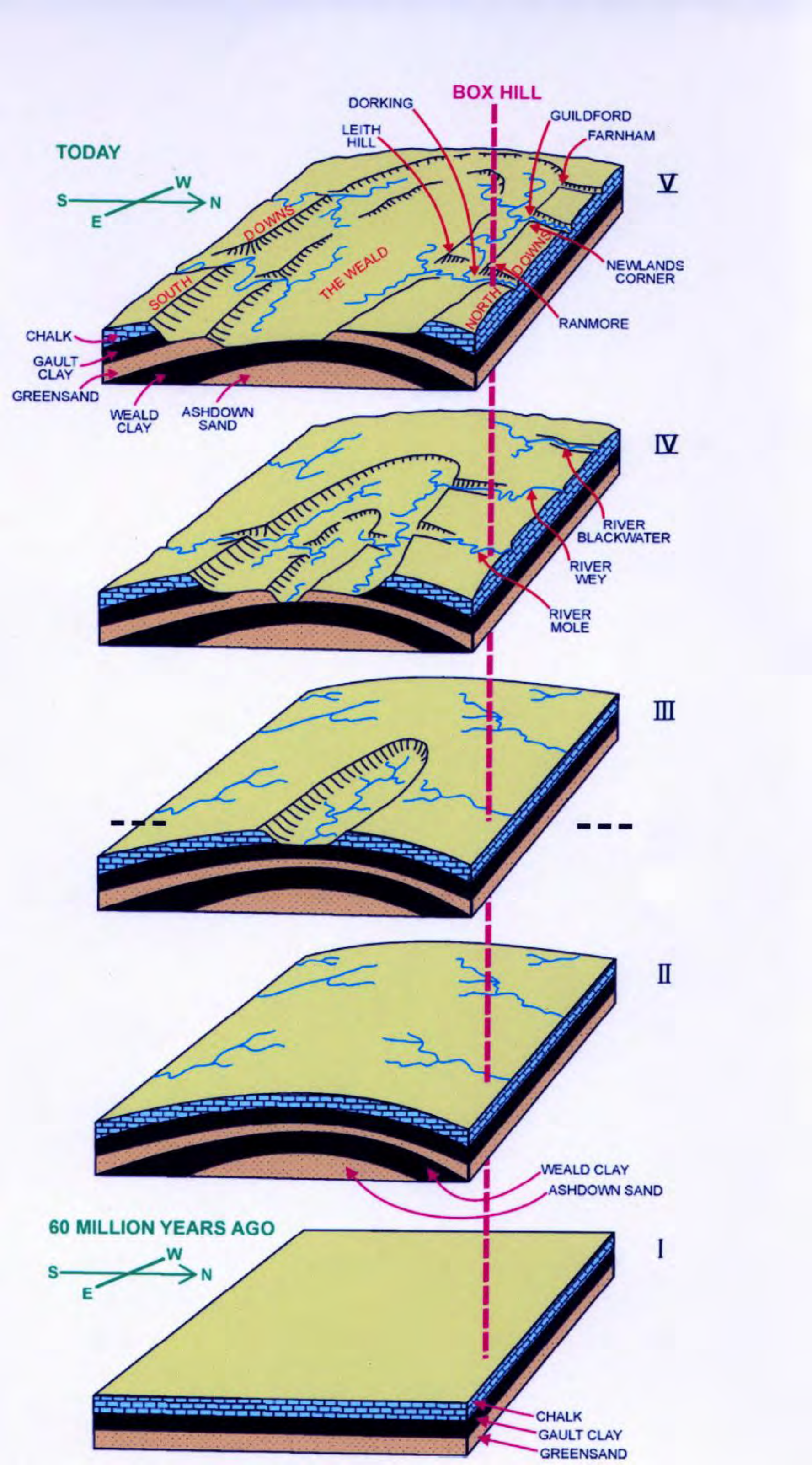

FIGURE 1. Diagrams to illustrate the surface geology & evolution of the landscape of the Weald. From Selley, R.C. 2006. The Box H ill & Mole Valley Book of Geology. Published by the Friends of Box Hill. Dorking. 35PP.

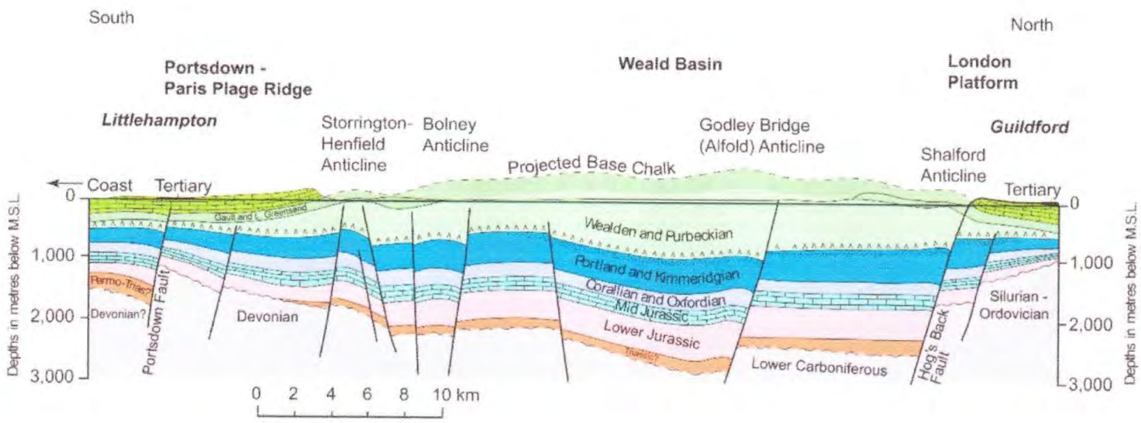

***Figure 2**. South to North cross-section of the Weald basin showing the continuity of the various rock strata. From Butler M. & Pullan C.P. 1990. Tertiary structures and hydrocarbon entrapment in the Weald basin of southern England. In: Hardman, RFP & Brooks J (Ads.) Sp. Pub. Geol Soc Lond. 55. 371-391.*

***Figure 3** shows the producing oil & gas fields of the Weald that provide information of known source rocks and reservoirs, and can be used to predict their distribution. Some variations in source rock and reservoir quality have been observed.*

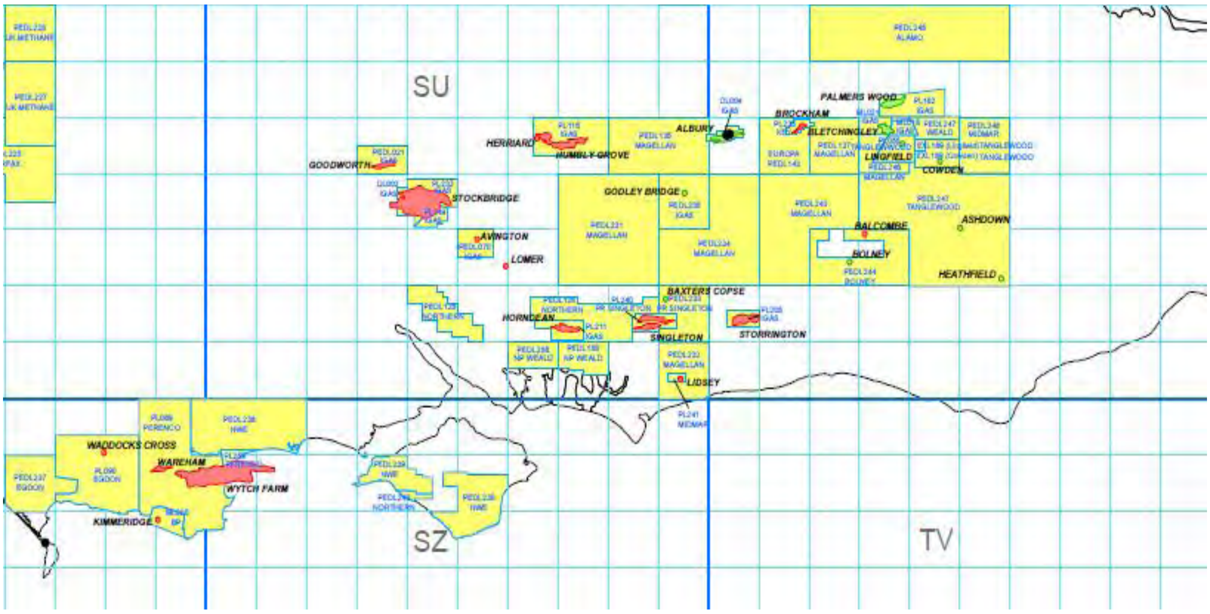

Figure 3. Map showing the location of Wealden oil & gas fields that provide evidence of reservoirs and their continuity © Department of Trade & Industry.

c) Some of the basics behind petroleum geology and terminology (e.g. Is the Weald Basin a ‘prospect’ and if not, what is the difference between a ‘prospect’ and a ‘target horizon’?)

***Figure 4** is a stratigraphic column that shows the sequence of rocks to be encountered across the Weald. This figure also shows the rock strata which are petroleum reservoirs for the various oil & gas fields of the basin. In the petroleum industry the term ‘**prospect’** is applied to a location where it is possible that petroleum may be trapped and that merits testing by drilling. The term ‘**prospective**’ is applied to an area where evidence suggests that petroleum may be trapped.*

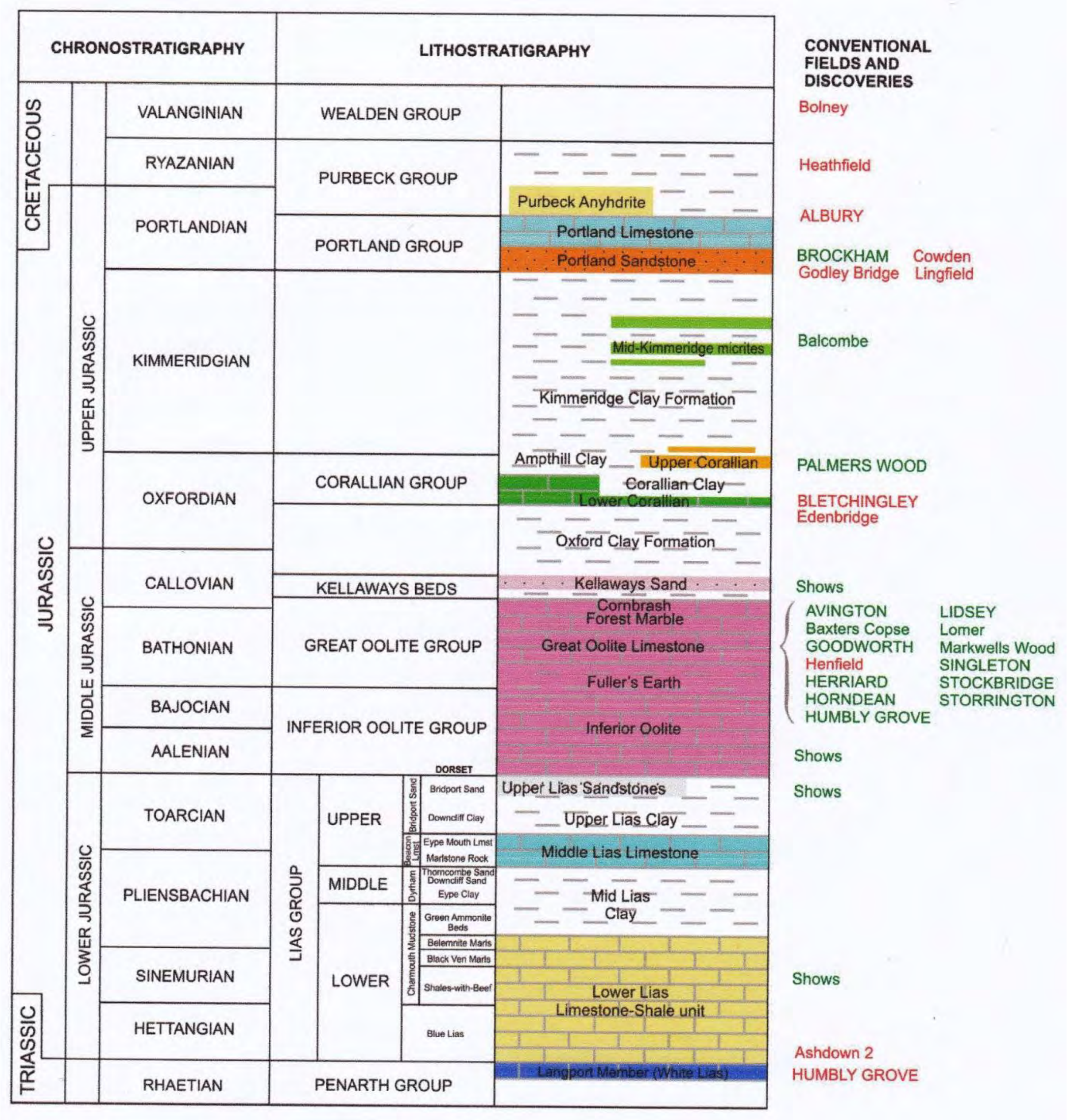

FIGURE 4 Summary of the sequence of rock of the Weald basin showing the main petroleum reservoirs. From: Andrews. 2014. © Department of Industry & Climate Change.

*Thus the Weald basin is known to be **prospectiv**e because it contains many producing oil and gas fields. A **prospect** has been identified beneath Nine Acre Copse that merits drilling.*

*A **target** horizon is a layer of rock, generally sedimentary (sandstone or limestone), that may contain petroleum, i.e. has the potential to be a petroleum reservoir, i.e. has porosity (pore spaces to hold petroleum) and permeability- the pores are interconnected, enabling petroleum and water to flow through the rock. Thus there are two **targets** for the Nine Acre Copse **prospect**: the Kimmeridge Limestones (Upper Jurassic) and the Great Oolite (Middle Jurassic).*

The Great Oolite is a major petroleum reservoir across the Weald basin producing oil in many fields such as Singleton, Storrington and Lydsey. The Kimmeridge Limestone target is not yet a proven reservoir in the Wessex Basin. This was the target for Cuadrilla's well at Balcombe.

d) To understand the porosity of the primary target; Kimmeridge Limestone formation and the secondary target; Great Oolite formation and the likelihood of whether the oil would be free flowing or trapped and require fraccing.

*The **Kimmeridge** Clay is a thick formation that is largely composed of mud that contains within it thin layers of fine-grained limestone. The Kimmeridge Clay has a high content of organic matter. This has generated petroleum in some areas, notably the North Sea, where it is the main source rock for North Sea petroleum. Within the Weald basin the thin limestone beds are particularly well –developed forming the rock unit that is termed the Kimmeridge Limestone. It must be understood that this is not a bed of solid limestone many metres thick but a sequence of thinly inter-bedded limestones and clays. The limestones have negligible porosity and permeability, but they are brittle and naturally fractured due to earth movements. Petroleum can migrate from the organic rich clay beds into the intervening naturally fractured limestones. Colloquially Americans call this the 'Oreo cooky' model (An oreo cooky is a biscuit composed of alternate layers of crisp biscuit and viscous sugary confection) **Figure 5.** This situation produces petroleum in the Franciscan cherts of California and the Bakken shale of Illinois. When the shale gas industry began in 1821 production was solely from natural fractures. The artificial fracturing of conventional reservoirs (sandstones and limestones) began in the late 19th century. For many decades explosives were used, more recently the less exciting and more environmentally friendly hydraulic fracturing was developed in the 1940's, first for conventional reservoirs and then for shale in the late 20th century.*

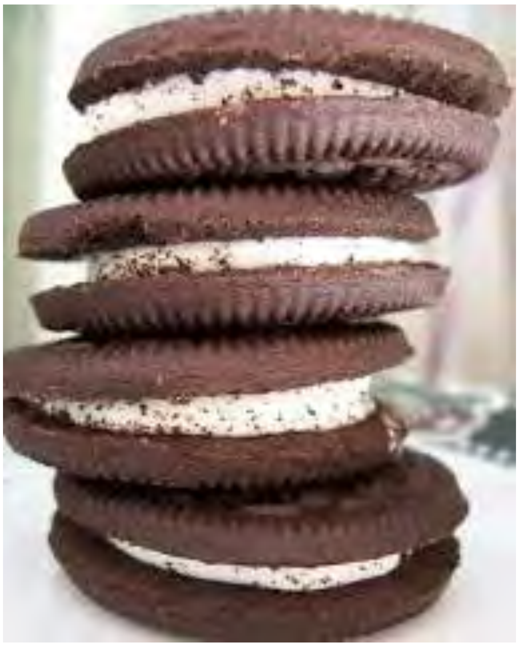

***FIGURE 5**. Upper: Photo of a vertically arranged pile of Oreo™ Cookies showing interbedded brittle biscuit and unctuous cream.*

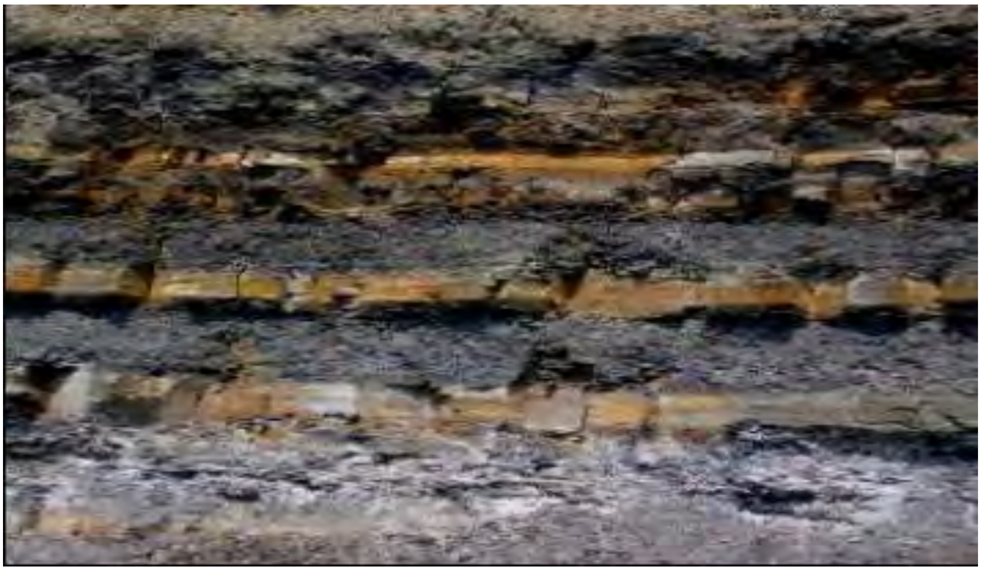

***FIGURE 5**. Lower. Outcrop of Kimmeridge Clay showing alternation of soft grey shale (source rock), and brittle fractured limestone (Reservoir –hopefully).*

*The **Great Oolite** is a limestone with good porosity and permeability. It is a type of rock that is a major petroleum reservoir around the world and in Arabia in particular. In some cases the carbonate grains are completely un-cemented, but generally there is some mineral cement that infills pores and thus diminishes both porosity and permeability. In such cases the rock is hydraulically fractured and flushed through with acid to leach out some of the carbonate and thus increase flow rate. The Great Oolite is a major producer of petroleum across the Weald basin in the Storrington, Singleton, Humbly Grove, Stockbridge, Baxter's Copse, Lidsey and Goodworth fields (Refer back to Figure 4).*

- **END** -

CONFLICT OF INTEREST STATEMENT

I am not currently, nor have been, nor expect to be involved with Celtique Energy and/or its partners as a consultant or shareholder. I have, in the past, consulted for several companies exploring for petroleum in the Weald and across the whole UK. I have no consulting arrangements in the Weald at the present time.

Richard C Selley
3 July 2014

Planning application no. SDNP/1305896/CM by Celtique Energie

to drill at Fernhurst, West Sussex:

Comments upon report to SDNPA by Prof. R. C. Selley

By

David K. Smythe

Emeritus Professor of Geophysics, University of Glasgow

La Fontenille, 1, rue du Couchant, 11120 Ventenac en Minervois, France

8 August 2014

Introduction

Professor Selley has supplied a report to the SDNPA dated 3 July 2014, in response to a request from the SDNPA dated 24 June 2014 (not 26 June 2014, as he states). I wish to comment on a number of contentious points and factual errors made by Prof. Selley, and note, for the record as well as for consideration by the appropriate Planning Committee, the issues upon which he did not respond, or responded in an incomplete manner.

In separate numbered sections, I quote from the report by Prof. Selley, in which he has first re-stated an extract from the SDNPA letter (upright black text), and then followed it by his response in *blue italics*. These pairs of extracts are indented and placed between quotation marks. My comments are in green.

Summary

Prof. Selley is complacent and uncritical regarding the completeness of the information supplied by the Applicant. He has failed to answer the question about whether the same geology can be found outside the licence area, choosing instead to offer some irrelevant information. He is factually in error regarding the Applicant's targets. He is also inaccurate regarding the nature of the Kimmeridgian limestones, and again has failed to answer a specific question asked of him - whether or not the limestones will require fracking.

1. Horizontal well (Selley pages 1-2)

"A key difference between the two, is that the further information submission amends the application by removing the horizontal drilling element, the reason for this is not clear.

I have asked Celtique's CEO, Mr Geoff Davies, for further clarification on this amendment. He replied that such queries should come direct from the SDNPA. Celtique's website states that they are not pursuing with the application for a horizontal well to save time and money. It is possible that the issue of trespass by underground drilling may have been a further factor in reaching this decision. Until this matter is resolved it might cause further delay in the application being processed and a decision reached. Recent pronouncements by Michael Fallon, the Minister for Energy & Climate Change, suggest that the issue of trespass may be resolved speedily to allow horizontal drilling below a depth of 300m to not require the permission of the landowner."

The SDNPA should therefore ask Celtique directly why the horizontal well was dropped.

2. Consistency, accuracy and level of completeness (Selley page 2)

"As part of the critical review, we would like to understand:

a) The consistency, accuracy and level of completeness of the geological information submitted. Is the information submitted with the application consistent with other sources of geological data/research for the Weald Basin?

The geological information of the application is consistent, accurate and sufficiently complete. In particular it is consistent with the definitive published report on the Weald by:

Andrews IJ 2014. The Jurassic Shales of the Weald Basin: geology and oil & gas resource estimates. British Geological a Survey for the Department of Energy & Climate Change. London 79pp.

Swan, G & Munns J. 2003. The Hydrocarbon Prospectivity of Britain's Onshore Basins. Department of Trade & industry. London. 81pp.

The Fernhurst well must regarded as high risk. The reservoir characteristics of the Kimmeridge limestones are unknown. Furthermore the BGS study suggests that only a small thickness of the Kimmeridge Clay source rock may have been heated sufficiently to generate oil."

The bald assertion by Prof. Selley, unsupported by any evidence or argument, that the geological part of the application is "*consistent, accurate and sufficiently complete*" is totally at variance with my submissions:

- **Smythe, D. K. 2014a**. Planning application no. SDNP/1305896/CM by Celtique Energie to drill at Fernhurst, West Sussex: Critique of environmental statement in the context of relevant geology and hydrogeology. Report and objection to SDNPA, 25 January 2014, 60 pp.
- **Smythe, D. K. 2014b**. Planning application no. SDNP/1305896/CM by Celtique Energie to drill at Fernhurst, West Sussex: Additional comments on environmental statement in the context of relevant geology and hydrogeology. Report and objection to SDNPA, 30 June 2014, 34 pp.

It would have been more useful for Prof. Selley to have been asked to comment in detail on these submissions.

The last two sentences, taken together, imply that the Kimmeridge Clay is the source and that the Kimmeridge limestones are the reservoir. This is potentially misleading, because it does not mention the possibility that 'unconventional' treatment of the rocks may well be required (as at Balcombe), for example by acid treatment of the limestone and/or fracking of the larger rock volume including the shales. The basic reservoir characteristics of the limestones are fairly well known, contrary to Prof. Selley's statement. The upper, principal, limestone is 30 m thick at Fernhurst; the limestones have fairly good porosity and very low permeability. In short, they do not constitute a conventional reservoir rock, which, *inter alia*, should have high permeability.

Prof. Selley's last sentence is unclear, and I do not see its relevance. If Prof. Selley is referring to the whole area of the BGS study, then the area identified by the BGS in which mature Kimmeridge Clay occurs is only one-twelfth of the total area studied. But within the latter mature area, most or all of the existing thickness of Kimmeridge Clay is indeed oil-mature; that is, it is likely to have generated oil.

3. Similar geology outside PEDL231 (Selley pp. 2-4)

> "b) To understand the target formations within PEDL 231 and how they compare to those outside the PEDL boundary, but within the Weald Basin. Can the same geology be found outside PEDL 231?

The general geology of the Weald is very well known after nearly 200 years of research. The surface geology has been mapped both by the British Geological Survey, by academic and amateur geologists ***(Figure 1.).***

The subsurface geology of the Weald is less well known, obviously, though many wells have been drilled across the basin since the Netherfield wells in 1875. The Weald is a basin of sedimentary rocks (limestones, sandstones & mud rocks) deposited layer on layer over many millions of years. Surface mapping and subsurface boreholes show that the main rock units are laterally continuous across the Weald ***(Figure 2)*** *though there are some minor changes in rock type across the basin.*

[Figs. 1 and 2 here]

Figure 3 *shows the producing oil & gas fields of the Weald that provide information of known source rocks and reservoirs, and can be used to predict their distribution. Some variations in source rock and reservoir quality have been observed.*

[Fig. 3 here]"

Figure 1, reproduced from a guidebook published by Prof. Selley, is irrelevant because it portrays the landscape evolution of the Weald, involving just the younger rocks from Ashdown Sands upwards, and not the deeper rocks of hydrocarbon interest.

Figure 2 is a 52 km long cross-section. It is potentially more useful than his figure 1, because it implies that the geology is similar from end to end, albeit with the layers of interest being somewhat thicker within the central Weald area. However, Prof. Selley does not discuss this figure.

Prof. Selley then states, regarding figure 3, that there are "*some variations*" in source rock and reservoir quality. But he does not conclude by answering the question, which should have been stated in the affirmative; <u>yes, the same geology is found outside the bounds of PEDL231</u>.

4. Basic terminology (Selley pp. 5-6)

"c) Some of the basics behind petroleum geology and terminology (e.g. Is the Weald Basin a ‘prospect’ and if not, what is the difference between a ‘prospect’ and a ‘target horizon’?)

Figure 4 *is a stratigraphic column that shows the sequence of rocks to be encountered across the Weald. This figure also shows the rock strata which are petroleum reservoirs for the various oil & gas fields of the basin. In the petroleum industry the term ‘****prospect’*** *is applied to a location where*

*it is possible that petroleum may be trapped and that merits testing by drilling. The term '**prospective**' is applied to an area where evidence suggests that petroleum may be trapped.*

[Fig. 4 here]

*Thus the Weald basin is known to be **prospectiv**e because it contains many producing oil and gas fields. A **prospect** has been identified beneath Nine Acre Copse that merits drilling.*

*A **target** horizon is a layer of rock, generally sedimentary (sandstone or limestone), that may contain petroleum, i.e. has the potential to be a petroleum reservoir, i.e. has porosity (pore spaces to hold petroleum) and permeability- the pores are interconnected, enabling petroleum and water to flow through the rock. Thus there are two **targets** for the Nine Acre Copse **prospect**: the Kimmeridge Limestones (Upper Jurassic) and the Great Oolite (Middle Jurassic).*

The Great Oolite is a major petroleum reservoir across the Weald basin producing oil in many fields such as Singleton, Storrington and Lydsey. The Kimmeridge Limestone target is not yet a proven reservoir in the Wessex Basin. This was the target for Cuadrilla's well at Balcombe."

The definitions given here of prospect and target are acceptable. However, Prof. Selley is wrong about the targets for the Fernhurst well. It is agreed that the Kimmeridge Limestones are a target, but he has omitted the Kimmeridge Clay and the Middle Lias shales, and his mention of the Great Oolite is misleading, because it is not a target. The other secondary targets are the Oxford Clay, Kellaways Beds and Cornbrash (see Smythe 2014b, sections 2.4 and 2.5 for a full discussion).

In addition, Prof. Selley's statement that a prospect has been identified beneath Nine Acre Copse, a small locality, is misleading, because the prospect is described by Celtique in a letter to DECC as *"laterally extensive"* (see Smythe 2014b, section 1.4 for a discussion of this letter).

5. Porosity and permeability of the targets

"d) To understand the porosity of the primary target; Kimmeridge Limestone formation and the secondary target; Great Oolite formation and the likelihood of whether the oil would be free flowing or trapped and require fraccing.

*The **Kimmeridge** Clay is a thick formation that is largely composed of mud that contains within it thin layers of fine-grained limestone. The Kimmeridge Clay has a high content of organic matter. This has generated petroleum in some areas, notably the North Sea, where it is the main source rock for North Sea petroleum. Within the Weald basin the thin limestone beds are particularly well*

–developed forming the rock unit that is termed the Kimmeridge Limestone. It must be understood that this is not a bed of solid limestone many metres thick but a sequence of thinly inter-bedded limestones and clays. The limestones have negligible porosity and permeability, but they are brittle and naturally fractured due to earth movements. Petroleum can migrate from the organic rich clay beds into the intervening naturally fractured limestones. Colloquially Americans call this the 'Oreo cooky' model (An oreo cooky is a biscuit composed of alternate layers of crisp biscuit and viscous sugary confection) ***Figure 5.*** *This situation produces petroleum in the Franciscan cherts of California and the Bakken shale of Illinois. When the shale gas industry began in 1821 production was solely from natural fractures. The artificial fracturing of conventional reservoirs (sandstones and limestones) began in the late 19th century. For many decades explosives were used, more recently the less exciting and more environmentally friendly hydraulic fracturing was developed in the 1940's, first for conventional reservoirs and then for shale in the late 20th century.*

[Fig. 5 here]

The ***Great Oolite*** *is a limestone with good porosity and permeability. It is a type of rock that is a major petroleum reservoir around the world and in Arabia in particular. In some cases the carbonate grains are completely un-cemented, but generally there is some mineral cement that infills pores and thus diminishes both porosity and permeability. In such cases the rock is hydraulically fractured and flushed through with acid to leach out some of the carbonate and thus increase flow rate. The Great Oolite is a major producer of petroleum across the Weald basin in the Storrington, Singleton, Humbly Grove, Stockbridge, Baxter's Copse, Lidsey and Goodworth fields (Refer back to Figure 4)."*

Prof. Selley's description of the 'Kimmeridge Limestone' is inaccurate and over-simplified. In the Weald there are two distinct limestones, the I-micrite and the J-micrite, correlatable from well to well throughout the basin. There is also a lower K-micrite of more limited extent, which is not present below the SDNP.

The Bakken play is not in the state of Illinois, as stated by Prof. Selley, but lies some 1200 km to the NW, in NW North Dakota and NE Montana, and continues north of the 49th parallel into Canada. Prof. Selley fails to make explicit that the Bakken, a good analogue to the Kimmeridge limestones of the Weald, was a failure as a conventional oil target between 1952 and 1980, but only began to become productive in oil after the advent of fracking in about 1980.

Prof. Selley's description of the Great Oolite as an important reservoir for the oilfields that have been developed around the fringes of the Weald Basin is irrelevant, because this limestone is *not* a target of the current proposal (Section 4 above). Coring is to be be confined to Middle and Upper Jurassic layers *above* the Great Oolite, and the Middle and Upper Lias *below* the Great Oolite. However, it raises an interesting point, which is that had Celtique been investigating a conventional target at Fernhurst, the Great Oolite would be a prime candidate for detailed investigation by coring. This is additional evidence that the Applicant is seeking unconventional plays.

Prof. Selley has not answered the principal question here, which is whether or not the limestone has low permeability and will need fracking. But he has not provided any new information to suggest that these limestones will contain oil which will flow freely without stimulation.

Oil & Gas
Authority

Oil & Gas Authority
Simon Toole
Kings Building
16 Smith Square
London SW1P 3HQ
T: +44 (0)300 067 1605
E: simon.toole@oga.gsi.gov.uk

Dr Sutcliffe
By email to: jillsutcliffe1@gmail.com

7 July 2016

Dear Dr Sutcliffe,

We received the briefing and copied press release and your letter of 26 June, in which you stated your objection to the extension of licence PEDL234. We are also in receipt of your letter of 28th June to the DECC Correspondence Unit on the subject of the Strategic Environmental Assessment undertaken before the 13th Onshore Licensing Round.

You may now be aware from the notice on our website that we have decided to extend the licence for two years. (see https://www.gov.uk/guidance/oil-and-gas-onshore-exploration-and-production#licensing)

We would like address your *specific points* as follows:

1) *The deadline for licence term extensions passed on 31st May 2016 and so it would be inappropriate if UKOG/KOGL were singled out for preferential treatment.*

There is no question of preferential treatment for any licensee. If licensees propose licence amendments, OGA considers all such requests on their own merits and against the same criteria and policies.

2) Granting an extension would set a precedent for other licence holders to be free to ignore set deadlines and accepted practices. Ad hoc extensions to licence terms would effectively result in licence blocks, including PEDL234, being taken 'off the market' and restrict the ability for open bidding in future licensing rounds. This would have the added effect of reducing the Government's anticipated income from hydrocarbon licensing.

There may be circumstances where a licensee may make substantial progress towards completion of an Initial Term Work Programme but still require more time because of delays that were beyond its control. In such cases, it is reasonable for OGA to consider requests for an extension.

3*) PEDL234 has already been granted two licence term extensions and variations to the agreed programme of works, but these were not carried out. There has been ample*

opportunity to complete the works, but failure to do so on the part of the operator should not be a reason to grant yet another licence term extension, particularly after the deadline for doing so.

In the case of PEDL234, the Licensee has made substantial progress, including constructing the Broadford Bridge wellsite pad after obtaining planning permission. OGA expects that the new extension provides OGUK with the time to progress further with meeting the licence commitments.

4) UKOG/KOGL took ownership of PEDL234 in full knowledge that the licence is due to expire on 30th June 2016. A change of ownership is not a reason to apply for, nor grant, a further licence term extension.

We agree. Of itself a change in ownership is not a justification for a licence extension.

5) In addition, The British Geological Survey (BGS) The Jurassic Shales of the Weald Basin – Geology, Shale Oil and Shale Gas Estimation 23/5/2014 demonstrated that there is NO oil "…identified the potential for a significant volume of oil-mature shale to be present at several horizons in the centre of the Jurassic , but shales further west and on the northern and southern flanks are not considered mature for oil generation (Figure 50)….

The scope of the BGS report was to evaluate the potential of the unconventional resources held within the shale, where horizontal drilling and high volume hydraulic fracturing would be needed to produce hydrocarbons from low permeability rocks. The published resource estimates are for the oil in the pores and trapped in kerogen in the shales which has not already migrated in to other strata. The Horse Hill 1 and Balcombe 1 wells lie within the area where BGS considers the Jurassic Kimmeridge Clay to be mature for oil generation but believe that the Kimmeridge Clay is not likely to have retained moveable oil in significant quantities. However, if natural fractures occur in the limestone intervals within the Kimmeridge, this is a "conventional" reservoir into which the oil will have migrated over geologic time (albeit only a short distance), and was not within the scope of the report (as stated on page 2).

"…concludes that there is no significant Jurassic shale gas potential in the Weald Basin. Even the deepest Lias shales are unlikely to have attached sufficient maturity to allow for significant gas generation.

We agree.

6) The very faulted nature of the Weald is yet another reason why drilling should not take place as it would both risk seismic reactions (as at Preese Hall, Lancs) as stated by Prof Mike Stephenson of the BGS, and, jeopardise the public water supply which emanates from the chalk downs of the South Downs National Park (Graham Warren, hydrogeologist and former Environment Agency staff worker).

Due to tectonic inversion, the central area of the Weald basin has been stripped of the Chalk aquifer and so the Broadford Bridge well site in the southeast of PEDL234 is not coincident with groundwater special protection zones (see below, Figure 51 in the same report mentioned in question 5 with the outline of PEDL234 added).

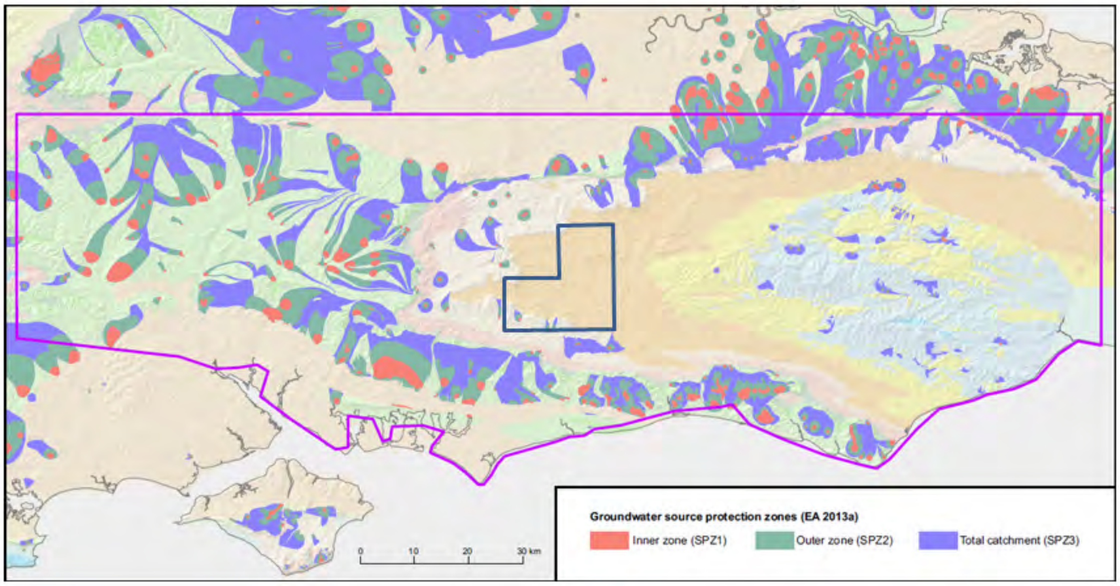

Oil and gas wells routinely intersect faults and there is no evidence that this has resulted in an increased risk to groundwater in the UK because of the rigorous well construction and design requirements of the Health and Safety Executive.

I assume you are also aware that the Infrastructure Act 2015 introduced a range of further requirements that must be met before an operator can carry out hydraulic fracturing. These include the assessment of environmental impacts, groundwater monitoring and community benefits and that associated hydraulic fracturing will not take place at a depth of less than 1000 metres. A hydraulic fracturing consent will not be issued unless the Secretary of State is satisfied that these conditions are met

Hydraulic fracturing into faults has been linked to an induced seismicity. OGA has published a protocol to mitigate against hydraulic fracturing induced seismicity (www.gov.uk/guidance/oil-and-gas-onshore-exploration-and-production#resumption-of-shale-gas-exploration) and part of the Hydraulic Fracture Plan is an assessment of faulting and formation stresses in the area and the risk that the operations could reactivate existing faults.

UKOG who has acquired 100% ownership of the PEDL234 licence have information on their website describing the potential oil resources they believe can be found in the naturally fractured limestone layers within the Jurassic Kimmeridge formation. The interval was tested at the Horse Hill 1 site where the well flowed at significant rates without hydraulic fracturing of the reservoir.

OGA has not received any proposals for high volume hydraulic fracturing in the Weald basin.

You raised a further point in your letter of 28th, the Strategic Environmental Assessment that was undertaken before the issue of that licence. We do not agree that hydrology and public water supply were not properly addressed in that SEA.

Water resources (aquifers, groundwater, surface waters) were addressed in the SEA along with Geology and Soils. The report concluded that the location of surface waters and aquifers (especially those of potable water) were well known and their protection effectively addressed through design standards and approvals processes for exploration, production and export facilities.

Petroleum Exploration and Development Licences (PEDLs) do not give permission for operations. Rather, they grant exclusivity to licensees, in relation to hydrocarbon exploration and extraction (including for shale gas but also for other forms), within a defined area. All operations, such as drilling, fracking or production, however require local planning permission, and applications for permission are subject to public consultation. Access agreement(s) with relevant landowner(s), Environment Agency permits, HSE scrutiny, and Oil and Gas Authority (OGA) consent are also required before operations can commence. Depending on the nature and location of the proposed operations, there may also be public consultation conducted by the Environment Agency on permit applications made to them.

I hope that this information is helpful to you.

Yours sincerely,

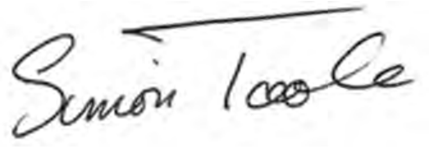

Simon Toole
Director of Licensing and Legal

From: Jones, Rob
To: ENV Development Control; Perigo, Stuart
Subject: BGS comments 20.08.13 to 05.03.0444 Scoping opinion, Grange Rd, Singleton
Date: 20 August 2013 16:50:30

From: Richards, Philip C. [mailto:pcr@bgs.ac.uk]
Sent: 20 August 2013 16:40
To: Jones, Rob
Cc: Mackenzie, Rachel; Hough, Edward
Subject: 05/13/0444 Scoping Opinion re Land South of Grange Road, Singleton IDA 219151 - (4)

Rob, Susan,

Firstly, please accept my apologies for the delay in replying to your original e-mail. I understand that it was never received here, and that the request was resent today.

I am afraid that BGS is not in a position to comment meaningfully on the planning application. The geology summary presented in the application seems reasonable. We are unable to comment on the plans that Cuadrilla propose for the well.

Regards,

Phil Richards
....

Dr Phil Richards
Manager, Hydrocarbons
British Geological Survey
Murchison House
West Mains Rd
Edinburgh, EH9 3LA

Direct Line: +44 1316500283
Mob: +7718339538
www.bgs.ac.uk

This message (and any attachments) is for the recipient only. NERC is subject to the Freedom of Information Act 2000 and the contents of this email and any reply you make may be disclosed by NERC unless it is exempt from release under the Act. Any material supplied to NERC may be stored in an electronic records management system.

CUADRILLA

Cuadrilla Resources Limited
Cuadrilla House Stowe Court Stowe Street Lichfield Staffordshire WS13 6AQ
Tel: +44 (0)1543 266444 Fax: +44 (0)1543 266440

Mr Charles Hendry
Minister of State for Energy
Department of Energy & Climate Change
3 Whitehall Place
London SW1A 2AW

8 February 2012

Dear Minister,

RESUMPTION OF HYDRAULIC FRACTURING IN LANCASHIRE

I am writing to update you on the extensive efforts which we have undertaken to thoroughly investigate the seismic events which took place on 1 April and 27 May 2011, now some ten and eight months ago respectively. Throughout this time we have been working extensively with your officials who have no doubt been keeping you fully informed on the progress with the substantial material we have submitted.

If we are given clearance to proceed, it is our intention to restart hydraulic fracturing operations in a cautious manner, using revised job procedures and a very safe and conservative traffic light system. Our proposals are designed for the present phase of operations, namely to establish the commercial viability of the natural gas resource. During this phase we will learn much more about how these proposals work in practice and we expect to revise them accordingly.

The studies conducted so far also suggest that seismicity will be negligible and can be carefully controlled. That conclusion has recently been publicly echoed by the British Geological Survey and Keele University. We are confident that we can test our new fracture design and traffic light system while maintaining an environment that is safe for the local residents and their property.

For information, the provisional timetable we have shared with your officials is as follows:

- Q1 2012 — Formal end of fracturing suspension (date to be confirmed)
- March 2012 — Community engagement and other preparations for resumption
- April 2012 — Start fracturing and testing of Grange Hill and Becconsall wells
- May 2012 — Commence drilling and testing of three additional wells
- Dec 2012 — Third-party estimate of recoverable reserves
- Q1 2013 — Submission of long-term production test plan to DECC
- Q1 2013 — Initiate long-term test production
- Q4 2013 — Submission of field development plan to DECC

Should there be any remaining issues on which you require any clarification I would be pleased to personally discuss these with you.

Yours sincerely,

Mark Miller
Chief Executive

Registered at: Cuadrilla Resources Limited Cuadrilla House Stowe Court Stowe Street Lichfield Staffordshire WS13 6AQ
Registration No: 06472493
VAT Registration No: 945 6375 88

DEPARTMENT OF ENERGY & CLIMATE CHANGE

Charles Hendry MP
Minister of State
Department of Energy & Climate Change
3 Whitehall Place
London
SW1A 2AW
www.decc.gov.uk

Lord Browne of Madingly, FRS, FREng
Chairman of the Board
Cuadrilla Resources Ltd.
Cuadrilla House
Stowe Court, Stowe Street
Lichfield
Staffs, WS13 6AQ

11 May 2012

Dear John,

I am writing to you in your capacity as the current Chairman of the Board of Cuadrilla Resources.

You will be aware that my Department is concerned that Cuadrilla failed to recognise the significance of the casing deformation experienced in the earth tremor triggered by fracking operations on 1 April 2011. So much so, that the company did not report it to my officials in contemporary discussions as to the possible cause of the tremor and the possibility that it might be linked to the fracking. In the light of Cuadrilla's responses to the Department's subsequent inquiries, I have formed the view that this failure discloses weaknesses in Cuadrilla's performance as a licensee, which need to be addressed.

I would like to discuss these matters with you, your Chief Executive Officer, and the Board representative of AJ Lucas, as soon as may be convenient, to hear what improvements you might propose to address these concerns, and how these might best be implemented.

Perhaps your office could contact my diary manager, to arrange a suitable date.

I very much hope we can resolve this in the near future.

Yours sincerely,

Charles Hendry

CHARLES HENDRY

CUADRILLA
RESOURCES

Cuadrilla Resources Limited
Cuadrilla House Stowe Court Stowe Street Lichfield Staffordshire WS13 6AQ
Tel: +44 (0)1543 266444 Fax: +44 (0)1543 266440

Mr Charles Hendry, MP
Minister for State
Department of Energy and Climate Change
3 Whitehall Place
London
SW1A 2AW

25 May 2012

Dear Charles

Thank you for your letter dated 11 May 2012. I look forward to meeting you on 2 July.

In the meantime I wanted to inform you of several steps we have already taken to enhance Cuadrilla's communications with DECC, as well as its general strength as operator:

- The Board has constituted a new Health and Safety Committee under the chairmanship of Roy Franklin. This committee will have responsibility to oversee the company's health and safety performance and will receive instant reports of any material incidents as they arise, and ensure they are appropriately reviewed and communicated.

- The Board has undertaken to refresh and augment the current management team, including the appointment of a new CEO and the creation of a number of new senior roles such as a Director of Communications. This transition will be complete before the end of the summer.

- The company has established and communicated a clear policy that operational incidents judged to have a potentially serious impact on health and safety or infrastructure integrity will be communicated to DECC immediately, before testing whether such matters are indeed germane. This is as acknowledged by CEO Mark Miller in a letter to on March 16.

I hope these steps demonstrate the Board's continuing commitment to safe operations. I look forward to hearing your views on these at our meeting.

Yours ever,

The Lord Browne of Madingley
Chairman of the Board

creating a better place
for people and wildlife

Environment Agency

Cornerstone Barristers

By email:
estelled@cornerstonebarristers.com

Our ref: CE/LT
Your ref:
Date: 22 July 2019

Dear Ms Dehon

Re: Hydraulic Fracturing Plan for petroleum exploration well Preston New Road – 2, Fylde, Lancashire

Combined response to letters: Harrison Grant dated 14 November 2018 and Cornerstone Barristers dated 12 July 2019.

Thank you for your letter of 12 July 2019.

I can assure you that we have reviewed the report published by Professor Smythe and have considered the concerns raised in November 2018, in the Harrison Grant solicitors' letter to the Environment Agency; namely, points 1 to 5, insofar as they relate to our assessment of the hydraulic fracture plan (HFP). Following your letter of 12 July we have also reviewed the additional concerns raised (points 6 to 9). These are all dealt with in turn below.

The requirements of the environmental permit and associated HFP focus on the protection of groundwater. The European Directive providing this protection is the Water Framework Directive (WFD) [2000/60/EC] and the operation is regulated as a groundwater activity under the Environmental Permitting Regulations 2016 (the Regulations). The relevant condition in the environmental permit states:

2.2.3 The groundwater activity A5 shall be not extend beyond the area edged in red on the site plan at schedule 7 of this permit and shall only be carried out within the Upper and Lower Bowland Shale and Hodder Mudstone Formation. The discharges shall be made at points along the laterals to be drilled in a westerly direction from the vertical boreholes and for a distance not exceeding 2000 metres as listed in table S3.2 (discharge points).

This activity A5, cannot be undertaken by Cuadrilla Bowland Limited (Cuadrilla) until the pre-operational condition requiring the approval of a HFP has been met. This is pre-operational condition PO3, as referenced in permit condition 2.4.4, which states:

The groundwater activity A5 shall not be carried on until the measures specified in PO3, PO5, PO6 and PO7 of schedule 1 table S1.3 have been completed.

Under the WFD a direct discharge to groundwater is prohibited. The water in the Millstone Grit is of exceptionally poor quality, but it is nevertheless groundwater and a discharge into this formation would not be lawful. By continuing to interpret the Millstone Grit as being present around 200m in thickness above the lateral section of PNR 2 wellbore, Cuadrilla is adopting a precautionary approach to their fracturing operations.

If we were to take the view that the Millstone Grit did not exist above the lateral well, then fractures could theoretically extend much further. By referencing the presence of Millstone Grit in the HFP we are satisfied that this is the most precautionary approach to take and will enable the operator to demonstrate compliance with their environmental permit by remaining within the stated permit boundary and minimise the risk of fractures outside the permit boundary and protecting groundwater. Whilst we acknowledge that other interpretations of the data could be valid, we have not requested that Cuadrilla changes the HFP to reflect this on the basis that the approach in the HFP provides the most protection to the environment.
Consideration of points raised in Harrison Grant letter of November 2018:

1, 2 & 3. We have previously challenged the interpretation of the presence of the Millstone Grit above the west end of the lateral. We spent time reviewing the available information and have discussed this with the Oil and Gas Authority (OGA) and Cuadrilla. There is a major reflector in the seismic record that correlates to the Permian Unconformity below the Collyhurst Sandstone and Manchester Marl formations, so if the Millstone Grit is absent from the geological record at this location then it would be reasonable to assume that the Bowland Shale is present here. We have reviewed the risk to the environment from the proposed activities should this formation be absent from the geological record at this location, and we are satisfied that by setting the permit boundary at the level the operator has proposed is conservative, creating a buffer between the permitted limit of the fracture extent and any groundwater bearing formation.

4. The presence of a fault which is actually poorly defined in the 3D seismic data volume has been reviewed together with the hydrogeological evidence in the area of the Wakepark Lake. The Lake is a manmade excavation of the peat that has intercepted groundwater. The hydrogeological evidence indicates that this groundwater is from the Middle Sands aquifer. This is the same aquifer unit that is being monitored in groundwater boreholes by Cuadrilla around the Preston New Road site. Evidence from boreholes in the local area and the British Geological Survey Memoir suggest that the water in the Middle Sands is of sufficient quantity to be the source of water in the Lake. The fact that the lake water is also reported to be fresh and of good quality again supports the Middle Sands as the source and counters the potential that the lake water could be derived from the deeper Upper Sherwood Sandstone.

The data published by the BGS from their recently drilled borehole into the Sherwood Sandstone shows that the groundwater quality at this depth is more saline than seawater. This leads to a hydrogeological conceptualisation that the water in the Wakepark Lake is derived from the shallow sands and not in hydraulic connectivity with the deeper groundwater of the Sherwood Sandstone. So despite the seismic potential of a fault, it is clear from the

hydrogeological evidence that even if the fault is present, and it did link the sandstone with the surface, it is not conducting water upwards to the Wakepark Lake.

5. There is evidence that hydrocarbon is only present below the Manchester Marls in the borehole logs for both previously drilled gas wells in the area, as well as from the new Preston New Road wells. This evidence strongly suggests that it does act as a barrier to upward movement of the gas and acts as a cap rock. Faults may act as pathways or they may form barriers and it appears from the evidence here that the faulting that exists through the Manchester Marl formation is not acting as a pathway and so not conducting hydrocarbons upwards through it.

Consideration of further points raised in your letter:

6. We are aware that there are likely to be such features within the Upper Bowland Shale, which could influence the fracture propagation. The HFP sets out how the operator will monitor and report the fracture growth, and we are satisfied that this is adequate to ensure that Cuadrilla stays in compliance with the permit. The operator showed that they were able to carry out the activity in this way during the PNR1z hydraulic fracturing operations.

7. We have looked at the hydrogeological data for the area and have found no evidence to suggest that any of the surface water features are fed from deep groundwater in the Sherwood Sandstone or the Mercia Mudstones. Water quality in these features tends to accord with what would be expected of shallow drift derived groundwater. Water from deep boreholes drilled over many years around this area has shown that the water quality decreases significantly with depth, as would be expected, and this is supported by the recently published data from BGS showing the Sherwood Sandstone to have a higher salinity than seawater. Evidence from the groundwater monitoring boreholes around the Preston New Road site has shown that groundwater within the Middle Sands deposits is sub-artesian and abundant and is therefore the most reasonable hydrogeological conceptualisation of the source of many of the surface water features in the area. (See also response no. 4)

8. Whilst the interpretation of the PNR-1 fault by Cuadrilla Bowland Limited and Professor David Smythe (12 November 2018) differ, the splays to which you refer are accurately reflected in appendix 2 to the HFP. Both interpretations are based on geophysical data (surface seismic survey) and are subject to the limitations of resolution and human interpretation. We have to decide if these differences are material to whether or not Cuadrilla are allowed to proceed with hydraulic fracturing in well PNR 2.

The difference of opinion concerning the form and structure of the PNR-1 fault is in the Lower and Upper Bowland Formations, which in this region represent the hydrocarbon source rocks. Further, it is not unusual for a wellbore to intersect faults and this is taken into consideration when a well is designed and constructed. Not only is the location of the disputed fault structure above the heel of the well in the vertical section, but Cuadrilla has carried out a fault reactivation study and is committed to not undertaking hydraulic fracturing in proximity to any faults that have been identified as having reactivation potential.

Even if the work of Professor Smythe is assumed to be the most accurate interpretation, the controls in place in the hydraulic fracture plan and the permit will ensure that it would not be unsafe for Cuadrilla to carry out stimulation of well PNR 2.

9. Hydraulic fracture modelling for PNR 2 has been undertaken using proprietary modelling software and the input parameters of the model have been updated for well PNR 2 as evidenced by the inclusion of hybrid gel-slickwater fluid systems.

While it is true that the early stage fracture plots for PNR 1z were mostly to the north, there was also a southerly component to fracture growth. This lateral bias is interesting, but is not fundamental to operational compliance. We require the operator to carry out downhole microseismic monitoring to assess the extent of fracture propagation in order to demonstrate compliance with their environmental permit.

Numerical models are a mathematical representation of the heterogeneous natural environment and require considerable refinement over time through a process of validation and calibration before they can be expected to give ideal results. Exploration at PNR is at a very early stage and we would not yet expect a perfect match between modelled and actual fracture extent. As with PNR 1z, for the PNR 2 well we are requiring the operator to undertake good quality downhole microseismic monitoring to assess the extent of fractures. We have considered the issues in your letter, Harrison Grant's letter and Professor Smythe's report and are of the view that we have fully addressed the concerns raised by Preston New Road Action Group.

As outlined above we are satisfied that Cuadrilla's interpretation of the geological data as set out in the HFP provides an effective basis against which we can ensure protection of the environment and assess compliance with the permit by applying a clear demarcation of the permit boundary and ensuring there is no direct discharge to groundwater beyond this point.

It is therefore not our intention to request any further changes to the HFP in terms of the geological interpretation of the site or to its requirements and are satisfied with this content of the HFP.

Yours sincerely

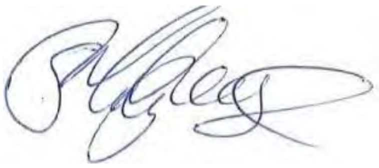

Steve Molyneux
Area Environment Manager

CC: Health and Safety Executive: Paul Bradley, Jim Stancliffe
OGA: Toni Harvey

Your ref:
Our Ref:ZG/IG/EP/PA/04

10th August 2017

[redacted]
Cheshire West and Chester Council
4 Civic Way
Ellesmere Port
Cheshire
CH65 OBE

Dear [redacted],

ELLESMERE PORT WELLSITE
EP-1 WELL TEST
PLANNING APPLICATION

Further to your email of 24th July 2017, we have now had the opportunity to review the existing planning consent for the Ellesmere Port Wellsite.

We note that Planning permission was granted in 2010 by Cheshire West and Chester Council (CW&CC), for the drilling of two (2) boreholes for the purpose of coal bed methane appraisal and production, which for clarity, included the installation of the boreholes, production and power generating facilities and extraction of coal bed methane followed by the restoration of the site. As set out in Section 9.3.6 of the Planning Statement *Nexen October 2009*, the appraisal boreholes would be drilled to an estimated minimum depth of 900m.

For further clarity, the Planning Statement made no reference to the maximum depth of the boreholes. Section 9.3.11 stated that the expected maximum duration of the appraisal drilling would be around thirty (30) days, but could be more or less depending on progress with the drilling and final total depth of the boreholes.

Under the extant planning consent, our client constructed the Ellesmere Port wellsite in 2011 and in 2014 mobilised a drilling rig to the wellsite and subsequently drilled one of two (2) permitted exploratory boreholes (EP-1), a single vertical well to a depth of 1,945m below ground level, with the objective of appraising the geological sequence of the Ellesmere Port prospect, including the coal beds. Following the drilling operation, the EP-1 well was suspended pending a detailed evaluation of the geological information acquired during drilling.

In response to your query, I can confirm that our client did drill the EP-1 well under the extant planning consent, to a depth exceeding 900m, therefore, consistent with the minimum depth of 900m stated within the planning statement and officers report. It is also worth noting that, prior to drilling the EP-1 well in 2014, our client met with representatives of CW&CC and advised those present of its

intention to drill the well beyond 900m. This was to fulfill obligations under the PEDL. The intention was to target the Dinantian limestone, which is a good regional marker within the upper limit of the Lower Carboniferous geological series. Furthermore, it is also worth noting that the EP-1 well was the subject of an application to the Environment Agency, under the Environmental Permitting (England and Wales) Regulations, within which our client clearly set out the objectives of the well, in the context of depth. The application was subject to public consultation prior to the Environment Agency determining the application.

In your query, specific reference is made the Petroleum Exploration and Development Licence (PEDL), under which our client is permitted *'to search, bore for and get petroleum'* within the Licence boundary, which was issued on behalf of the Secretary of State by the Department of Energy and Climate Change (DECC), now the Oil and Gas Authority (OGA), following the 13th Landward Licensing Round. As I understand it, your query relates to the depth of the well, as set out in Schedule 3 of the PEDL.

To clarify any confusion, Schedule 3 of the PEDL relates to a minimum work programme that the Operator applying for the PEDL proposes to undertake, should it be awarded the PEDL. Applications for PEDLs are reviewed by OGA against criteria, which, amongst other things, include technical competence, financial provisions and proposed work commitment. The purpose of undertaking competitive Licensing Rounds is to award the licence to the Operator that promises to maximise the economic recovery of the UK's oil and gas resources. In drilling the EP-1 well to a depth deeper than that set out in Schedule 3 of the PEDL, our client has exceeded its minimum obligation and, consistent with the objectives of the competitive Licensing Rounds, is seeking to maximise economic recovery by comprehensively exploring the oil and gas resource potential underlying the PEDL. For further information and clarity on the PEDL, please contact OGA.

In the context of other Regulators, Planning Practice Guidance sets out the key regulators and their role in hydrocarbon development. The OGA issues Petroleum Licences, gives consent to drill under the Licence once other permissions and approvals are in place. The role of the mineral planning authorities is to grant permission for the location of any wells and wellpads, and impose conditions to ensure that the impact on the use of the land is acceptable.

Both OGA and Environment Agency consented to the drilling of the EP-1 well.

I trust this answers your question, however, should you require any additional clarification please do not hesitate to contact me.

Yours sincerely
for **Island Gas Limited**

[redacted]
